# Stereodivergent assembly of tetrahydro-γ-carbolines via synergistic catalytic asymmetric cascade reaction

**DOI:** 10.1038/s41467-019-13529-z

**Published:** 2019-12-05

**Authors:** Shi-Ming Xu, Liang Wei, Chong Shen, Lu Xiao, Hai-Yan Tao, Chun-Jiang Wang

**Affiliations:** 1 Engineering Research Center of Organosilicon Compounds & Materials, Ministry of Education, College of Chemistry and Molecular Sciences, Wuhan University, Wuhan, 430072 China; 2 State Key Laboratory of Organometallic Chemistry, Shanghai Institute of Organic Chemistry, Shanghai, 230021 China

**Keywords:** Asymmetric catalysis, Synthetic chemistry methodology

## Abstract

Enantiomerically enriched indole-containing heterocycles play a vital role in bioscience, medicine, and chemistry. As one of the most attractive subtypes of indole alkaloids, highly substituted tetrahydro-γ-carbolines are the basic structural unit in many natural products and pharmaceuticals. However, the syntheses of tetrahydro-γ-carbolines with high functionalities from readily available reagents are significant challenging. In particular, the stereodivergent syntheses of tetrahydro-γ-carbolines containing multi-stereogenic centers remain quite difficult. Herein, we report an expedient and stereodivergent assembly of tetrahydro-γ-carbolines with remarkably high levels of stereoselective control in an efficient cascade process from aldimine esters and indolyl allylic carbonates via a synergistic Cu/Ir catalyst system. Control experiments-guided optimization of synergistic catalysts and mechanistic investigations reveal that a stereodivergent allylation reaction and a subsequent highly stereoselective *iso*-Pictet-Spengler cyclization are the key elements to success.

## Abstract (web summary)

Tetrahydro-γ-carbolines are the basic structural unit in many natural products and pharmaceuticals. Here, the authors report a synergistic Cu/Ir system to assemble chiral tetrahydro-γ-carbolines via a stereodivergent allylation followed by a stereoselective iso-Pictet-Spengler cyclization.

## Introduction

**Fig. 1:**
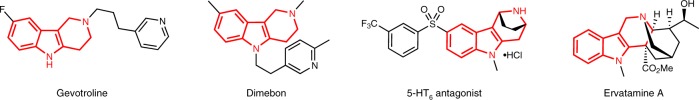
Representative examples of bioactive compounds. Tetrahydro-γ-carboline scaffolds are structural cores in many natural products and pharmaceuticals.

Catalytic asymmetric cascade reaction is recognized as one of the most efficient strategies for the synthesis of enantioenriched molecules^1–3^. The power of catalytic asymmetric cascade process is exceptionally intriguing in qualifying direct transformations of simple and achiral starting materials into complex and chiral compounds incorporating multi-stereogenic centers, as constructions of such frameworks are time-consuming process and normally require multiple synthetic steps. Accordingly, much attention has been paid to the development of such cascade methodologies, where the focus has been on the synthesis of biologically important and highly functionalized heterocycles^4–9^. The indole-containing heterocycles hold a vital role in modern organic chemistry owing to their widely distribution in nature^10–14^, and the tetrahydro-γ-carboline scaffolds are one of the most attractive subtypes of indole alkaloids since they have long been identified as structural cores in many bioactive natural products and pharmaceuticals^15–21^. Representative examples, such as gevotroline, dimebon, tubastatin A, (7*S*,10*R*)-epiminocyclohepta[*b*]indole and ervatamine A^22–27^, all have polycyclic tetrahydro-γ-carboline structures (Fig. 1).

The appealing skeletons and potential biological activities of tetrahydro-γ-carboline skeletons have received much attention among medicinal and synthetic chemists, and a number of approaches to chiral tetrahydro-γ-cabolines have been developed including the classical resolution^28^ and diastereoselective *iso*-Pictet-Spengler cyclization reactions^29–31^. Catalytic asymmetric synthesis of enantiomerically enriched tetrahydro-γ-carbolines was also realized recently. Pioneering study by Jacobsen and co-workers has been carried out on organo-catalyzed enantioselective *iso*-Pictet-Spengler cyclization reaction of isotryptamines and aldehydes^32^. Most recently, Shi and Deng, respectively, identified azomethine ylides as efficient reaction components in chiral Bronsted or chiral Lewis acid-catalyzed cycloadditions for the elegant construction of highly substituted tetrahydro-γ-carbolines with good to high diastereoselectivity and excellent enantioselectivity^33–36^. All those reported catalytic asymmetric cyclization methods have focused on the preparation of only one stereoisomer or its enantiomer, which was rendered by selecting switch between a pair of enantiomeric catalysts. However, no stereodivergent protocol has been developed so far for the construction of biologically active tetrahydro-γ-carboline derivatives bearing multiple stereogenic centers.

**Fig. 2:**
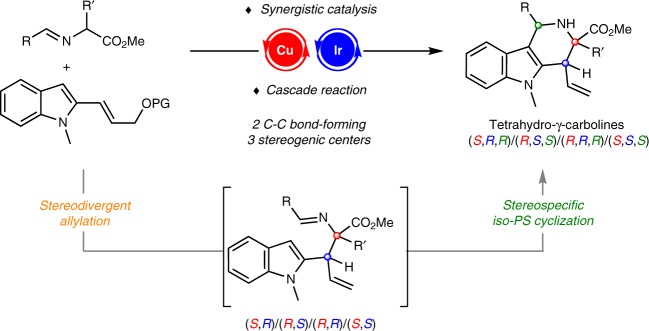
Reaction design. Stereodivergent synthesis via synergistic catalytic asymmetric cascade allylation and *iso*-Pictet-Spengler cyclization reaction.

Stereodivergent synthesis^37–50^ has emerged as a powerful synthetic tool that allows access to stereoisomers of a product incorporating multiple stereogenic centers from the same set of substrates, which exactly caters for the ever-increasing drive in the pharmaceutical industry for molecular complexity in an effort to advance drug discovery and chemical-biology study^51–56^. Based on the utility of such strategy, we^57^ and others^58^ previously developed synergistic Cu/Ir catalyst systems for the stereodivergent access to α,α-disubstituted α-amino acids by the asymmetric allylation of aldimine esters with full control of the diastereoselectivity and enantioselectivity. The current design stemmed from the contemplation of the imino functional group retaining in the allylation products, which might serve as potential electrophilic moiety for a subsequent intramolecular nucleophilic cyclization to construct highly functionalized nitrogen-containing heterocycles. We envisioned that appropriate incorporation of indole moiety in allyl carbonate could facilitate a cascade allylation/*iso*-Pictet-Spengler cyclization, and therefore provides an expedient access to biologically important tetrahydro-γ-carboline derivatives with multiple stereocenters and high functionality. The cascade strategy that combines asymmetric allylation with subsequent intramolecular nucleophilic cyclization imparts three stereogenic centers to the overall two carbon–carbon bond-forming processes and constitutes an intriguing protocol to rapidly build complex molecular architectures from readily available reagents (Fig. 2).

However, several challenging problems need to be considered in this reaction design: (1) the compatibility of indolyl-containing allyl carbonates in our previously developed Cu/Ir dual catalyst system, that is, no deterioration of the stereo-integrity in the initial allylation step; (2) the feasibility of the ensuing intramolecular cyclization under basic reaction condition, which is an essential pre-requisite for promoting the event of azomethine ylide-involved allylation process but inconsistent with the well-established acid-promoted Pictet-Spengler-type cyclization^59,60^; (3) the efficacy of the diastereoselectivity control of the second carbon–carbon bond-forming event in the ensuing *iso*-Pictet-Spengler cyclization with respect to how it is affected by the initially generated two contiguous stereogenic centers in the potential four sets of allylation stereoisomers.

Herein, we reported the development of stereodivergent assembly of tetrahydro-γ-carbolines via a synergistic Cu/Ir catalyst system to achieve a stereodivergent allylation of aldimine esters and indolyl allylic carbonates followed by a spontaneous intramolecular *iso*-Pictet-Spengler cyclization. The stereochemical outcome of the cascade process is remarkable in view of two sequential C–C bond-forming events along with three generated stereogenic centers. The current protocol enables a general process for the predictable preparation of four different stereoisomers of tetrahydro-γ-carbolines at will from readily available starting materials by using four available sets of catalyst permutations.

## Results

### Reaction development and optimization

We began with an investigation of the model reaction between glycine-derived aldimine ester **1a** (serving as Cu-azomethine ylide^61–63^ precursor) and 2-indolyl allyl carbonate **2a** (serving as Ir-π-ally^64^ precursor) in dichloromethane at room temperature under our previously established dual Cu/Ir catalyst system for the stereodivergent α-allylation of aldimine ester^57^. *N*,*N*-Diisopropylethylamine (DIPEA) was chosen as the base in order to suppress the undesired epimerization of the allylation products. With one set of previously utilized catalyst combination [Cu(I)/(*S*,*S*_*p*_)-**L1** + Ir(I)/(*S*,*S*,*S*_*a*_)-**L5**], the designed cascade allylation/*iso*-Pictet-Spengler cyclization occurred smoothly and the desired tetrahydro-γ-carboline 3a was isolated in good yield albeit with a 1:1 diastereoselectivity and acceptable enantioselectivities (91% and 90% ee for the two inseparable diastereomers, respectively) (Table 1, entry 1). Similar stereoselectivity control (1:1 dr with 93% and 97% ee) was also observed when another set of diastereomeric catalyst combination [Cu(I)/(*S*,*S*_*p*_)-**L1** + Ir(I)/(*R*,*R*,*R*_*a*_)-**L5**] was employed to promote this annulation (Table 1, entry 2). Considering two carbon–carbon bond-forming events accompanied by three generated stereogenic centers in this cascade allylation/*iso*-Pictet-Spengler process, we wondered whether the observed lower diastereoselectivity was caused by the coupling of Cu(I)-ylide and Ir(III)-π-allyl species or by the ensuing intramolecular *iso*-Pictet-Spengler cyclization. To investigate the stereoselective control of the allylation step, two control experiments catalyzed with the combination of [chiral Cu-complex + racemic Ir-complex] and [racemic Cu-complex + chiral Ir-complex] were performed, respectively. Employing the combined [Cu/(*S*,*S*_*p*_)-**L1** + Ir/*rac*-**L5**] complexes as the catalyst, the same two diastereomers were generated in good yields with similar diastereoselectivity (1:1 dr) and enantioselectivities (88 and 89% ee) (Table 1, entry 3). On the contrary, using the combined [Cu/*rac*-**L1** + r/(*S*,*S*,*S*_*a*_)-**L5**] complexes as the catalyst, 1.6:1 diastereoselective ratio was observed for the two cycloisomers with only 22 and 15% ee, respectively (Table 1, entry 4). The obtained cycloadducts were revealed as the same two sets of stereoisomers with the crude ^1^H NMR spectra analysis regardless of the different set of catalyst combination utilized in the above four experiments. In consideration of the similar enantioselectivity controls delivered by Cu(I)/(*S*,*S*_*p*_)-**L1** combined respectively with Ir(I)/(*S*,*S*,*S*_*a*_)-**L5**, Ir(I)/(*R*,*R*,*R*_*a*_)-**L5** or Ir(I)/*rac*-**L5**, we surmised that the unsatisfied diastereoselectivity in this cascade process was probably caused by the inferior stereoselectivity control in the first allylation step, which was caused by the indistinctively facial recognition of the in situ-generated electrophilic indole-containing Ir-π-allyl intermediate coordinated with (*S*,*S*,*S*_*a*_)-**L5** or (*R*,*R*,*R*_*a*_)-**L5**, and the ensuing intramolecular cyclization is highly stereoselective transformation. The indiscriminating facial selectivity of the in situ-generated nucleophilic *rac*-Cu(I)-ylide further deteriorated the whole stereochemical outcome and rationalizes the worse diastereoselectivity and enantioselectivity control associated with the catalyst combination of [Cu/*rac*-**L1** + Ir/(*S*,*S*,*S*_*a*_)-**L5**] (Table 1, entry 4). Therefore, in order to improve the diastereoselectivity, we further examined several chiral phosphoramidite ligands in Ir-complex combined with Cu(I)/(*S*,*S*_*p*_)-**L1** complex, as summarized in Table 1. The employment of the chiral ligand (*S*_*a*_,*R*,*R*)-**L6**, a diastereoisomer of (*S*_*a*_,*S*,*S*)-**L5**, delivered 2:7 diastereoselectivity and slightly enhanced enantioselectivities (92% and 99% ee) (Table 1, entry 5). The reactions with Ir-complex coordinated by ligand **L7** or **L8** bearing bulky chiral amine moieties afforded the cycloadducts with a slightly lower or reversed diastereoselectivity (Table 1, entries 6 and 7). To our delight, after screening THQPhos (*R*,*R*_*a*_)-**L9** and (*S*,*R*_*a*_)-**L10** developed by You’s group^65^ (Table 1, entries 8 and 9), we found that the combined [Cu(I)/(*S*,*S*_*p*_)-**L1** + Ir(I)/(*R*,*R*_*a*_)-**L9**] complexes provided the promising results in terms of diastereoselectivity and enantioselectivity (>20:1 dr and 99% ee). Subsequently, a series of chiral Phosferrox ligands with various substituents on the oxazoline ring were further evaluated (Table 1, entries 8 and 10–12), and Cu(I)/**L2**-**L4** complex combined with Ir(I)/(*R*,*R*_*a*_)-**L9** complex all exhibited high catalytic activities with excellent enantioselectivities but slightly lower diastereoselectivities. No reaction occurred when acyl protected cinnamyl alcohol was employed as the π-ally precursor, and *tert*-butyl carbonate was also proved to be unsuccessful in terms of yield and diastereoselectivity (Table 1, entries 13 and 14). Finally, two additional control experiments executed in the absence of either Cu(I)/**L1** or Ir(I)/**L9** are proved to be nonreactive, which verifies the superiority of synergistic catalysis in this cascade transformation (entries 15 and 16).

**Table 1:** Optimization of reaction conditions^a^.


Entry	L for Cu	L for Ir	PG	Yield (%)^b^	dr^c^	ee (%)^d^
1	(*S*,*S*_*p*_)-**L1**	(*S*_*a*_,*S*,*S*)-**L5**	CO_2_Me	95	1:1	91 (90)
2	(*S*,*S*_*p*_)-**L1**	(*R*_*a*_,*R*,*R*)-**L5**	CO_2_Me	96	1:1	93 (97)
3	(*S*,*S*_*p*_)-**L1**	*rac*-**L5**	CO_2_Me	95	1:1	88 (89)
4	*rac*-**L1**	(*S*_*a*_,*S*,*S*)-**L5**	CO_2_Me	94	1.6:1	22 (15)
5	(*S*,*S*_*p*_)-**L1**	(*S*_*a*_,*R*,*R*)-**L6**	CO_2_Me	92	2:7	92 (99)
6	(*S*,*S*_*p*_)-**L1**	(*S*_*a*_,*S*,*S*)-**L7**	CO_2_Me	90	1:1	98 (98)
7	(*S*,*S*_*p*_)-**L1**	(*S*_*a*_,*S*,*S*)-**L8**	CO_2_Me	94	3:2	99 (93)
8	(*S*,*S*_*p*_)-**L1**	(*R*,*R*_*a*_)-**L9**	CO_2_Me	98	>20:1	99
9	(*S*,*S*_*p*_)-**L1**	(*S*,*R*_*a*_)-**L10**	CO_2_Me	96	9:1	99 (63)
10	(*S*,*S*_*p*_)-**L2**	(*R*,*R*_*a*_)-**L9**	CO_2_Me	95	5:1	99 (97)
11	(*S*,*S*_*p*_)-**L3**	(*R*,*R*_*a*_)-**L9**	CO_2_Me	89	10:1	99 (89)
12	(*S*,*S*_*p*_)-**L4**	(*R*,*R*_*a*_)-**L9**	CO_2_Me	90	9:1	99 (94)
13	(*S*,*S*_*p*_)-**L1**	(*R*,*R*_*a*_)-**L9**	Ac	n.r.	-	-
14	(*S*,*S*_*p*_)-**L1**	(*R*,*R*_*a*_)-**L9**	Boc	30	12:1	99
15^*e*^	-	(*R*,*R*_*a*_)-**L9**	CO_2_Me	n.r.	-	-
16^*f*^	(*S*,*S*_*p*_)-**L1**	-	CO_2_Me	n.r.	-	-

^a^All reactions were carried out with 0.30 mmol of **1a**, 0.20 mmol of **2** and 0.40 mmol of DIPEA in 2 mL of CH_2_Cl_2_ at room temperature (see Supplementary Methods for the detailed preparation of Cu/**L** and Ir(I)/**L** complexes). Cu(I) = Cu(MeCN)_4_BF_4_, Ir(I) = [Ir(COD)Cl]_2_

^b^Yields refer to the isolated products after chromatographic purification

^c^The dr value was determined by the crude ^1^H NMR

^d^The ee value was determined by HPLC analysis

^e^Without Cu(I)/(*S*,*S*_*p*_)-**L1**

^f^Without Ir(I)/(*R*,*R*_*a*_)-**L9**

### Substrate scope

With the optimized reaction conditions in hand, we then decided to explore various aldimine esters to examine the generality of this cascade process with the combined [Cu(I)/(*S*,*S*_*p*_)-**L1** + Ir(I)/(*R*,*R*_*a*_)-**L9**] complexes as the dual catalysts. The representative results are shown in Table 2. Various aldimine esters derived from glycinate bearing electron-deficient (*para*-Cl, *ortho*-Cl), electron-neutral, and electron-rich substituents (*para*-Me, *meta*-Me, *ortho*-Me) on the aromatic ring reacted smoothly with 2-indoly allylic carbonate **2a**, affording the corresponding tetrahydro-γ-carbolines in high yields (78–99%), exclusive diastereoselectivity (>20:1 dr) and almost perfect enantioselectivities (99% ee) (Table 2, entries 1–6). The substitution pattern of the arene had no negative effect on the stereoselectivity of this cascade reaction, and *ortho*-chloro and *ortho*-methyl substituted aldimino ester **1b** and **1f** were well tolerated in this annulation delivering the desired tetrahydro-γ-carbolines **3b** and **3f** in good yields with excellent enantioselectivities (Table 2, entries 2 and 6). Encouraged by the excellent results for less sterically congested α-nonsubstituted aldimine esters derived from glycinate, we then investigated the performance of more challenging α-substituted aldimine ester in this annulation process, from which a *N*-quaternary stereogenic center can be generated along with two tertiary stereogenic centers in the heterocyclic rings. Switching the base from DIPEA to Cs_2_CO_3_ under otherwise similar reaction conditions, we were particularly delighted to find that a wide array of aldimine esters derived from (±)-alanine have proved to be viable ylide precursors, affording the corresponding cycloadducts in good to high yields with exclusive diastereoselectivities and excellent enantioselectivities, regardless of electronic properties and positions of the substituted groups on the arene ring (Table 2, entries 7–15). Furthermore, aldimine esters bearing heteroaromatic groups underwent this transformation successfully as fused 2-naphthylaldehyde derived aldimine ester, affording the desired cycloadducts in good to high yields with excellent stereoselective control (Table 2, entries 16–18). In addition, aldimine ester **1s** bearing cinnamyl group was proven to be a viable substrate, affording satisfied yield and stereoselectivity (Table 2, entry 19). Importantly, less-reactive alkyl imino esters **1t** derived from valeraldehyde was also tolerated in this reaction, resulting in the corresponding adduct **3t** in good yield with excellent diastereoselectivity and enantioselectivity (Table 2, entry 20). Aldimine esters derived from other α-substituted amino esters have also been investigated. Under the above reaction conditions, satisfied yields and exclusive diastereoselectivities and excellent enantioselectivities were consistently obtained for (±)-2-aminobutyric acid, (±)-phenylalanine, and (±)-glutamic acid derived aldimine esters (Table 2, entries 22–24). Notably, (±)-homoserine-derived cyclic aldimine ester **1y** also worked well in this cascade reaction (81% yield, >20:1 dr and 99% ee), affording the corresponding highly functionalized spiro heterocycle **3y** incorporating both biologically important tetrahydro-γ-carboline^15–21^ and γ-butyrolactone^66,67^ moieties (Table 2, entry 25).

**Table 2:** Substrate scope of aldimine esters^a^.

^a^All reactions were carried out with 0.30 mmol of **1**, 0.20 mmol of **2a** in 2 mL of CH_2_Cl_2_ at room temperature for 6–10 h

^b^Yields refer to the isolated products after chromatographic purification

^c^The dr value was determined by the crude ^1^H NMR

^d^The ee value was determined by HPLC analysis

^e^Cs_2_CO_3_ was used as the base

^f^*tert*-Butyl aldimine ester was used

Next, we examined the generality of this asymmetric cascade allylation/*iso*-Pictet-Spengler cyclization process with respect to 2-indolyl allyl carbonates. The representative results are summarized in Table 3. Reactions of various 2-indolyl allyl carbonates containing *N*-methyl or *N*-benzyl group all provided the corresponding cycloadducts in good yields (80–96%) with high diastereoselectivities (12:1–>20:1) and excellent enantioselectivities (99% ee) (Table 3, entries 1–9). Meanwhile, the cascade process also proved to be almost unbiased toward either electron property (electron-deficient, -rich or -neutral) or substitution position (C-4, -5, -6, or -7) on the indole core, and the corresponding cycloadducts were obtained in good yields with excellent stereoselectivities. However, no reaction occurred when 2-indolyl allyl carbonate bearing methyl group at C3 position on the indole ring probably owing to the steric congestion. (*E*)-methyl (3-(1-methyl-1*H*-pyrrol-3-yl)allyl) carbonate, in which indole ring is replaced by pyrrole one, was also tested under the standard reaction conditions, and the reaction became messy and no desired product was observed.

**Table 3:** Substrate scope of 2-indolyl alyl carbonates^a^.


Entry	R	R'	PG	(1*R*,3*S*,4*R*)-3	yield (%)^b^	dr^c^	ee (%)^d^
1	H	5-Me	Me	**3z**	92	11:1	99
2	H	5-Cl	Me	**3A**	96	14:1	99
3	Me	4-Me	Me	**3B**	85	>20:1	99
4	Me	5-Me	Me	**3C**	84	>20:1	99
5	Me	6-Me	Me	**3D**	88	>20:1	99
6	Me	7-Me	Me	**3E**	95	>20:1	99
7	Me	5-Cl	Me	**3F**	93	>20:1	99
8	Me	5-Br	Me	**3G**	80	>20:1	99
9	Me	H	Bn	**3H**	95	>20:1	99

^a^All reactions were carried out with 0.30 mmol of **1**, 0.20 mmol of **2** in 2 mL of CH_2_Cl_2_ at room temperature for 6–10 h

^b^Yields refer to the isolated products after chromatographic purification

^c^The dr value was determined by the ^1^H NMR

^d^The ee value was determined by HPLC analysis

After investigating the generality of this asymmetric cascade allylation/*iso*-Pictet-Spengler cyclization with the set of [Cu(I)/(*S*,*S*_*p*_)-**L1** + Ir(I)/(*R*,*R*_*a*_)-**L9**] catalyst, we further explored the performance of the other three sets of catalyst combinations with 2-indolyl allylic carbonate **2a** under otherwise similar reaction conditions. Aldimine esters **1a**, **1c**, **1d**, and **1g** were chosen as the representative ylide precursors. As tabulated in Table 4, with the full four sets of [Cu/**L1** + Ir/**L9**] combinations, in each case, four stereoisomeric cycloadducts containing three stereogenic centers were generated in good yields with high diastereoselectivity and excellent enantioselectivities via a ligand-controlled stereodivergent allylation followed by a spontaneous stereoselective intramolecular *iso*-Pictet-Spengler cyclization. The absolute configuration of cycloadduct **3g** and its enantiomer using [Cu(I)/(*R*,*R*_*p*_)-**L1** + Ir(I)/(*S*,*S*_*a*_)-**L9**] or [Cu(I)/(*S*,*S*_*p*_)-**L1** + Ir(I)/(*R*,*R*_*a*_)-**L9**] as the catalyst combinations was unequivocally determined as (1*S*,3*R*,4*S*) and (1*R*,3*S*,4*R*) by X-ray diffraction analysis, respectively; the absolute configuration of cycloadduct **3c** and its enantiomer using [Cu(I)/(*S*,*S*_*p*_)-**L1** + Ir(I)/(*S*,*S*_*a*_)-**L9**] or [Cu(I)/(*R*,*R*_*p*_)-**L1** + Ir(I)/(*R*,*R*_*a*_)-**L9**] as the catalyst combinations were unequivocally determined as (1*S*,3*S*,4*S*) and (1*R*,3*R*,4*R*) by X-ray diffraction analysis, respectively.

**Table 4:** Representative examples of stereodivergent synthesis of four stereoisomers from reaction of 2-indolyl allyl carbonates with aldimine esters^a^.

^a^All reactions were carried out with 0.30 mmol of **1** and 0.20 mmol of **2a** in 2 mL of CH_2_Cl_2_ at room temperature. Yields refer to the isolated products after chromatographic purification. The dr value was determined by the crude ^1^H NMR. The ee value was determined by HPLC analysis

### Gram scale and synthetic applications

**Fig. 3:**
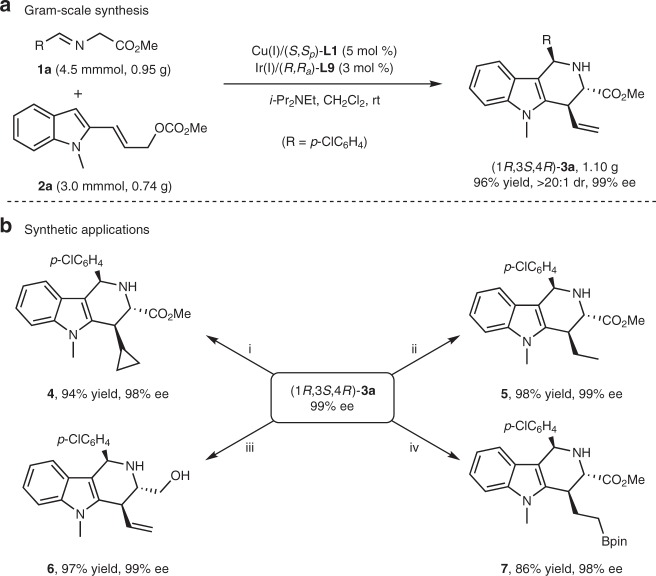
Gram-scale synthesis and derivatization of the cycloadduct **3a**. **a** Gram-scale reaction. **b** Synthetic applications. i: Pd(OAc)_2_ (1 mol %), CH_2_N_2_, Et_2_O, −20 °C; ii: Pd/C (catal.), H_2_ (1 atm), MeOH, room temperature; iii: DIBAL-H, THF, −40 °C to rt; iv: [Ir(COD)Cl]_2_ (3 mol %), dppm (6 mol %), HBpin, CH_2_Cl_2_, rt.

To highlight the synthetic utility of this asymmetric cascade reaction, a gram-scale synthesis of (1*R*,3*S*,4*R*)-**3a** was performed under the standard conditions with the combined [Cu(I)/(*S*,*S*_*p*_)-**L1** + Ir(I)/(*R*,*R*_*a*_)-**L9**] complexes as the co-catalysts, and comparable yields were obtained without loss of diastereoselectivity and enantioselectivity as shown in Fig. 3a. The tetrahydro-γ-carboline derivatives obtained herein can be readily converted into synthetically useful compounds in a facile manner. For example, subjecting **3a** to a Pd(II)-catalyzed cyclopropanation with diazomethane provided compound **4** in 94% yield and with maintained stereoselectivity. Exposure of **3a** to usual hydrogenation conditions (H_2_, Pd/C in methanol) at room temperature afforded compound **5** in 98% yield without erosion of stereoselectivity. In addition, reduction of **3a** with DIBAL-H furnished the amino-alcohol compound **6** in 97% yield without loss of enantioselectivity. Hydroboration of terminal alkene in **3a** catalyzed by Ir/dppm complex afforded the borate **7** in 86% yield^68^. These elaborations clearly demonstrate the utility of the asymmetric cascade allylation/*iso*-Pictet-Spengler cyclization as an expedient method to synthesize various optically active tetrahydro-γ-carboline derivatives (Fig. 3b).

### Mechanism explanations and control experiments

**Fig. 4:**
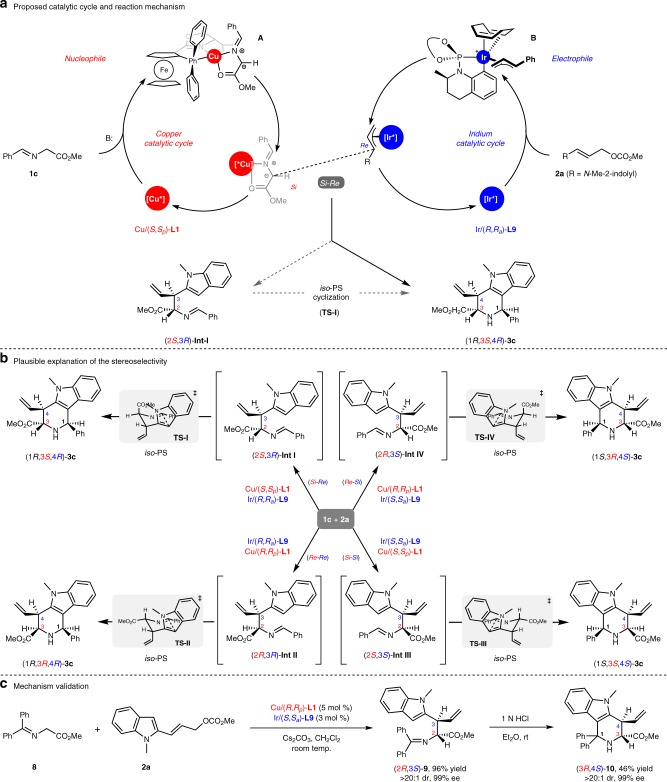
Proposed mechanism with experimental validation and rationalization of the stereoselectivity. **a** Proposed catalytic cycle. **b** Plausible explanation of the stereoselectivity. **c** Mechanism validation.

A full mechanism involved two catalytic cycles was proposed for this cascade allylation/*iso*-Pictet-Spengler cyclization with the set of catalyst combination of [Cu(I)/(*S*,*S*_*p*_)-**L1** + Ir(I)/(*R*,*R*_*a*_)-**L9**]. As shown in Fig. 4a, the nucleophilic Cu(I)-azomethine ylide (**A**) coordinated by chiral ligand (*S*,*S*_*p*_)-**L1** was in situ formed under basic condition. The orientation of Cu(I)-ylide (**A**) was supported by the reported DFT calculations^69^, and its *Re*-face was blocked by the isopropyl group on the oxazoline ring. Meanwhile, the electrophilic Ir(III)-π-allyl species (**B**) was generated by 2-indolyl allyl carbonate (**2a**) and Ir/(*R*,*R*_*a*_)-**L9** complex through decarboxylative oxidative addition. The orientation of Ir(III)-π-allyl intermediate (**B**) was revealed by the reported X-ray structure of the related metallacyclic allyl iridium complex^65^, and its *Si*-face was shielded by the cyclometallated moiety and blocks the approach of the aforementioned Cu(I)-ylide **A**. Thus, the two catalytic cycles merge via a preferential nucleophilic addition of the *Si*-face of the Cu(I)-ylide species to the *Re*-face of the Ir(III)-π-allyl species (*Si*-*Re*), in which the two distinct metal complexes exert almost absolute stereoselective control over the two active intermediates, leads to the allylation intermediate (2*S*,3*R*)-**Int-I** and regenerates both catalysts. Based on the relative and absolute configuration of the final cycloadduct, it is believed that the ensuing intramolecular nucleophilic attack of the indole C3 position to the *Si*-face of imine moiety in highly stereoselective manner followed by deprotonation (*iso*-Pictet-Spengler cyclization) delivers tetrahydro-γ-carboline 3c with (1*R*,3*S*,4*R*)-configuration, in which the diastereoselectivity of intramolecular cyclization is harnessed likely through a highly ordered chair-like Zimmerman-Traxler transition state **TS-I**^70^. The highly stereoselective *iso*-Pictet-Spengler cyclization of allylation intermediates (2*R*,3*R*)-**Int-II**, (2*S*,3*S*)-**Int-III**, and (2*R*,3*S*)-**Int-IV** correlated with the other three set of catalyst combinations, could also be rationalized via similar Zimmerman-Traxler transition states **TS-II-IV** as shown in Fig. 4b, respectively. The electrophilicity of the imine moiety in the allylation intermediates might be further enhanced via a coordination of copper cation, which facilitates the ensuing intramolecular cyclization.

To gain insight into the mechanism and stereoselective control of this cascade reaction, control experiments were further performed to validate the key allylation intermediate. The attempt to directly isolate the allylation intermediate through interrupting the reaction at incomplete conversion failed, probably due to the intrinsically preferred intramolecular cyclization. Then, benzophenone derived ketimine ester **8** instead of aldimine ester was utilized in order to slow or suppress the ensuing intramolecular cyclization with the hope of enhancing the steric hindrance of the imine moiety to reduce its electrophilicity. To our delight, the reaction of ketimine ester **8** under standard reaction conditions with the combined [Cu(I)/(*R*,*R*_*p*_)-**L1** + Ir(I)/(*S*,*S*_*a*_)-**L9**] complexes, did stall after the first C-C bond-forming step, and the branched allylation intermediate (2*R*,3*S*)-**9** was accumulated in high yield with excellent stereoselectivity without further intramolecular cyclization to the tetrahydro-γ-carboline under the optimal reaction conditions. Notably, an acid-promoted intramolecular *iso*-Pictet-Spengler cyclization of the allylation intermediate (2*R*,3*S*)-**9** occurred smoothly, and successfully delivered (3*R*,4*S*)-tetrahydro-γ-carboline **10** in moderate yield without erosion of the diastereoselectivity and enantioselectivity (Fig. 4c). The direct isolation of allylation intermediate (2*R*,3*S*)-**9** and the proton-mediated further cyclization explicitly supported the proposed cascade reaction pathway incorporating sequential stereodivergent allylation and highly stereoselective intramolecular cyclization.

## Discussion

In summary, we have successfully developed a synergistic Cu/Ir-catalyzed asymmetric cascade allylation/*iso*-Pictet-Spengler cyclization reaction with aldimine esters and indolyl allylic carbonates. Control experiments-guided development of a dual metallic catalytic system allows stereoselective control over in situ formed Cu(I)-ylide and Ir(III)-π-ally species independently. Consequently, the desired cascade reaction is established as the stereodivergent allylation followed by subsequent highly stereoselective *iso*-Pictet-Spengler cyclization while tolerating a wide range of variations including both the ylide precursors and the allyl precursors. The cascade process presented herein opens up a conceptually novel prospect for the stereoselective construction of highly functionalized tetrahydro-γ-carboline, a valuable core structure for drug discovery, up to four stereoisomers with predictable stereoselective control.

## Methods

### General reaction procedure

A flame dried Schlenk tube **A** was cooled to room temperature and filled with N_2_. To this flask were added [Ir(COD)Cl]_2_ (0.003 mmol, 1.5 mol %), (*R*,*R*_*a*_)-Me-THQphos-**L9** (0.006 mmol, 3.0 mol %), degassed THF (0.5 mL) and degassed *n*-propylamine (0.5 mL). The reaction mixture was heated at 50 °C for 30 min and then the volatile solvents were removed under vacuum to gain a pale yellow solid. Meanwhile, Cu(MeCN)_4_BF_4_ (0.01 mmol, 5 mol %) and (*S*,*S*_*p*_)-^*i*^Pr-Phosferrox-**L1** (0.011 mmol, 5.5 mol %) were dissolved in 1.0 mL of CH_2_Cl_2_ in a Schlenk tube **B**, and stirred at room temperature for ~ 30 min. Indole-derived allylic carbonates (0.20 mmol), aldimine esters (0.30 mmol), base (0.40 mmol DIPEA for glycine-derived aldimine esters and 0.40 mmol Cs_2_CO_3_ for α-substituted aldimine esters) and CH_2_Cl_2_ (1.0 mL) were added into the Schlenk tube **A** and filled with N_2_. The Cu/**L1** complex solution was then transferred from the Schlenk tube **B** to the Schlenk tube **A** via syringe. Finally, the reaction mixture was continuously stirred at room temperature under N_2_ atmosphere. Although the starting material was consumed (monitored by thin layer chromatography), the reaction mixture was quenched by adding 1 m HCl aqueous solution (2.0 mL) and stirring vigorously for 1 min. The organic layers were separated, and the aqueous layer was extracted with CH_2_Cl_2_ (5.0 mL × 2). The organic layer was combined, washed with saturated brine (10 mL) and dried over anhydrous Na_2_SO_4_. The organic solvent was removed by rotary evaporation to obtain a crude mixture, which was purified by flash column chromatography to give the pure product. The dr value was determined by ^1^H NMR spectrum of the product, and the enantiomeric excess was recorded by HPLC analysis in comparison with the racemic sample.

## Supplementary information

Supplementary Information

## Data Availability

Experimental procedures and characterization data are available within this article and its Supplementary Information. Data are also available from the corresponding author on request. The X-ray crystallographic coordinates for the structures of compounds (1*S*,3*R*,4*S*)-**3g**, (1*R*,3*S*,4*R*)-**3g**, (1*S*,3*S*,4*S*)-**3c**, and (1*R*,3*R*,4*R*)-**3c** reported in this study have been deposited at the Cambridge Crystallographic Data Centre (CCDC), under deposition numbers 1915538, 1915539, 1915540, and 1915541. These data can be obtained free of charge from The Cambridge Crystallographic Data Centre via www.ccdc.cam.ac.uk/data_request/cif.
