# Supplementary material for: Stereodivergent assembly of tetrahydro-γ-carbolines via synergistic catalytic asymmetric cascade reaction
Source: Nat Commun. 2019 Dec 5;10:5553. doi: 10.1038/s41467-019-13529-z (PMC6895234; doi:10.1038/s41467-019-13529-z)
Supplement: Supplementary file 1 — Supplementary Information [file 41467_2019_13529_MOESM1_ESM.pdf]

Supplementary Information for

Stereodivergent assembly of tetrahydro-γ-carbolines *via* synergistic catalytic asymmetric cascade reaction

Xu *et al.*

Supplementary Methods

General information: Unless otherwise stated, all reactions were set up under nitrogen atmosphere in oven-dried glassware using standard Schlenk techniques, monitored by TLC with silica-gel coated plates and purified by flash column chromatography. Commercially available reagents were used without further purification. Solvents were purified prior to use according to the standard methods. Aldimine esters[1], 2-indolyl allyl carbonates[2], chiral ligands **L1**-**L4**[3] and **L5**-**L7**[4] were prepared according to the literature procedure. ^{1}H NMR spectra were recorded on a Bruker 400 MHz spectrometer in $CDCl_3$. Chemical shifts are reported in ppm with the internal TMS signal at 0.0 ppm as a standard. The data are reported as (s = single, d = double, t = triple, q = quarte, m = multiple or unresolved, brs = broad single, coupling constant(s) in Hz, integration). ^{13}C NMR spectra were recorded on a Bruker 100 MHz spectrometer in $CDCl_3$. Chemical shifts are reported in ppm with the internal chloroform signal at 77.0 ppm as a standard. Enantiomeric excess values were determined by HPLC analysis employing AS-H, AD-H, IA, IC, ID, IE and OD-H chiral columns, using hexane and *i*-propanol as solvents. The racemic products were obtained by running reactions with racemic catalysts or blending equal amount of two enantiomers. The absolute configurations of compound **3a** and **3g** were determined unequivocally according to the X-ray diffraction analysis, and those of other tetrahydro-γ-carbolines were deduced on the basis of these results.

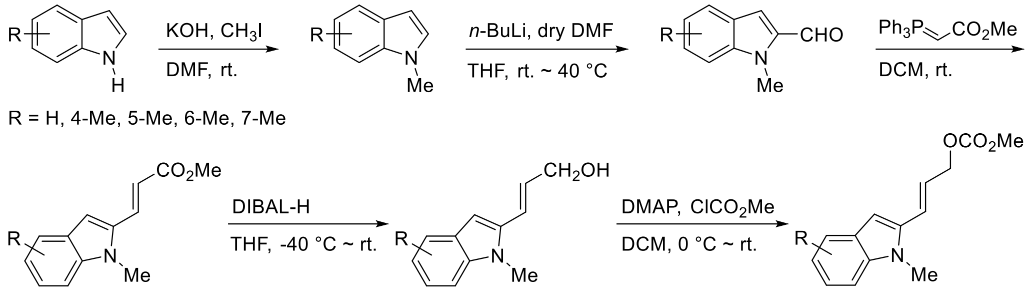

Supplementary Figure 1. Synthetic procedure **A** for substrate **2**. Pathway for the synthesis of allyl carbonates started from unsubstituted and methyl-substituted indoles (**2a-2e**).

Step A1: To a DMF (100 mL) solution of indole (50 mmol) was added KOH (100 mmol) at 25 ºC. The reaction mixture was stirred at the same temperature for 30 min. Then CH_3I (75 mmol) was added *via* syringe. The reaction mixture was stirred at 25 ºC until complete consumption of starting material (detected by TLC). Then the reaction was quenched with water and extracted with EA (× 3). The organic layer was combined, washed with brine and dried over Na_2SO_4. After filtration and evaporation, the residue was purified by a flash column chromatography (PE/EA = 10/1) to provide 1-methyl-1*H*-indole (6.24 g, 95% yield) as a colorless oil.

Step A2: Under nitrogen atmosphere, to a solution of 1-methyl-1*H*-indole (6.24 g, 47.5 mmol) in anhydrous THF (50 mL) was added *n*-BuLi (2.4 M, 25 mL, 60 mmol) dropwise at 25 ºC. The mixture was heated to 40 ºC for 3 h and cooled to 25 ºC, followed by the addition of anhydrous DMF (5.8 mL, 75 mmol) dropwise. The mixture was then stirred at 40 ºC until complete consumption of starting material (detected by TLC). After quenching with a saturated solution of NH_4Cl, the reaction mixture was extracted with EA (× 3). The organic layer was combined, washed with brine and dried over Na_2SO_4. After filtration and evaporation, the residue was purified by a flash column chromatography (PE/EA = 10/1, with 1% Et_3N) to afford 1-methyl-1*H*-indole-2-carbaldehyde (5.92 g, 78% yield) as a yellow solid.

Step A3: To a solution of 1-methyl-1*H*-indole-2-carbaldehyde (5.92 g, 37.2 mmol) in CH_2Cl_2 (80 mL) was added phosphorus ylide (13.36 g, 40 mmol) in one portion at 25 ºC. The reaction was then stirred at 25 ºC until complete consumption of starting material (detected by TLC). The mixture was concentrated under reduced pressure, and the residue was purified by a flash column chromatography (PE/EA = 5/1, with 1% Et_3N) to provide methyl (*E*)-3-(1-methyl-1*H*-indol-2-yl)acrylate (7.95 g, 99%

yield) as a light yellow solid.

Step A4: Under nitrogen atmosphere, to a solution of methyl (*E*)-3-(1-methyl-1*H*-indol-2-yl)acrylate (4.3 g, 20 mmol) in anhydrous THF (40 mL) was added DIBAL-H (1.0 M, 50 mL, 50 mmol) dropwise at -40 ºC. After stirring at -40 ºC for 30 min, the reaction was then moved into 25 ºC and continuously stirred until complete consumption of starting material (detected by TLC). The reaction mixture was quenched with 2.0 M NaOH (aq.), extracted with EA (× 3) and filtered through celite to remove the colloid. The organic layer was combined, washed with brine, dried over Na_2SO_4 before evaporation. Then the residue was purified by a flash column chromatography (PE/EA = 3/1, with 1% Et_3N) to afford (*E*)-3-(1-methyl-1*H*-indol-2-yl)prop-2-en-1-ol (3.65 g, 98% yield) as a yellow solid.

Step A5: DMAP (3.66 g, 30 mmol) and (*E*)-3-(1-methyl-1*H*-indol-2-yl)prop-2-en-1-ol (3.65 g, 19.5 mmol) were dissolved in CH_2Cl_2 (40 mL) before cooled to 0 ºC. Then methyl chlorocarbonate (1.93 mL, 25 mmol) was added dropwise *via* syringe at the same temperture. After stirring at 0 ºC for 30 min, the reaction was then moved into 25 ºC and continuously stirred until complete consumption of starting material (detected by TLC). Then the reaction mixture was quenched with a saturated solution of $NaHCO_3$ and extracted with CH_2Cl_2 (× 3). The organic layer was combined, washed with brine and dried over Na_2SO_4. After filtration and evaporation, the residue was purified by a flash column chromatography (PE/EA = 10/1, with 1% Et_3N to PE/EA = 5/1, with 1% Et_3N) to provide (*E*)-methyl (3-(1-methyl-1*H*-indol-2-yl)allyl) carbonate **2a** (4.50 g, 94% yield) as a white solid.

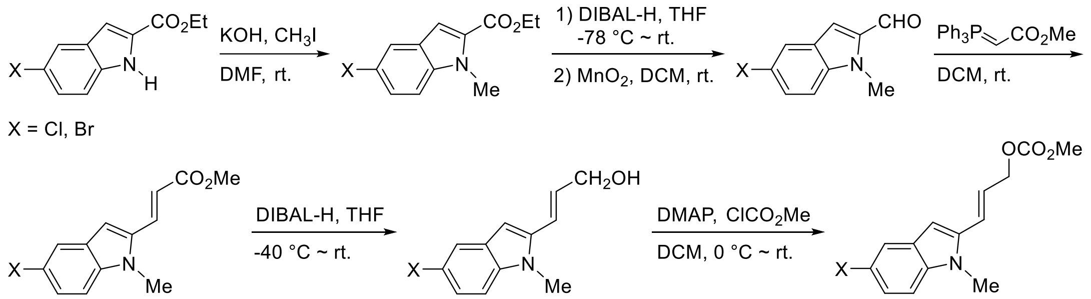

Supplementary Figure 2. Synthetic procedure **B** for substrate **2**. Pathway for the synthesis of allyl carbonates started from ethyl 5-chloro-2-indolecarboxylate and ethyl 5-bromo-2-indolecarboxylate (**2f** and **2g**).

Step B1 (10 mmol starting material) followed the similar procedure as **Step A1** above to provide

ethyl 5-chloro-1-methyl-1*H*-indole-2-carboxylate (2.33 g, 98% yield) as a white solid.

Step B2: Under nitrogen atmosphere, to a solution of 5-chloro-1-methyl-1*H*-indole-2-carboxylate (2.33 g, 9.8 mmol) in anhydrous THF (20 mL) was added DIBAL-H (1.0 M, 25 mL, 25 mmol) dropwise at -78 ºC. After stirring at -78 ºC for 30 min, the reaction was then moved into 25 ºC and continuously stirred until complete consumption of starting material (detected by TLC). The reaction mixture was quenched with 2.0 M NaOH (aq.), extracted with EA (× 3) and filtered through celite to remove the colloid. The organic layer was combined, washed with brine, dried over Na_2SO_4 before evaporation. Then the crude material was dissolved in CH_2Cl_2 (50 mL) along with fresh MnO_2 (100 mmol) and stirred vigorously until complete consumption of the crude material (detected by TLC). The reaction mixture was filtered, evaporated and purified by a flash column chromatography (PE/EA = 5/1, with 1% Et_3N) to give 5-chloro-1-methyl-1*H*-indole-2-carbaldehyde (1.62 g, 84% yield) as a yellow solid.

Step B3-B5 followed the similar procedures as **Step A3-A5** above to provide the final product (*E*)-3-(5-chloro-1-methyl-1*H*-indol-2-yl)allyl methyl carbonate **2f** (1.89 g, 81% yield over 3 steps) as a white solid.

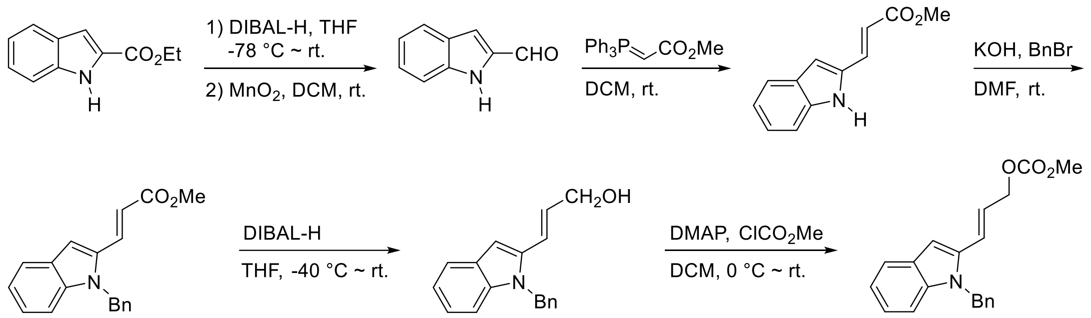

Supplementary Figure 3. Synthetic procedure **C** for substrate **2**. Pathway for the synthesis of *N*-substituted allyl carbonates started from unsubstituted ethyl indole-2-carboxylate (**2h**).

Step C1-C2 (10 mmol starting material) followed the similar procedures as **Step B2-B3** above to provide methyl (*E*)-3-(1*H*-indol-2-yl)acrylate (1.59 g, 79% yield over 2 steps) as a white solid.

Step C3 followed the similar procedure as **Step B1** above to provide *N*-protected product methyl (*E*)-3-(1-benzyl-1*H*-indol-2-yl)acrylate (2.19 g, 95% yield) as a white solid.

Step C4-C5 followed the similar procedures as **Step B4-B5** above to provide the final product

(*E*)-3-(1-benzyl-1*H*-indol-2-yl)allyl methyl carbonate **2h** (2.13 g, 88% yield over 2 steps) as a white solid.

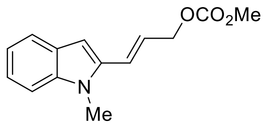

(*E*)-methyl (3-(1-methyl-1*H*-indol-2-yl)allyl) carbonate (2a): White solid; m.p. = 96 ºC. ^{1}H NMR (400 MHz, $CDCl_3$) δ 7.57 (d, *J* = 8.0 Hz, 1H), 7.28 (d, *J* = 8.0 Hz, 1H), 7.20 (t, *J* = 7.2 Hz, 1H), 7.09 (t, *J* = 7.2 Hz, 1H), 6.77 (d, *J* = 15.6 Hz, 1H), 6.69 (s, 1H), 6.35 (dt, *J* = 15.6, 6.4 Hz, 1H), 4.83 (d, *J* = 6.0 Hz, 2H), 3.82 (s, 3H), 3.74 (s, 3H); ^{13}C NMR (100 MHz, $CDCl_3$) δ 155.55, 137.91, 136.56, 127.56, 124.49, 123.51, 121.94, 120.53, 119.82, 109.18, 99.86, 68.25, 54.83, 29.80.

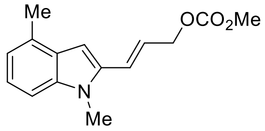

(*E*)-3-(1,4-dimethyl-1*H*-indol-2-yl)allyl methyl carbonate (2b): White solid; m.p. = 90 ºC. ^{1}H NMR (400 MHz, $CDCl_3$) δ 7.14-7.09 (m, 2H), 6.91-6.85 (m, 1H), 6.77 (d, *J* = 16.0 Hz, 1H), 6.71 (s, 1H), 6.37 (dt, *J* = 15.6, 6.4 Hz, 1H), 4.82 (dd, *J* = 6.4, 1.2 Hz, 2H), 3.81 (s, 3H), 3.72 (s, 3H), 2.52 (s, 3H); ^{13}C NMR (100 MHz, $CDCl_3$) δ 155.61, 137.72, 135.99, 130.10, 127.52, 124.14, 123.73, 122.18, 119.99, 106.85, 98.53, 68.36, 54.86, 30.00, 18.59.

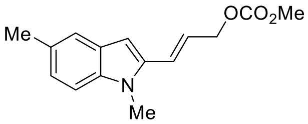

(*E*)-3-(1,5-dimethyl-1*H*-indol-2-yl)allyl methyl carbonate (2c): White solid; m.p. = 92 ºC. ^{1}H NMR (400 MHz, $CDCl_3$) δ 7.34 (s, 1H), 7.15 (d, *J* = 8.4 Hz, 1H), 7.01 (dd, *J* = 8.4, 1.6 Hz, 1H), 6.74 (d, *J* = 15.6 Hz, 1H), 6.59 (s, 1H), 6.32 (dt, *J* = 15.6, 6.4 Hz, 1H), 4.81 (dd, *J* = 6.4, 1.6 Hz, 2H), 3.81 (s, 3H), 3.69 (s, 3H), 2.42 (s, 3H); ^{13}C NMR (100 MHz, $CDCl_3$) δ 155.59, 136.55, 136.45, 129.04, 127.81, 124.06, 123.75, 123.69, 120.12, 108.89, 99.37, 68.36, 54.85, 29.87, 21.34.

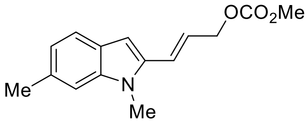

(*E*)-3-(1,6-dimethyl-1*H*-indol-2-yl)allyl methyl carbonate (2d): White solid; m.p. = 98 ºC. ^{1}H NMR (400 MHz, $CDCl_3$) δ 7.34 (s, 1H), 7.15 (d, *J* = 8.4 Hz, 1H), 7.01 (dd, *J* = 8.4, 1.6 Hz, 1H), 6.74 (d, *J* = 15.6 Hz, 1H), 6.59 (s, 1H), 6.32 (dt, *J* = 15.6, 6.4 Hz, 1H), 4.81 (dd, *J* = 6.4, 1.6 Hz, 2H), 3.81 (s, 3H), 3.69 (s, 3H), 2.42 (s, 3H); ^{13}C NMR (100 MHz, $CDCl_3$) δ 155.59, 138.40, 136.01, 131.89, 125.43, 123.84, 123.72, 121.69, 120.20, 109.17, 99.85, 68.40, 54.83, 29.77, 21.95.

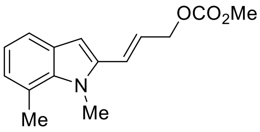

(*E*)-3-(1,7-dimethyl-1*H*-indol-2-yl)allyl methyl carbonate (2e): White solid; m.p. = 80 ºC. ^{1}H NMR (400 MHz, $CDCl_3$) δ 7.38 (d, *J* = 8.0 Hz, 1H), 6.93 (t, *J* = 7.2 Hz, 1H), 6.87 (d, *J* = 6.8 Hz, 1H), 6.73 (d, *J* = 15.6 Hz, 1H), 6.63 (s, 1H), 6.29 (dt, *J* = 15.6, 6.4 Hz, 1H), 4.81 (dd, *J* = 6.4, 1.2 Hz, 2H), 3.95 (s, 3H), 3.81 (s, 3H), 2.74 (s, 3H); ^{13}C NMR (100 MHz, $CDCl_3$) δ 155.57, 137.37, 136.98, 128.42, 125.12, 124.83, 123.85, 120.95, 119.91, 118.76, 100.66, 68.27, 54.85, 32.91, 20.39.

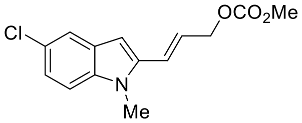

(*E*)-3-(5-chloro-1-methyl-1*H*-indol-2-yl)allyl methyl carbonate (2f): White solid; m.p. = 96 ºC. ^{1}H NMR (400 MHz, $CDCl_3$) δ 7.50 (d, *J* = 1.4 Hz, 1H), 7.14 (s, 1H), 7.12 (d, *J* = 1.9 Hz, 1H), 6.71 (d, *J* = 16.1 Hz, 1H), 6.58 (s, 1H), 6.34 (dt, *J* = 15.8, 6.3 Hz, 1H), 4.81 (dd, *J* = 6.3, 1.4 Hz, 2H), 3.81 (s, 3H), 3.67 (s, 3H); ^{13}C NMR (100 MHz, $CDCl_3$) δ 155.52, 137.87, 136.26, 128.47, 125.57, 125.42, 122.87, 122.12, 119.71, 110.18, 99.22, 68.02, 54.89, 29.97.

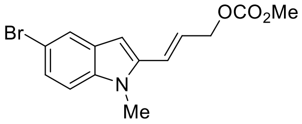

(*E*)-3-(5-bromo-1-methyl-1*H*-indol-2-yl)allyl methyl carbonate (2g): White solid; m.p. = 98 ºC. ^{1}H NMR (400 MHz, $CDCl_3$) δ 7.67 (d, *J* = 2.0 Hz, 1H), 7.28-7.23 (m, 1H), 7.12 (d, *J* = 8.8 Hz, 1H),

6.73 (d, *J* = 16.0 Hz, 1H), 6.59 (s, 1H), 6.36 (dt, *J* = 15.6, 6.4 Hz, 1H), 4.82 (dd, *J* = 6.4, 1.2 Hz, 2H), 3.82 (s, 3H), 3.70 (s, 3H); ^{13}C NMR (100 MHz, $CDCl_3$) δ 155.55, 137.76, 136.55, 129.19, 125.68, 124.70, 122.86, 113.07, 110.65, 99.17, 68.04, 54.92, 30.01.

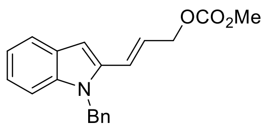

(*E*)-3-(1-benzyl-1*H*-indol-2-yl)allyl methyl carbonate (2h): White solid; m.p. = 88 ºC. ^{1}H NMR (400 MHz, $CDCl_3$) δ 7.60 (dd, *J* = 6.8, 1.2 Hz, 1H), 7.28-7.20 (m, 4H), 7.16-7.12 (m, 1H), 7.12-7.07 (m, 1H), 7.03-6.96 (m, 2H), 6.77 (s, 1H), 6.70-6.62 (m, 1H), 6.34 (dt, *J* = 15.6, 6.4 Hz, 1H), 5.36 (s, 2H), 4.73 (dd, *J* = 6.4, 1.2 Hz, 2H), 3.77 (s, 3H); ^{13}C NMR (100 MHz, $CDCl_3$) δ 155.49, 137.75, 137.49, 136.51, 128.76, 127.82, 127.37, 125.91, 124.96, 123.39, 122.28, 120.67, 120.17, 109.61, 100.50, 68.19, 54.82, 46.62.

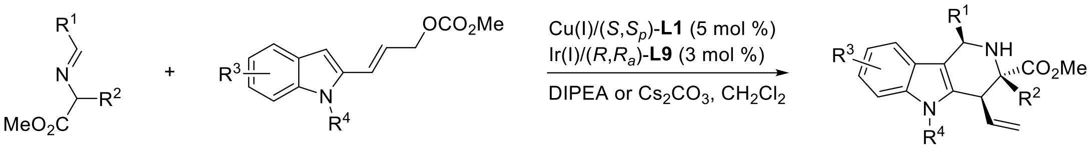

Supplementary Figure 4. Typical procedures for the synthesis of tetrahydro-γ-carbolines **3**. Various substitutions are tolerated in this reaction; four stereoisomers are predictably prepared at will by using four available sets of catalyst permutations, respectively.

A flame dried Schlenk tube **A** was cooled to room temperature and filled with N_2. To this flask were added $[Ir(COD)Cl]_2$ (0.003 mmol, 1.5 mol %), (R,R_a)-Me-THQphos-**L9** (0.006 mmol, 3.0 mol %), degassed THF (0.5 mL) and degassed *n*-propylamine (0.5 mL). The reaction mixture was heated at 50 ºC for 30 min and then the volatile solvents were removed under vacuum to gain a pale yellow solid. Meanwhile, $Cu(MeCN)_4BF_4$ (0.01 mmol, 5 mol %) and (S,S_p)-^{i}Pr-Phosferrox-**L1** (0.011 mmol, 5.5 mol %) were dissolved in 1.0 mL of CH_2Cl_2 in a Schlenk tube **B**, and stirred at room temperature for about 30 min. Indole derived allylic carbonates (0.20 mmol), aldimine esters (0.30 mmol), base (0.40 mmol DIPEA for glycine derived aldimine esters and 0.40 mmol Cs_2CO_3 for α-substituted aldimine esters) and CH_2Cl_2 (1.0 mL) were added into the Schlenk tube **A** and filled with N_2. The Cu/**L1** complex solution was then transferred from the Schlenk tube **B** to the Schlenk tube **A** *via* syringe. Finally, the reaction mixture was continuously stirred at room temperature under N_2 atmosphere.

While the starting material was consumed (monitored by TLC), the reaction mixture was quenched by adding 1 M HCl aqueous solution (2.0 mL) and stirring vigorously for 1 min. The organic layers were separated, and the aqueous layer was extracted with CH_2Cl_2 (5.0 mL × 2). The organic layer was combined, washed with saturated brine (10 mL) and dried over anhydrous Na_2SO_4. The organic solvent was removed by rotary evaporation to obtain a crude mixture, which was purified by flash column chromatography to give the pure product. The dr value was determined by ^{1}H NMR spectrum of the product, and the enantiomeric excess was recorded by HPLC analysis in comparison with the racemic sample.

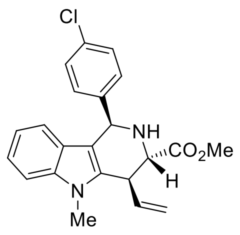

(1*R*,3*S*,4*R*)-**3a**

Methyl (1*R*,3*S*,4*R*)-1-(4-chlorophenyl)-5-methyl-4-vinyl-2,3,4,5-tetrahydro-1*H*-pyrido[4,3-*b*] indole-3-carboxylate (1*R*,3*S*,4*R*-**3a)**: Yield (98%); white solid; m.p. = 70 ºC. $[\alpha]^{20}_{D}$ = +52.4 (*c* 0.5, CH_2Cl_2); ^1H NMR (400 MHz, $CDCl_3$) δ 7.35 (d, *J* = 8.4 Hz, 2H), 7.29-7.23 (m, 3H), 7.15-7.11 (m, 1H), 6.91-6.85 (m, 1H), 6.82 (d, *J* = 8.0 Hz, 1H), 6.23 (ddd, *J* = 17.2, 10.0, 6.4 Hz, 1H), 5.40 (s, 1H), 5.31 (d, *J* = 10.0 Hz, 1H), 5.12 (d, *J* = 17.2 Hz, 1H), 4.21 (d, *J* = 6.4 Hz, 1H), 3.92 (d, *J* = 2.4 Hz, 1H), 3.71 (s, 3H), 3.66 (s, 3H). ^{13}C NMR (101 MHz, $CDCl_3$) δ 172.75, 142.21, 138.67, 137.22, 133.46, 133.15, 130.00, 128.55, 124.96, 121.23, 119.07, 118.98, 117.75, 110.02, 108.78, 60.26, 54.15, 52.34, 38.90, 29.24. HRMS (ESI+) Calcd. For $C_{22}H_{22}ClN_2O_2$ ($[M+H]^+$): 381.1364, found: 381.1360. The product was analyzed by HPLC to determine the enantiomeric excess: 99% ee (Chiralpak AD-H, *i*-propanol/hexane = 2/98, flow rate 1.0 mL/min, λ = 282 nm); t_r = 15.53 and 18.77 min.

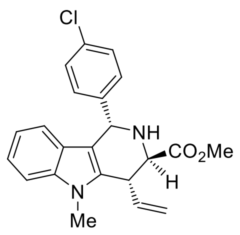

(1*S*,3*R*,4*S*)-**3a**

Methyl (1*S*,3*R*,4*S*)-1-(4-chlorophenyl)-5-methyl-4-vinyl-2,3,4,5-tetrahydro-1*H*-pyrido[4,3-*b*] indole-3-carboxylate (1*S*,3*R*,4*S*-**3a)**: Yield (95%); white solid. $[\alpha]^{20}_{D}$ = -50.3 (*c* 0.7, CH_2Cl_2). The product was analyzed by HPLC to determine the enantiomeric excess: 99% ee (Chiralpak AD-H, *i*-propanol/hexane = 2/98, flow rate 1.0 mL/min, λ = 282 nm); t_r = 15.53 and 18.77 min.

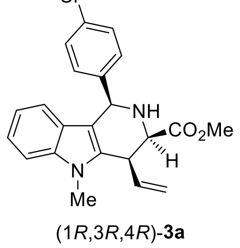

(1*R*,3*R*,4*R*)-**3a**

Methyl (1*R*,3*R*,4*R*)-1-(4-chlorophenyl)-5-methyl-4-vinyl-2,3,4,5-tetrahydro-1*H*-pyrido[4,3-*b*] indole-3-carboxylate (1*R*,3*R*,4*R*-**3a)**: Yield (92%); white solid; m.p. = 86 ºC. $[\alpha]^{20}_D$ = +146.3 (*c* 1.0, CH_2Cl_2); ^1H NMR (400 MHz, $CDCl_3$) δ 7.36-7.32 (m, 2H), 7.31-7.25 (m, 2H), 7.17-7.10 (m, 1H), 6.91-6.84 (m, 1H), 6.73 (d, *J* = 8.0 Hz, 1H), 6.02 (ddd, *J* = 17.2, 10.0, 7.2 Hz, 1H), 5.32 (d, *J* = 10.4 Hz, 1H), 5.22 (s, 1H), 5.17-5.08 (m, 1H), 4.14 (d, *J* = 3.6 Hz, 1H), 3.99 (dd, *J* = 6.0, 4.0 Hz, 1H), 3.79 (s, 3H), 3.64 (s, 3H). ^{13}C NMR (101 MHz, $CDCl_3$) δ 171.37, 141.19, 137.24, 135.12, 135.02, 133.42, 130.03, 128.64, 124.51, 121.33, 119.69, 119.24, 119.12, 111.06, 108.79, 60.83, 57.69, 52.12, 40.02, 28.98. HRMS (ESI+) Calcd. For $C_{22}H_{22}ClN_2O_2$ ($[M+H]^+$): 381.1364, found: 381.1369. The product was analyzed by HPLC to determine the enantiomeric excess: 99% ee (Chiralpak AD-H, *i*-propanol/hexane = 2/98, flow rate 1.0 mL/min, λ = 282 nm); t_r = 25.07 and 28.05 min.

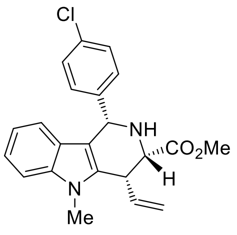

(1*S*,3*S*,4*S*)-**3a**

Methyl (1*S*,3*S*,4*S*)-1-(4-chlorophenyl)-5-methyl-4-vinyl-2,3,4,5-tetrahydro-1*H*-pyrido[4,3-*b*] indole-3-carboxylate (1*S*,3*S*,4*S*-**3a)**: Yield (96%); white solid. $[\alpha]^{20}_D$ = -156.0 (*c* 0.8, CH_2Cl_2). The product was analyzed by HPLC to determine the enantiomeric excess: 99% ee (Chiralpak AD-H, *i*-propanol/hexane = 2/98, flow rate 1.0 mL/min, λ = 282 nm); t_r = 25.07 and 28.05 min.

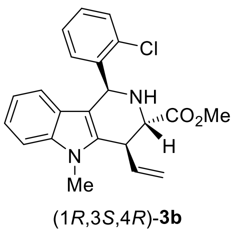

(1*R*,3*S*,4*R*)-**3b**

Methyl (1*S*,3*S*,4*R*)-1-(2-chlorophenyl)-5-methyl-4-vinyl-2,3,4,5-tetrahydro-1*H*-pyrido[4,3-*b*] indole-3-carboxylate (1*R*,3*S*,4*R*-**3b)**: Yield (82%); white solid; m.p. = 64 ºC. $[\alpha]^{20}_D$ = +52.3 (*c* 1.0, CH_2Cl_2); ^1H NMR (400 MHz, $CDCl_3$) δ 7.46-7.41 (m, 1H), 7.29 (d, *J* = 8.4 Hz, 1H), 7.23-7.12 (m, 3H), 7.12-7.05 (m, 1H), 6.89 (t, *J* = 7.2 Hz, 1H), 6.82 (d, *J* = 7.6 Hz, 1H), 6.24 (ddd, *J* = 17.2, 10.0,

6.8 Hz, 1H), 5.96 (s, 1H), 5.30 (d, *J* = 10.4 Hz, 1H), 5.13 (d, *J* = 16.8 Hz, 1H), 4.24 (d, *J* = 6.4 Hz, 1H), 3.95 (d, *J* = 1.6 Hz, 1H), 3.74 (s, 3H), 3.68 (s, 3H). ^{13}C NMR (101 MHz, $CDCl_3$) δ 172.77, 140.24, 138.63, 137.29, 134.10, 134.02, 130.37, 129.33, 128.72, 127.19, 124.89, 121.21, 119.30, 118.97, 117.72, 109.32, 108.77, 60.37, 52.35, 50.66, 38.75, 29.29. HRMS (ESI+) Calcd. For $C_{22}H_{22}ClN_2O_2$ ($[M+H]^+$): 381.1364, found: 381.1366. The product was analyzed by HPLC to determine the enantiomeric excess: 99% ee (Chiralpak AD-H, *i*-propanol/hexane = 2/98, flow rate 1.0 mL/min, λ = 234 nm); t_r = 9.40 and 10.13 min.

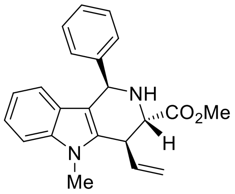

(1*R*,3*S*,4*R*)-**3c**

Methyl (1*R*,3*S*,4*R*)-5-methyl-1-phenyl-4-vinyl-2,3,4,5-tetrahydro-1*H*-pyrido[4,3-*b*] indole-3-carboxylate (1*R*,3*S*,4*R*-**3c)**: Yield (99%); white solid; m.p. = 54 ºC. $[\alpha]^{20}_D$ = -7.3 (*c* 1.4, CH_2Cl_2); ^{1}H NMR (400 MHz, $CDCl_3$) δ 7.41 (dd, *J* = 8.0, 1.6 Hz, 2H), 7.33-7.25 (m, 4H), 7.12 (ddd, *J* = 8.0, 6.4, 1.6 Hz, 1H), 6.89-6.80 (m, 2H), 6.25 (ddd, *J* = 17.2, 10.0, 6.8 Hz, 1H), 5.41 (s, 1H), 5.31 (d, *J* = 10.0 Hz, 1H), 5.14 (d, *J* = 17.2 Hz, 1H), 4.22 (d, *J* = 6.4 Hz, 1H), 3.94 (d, *J* = 2.0 Hz, 1H), 3.72 (s, 3H), 3.66 (s, 3H). ^{13}C NMR (101 MHz, $CDCl_3$) δ 172.86, 143.54, 138.77, 137.22, 133.40, 128.59, 128.40, 127.56, 125.16, 121.08, 119.26, 118.84, 117.72, 110.54, 108.67, 60.36, 54.83, 52.31, 38.91, 29.23. HRMS (ESI+) Calcd. For $C_{22}H_{23}N_2O_2$ ($[M+H]^+$): 347.1754, found: 347.1754. The product was analyzed by HPLC to determine the enantiomeric excess: 99% ee (Chiralpak AD-H, *i*-propanol/hexane = 2/98, flow rate 1.0 mL/min, λ = 232 nm); t_r = 13.33 and 15.82 min.

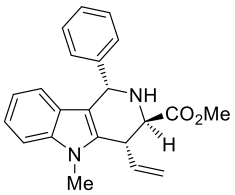

(1*S*,3*R*,4*S*)-**3c**

Methyl (1*S*,3*R*,4*S*)-5-methyl-1-phenyl-4-vinyl-2,3,4,5-tetrahydro-1*H*-pyrido[4,3-*b*] indole-3-carboxylate (1*S*,3*R*,4*S*-**3c)**: Yield (98%); white solid. $[\alpha]^{20}_D$ = +6.9 (*c* 0.9, CH_2Cl_2). The

product was analyzed by HPLC to determine the enantiomeric excess: 99% ee (Chiralpak AD-H, *i*-propanol/hexane = 2/98, flow rate 1.0 mL/min, λ = 232 nm); t_r = 13.33 and 15.82 min.

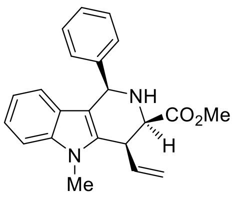

(1*R*,3*R*,4*R*)-**3c**

Methyl (1*R*,3*R*,4*R*)-5-methyl-1-phenyl-4-vinyl-2,3,4,5-tetrahydro-1*H*-pyrido[4,3-*b*] indole-3-carboxylate (1*R*,3*R*,4*R*-**3c)**: Yield (96%); white solid; m.p. = 62 ºC. $[\alpha]^{20}_D$ = +89.2 (*c* 0.2, CH_2Cl_2); ^1H NMR (400 MHz, $CDCl_3$) δ 7.43-7.38 (m, 2H), 7.34-7.26 (m, 4H), 7.15-7.09 (m, 1H), 6.87-6.82 (m, 1H), 6.73 (d, *J* = 8.0 Hz, 1H), 6.03 (ddd, *J* = 17.2, 10.4, 7.2 Hz, 1H), 5.33 (d, *J* = 10.4 Hz, 1H), 5.25 (s, 1H), 5.13 (d, *J* = 17.2 Hz, 1H), 4.17 (d, *J* = 4.0 Hz, 1H), 4.02-3.98 (m, 1H), 3.78 (s, 3H), 3.64 (s, 3H). ^{13}C NMR (101 MHz, $CDCl_3$) δ 171.48, 142.58, 137.22, 135.17, 134.89, 128.62, 128.47, 127.78, 124.70, 121.19, 119.69, 119.43, 118.98, 111.61, 108.68, 60.91, 58.37, 52.05, 39.99, 28.95. HRMS (ESI+) Calcd. For $C_{22}H_{23}N_2O_2$ ($[M+H]^+$): 347.1754, found: 347.1756. The product was analyzed by HPLC to determine the enantiomeric excess: 99% ee (Chiralpak AD-H, *i*-propanol/hexane = 2/98, flow rate 1.0 mL/min, λ = 232 nm); t_r = 29.80 and 34.58 min.

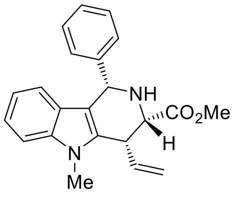

(1*S*,3*S*,4*S*)-**3c**

Methyl (1*S*,3*S*,4*S*)-5-methyl-1-phenyl-4-vinyl-2,3,4,5-tetrahydro-1*H*-pyrido[4,3-*b*] indole-3-carboxylate (1*S*,3*S*,4*S*-**3c)**: Yield (94%); white solid. $[\alpha]^{20}_D$ = -88.9 (*c* 0.8, CH_2Cl_2). The product was analyzed by HPLC to determine the enantiomeric excess: 99% ee (Chiralpak AD-H, *i*-propanol/hexane = 2/98, flow rate 1.0 mL/min, λ = 232 nm); t_r = 29.80 and 34.58 min.

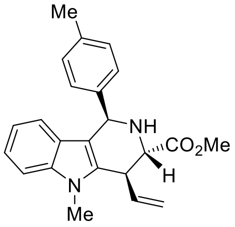

(1*R*,3*S*,4*R*)-**3d**

Methyl (1*R*,3*S*,4*R*)-5-methyl-1-(*p*-tolyl)-4-vinyl-2,3,4,5-tetrahydro-1*H*-pyrido[4,3-*b*] indole-3-carboxylate (1*R*,3*S*,4*R*-**3d)**: Yield (82%); white solid; m.p. = 54 ºC. $[\alpha]^{20}_D$ = +16.8 (*c* 1.5, CH_2Cl_2); ^1H NMR (400 MHz, $CDCl_3$) δ7.30-7.25 (m, 3H), 7.14-7.08 (m, 3H), 6.89-6.83 (m, 2H), 6.24 (ddd, *J* = 16.8, 10.0, 6.8 Hz, 1H), 5.38 (s, 1H), 5.30 (d, *J* = 10.0 Hz, 1H), 5.13 (d, *J* = 17.2 Hz, 1H), 4.21 (d, *J* = 6.0 Hz, 1H), 3.93 (d, *J* = 2.0 Hz, 1H), 3.71 (s, 3H), 3.66 (s, 3H), 2.32 (s, 3H). ^{13}C NMR (101 MHz, $CDCl_3$) δ 172.89, 140.48, 138.79, 137.21, 137.07, 133.43, 129.07, 128.43, 125.22, 121.04, 119.34, 118.79, 117.69, 110.67, 108.64, 60.36, 54.45, 52.29, 38.97, 29.23, 21.17. HRMS (ESI+) Calcd. For $C_{23}H_{25}N_2O_2$ ($[M+H]^+$): 361.1911, found: 361.1910. The product was analyzed by HPLC to determine the enantiomeric excess: 99% ee (Chiralpak AD-H plus AD-H, *i*-propanol/hexane = 2/98, flow rate 1.0 mL/min, λ = 238 nm); t_r = 28.13 and 33.35 min.

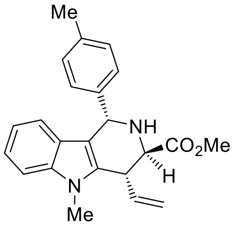

(1*S*,3*R*,4*S*)-**3d**

Methyl (1*S*,3*R*,4*S*)-5-methyl-1-(*p*-tolyl)-4-vinyl-2,3,4,5-tetrahydro-1*H*-pyrido[4,3-*b*] indole-3-carboxylate (1*S*,3*R*,4*S*-**3d)**: Yield (78%); white solid. $[\alpha]^{20}_D$ = -17.4 (*c* 1.2, CH_2Cl_2). The product was analyzed by HPLC to determine the enantiomeric excess: 99% ee (Chiralpak AD-H plus AD-H, *i*-propanol/hexane = 2/98, flow rate 1.0 mL/min, λ = 238 nm); t_r = 28.13 and 33.35 min.

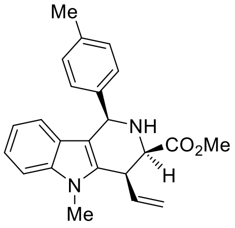

(1*R*,3*R*,4*R*)-**3d**

Methyl (1*R*,3*R*,4*R*)-5-methyl-1-(*p*-tolyl)-4-vinyl-2,3,4,5-tetrahydro-1*H*-pyrido[4,3-*b*] indole-3-carboxylate (1*R*,3*R*,4*R*-**3d)**: Yield (76%); white solid; m.p. = 50 ºC. $[\alpha]^{20}_{D}$ = +73.9 (*c* 1.1, CH_2Cl_2); ^1H NMR (400 MHz, $CDCl_3$) δ7.30-7.26 (m, 3H), 7.14-7.09 (m, 3H), 6.86 (t, *J* = 7.6 Hz, 1H), 6.76 (d, *J* = 8.0 Hz, 1H), 6.01 (ddd, *J* = 16.8, 10.0, 7.2 Hz, 1H), 5.32 (d, *J* = 10.0 Hz, 1H), 5.22 (s, 1H), 5.12 (d, *J* = 16.8 Hz, 1H), 4.16 (d, *J* = 3.6 Hz, 1H), 4.02-3.96 (m, 1H), 3.78 (s, 3H), 3.63 (s, 3H), 2.34 (s, 3H). ^{13}C NMR (101 MHz, $CDCl_3$) δ 171.52, 139.55, 137.33, 137.18, 135.14, 134.84, 129.14, 128.44, 124.74, 121.15, 119.68, 119.51, 118.92, 111.71, 108.64, 60.88, 57.93, 52.03, 39.94, 28.94, 21.18. HRMS (ESI+) Calcd. For $C_{23}H_{25}N_2O_2$ ($[M+H]^+$): 361.1911, found: 361.1912. The product was analyzed by HPLC to determine the enantiomeric excess: 99% ee (Chiralpak AD-H plus AD-H, *i*-propanol/hexane = 2/98, flow rate 1.0 mL/min, λ = 238 nm); t_r = 48.58 and 46.05 min.

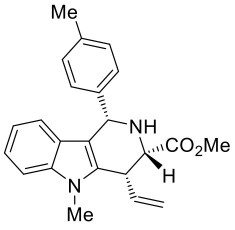

(1*S*,3*S*,4*S*)-**3d**

Methyl (1*S*,3*S*,4*S*)-5-methyl-1-(*p*-tolyl)-4-vinyl-2,3,4,5-tetrahydro-1*H*-pyrido[4,3-*b*] indole-3-carboxylate (1*S*,3*S*,4*S*-**3d)**: Yield (80%); white solid. $[\alpha]^{20}_{D}$ = -72.8 (*c* 0.9, CH_2Cl_2). The product was analyzed by HPLC to determine the enantiomeric excess: 99% ee (Chiralpak AD-H plus AD-H, *i*-propanol/hexane = 2/98, flow rate 1.0 mL/min, λ = 238 nm); t_r = 48.58 and 46.05 min.

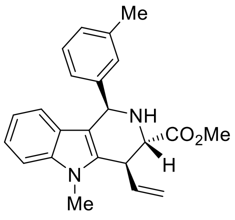

(1*R*,3*S*,4*R*)-**3e**

Methyl (1*R*,3*S*,4*R*)-5-methyl-1-(*m*-tolyl)-4-vinyl-2,3,4,5-tetrahydro-1*H*-pyrido[4,3-*b*] indole-3-carboxylate (1*R*,3*S*,4*R*-**3e)**: Yield (85%); white solid; m.p. = 58 ºC. $[\alpha]^{20}_D$ = +33.6 (*c* 0.9, CH_2Cl_2); ^1H NMR (400 MHz, $CDCl_3$) δ 7.27 (d, *J* = 8.4 Hz, 1H), 7.24-7.17 (m, 3H), 7.14-7.09 (m, 1H), 7.09-7.06 (m, 1H), 6.88-6.83 (m, 2H), 6.25 (ddd, *J* = 17.2, 10.0, 6.8 Hz, 1H), 5.37 (s, 1H), 5.32 (d, *J* = 10.0 Hz, 1H), 5.14 (d, *J* = 17.2 Hz, 1H), 4.22 (d, *J* = 6.4 Hz, 1H), 3.94 (d, *J* = 2.0 Hz, 1H), 3.72 (s, 3H), 3.66 (s, 3H), 2.30 (s, 3H). ^{13}C NMR (101 MHz, $CDCl_3$) δ 172.84, 143.39, 138.80, 137.90, 137.20, 133.36, 129.28, 128.34, 128.23, 125.58, 125.22, 121.03, 119.35, 118.80, 117.71, 110.56, 108.62, 60.38, 54.83, 52.30, 38.90, 29.21, 21.45. HRMS (ESI+) Calcd. For $C_{23}H_{25}N_2O_2$ ($[M+H]^+$): 361.1911, found: 361.1912. The product was analyzed by HPLC to determine the enantiomeric excess: 99% ee (Chiralpak AD-H plus AD-H, *i*-propanol/hexane = 2/98, flow rate 1.0 mL/min, λ = 234 nm); t_r = 21.94 and 23.41 min.

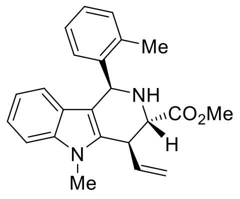

(1*R*,3*S*,4*R*)-**3f**

Methyl (1*R*,3*S*,4*R*)-5-methyl-1-(*o*-tolyl)-4-vinyl-2,3,4,5-tetrahydro-1*H*-pyrido[4,3-*b*] indole-3-carboxylate (1*R*,3*S*,4*R*-**3f)**: Yield (78%); white solid; m.p. = 52 ºC. $[\alpha]^{20}_D$ = +74.7 (*c* 0.7, CH_2Cl_2); ^1H NMR (400 MHz, $CDCl_3$) δ 7.28 (d, *J* = 8.0 Hz, 1H), 7.22 (d, *J* = 8.4 Hz, 1H), 7.18 (dd, *J* = 7.2, 1.2 Hz, 1H), 7.16-7.10 (m, 2H), 7.04 (t, *J* = 7.2 Hz, 1H), 6.88-6.82 (m, 1H), 6.71 (d, *J* = 7.6 Hz, 1H), 6.23 (ddd, *J* = 17.2, 10.0, 6.8 Hz, 1H), 5.65 (brs, 1H), 5.32-5.27 (m, 1H), 5.13 (d, *J* = 17.2 Hz, 1H), 4.22 (d, *J* = 6.4 Hz, 1H), 3.94 (d, *J* = 2.0 Hz, 1H), 3.73 (s, 3H), 3.67 (s, 3H), 2.60 (brs, 3H). ^{13}C NMR (101 MHz, $CDCl_3$) δ 172.76, 140.53, 138.60, 137.19, 133.73, 130.32, 128.64, 127.33, 126.18, 125.02, 121.07, 119.35, 118.80, 117.80, 110.65, 108.65, 60.50, 52.28, 38.76, 29.25, 19.09. HRMS (ESI+) Calcd. For $C_{23}H_{25}N_2O_2$ ($[M+H]^+$): 361.1911, found: 361.1914. The product was analyzed by HPLC to determine the enantiomeric excess: 99% ee (Chiralpak AD-H plus AD-H, *i*-propanol/hexane = 2/98, flow rate 1.0 mL/min, λ = 234 nm); t_r = 16.94 and 18.81 min.

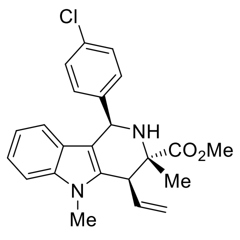

(1*R*,3*S*,4*R*)-**3g**

Methyl (1*R*,3*S*,4*R*)-1-(4-chlorophenyl)-3,5-dimethyl-4-vinyl-2,3,4,5-tetrahydro-1*H*-pyrido [4,3-*b*]indole-3-carboxylate (1*R*,3*S*,4*R*-**3g)**: Yield (80%); white solid; m.p. = 84 ºC. $[\alpha]^{20}_{D}$ = +125.7 (*c* 1.0, CH_2Cl_2); ^{1}H NMR (400 MHz, $CDCl_3$) δ 7.37-7.32 (m, 2H), 7.30-7.25 (m, 2H), 7.24 (t, *J* = 8.0 Hz, 1H), 7.10 (ddd, *J* = 8.0, 6.8, 1.2 Hz, 1H), 6.88-6.81 (m, 1H), 6.78 (d, *J* = 7.6 Hz, 1H), 6.07 (ddd, *J* = 17.2, 10.0, 8.8 Hz, 1H), 5.35 (s, 1H), 5.29 (m, 2H), 4.11 (d, *J* = 8.8 Hz, 1H), 3.66 (s, 3H), 3.65 (s, 3H), 1.38 (s, 3H). ^{13}C NMR (101 MHz, $CDCl_3$) δ 176.12, 142.72, 137.47, 136.83, 135.71, 133.10, 130.02, 128.56, 124.85, 121.06, 119.03, 118.83, 118.34, 109.09, 108.77, 62.45, 55.24, 52.41, 43.40, 28.98, 25.27. HRMS (ESI+) Calcd. For $C_{23}H_{24}ClN_2O_2$ ($[M+H]^+$): 395.1521, found: 395.1524. The product was analyzed by HPLC to determine the enantiomeric excess: 99% ee (Chiralpak AS-H, *i*-propanol/hexane = 2/98, flow rate 0.5 mL/min, λ = 282 nm); t_r = 11.25 and 13.41 min.

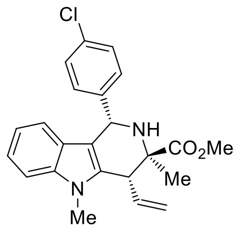

(1*S*,3*R*,4*S*)-**3g**

Methyl (1*S*,3*R*,4*S*)-1-(4-chlorophenyl)-3,5-dimethyl-4-vinyl-2,3,4,5-tetrahydro-1*H*-pyrido [4,3-*b*]indole-3-carboxylate (1*S*,3*R*,4*S*-**3g)**: Yield (77%); white solid. $[\alpha]^{20}_{D}$ = -118.0 (*c* 0.7, CH_2Cl_2). The product was analyzed by HPLC to determine the enantiomeric excess: 99% ee (Chiralpak AS-H, *i*-propanol/hexane = 2/98, flow rate 0.5 mL/min, λ = 282 nm); t_r = 11.25 and 13.41 min.

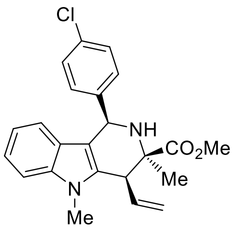

(1*R*,3*R*,4*R*)-**3g**

Methyl (1*R*,3*R*,4*R*)-1-(4-chlorophenyl)-3,5-dimethyl-4-vinyl-2,3,4,5-tetrahydro-1*H*-pyrido [4,3-*b*]indole-3-carboxylate (1*R*,3*R*,4*R*-**3g)**: Yield (76%); white solid; m.p. = 46 ºC. $[\alpha]^{20}_{D}$ = +252.2 (*c* 0.9, CH_2Cl_2); ^{1}H NMR (400 MHz, $CDCl_3$) δ 7.38-7.33 (m, 2H), 7.32-7.25 (m, 3H), 7.14 (ddd, *J* = 8.4, 7.2, 1.2 Hz, 1H), 6.93-6.85 (m, 1H), 6.78 (d, *J* = 8.0 Hz, 1H), 5.94 (ddd, *J* = 17.2, 10.0, 7.2 Hz, 1H), 5.28-5.22 (m, 1H), 5.17 (s, 1H), 5.11-5.03 (m, 1H), 3.75 (s, 3H), 3.72 (d, *J* = 6.8 Hz, 1H), 3.63 (s, 3H), 1.59 (s, 3H). ^{13}C NMR (101 MHz, $CDCl_3$) δ 174.36, 141.15, 137.39, 136.47, 134.56, 133.35, 130.22, 128.68, 124.55, 121.16, 119.30, 119.06, 119.04, 108.81, 108.77, 63.13, 53.81, 52.18, 45.19, 28.95, 22.06. HRMS (ESI+) Calcd. For $C_{23}H_{24}ClN_2O_2$ ($[M+H]^+$): 395.1521, found: 395.1519. The product was analyzed by HPLC to determine the enantiomeric excess: 99% ee (Chiralpak AS-H, *i*-propanol/hexane = 2/98, flow rate 1.0 mL/min, λ = 282 nm); t_r = 6.85 and 8.55 min.

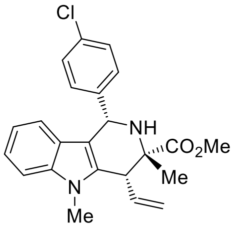

(1*S*,3*S*,4*S*)-**3g**

Methyl (1*S*,3*S*,4*S*)-1-(4-chlorophenyl)-3,5-dimethyl-4-vinyl-2,3,4,5-tetrahydro-1*H*-pyrido [4,3-*b*]indole-3-carboxylate (1*S*,3*S*,4*S*-**3g)**: Yield (75%); white solid. $[\alpha]^{20}_{D}$ = -268.3 (*c* 1.0, CH_2Cl_2). The product was analyzed by HPLC to determine the enantiomeric excess: 99% ee (Chiralpak AS-H, *i*-propanol/hexane = 2/98, flow rate 1.0 mL/min, λ = 282 nm); t_r = 6.85 and 8.55 min.

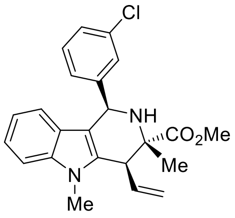

(1*R*,3*S*,4*R*)-**3h**

Methyl (1*R*,3*S*,4*R*)-1-(3-chlorophenyl)-3,5-dimethyl-4-vinyl-2,3,4,5-tetrahydro-1*H*-pyrido [4,3-*b*]indole-3-carboxylate (1*R*,3*S*,4*R*-**3h)**: Yield (86%); white solid; m.p. = 56 ºC. $[\alpha]^{20}_{D}$ = +124.5 (*c* 1.0, CH_2Cl_2); ^{1}H NMR (400 MHz, $CDCl_3$) δ 7.42 (s, 1H), 7.32-7.29 (m, 1H), 7.25-7.21 (m, 3H), 7.10 (ddd, *J* = 8.0, 6.8, 1.6 Hz, 1H), 6.88-6.83 (m, 1H), 6.83-6.79 (m, 1H), 6.08 (ddd, *J* = 17.2, 10.0, 8.8 Hz, 1H), 5.35 (s, 1H), 5.30 (m, 2H), 4.11 (d, *J* = 8.8 Hz, 1H) 3.66 (s, 3H), 3.64 (s, 3H), 1.38 (s, 3H). ^{13}C NMR (101 MHz, $CDCl_3$) δ 176.06, 146.32, 137.41, 136.77, 135.64, 134.04, 129.63, 128.76, 127.69, 126.86, 124.80, 121.02, 118.95, 118.83, 118.36, 108.84, 108.75, 62.39, 55.55, 52.38, 43.37, 28.94, 25.23. HRMS (ESI+) Calcd. For $C_{23}H_{24}ClN_2O_2$ ($[M+H]^+$): 395.1521, found: 395.1518. The product was analyzed by HPLC to determine the enantiomeric excess: 99% ee (Chiralpak AS-H, *i*-propanol/hexane = 2/98, flow rate 0.5 mL/min, λ = 234 nm); t_r = 10.67 and 12.02 min.

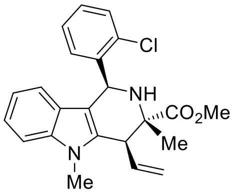

(1*R*,3*S*,4*R*)-**3i**

Methyl (1*S*,3*S*,4*R*)-1-(2-chlorophenyl)-3,5-dimethyl-4-vinyl-2,3,4,5-tetrahydro-1*H*-pyrido [4,3-*b*]indole-3-carboxylate (1*R*,3*S*,4*R*-**3i)**: Yield (82%); white solid; m.p. = 58 ºC. $[\alpha]^{20}_{D}$ = +170.0 (*c* 1.0, CH_2Cl_2); ^{1}H NMR (400 MHz, $CDCl_3$) δ 7.45-7.39 (m, 1H), 7.26 (d, *J* = 8.0 Hz, 1H), 7.21-7.15 (m, 2H), 7.14-7.07 (m, 2H), 6.90-6.83 (m, 1H), 6.78 (d, *J* = 8.0 Hz, 1H), 6.11-5.98 (m, 1H), 5.93 (s, 1H), 5.29 (s, 1H), 5.28-5.23 (m, 1H), 4.12 (d, *J* = 8.4 Hz, 1H), 3.68 (s, 3H), 3.67 (s, 3H), 1.39 (s, 3H). ^{13}C NMR (101 MHz, $CDCl_3$) δ 176.16, 140.64, 137.44, 136.78, 136.39, 133.78, 130.55, 129.16, 128.57, 127.26, 124.79, 121.01, 119.26, 118.80, 118.20, 108.75, 108.27, 62.44, 52.34, 51.39, 43.34, 28.99, 25.12. HRMS (ESI+) Calcd. For $C_{23}H_{24}ClN_2O_2$ ($[M+H]^+$): 395.1521, found: 395.1522. The product was analyzed by HPLC to determine the enantiomeric excess: 99% ee (Chiralpak AS-H,

i-propanol/hexane = 2/98, flow rate 0.5 mL/min, λ = 234 nm); t_r = 9.51 and 10.65 min.

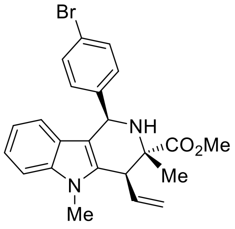

(1*R*,3*S*,4*R*)-**3j**

Methyl (1*R*,3*S*,4*R*)-1-(4-bromophenyl)-3,5-dimethyl-4-vinyl-2,3,4,5-tetrahydro-1*H*-pyrido[4,3-*b*]indole-3-carboxylate (1*R*,3*S*,4*R*-**3j)**: Yield (79%); white solid; m.p. = 60 ºC. $[\alpha]^{20}_{D}$ = +112.9 (*c* 0.8, CH_2Cl_2); ^{1}H NMR (400 MHz, $CDCl_3$) δ 7.45-7.40 (m, 2H), 7.31-7.27 (m, 2H), 7.24 (d, *J* = 8.4 Hz, 1H), 7.10 (ddd, *J* = 8.0, 7.2, 1.2 Hz, 1H), 6.87-6.82 (m, 1H), 6.78 (d, *J* = 8.0 Hz, 1H), 6.06 (ddd, *J* = 17.2, 10.0, 8.8 Hz, 1H), 5.34 (s, 1H), 5.32-5.24 (m, 2H), 4.11 (d, *J* = 8.8 Hz, 1H), 3.66 (s, 3H), 3.65 (s, 3H), 1.38 (s, 3H). ^{13}C NMR (101 MHz, $CDCl_3$) δ 176.09, 143.21, 137.42, 136.78, 135.69, 131.49, 130.38, 124.80, 121.27, 121.05, 119.00, 118.82, 118.35, 108.96, 108.76, 62.41, 55.26, 52.41, 43.36, 28.96, 25.25. HRMS (ESI+) Calcd. For $C_{23}H_{24}BrN_2O_2$ ($[M+H]^+$): 439.1016, found: 439.1022. The product was analyzed by HPLC to determine the enantiomeric excess: 99% ee (Chiralpak AS-H, *i*-propanol/hexane = 2/98, flow rate 1.0 mL/min, λ = 234 nm); t_r = 5.44 and 6.73 min.

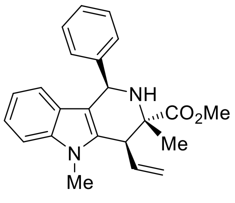

(1*R*,3*S*,4*R*)-**3k**

Methyl (1*R*,3*S*,4*R*)-3,5-dimethyl-1-phenyl-4-vinyl-2,3,4,5-tetrahydro-1*H*-pyrido[4,3-*b*]indole-3-carboxylate (1*R*,3*S*,4*R*-**3k)**: Yield (93%); white solid; m.p. = 54 ºC. $[\alpha]^{20}_{D}$ = +118.2 (*c* 0.9, CH_2Cl_2); ^{1}H NMR (400 MHz, $CDCl_3$) δ 7.41 (dd, *J* = 8.0, 1.6 Hz, 2H), 7.33-7.21 (m, 4H), 7.08 (ddd, *J* = 8.0, 6.4, 1.6 Hz, 1H), 6.85-6.80 (m, 1H), 6.79-6.77 (m, 1H), 6.10 (ddd, *J* = 17.2, 10.0, 8.8 Hz, 1H), 5.36 (s, 1H), 5.31-5.25 (m, 2H), 4.12 (d, *J* = 8.8 Hz, 1H), 3.66 (s, 3H), 3.66 (s, 3H), 1.38 (s, 3H). ^{13}C NMR (101 MHz, $CDCl_3$) δ 176.23, 144.07, 137.44, 136.99, 135.63, 128.64, 128.39, 127.51,

125.04, 120.89, 119.19, 118.67, 118.19, 109.59, 108.65, 62.46, 55.91, 52.35, 43.42, 28.95, 25.31. HRMS (ESI+) Calcd. For $C_{23}H_{25}N_2O_2$ ([M+H]$^+$): 361.1911, found: 361.1914. The product was analyzed by HPLC to determine the enantiomeric excess: 99% ee (Chiralpak AS-H, *i*-propanol/hexane = 2/98, flow rate 0.5 mL/min, λ = 234 nm); t_r = 12.05 and 13.98 min.

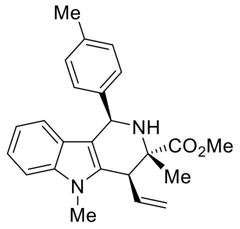

(1*R*,3*S*,4*R*)-**3l**

Methyl (1*R*,3*S*,4*R*)-3,5-dimethyl-1-(*p*-tolyl)-4-vinyl-2,3,4,5-tetrahydro-1*H*-pyrido[4,3-*b*] indole-3-carboxylate (1*R*,3*S*,4*R*-**3l)**: Yield (84%); white solid; m.p. = 50 ºC. $[\alpha]^{20}_D$ = +116.5 (*c* 1.1, CH_2Cl_2); ^1H NMR (400 MHz, $CDCl_3$) δ 7.29 (d, *J* = 8.0 Hz, 2H), 7.22 (d, *J* = 8.4 Hz, 1H), 7.12-7.05 (m, 3H), 6.86-6.79 (m, 2H), 6.08 (ddd, *J* = 17.2, 10.0, 8.8 Hz, 1H), 5.33 (s, 1H), 5.30-5.23 (m, 2H), 4.11 (d, *J* = 8.8 Hz, 1H), 3.65 (s, 3H), 3.64 (s, 3H), 2.31 (s, 3H), 1.37 (s, 3H). ^{13}C NMR (101 MHz, $CDCl_3$) δ 176.22, 141.02, 137.39, 136.99, 135.62, 129.05, 128.41, 125.05, 120.83, 119.23, 118.59, 118.10, 109.62, 108.60, 62.42, 55.45, 52.30, 43.40, 28.90, 25.27, 21.14. HRMS (ESI+) Calcd. For $C_{24}H_{27}N_2O_2$ ([M+H]$^+$): 375.2067, found: 375.2070. The product was analyzed by HPLC to determine the enantiomeric excess: 99% ee (Chiralpak AS-H, *i*-propanol/hexane = 2/98, flow rate 1.0 mL/min, λ = 254 nm); t_r = 4.51 and 5.38 min.

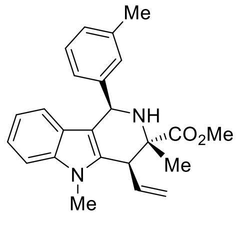

(1*R*,3*S*,4*R*)-**3m**

Methyl (1*R*,3*S*,4*R*)-3,5-dimethyl-1-(*m*-tolyl)-4-vinyl-2,3,4,5-tetrahydro-1*H*-pyrido[4,3-*b*] indole-3-carboxylate (1*R*,3*S*,4*R*-**3m)**: Yield (80%); white solid; m.p. = 62 ºC. $[\alpha]^{20}_D$ = +121.9 (*c* 1.1, CH_2Cl_2); ^1H NMR (400 MHz, $CDCl_3$) δ 7.26-7.22 (m, 2H), 7.21-7.17 (m, 2H), 7.09 (ddd, *J* = 8.4, 6.0, 2.4 Hz, 2H), 6.86-6.78 (m, 2H), 6.10 (ddd, *J* = 17.2, 10.0, 8.8 Hz, 1H), 5.32-5.24 (m, 3H), 4.12

(d, J = 8.8 Hz, 1H), 3.67 (s, 3H), 3.66 (s, 3H), 2.31 (s, 3H), 1.38 (s, 3H). ^{13}C NMR (101 MHz, $CDCl_3$) δ 176.23, 143.90, 137.88, 137.41, 137.02, 135.63, 129.29, 128.31, 128.26, 125.65, 125.10, 120.84, 119.29, 118.64, 118.18, 109.59, 108.62, 62.48, 55.89, 52.35, 43.41, 28.95, 25.33, 21.47. HRMS (ESI+) Calcd. For $C_{24}H_{27}N_2O_2$ ([M+H]$^+$): 375.2067, found: 375.2071. The product was analyzed by HPLC to determine the enantiomeric excess: 99% ee (Chiralpak AS-H, *i*-propanol/hexane = 2/98, flow rate 0.5 mL/min, λ = 234 nm); t_r = 9.55 and 11.24 min.

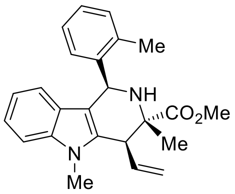

(1*R*,3*S*,4*R*)-**3n**

Methyl (1*R*,3*S*,4*R*)-3,5-dimethyl-1-(*o*-tolyl)-4-vinyl-2,3,4,5-tetrahydro-1*H*-pyrido[4,3-*b*] indole-3-carboxylate (1*R*,3*S*,4*R*-**3n)**: Yield (84%); white solid; m.p. = 54 ºC. $[\alpha]^{20}_{D}$ = +232.7 (*c* 1.0, CH_2Cl_2); ^{1}H NMR (400 MHz, $CDCl_3$) δ 7.25 (d, J = 8.4 Hz, 2H), 7.16 (t, J = 6.8 Hz, 2H), 7.12-7.04 (m, 2H), 6.82 (t, J = 7.6 Hz, 1H), 6.67 (d, J = 7.6 Hz, 1H), 6.07 (ddd, J = 17.2, 10.0, 8.8 Hz, 1H), 5.64 (brs, 1H), 5.30-5.22 (m, 2H), 4.12 (d, J = 8.8 Hz, 1H), 3.67 (s, 3H), 3.67 (s, 3H), 2.63 (brs, 3H), 1.37 (s, 3H). ^{13}C NMR (101 MHz, $CDCl_3$) δ 176.23, 141.15, 137.39, 136.88, 135.98, 135.77, 130.14, 128.84, 127.22, 126.38, 124.89, 120.86, 119.23, 118.64, 118.15, 109.65, 108.64, 62.46, 52.30, 50.66, 43.29, 28.99, 25.35, 19.06. HRMS (ESI+) Calcd. For $C_{24}H_{27}N_2O_2$ ([M+H]$^+$): 375.2067, found: 375.2073. The product was analyzed by HPLC to determine the enantiomeric excess: 99% ee (Chiralpak IC plus IA, *i*-propanol/hexane = 1/99, flow rate 0.5 mL/min, λ = 234 nm); t_r = 9.55 and 11.24 min.

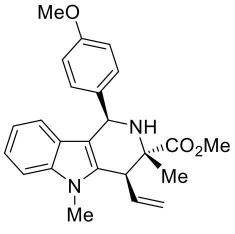

(1*R*,3*S*,4*R*)-**3o**

Methyl (1*R*,3*S*,4*R*)-3,5-dimethyl-1-(4-methoxyphenyl)-4-vinyl-2,3,4,5-tetrahydro-1*H*-pyrido

[4,3-*b*]indole-3-carboxylate (1*R*,3*S*,4*R*-3o): Yield (88%); white solid; m.p. = 58 ºC. $[\alpha]^{20}_D$ = +91.8 (*c* 0.9, CH_2Cl_2); ^1H NMR (400 MHz, $CDCl_3$) δ 7.35-7.32 (m, 1H), 7.32-7.30 (m, 1H), 7.23 (d, *J* = 8.8 Hz, 1H), 7.09 (ddd, *J* = 8.4, 6.4, 2.0 Hz, 1H), 6.86-6.78 (m, 4H), 6.08 (ddd, *J* = 17.2, 10.0, 8.4 Hz, 1H), 5.32 (d, *J* = 0.8 Hz, 1H), 5.31-5.23 (m, 2H), 4.11 (d, *J* = 8.4 Hz, 1H), 3.77 (s, 3H), 3.65 (s, 3H), 3.65 (s, 3H), 1.37 (s, 3H). ^{13}C NMR (101 MHz, $CDCl_3$) δ 176.23, 158.88, 137.40, 137.00, 136.26, 135.58, 129.60, 125.06, 120.85, 119.25, 118.61, 118.14, 113.71, 109.80, 108.62, 62.44, 55.16, 55.12, 52.32, 43.36, 28.93, 25.30. HRMS (ESI+) Calcd. For $C_{24}H_{27}N_2O_3$ ($[M+H]^+$): 391.2016, found: 391.2021. The product was analyzed by HPLC to determine the enantiomeric excess: 99% ee (Chiralpak AS-H, *i*-propanol/hexane = 2/98, flow rate 0.5 mL/min, λ = 234 nm); t_r = 22.14 and 26.23 min.

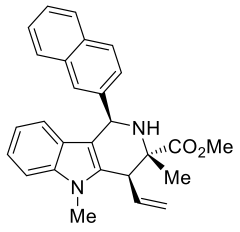

(1*R*,3*S*,4*R*)-**3p**

Methyl (1*R*,3*S*,4*R*)-3,5-dimethyl-1-(naphthalen-2-yl)-4-vinyl-2,3,4,5-tetrahydro-1*H*-pyrido [4,3-*b*]indole-3-carboxylate (1*R*,3*S*,4*R*-3p): Yield (75%); white solid; m.p. =136 ºC. $[\alpha]^{20}_D$ = -10.2 (*c* 1.0, CH_2Cl_2); ^1H NMR (400 MHz, $CDCl_3$) δ 7.97 (s, 1H), 7.88-7.83 (m, 1H), 7.81-7.77 (m, 1H), 7.72 (d, *J* = 8.4 Hz, 1H), 7.50-7.41 (m, 3H), 7.23 (d, *J* = 8.4 Hz, 1H), 7.06 (ddd, *J* = 8.0, 6.4, 1.6 Hz, 1H), 6.79-6.70 (m, 2H), 6.16 (ddd, *J* = 16.4, 10.8, 8.8 Hz, 1H), 5.54 (s, 1H), 5.34 (s, 1H), 5.33-5.28 (m, 1H), 4.16 (d, *J* = 8.4 Hz, 1H), 3.68 (s, 6H), 1.40 (s, 3H). ^{13}C NMR (101 MHz, $CDCl_3$) δ 176.27, 141.58, 137.44, 137.01, 135.71, 133.30, 133.15, 128.36, 127.86, 127.67, 127.28, 126.67, 125.77, 125.61, 125.05, 120.93, 119.15, 118.73, 118.22, 109.31, 108.65, 62.46, 56.08, 52.41, 43.51, 29.00, 25.34. HRMS (ESI+) Calcd. For $C_{27}H_{27}N_2O_2$ ($[M+H]^+$): 411.2067, found: 411.2071. The product was analyzed by HPLC to determine the enantiomeric excess: 99% ee (Chiralpak AS-H, *i*-propanol/hexane = 2/98, flow rate 1.0 mL/min, λ = 254 nm); t_r = 5.54 and 6.78 min.

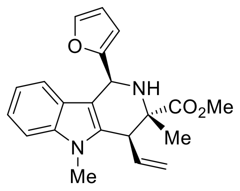

(1*R*,3*S*,4*R*)-**3q**

Methyl (1*R*,3*S*,4*R*)-3,5-dimethyl-1-(furan-2-yl)-4-vinyl-2,3,4,5-tetrahydro-1*H*-pyrido[4,3-*b*] indole-3-carboxylate (1*R*,3*S*,4*R*-**3q)**: Yield (83%); white solid; m.p. = 52 ºC. $[\alpha]^{20}_D$ = +258.9 (*c* 1.0, CH_2Cl_2); ^1H NMR (400 MHz, $CDCl_3$) δ 7.34 (dd, *J* = 1.6, 0.8 Hz, 1H), 7.25 (d, *J* = 8.4 Hz, 1H), 7.16-7.09 (m, 2H), 6.97-6.91 (m, 1H), 6.34-6.27 (m, 2H), 6.05 (ddd, *J* = 17.2, 10.0, 8.4 Hz, 1H), 5.58 (s, 1H), 5.29 (dd, *J* = 11.6, 0.8 Hz, 1H), 5.23 (d, *J* = 17.2 Hz, 1H), 4.10 (d, *J* = 8.4 Hz, 1H), 3.64 (s, 3H), 3.63 (s, 3H), 1.41 (s, 3H). ^{13}C NMR (101 MHz, $CDCl_3$) δ 175.96, 156.06, 142.07, 137.33, 136.10, 135.63, 124.93, 121.09, 118.94, 118.88, 118.81, 109.95, 108.79, 107.19, 106.94, 62.33, 52.42, 48.84, 43.10, 28.91, 25.13. HRMS (ESI+) Calcd. For $C_{21}H_{23}N_2O_3$ ($[M+H]^+$): 351.1703, found: 351.1698. The product was analyzed by HPLC to determine the enantiomeric excess: 99% ee (Chiralpak AS-H, *i*-propanol/hexane = 2/98, flow rate 1.0 mL/min, λ = 234 nm); t_r = 5.78 and 6.61 min.

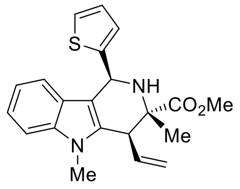

(1*R*,3*S*,4*R*)-**3r**

Methyl (1*R*,3*S*,4*R*)-3,5-dimethyl-1-(thiophen-2-yl)-4-vinyl-2,3,4,5-tetrahydro-1*H*-pyrido[4,3-*b*] indole-3-carboxylate (1*R*,3*S*,4*R*-**3r)**: Yield (84%); white solid; m.p. = 56 ºC. $[\alpha]^{20}_D$ = +209.8 (*c* 1.0, CH_2Cl_2); ^1H NMR (400 MHz, $CDCl_3$) δ 7.25-7.21 (m, 2H), 7.19 (d, *J* = 5.2 Hz, 1H), 7.14-7.09 (m, 1H), 7.05 (d, *J* = 8.0 Hz, 1H), 6.95 (dd, *J* = 5.2, 3.6 Hz, 1H), 6.90 (t, *J* = 7.2 Hz, 1H), 6.09 (ddd, *J* = 17.2, 10.0, 8.8 Hz, 1H), 5.77 (s, 1H), 5.30 (dd, *J* = 10.0, 1.2 Hz, 1H), 5.28-5.23 (m, 1H), 4.08 (d, *J* = 8.8 Hz, 1H), 3.64 (s, 3H), 3.63 (s, 3H), 1.39 (s, 3H). ^{13}C NMR (101 MHz, $CDCl_3$) δ 176.10, 149.42, 137.39, 136.45, 135.16, 125.83, 125.25, 125.18, 125.07, 121.03, 118.92, 118.84, 118.50, 109.50, 108.76, 62.50, 52.40, 50.80, 43.42, 28.91, 25.11. HRMS (ESI+) Calcd. For $C_{21}H_{23}N_2O_2S$ ($[M+H]^+$): 367.1475, found: 367.1473. The product was analyzed by HPLC to determine the enantiomeric

excess: 99% ee (Chiralpak AS-H, *i*-propanol/hexane = 2/98, flow rate 1.0 mL/min, λ = 234 nm); t_r = 5.86 and 6.84 min.

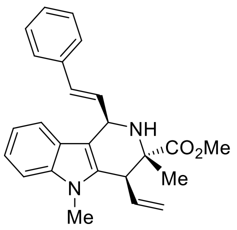

(1*R*,3*S*,4*R*)-**3s**

Methyl (1*R*,3*S*,4*R*)-3,5-dimethyl-1-((*E*)-styryl)-4-vinyl-2,3,4,5-tetrahydro-1*H*-pyrido[4,3-*b*] indole-3-carboxylate (1*R*,3*S*,4*R*-**3s)**: Yield (89%); white solid; m.p. = 62 ºC. $[\alpha]^{20}_D$ = +219.8 (*c* 1.0, CH_2Cl_2); ^1H NMR (400 MHz, $CDCl_3$) δ 7.48 (d, *J* = 8.0 Hz, 1H), 7.42 (d, *J* = 7.2 Hz, 2H), 7.31-7.18 (m, 4H), 7.17-7.12 (m, 1H), 6.99-6.94 (m, 1H), 6.87 (d, *J* = 15.6 Hz, 1H), 6.25 (dd, *J* = 15.6, 8.4 Hz, 1H), 6.00 (ddd, *J* = 17.2, 10.0, 8.4 Hz, 1H), 5.28 (dd, *J* = 10.0, 1.6 Hz, 1H), 5.19 (d, *J* = 17.2 Hz, 1H), 5.01 (d, *J* = 8.4 Hz, 1H), 4.07 (d, *J* = 8.8 Hz, 1H), 3.63 (s, 3H), 3.62 (s, 3H), 1.40 (s, 3H). ^{13}C NMR (101 MHz, $CDCl_3$) δ 176.22, 137.40, 136.93, 136.42, 134.97, 132.95, 131.47, 128.41, 127.38, 126.53, 125.28, 121.01, 119.18, 118.83, 118.45, 108.75, 108.21, 61.93, 54.08, 52.33, 43.19, 28.92, 25.16. HRMS (ESI+) Calcd. For $C_{25}H_{27}ClN_2O_2$ ($[M+H]^+$): 387.2067, found: 387.2072. The product was analyzed by HPLC to determine the enantiomeric excess: 99% ee (Chiralpak AS-H, *i*-propanol/hexane = 2/98, flow rate 1.0 mL/min, λ = 254 nm); t_r = 5.08 and 5.98 min.

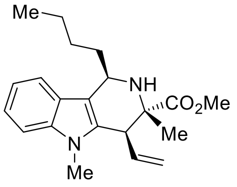

(1*R*,3*S*,4*R*)-**3t**

Methyl (1*R*,3*S*,4*R*)-1-butyl-3,5-dimethyl-4-vinyl-2,3,4,5-tetrahydro-1*H*-pyrido[4,3-*b*] indole-3-carboxylate (1*R*,3*S*,4*R*-**3t)**: Yield (83%); yellow oil; $[\alpha]^{20}_D$ = +387.818 (*c* 1.1, CH_2Cl_2); ^1H NMR (400 MHz, $CDCl_3$) δ 7.51 (d, *J* = 7.6 Hz, 1H), 7.26 (d, *J* = 8.0 Hz, 1H), 7.19-7.13 (m, 1H), 7.08-7.01 (m, 1H), 5.95 (ddd, *J* = 17.2, 10.0, 8.8 Hz, 1H), 5.23 (dd, *J* = 10.0, 1.6 Hz, 1H), 5.15 (d, *J* = 17.2 Hz, 1H), 4.42 (dd, *J* = 8.0, 1.6 Hz, 1H), 4.01 (d, *J* = 8.4 Hz, 1H), 3.60 (s, 3H), 3.55 (s, 3H),

2.12-2.03 (m, 1H), 1.82-1.71 (m, 1H), 1.50-1.38 (m, 2H), 1.37 (s, 3H), 1.34-1.25 (m, 2H), 0.90 (t, *J* = 7.2 Hz, 3H). ^{13}C NMR (101 MHz, $CDCl_3$) δ 176.43, 137.46, 136.77, 135.28, 125.01, 120.72, 119.03, 118.53, 118.14, 110.24, 108.84, 61.96, 52.15, 50.26, 43.51, 36.34, 28.81, 27.24, 25.26, 22.98, 14.11. HRMS (ESI+) Calcd. For $C_{21}H_{29}N_2O_2$ ($[M+H]^+$): 341.2224, found: 341.2223. The product was analyzed by HPLC to determine the enantiomeric excess: 99% ee (Chiralpak ID, *i*-propanol/hexane = 2/98, flow rate 0.5 mL/min, λ = 254 nm); t_r = 9.14 and 9.70 min.

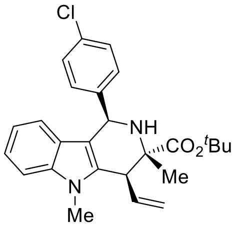

(1*R*,3*S*,4*R*)-**3u**

***tert*-butyl (1*R*,3*S*,4*R*)-1-(4-chlorophenyl)-3,5-dimethyl-4-vinyl-2,3,4,5-tetrahydro-1*H*-pyrido [4,3-*b*]indole-3-carboxylate (**1*R*,3*S*,4*R*-3u)**: Yield (87%); white solid; m.p. = 48 ºC. $[\alpha]^{20}_D$ = +97.2 (*c* 1.0, CH_2Cl_2); ^{1}H NMR (400 MHz, $CDCl_3$) δ 7.36-7.31 (m, 2H), 7.29-7.25 (m, 2H), 7.23 (d, *J* = 8.4 Hz, 1H), 7.09 (ddd, *J* = 8.0, 6.8, 1.2 Hz, 1H), 6.87-6.80 (m, 1H), 6.78 (d, *J* = 7.6 Hz, 1H), 6.07 (ddd, *J* = 17.2, 10.0, 8.4 Hz, 1H), 5.35 (s, 1H), 5.30-5.21 (m, 2H), 4.03 (d, *J* = 8.4 Hz, 1H), 3.66 (s, 3H), 1.35 (s, 3H), 1.30 (s, 9H). ^{13}C NMR (101 MHz, $CDCl_3$) δ 174.65, 142.89, 137.36, 137.23, 136.24, 132.98, 129.95, 128.52, 124.89, 120.86, 118.88, 118.71, 117.93, 108.83, 108.61, 81.22, 62.67, 55.31, 43.45, 28.82, 27.89, 24.76. HRMS (ESI+) Calcd. For $C_{26}H_{30}ClN_2O_2$ ($[M+H]^+$): 437.1990, found: 437.1997. The product was analyzed by HPLC to determine the enantiomeric excess: 99% ee (Chiralpak OD-H, *i*-propanol/hexane = 2/98, flow rate 0.5 mL/min, λ = 238 nm); t_r = 9.26 and 10.47 min.

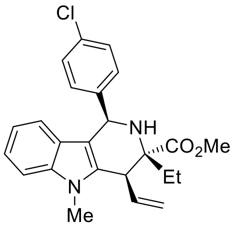

(1*R*,3*S*,4*R*)-**3v**

Methyl (1*R*,3*S*,4*R*)-1-(4-chlorophenyl)-3-ethyl-5-methyl-4-vinyl-2,3,4,5-tetrahydro-1*H*-pyrido

[4,3-*b*]indole-3-carboxylate (1*R*,3*S*,4*R*-3v): Yield (85%); white solid; m.p. = 78 ºC. $[\alpha]^{20}_{D}$ = +96.9 (*c* 1.3, CH_2Cl_2); ^{1}H NMR (400 MHz, $CDCl_3$) δ 7.35 (d, *J* = 8.4 Hz, 2H), 7.26 (d, *J* = 8.4 Hz, 2H), 7.23 (d, *J* = 8.4 Hz, 1H), 7.12-7.05 (m, 1H), 6.84 (t, *J* = 7.2 Hz, 1H), 6.77 (d, *J* = 7.6 Hz, 1H), 6.06 (ddd, *J* = 17.2, 10.0, 9.2 Hz, 1H), 5.35-5.21 (m, 3H), 4.15 (d, *J* = 8.8 Hz, 1H), 3.67 (s, 3H), 3.63 (s, 3H), 1.83 (dq, *J* = 14.8, 7.6 Hz, 1H), 1.64 (dq, *J* = 14.8, 7.6 Hz, 1H), 0.91 (t, *J* = 7.6 Hz, 3H). ^{13}C NMR (101 MHz, $CDCl_3$) δ 174.84, 142.79, 137.46, 136.72, 135.74, 133.05, 130.03, 128.51, 124.83, 120.97, 118.93, 118.78, 117.82, 109.26, 108.75, 66.37, 55.29, 52.04, 41.77, 30.69, 28.96, 7.72. HRMS (ESI+) Calcd. For $C_{24}H_{26}ClN_2O_2$ ($[M+H]^+$): 409.1677, found: 409.1682. The product was analyzed by HPLC to determine the enantiomeric excess: 99% ee (Chiralpak AS-H, *i*-propanol/hexane = 2/98, flow rate 1.0 mL/min, λ = 236 nm); t_r = 4.75 and 5.49 min.

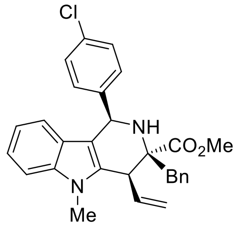

(1*R*,3*S*,4*R*)-**3w**

Methyl (1*R*,3*S*,4*R*)-3-benzyl-1-(4-chlorophenyl)-5-methyl-4-vinyl-2,3,4,5-tetrahydro-1*H*-pyrido [4,3-*b*]indole-3-carboxylate (1*R*,3*S*,4*R*-3w): Yield (86%); white solid; m.p. = 74 ºC. $[\alpha]^{20}_{D}$ = +11.2 (*c* 1.1, CH_2Cl_2); ^{1}H NMR (400 MHz, $CDCl_3$) δ 7.35 (d, *J* = 8.4 Hz, 2H), 7.29-7.20 (m, 6H), 7.11-7.05 (m, 3H), 6.87-6.77 (m, 2H), 6.23 (dt, *J* = 17.2, 9.6 Hz, 1H), 5.41 (d, *J* = 9.2 Hz, 1H), 5.37 (s, 1H), 5.31 (s, 1H), 4.22 (d, *J* = 9.2 Hz, 1H), 3.65 (s, 3H), 3.43 (s, 3H), 3.08 (d, *J* = 13.2 Hz, 1H), 2.94 (d, *J* = 13.2 Hz, 1H). ^{13}C NMR (101 MHz, $CDCl_3$) δ 174.32, 143.15, 137.49, 137.16, 135.58, 135.10, 132.92, 130.13, 129.68, 128.44, 128.39, 127.17, 124.83, 121.10, 118.97, 118.81, 118.40, 109.50, 108.76, 66.95, 55.16, 51.71, 44.51, 43.96, 28.98. HRMS (ESI+) Calcd. For $C_{29}H_{28}ClN_2O_2$ ($[M+H]^+$): 471.1834, found: 471.1830. The product was analyzed by HPLC to determine the enantiomeric excess: 99% ee (Chiralpak IE, *i*-propanol/hexane = 2/98, flow rate 1.0 mL/min, λ = 236 nm); t_r = 7.40 and 8.79 min.

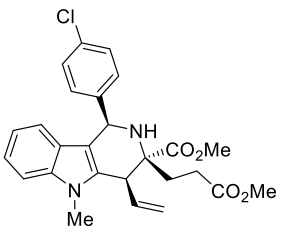

(1*R*,3*S*,4*R*)-**3x**

Methyl (1*R*,3*S*,4*R*)-1-(4-chlorophenyl)-3-(3-methoxy-3-oxopropyl)-5-methyl-4-vinyl-2,3,4,5-tetrahydro-1*H*-pyrido[4,3-*b*]indole-3-carboxylate (1*R*,3*S*,4*R*-**3x)**: Yield (82%); white solid; m.p. = 76 ºC. $[\alpha]^{20}_D$ = +100.2 (*c* 0.9, CH_2Cl_2); ^{1}H NMR (400 MHz, $CDCl_3$) δ 7.37-7.32 (m, 2H), 7.30-7.25 (m, 2H), 7.38 (d, *J* = 8.4 Hz, 1H), 7.13-7.07 (m, 1H), 6.88-6.81 (m, 1H), 6.78 (d, *J* = 8.0 Hz, 1H), 6.07 (ddd, *J* = 17.2, 10.0, 9.2 Hz, 1H), 5.33 (m, 3H), 4.13 (d, *J* = 8.8 Hz, 1H), 3.66 (s, 3H), 3.66 (s, 3H), 3.63 (s, 3H), 2.50-2.40 (m, 1H), 2.39-2.30 (m, 1H), 2.16-2.08 (m, 1H), 2.03-1.94 (m, 1H). ^{13}C NMR (101 MHz, $CDCl_3$) δ 174.30, 172.91, 142.49, 137.49, 136.36, 135.14, 133.16, 130.01, 128.55, 124.74, 121.16, 119.01, 118.88, 118.43, 109.25, 108.79, 65.03, 55.16, 52.36, 51.78, 42.25, 32.34, 28.98, 28.14. HRMS (ESI+) Calcd. For $C_{26}H_{28}ClN_2O_4$ ($[M+H]^+$): 467.1732, found: 467.1734. The product was analyzed by HPLC to determine the enantiomeric excess: 99% ee (Chiralpak IE, *i*-propanol/hexane = 5/95, flow rate 1.0 mL/min, λ = 236 nm); t_r = 13.91 and 16.61 min.

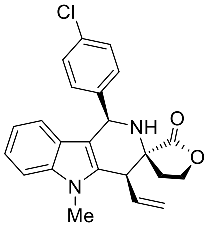

(1*R*,3*S*,4*R*)-**3y**

(1'*R*,3*S*,4'*R*)-1'-(4-chlorophenyl)-5'-Methyl-4'-vinyl-1',2',4,4',5,5'-hexahydro-2*H*-spiro [furan-3,3'-pyrido[4,3-*b*]indol]-2-one (1*R*,3*S*,4*R*-**3y)**: Yield (81%); white solid; m.p. = 60 ºC. $[\alpha]^{20}_D$ = +58.3 (*c* 1.2, CH_2Cl_2); ^{1}H NMR (400 MHz, $CDCl_3$) δ 7.37-7.31 (m, 2H), 7.31-7.27 (m, 2H), 7.26 (d, *J* = 8.4 Hz, 1H), 7.14-7.07 (m, 1H), 6.89-6.81 (m, 1H), 6.69 (d, *J* = 8.0 Hz, 1H), 6.03 (ddd, *J* = 17.6, 10.0, 8.0 Hz, 1H), 5.35 (dd, *J* = 10.0, 0.8 Hz, 1H), 5.25 (s, 1H), 5.19 (d, *J* = 13.2 Hz, 1H), 4.40 (td, *J* = 9.6, 6.0 Hz, 1H), 4.29 (td, *J* = 8.4, 2.4 Hz, 1H), 3.62 (s, 3H), 3.56 (d, *J* = 7.6 Hz, 1H), 2.46 (ddd, *J* = 13.6, 9.6, 8.4 Hz, 1H), 1.98 (ddd, *J* = 13.6, 6.0, 2.4 Hz, 1H). ^{13}C NMR (101 MHz, $CDCl_3$) δ 174.88, 141.15, 137.38, 135.68, 133.53, 133.40, 130.17, 128.74, 124.59, 121.07, 119.39, 119.31,

118.94, 109.70, 108.78, 64.88, 60.68, 55.03, 41.42, 36.21, 29.24. HRMS (ESI+) Calcd. For $C_{23}H_{22}ClN_2O_2$ ($[M+H]^+$): 393.1364, found: 393.1358. The product was analyzed by HPLC to determine the enantiomeric excess: 99% ee (Chiralpak AS-H, *i*-propanol/hexane = 8/92, flow rate 1.0 mL/min, λ = 254 nm); t_r = 28.69 and 34.54 min.

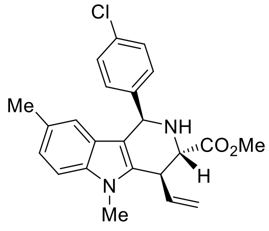

(1*R*,3*S*,4*R*)-**3z**

Methyl (1*R*,3*S*,4*R*)-1-(4-chlorophenyl)-5,8-dimethyl-4-vinyl-2,3,4,5-tetrahydro-1*H*-pyrido [4,3-*b*]indole-3-carboxylate (1*R*,3*S*,4*R*-**3z)**: Yield (92%); white solid; m.p. = 48 ºC. $[\alpha]^{20}_D$ = +97.1 (*c* 1.2, CH_2Cl_2); ^1H NMR (400 MHz, $CDCl_3$) δ 7.36-7.32 (m, 2H), 7.29-7.24 (m, 2H), 7.16 (d, *J* = 8.4 Hz, 1H), 6.96 (dd, *J* = 8.4, 1.2 Hz, 1H), 6.62 (s, 1H), 6.21 (ddd, *J* = 17.2, 10.0, 6.8 Hz, 1H), 5.38 (s, 1H), 5.30 (d, *J* = 10.0 Hz, 1H), 5.11 (d, *J* = 17.2 Hz, 1H), 4.18 (dd, *J* = 6.8, 1.2 Hz, 1H), 3.89 (d, *J* = 2.4 Hz, 1H), 3.70 (s, 3H), 3.63 (s, 3H), 2.26 (s, 3H). ^{13}C NMR (101 MHz, $CDCl_3$) δ 172.72, 142.25, 138.65, 135.64, 133.55, 133.06, 129.95, 128.53, 128.24, 125.16, 122.77, 118.69, 117.73, 109.38, 108.49, 60.16, 54.12, 52.29, 39.03, 29.28, 21.32. HRMS (ESI+) Calcd. For $C_{23}H_{24}ClN_2O_2$ ($[M+H]^+$): 395.1521, found: 395.1515. The product was analyzed by HPLC to determine the enantiomeric excess: 99% ee (Chiralpak AD-H, *i*-propanol/hexane = 2/98, flow rate 1.0 mL/min, λ = 226 nm); t_r = 10.57 and 14.27 min.

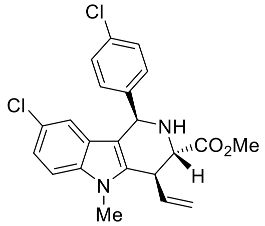

(1*R*,3*S*,4*R*)-**3A**

Methyl (1*R*,3*S*,4*R*)-8-chloro-1-(4-chlorophenyl)-5-methyl-4-vinyl-2,3,4,5-tetrahydro-1*H*-pyrido [4,3-*b*]indole-3-carboxylate (1*R*,3*S*,4*R*-**3A)**: Yield (96%); white solid; m.p. = 56 ºC. $[\alpha]^{20}_D$ = +54.3 (*c* 1.1, CH_2Cl_2); ^1H NMR (400 MHz, $CDCl_3$) δ 7.34-7.31 (m, 2H), 7.30-7.27 (m, 2H), 7.17 (d, *J* =

8.4 Hz, 1H), 7.07 (dd, *J* = 8.8, 2.0 Hz, 1H), 6.77 (d, *J* = 2.0 Hz, 1H), 6.22 (ddd, *J* = 16.8, 10.4, 6.4 Hz, 1H), 5.33 (d, *J* = 10.4 Hz, 2H), 5.12 (d, *J* = 16.8 Hz, 1H), 4.19 (d, *J* = 6.4 Hz, 1H), 3.91 (d, *J* = 2.0 Hz, 1H), 3.72 (s, 3H), 3.64 (s, 3H). ^{13}C NMR (101 MHz, $CDCl_3$) δ 172.66, 141.71, 138.33, 135.68, 134.95, 133.41, 129.85, 128.72, 125.91, 124.73, 121.48, 118.36, 117.94, 109.81, 109.76, 60.14, 53.97, 52.39, 39.01, 29.45. HRMS (ESI+) Calcd. For $C_{22}H_{21}Cl_2N_2O_2$ ([M+H]$^+$): 415.0975, found: 415.0977. The product was analyzed by HPLC to determine the enantiomeric excess: 99% ee (Chiralpak AD-H, *i*-propanol/hexane = 2/98, flow rate 1.0 mL/min, λ = 238 nm); t_r = 17.37 and 30.53 min.

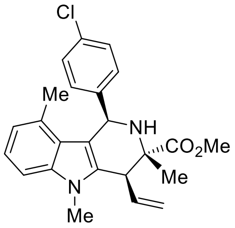

(1*R*,3*S*,4*R*)-**3B**

Methyl (1*R*,3*S*,4*R*)-1-(4-chlorophenyl)-3,5,9-trimethyl-4-vinyl-2,3,4,5-tetrahydro-1*H*-pyrido [4,3-*b*]indole-3-carboxylate (1*R*,3*S*,4*R*-**3B**)**: Yield (85%); white solid; m.p. = 78 ºC. $[\alpha]^{20}_D$ = +131.0 (*c* 1.0, CH_2Cl_2); ^{1}H NMR (400 MHz, $CDCl_3$) δ 7.28-7.21 (m, 2H), 7.20-7.16 (m, 2H), 7.11 (d, *J* = 8.0 Hz, 1H), 7.05-7.00 (m, 1H), 6.66 (d, *J* = 7.2 Hz, 1H), 6.01 (ddd, *J* = 17.2, 10.0, 8.4 Hz, 1H), 5.68 (s, 1H), 5.23-5.31 (m, 2H), 4.09 (d, *J* = 8.4 Hz, 1H), 3.65 (s, 3H), 3.62 (s, 3H), 1.88 (s, 3H), 1.34 (s, 3H). ^{13}C NMR (101 MHz, $CDCl_3$) δ 175.90, 145.14, 137.86, 136.45, 136.37, 132.89, 130.38, 129.40, 128.74, 124.54, 121.45, 120.64, 118.64, 108.70, 106.46, 62.31, 55.89, 52.39, 43.89, 28.93, 24.95, 21.06. HRMS (ESI+) Calcd. For $C_{24}H_{26}ClN_2O_2$ ([M+H]$^+$): 409.1677, found: 409.1679. The product was analyzed by HPLC to determine the enantiomeric excess: 99% ee (Chiralpak AS-H, *i*-propanol/hexane = 2/98, flow rate 1.0 mL/min, λ = 238 nm); t_r = 5.24 and 6.64 min.

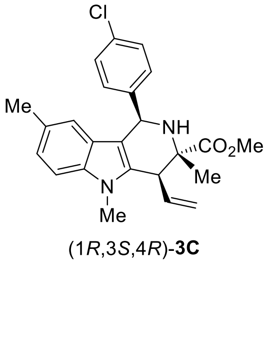

(1*R*,3*S*,4*R*)-**3C**

Methyl (1*R*,3*S*,4*R*)-1-(4-chlorophenyl)-3,5,8-trimethyl-4-vinyl-2,3,4,5-tetrahydro-1*H*-pyrido [4,3-*b*]indole-3-carboxylate (1*R*,3*S*,4*R*-**3C)**: Yield (84%); white solid; m.p. = 74 ºC. $[\alpha]^{20}_D$ = +141.3 (*c* 0.9, CH_2Cl_2); ^{1}H NMR (400 MHz, $CDCl_3$) δ 7.34 (d, *J* = 8.4 Hz, 2H), 7.27 (d, *J* = 8.4 Hz, 2H), 7.12 (d, *J* = 8.4 Hz, 1H), 6.92 (d, *J* = 8.4 Hz, 1H), 6.56 (s, 1H), 6.13-5.99 (m, 1H), 5.33 (s, 1H), 5.31-5.20 (m, 2H), 4.08 (d, *J* = 8.4 Hz, 1H), 3.63 (s, 3H), 3.62 (s, 3H), 2.24 (s, 3H), 1.36 (s, 3H). ^{13}C NMR (101 MHz, $CDCl_3$) δ 176.07, 142.84, 136.84, 135.88, 135.79, 133.00, 129.97, 128.54, 128.03, 125.04, 122.57, 118.67, 118.24, 108.46, 108.43, 62.42, 55.21, 52.33, 43.42, 28.95, 25.22, 21.32. HRMS (ESI+) Calcd. For $C_{24}H_{26}ClN_2O_2$ ($[M+H]^+$): 409.1677, found: 409.1682. The product was analyzed by HPLC to determine the enantiomeric excess: 99% ee (Chiralpak AS-H, *i*-propanol/hexane = 2/98, flow rate 1.0 mL/min, λ = 238 nm); t_r = 4.53 and 5.32 min.

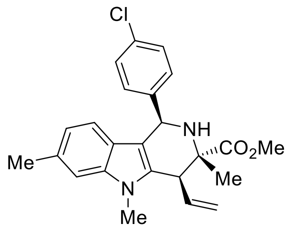

(1*R*,3*S*,4*R*)-**3D**

Methyl (1*R*,3*S*,4*R*)-1-(4-chlorophenyl)-3,5,7-trimethyl-4-vinyl-2,3,4,5-tetrahydro-1*H*-pyrido [4,3-*b*]indole-3-carboxylate (1*R*,3*S*,4*R*-**3D)**: Yield (88%); white solid; m.p. = 70 ºC. $[\alpha]^{20}_D$ = +113.3 (*c* 1.3, CH_2Cl_2); ^{1}H NMR (400 MHz, $CDCl_3$) δ 7.38-7.31 (m, 2H), 7.30-7.23 (m, 2H), 7.04 (s, 1H), 6.72-6.62 (m, 2H), 6.06 (ddd, *J* = 17.2, 10.0, 8.8 Hz, 1H), 5.34 (s, 1H), 5.32-5.21 (m, 2H), 4.09 (d, *J* = 8.8 Hz, 1H), 3.64 (s, 3H), 3.62 (s, 3H), 2.41 (s, 3H), 1.37 (s, 3H). ^{13}C NMR (101 MHz, $CDCl_3$) δ 176.12, 142.81, 137.87, 136.92, 135.09, 133.03, 130.93, 129.99, 128.53, 122.69, 120.44, 118.65, 118.22, 108.94, 108.91, 62.45, 55.21, 52.36, 43.36, 28.88, 25.26, 21.76. HRMS (ESI+) Calcd. For $C_{24}H_{26}ClN_2O_2$ ($[M+H]^+$): 409.1677, found: 409.1676. The product was analyzed by HPLC to determine the enantiomeric excess: 99% ee (Chiralpak AS-H, *i*-propanol/hexane = 2/98, flow rate 1.0 mL/min, λ = 238 nm); t_r = 4.66 and 5.71 min.

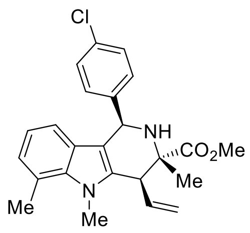

(1*R*,3*S*,4*R*)-**3E**

Methyl (1*R*,3*S*,4*R*)-1-(4-chlorophenyl)-3,5,7-trimethyl-4-vinyl-2,3,4,5-tetrahydro-1*H*-pyrido [4,3-*b*]indole-3-carboxylate (1*R*,3*S*,4*R*-**3E)**: Yield (95%); white solid; m.p. = 72 ºC. $[\alpha]^{20}_{D}$ = +40.9 (*c* 0.9, CH_2Cl_2); ^1H NMR (400 MHz, $CDCl_3$) δ 7.32 (d, *J* = 8.4 Hz, 2H), 7.25 (d, *J* = 8.4 Hz, 2H), 6.78 (d, *J* = 6.8 Hz, 1H), 6.69 (t, *J* = 7.6 Hz, 1H), 6.59 (d, *J* = 8.0 Hz, 1H), 6.06 (ddd, *J* = 17.2, 10.0, 8.4 Hz, 1H), 5.35-5.21 (m, 3H), 4.08 (d, *J* = 8.4 Hz, 1H), 3.92 (s, 3H), 3.65 (s, 3H), 2.73 (s, 3H), 1.37 (s, 3H). ^{13}C NMR (101 MHz, $CDCl_3$) δ 176.09, 142.76, 136.84, 136.29, 136.19, 133.03, 130.01, 128.54, 125.62, 124.26, 120.73, 118.91, 118.40, 117.12, 109.10, 62.53, 55.15, 52.37, 43.39, 32.04, 25.28, 20.34. HRMS (ESI+) Calcd. For $C_{24}H_{26}ClN_2O_2$ ($[M+H]^+$): 409.1677, found: 409.1677. The product was analyzed by HPLC to determine the enantiomeric excess: 99% ee (Chiralpak AS-H, *i*-propanol/hexane = 2/98, flow rate 1.0 mL/min, λ = 238 nm); t_r = 5.24 and 6.13 min.

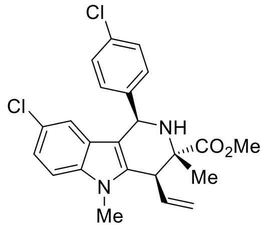

(1*R*,3*S*,4*R*)-**3F**

Methyl (1*R*,3*S*,4*R*)-8-chloro-1-(4-chlorophenyl)-3,5-dimethyl-4-vinyl-2,3,4,5-tetrahydro-1*H*-pyrido[4,3-*b*]indole-3-carboxylate (1*R*,3*S*,4*R*-**3F)**: Yield (93%); white solid; m.p. = 54 ºC. $[\alpha]^{20}_{D}$ = +181.9 (*c* 1.2, CH_2Cl_2); ^1H NMR (400 MHz, $CDCl_3$) δ 7.35-7.30 (m, 2H), 7.30-7.26 (m, 2H), 7.14 (d, *J* = 8.8 Hz, 1H), 7.04 (dd, *J* = 8.8, 2.0 Hz, 1H), 6.73 (d, *J* = 2.0 Hz, 1H), 6.06 (ddd, *J* = 17.2, 10.0, 8.8 Hz, 1H), 5.32 (dd, *J* = 10.0, 1.2 Hz, 1H), 5.29-5.24 (m, 2H), 4.09 (d, *J* = 8.8 Hz, 1H), 3.65 (s, 3H), 3.64 (s, 3H), 1.37 (s, 3H). ^{13}C NMR (101 MHz, $CDCl_3$) δ 176.00, 142.22, 137.18, 136.48, 135.90, 133.35, 129.86, 128.72, 125.79, 124.56, 121.30, 118.62, 118.30, 109.78, 108.81, 62.40, 55.05, 52.43, 43.54, 29.14, 25.11. HRMS (ESI+) Calcd. For $C_{23}H_{23}Cl_2N_2O_2$ ($[M+H]^+$): 429.1131, found: 429.1132. The product was analyzed by HPLC to determine the enantiomeric excess: 99% ee

(Chiralpak AS-H, *i*-propanol/hexane = 2/98, flow rate 1.0 mL/min, λ = 248 nm); t_r = 5.82 and 7.05 min.

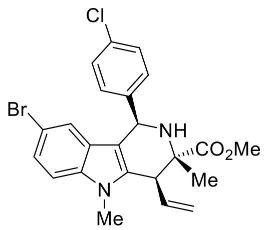

(1*R*,3*S*,4*R*)-**3G**

Methyl (1*R*,3*S*,4*R*)-8-bromo-1-(4-chlorophenyl)-3,5-dimethyl-4-vinyl-2,3,4,5-tetrahydro-1*H*-pyrido[4,3-*b*]indole-3-carboxylate (1*R*,3*S*,4*R*-**3G)**: Yield (80%); white solid; m.p. = 68 ºC. $[\alpha]^{20}_D$ = +395.6 (*c* 0.9, CH_2Cl_2); ^1H NMR (400 MHz, $CDCl_3$) δ 7.35-7.30 (m, 2H), 7.30-7.26 (m, 2H), 7.16 (dd, *J* = 8.8, 2.0 Hz, 1H), 7.09 (d, *J* = 8.8 Hz, 1H), 6.89 (d, *J* = 2.0 Hz, 1H), 6.05 (ddd, *J* = 17.2, 10.0, 8.8 Hz, 1H), 5.32 (dd, *J* = 10.0, 1.6 Hz, 1H), 5.30-5.23 (m, 2H), 4.09 (d, *J* = 8.7 Hz, 1H), 3.65 (s, 3H), 3.63 (s, 3H), 1.37 (s, 3H). ^{13}C NMR (101 MHz, $CDCl_3$) δ 175.96, 142.19, 137.04, 136.43, 136.15, 133.33, 129.83, 128.70, 126.44, 123.86, 121.32, 118.64, 112.15, 110.25, 108.72, 62.38, 55.01, 52.42, 43.50, 29.11, 25.08. HRMS (ESI+) Calcd. For $C_{23}H_{23}BrClN_2O_2$ ($[M+H]^+$): 473.0626, found: 473.0625. The product was analyzed by HPLC to determine the enantiomeric excess: 99% ee (Chiralpak AS-H, *i*-propanol/hexane = 2/98, flow rate 1.0 mL/min, λ = 246 nm); t_r = 5.81 and 7.19 min.

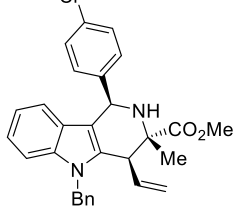

(1*R*,3*S*,4*R*)-**3H**

Methyl (1*R*,3*S*,4*R*)-5-benzyl-1-(4-chlorophenyl)-3-methyl-4-vinyl-2,3,4,5-tetrahydro-1*H*-pyrido [4,3-*b*]indole-3-carboxylate (1*R*,3*S*,4*R*-**3H)**: Yield (95%); white solid; m.p. = 52 ºC. $[\alpha]^{20}_D$ = +131.3 (*c* 1.1, CH_2Cl_2); ^1H NMR (400 MHz, $CDCl_3$) δ 7.41-7.35 (m, 2H), 7.32-7.26 (m, 4H), 7.26-7.24 (m, 1H), 7.12 (d, *J* = 8.0 Hz, 1H), 7.05-7.00 (m, 3H), 6.88-6.80 (m, 2H), 6.09 (ddd, *J* = 17.2, 10.0, 8.8 Hz, 1H), 5.43 (d, *J* = 0.8 Hz, 1H), 5.34-5.21 (m, 4H), 4.02 (d, *J* = 8.8 Hz, 1H), 3.50 (s, 3H), 1.32 (s,

3H). ^{13}C NMR (101 MHz, $CDCl_3$) δ 175.89, 142.69, 137.83, 137.16, 137.08, 135.40, 133.14, 130.07, 128.61, 128.58, 127.20, 126.24, 125.12, 121.44, 119.20, 119.17, 118.47, 109.80, 109.45, 62.36, 55.16, 52.10, 46.10, 43.50, 25.14. HRMS (ESI+) Calcd. For $C_{29}H_{28}ClN_2O_2$ ($[M+H]^+$): 471.1834, found: 471.1841. The product was analyzed by HPLC to determine the enantiomeric excess: 99% ee (Chiralpak AS-H, *i*-propanol/hexane = 2/98, flow rate 0.5 mL/min, λ = 228 nm); t_r = 10.25 and 12.71 min.

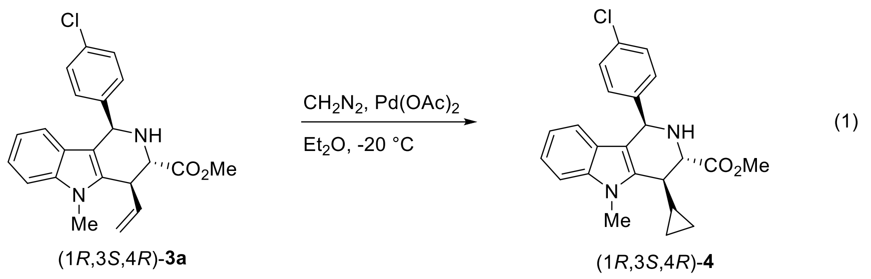

Supplementary Figure 5. Derivatization 1. Cyclopropanation of (1*R*,3*S*,4*R*)-**3a** for the synthesis of (1*R*,3*S*,4*R*)-**4**[5].

Fresh prepared diazomethane solution (0.5 M in Et_2O, 2 mL) and (1*R*,3*S*,4*R*)-**3a** (76.2 mg, 0.2 mmol) were added into a Schlenk tube. Under a positive nitrogen pressure, the reaction was cooled to -20 °C, and $Pd(OAc)_2$ (1.5 mg, 1 mol %) was added in one portion with gas evolution. After stirring for 1 hour in -20 °C, the reaction was moved to room temperature and stirred overnight. While the reaction was partly completed, the solvent was removed under reduced pressure and the residue was purified by a flash column chromatography (PE/EA = 6/1) to afford the product (1*R*,3*S*,4*R*)-**4**.

Methyl (1*R*,3*S*,4*R*)-1-(4-chlorophenyl)-4-cyclopropyl-5-methyl-2,3,4,5-tetrahydro-1*H*-pyrido [4,3-*b*]indole-3-carboxylate (1*R*,3*S*,4*R*-**4**): Yield (94%); white solid; m.p. = 62 ºC. $[\alpha]^{20}_D$ = +11.8 (*c* 1.0, CH_2Cl_2); ^1H NMR (400 MHz, $CDCl_3$) δ 7.38 (d, *J* = 8.4 Hz, 2H), 7.29-7.25 (m, 3H), 7.12 (t, *J* = 7.2 Hz, 1H), 6.86 (t, *J* = 7.2 Hz, 1H), 6.78 (d, *J* = 8.0 Hz, 1H), 5.44 (s, 1H), 4.12 (d, *J* = 1.2 Hz, 1H), 3.75 (s, 3H), 3.65 (s, 3H), 2.87 (d, *J* = 9.2 Hz, 1H), 2.27 (brs, 1H), 1.46-1.38 (m, 1H), 0.83-0.72 (m, 2H), 0.71-0.62 (m, 1H), 0.37 (td, *J* = 10.0, 5.2 Hz, 1H). ^{13}C NMR (101 MHz, $CDCl_3$) δ 173.33, 142.79, 137.33, 136.37, 133.08, 129.99, 128.58, 125.05, 121.13, 119.02, 118.93, 109.04, 108.85, 61.48, 54.47, 52.16, 38.96, 30.09, 17.27, 6.75, 3.77. HRMS (ESI+) Calcd. For $C_{23}H_{24}ClN_2O_2$ ($[M+H]^+$): 395.1521, found: 395.1513. The product was analyzed by HPLC to determine the enantiomeric excess: 98% ee (Chiralpak AD-H, *i*-propanol/hexane = 2/98, flow rate 1.0 mL/min, λ = 282 nm); t_r = 16.28 and 21.08 min.

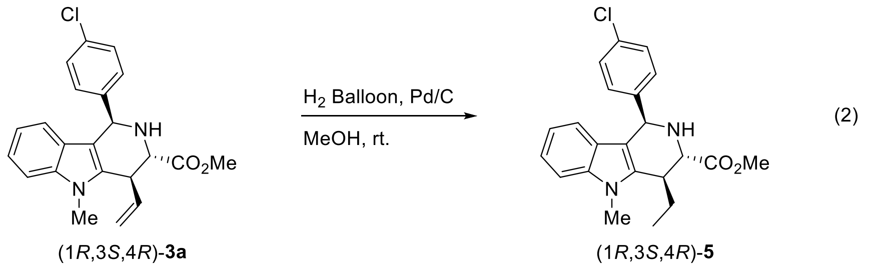

Supplementary Figure 6. Derivatization 2. Hydrogenation of (1*R*,3*S*,4*R*)-**3a** for the synthesis of (1*R*,3*S*,4*R*)-**5**[6].

Pd/C (21.0 mg, palladium on activated carbon, 10% Pd basis, 1 mol %) was added to a solution of (1*R*,3*S*,4*R*)-**3a** (76.2 mg, 0.2 mmol) in anhydrous MeOH (4 mL). The reaction mixture was stirred under H_2 atmosphere (1 atm) at room temperature for 6 hours. After the reaction was completed (monitored by TLC), the crude reaction mixture was filtrated with celite and washed with MeOH. The solvent was removed under reduced pressure, then the residue was purified by a flash column chromatography (PE/EA = 6/1) to afford the product (1*R*,3*S*,4*R*)-**5**.

Methyl (1*R*,3*S*,4*R*)-1-(4-chlorophenyl)-4-ethyl-5-methyl-2,3,4,5-tetrahydro-1*H*-pyrido [4,3-*b*]indole-3-carboxylate (1*R*,3*S*,4*R*-**5**)**: Yield (98%); white solid; m.p. = 58 ºC. $[\alpha]^{20}_{D}$ = -30.6 (*c* 0.8, CH_2Cl_2); ^{1}H NMR (400 MHz, $CDCl_3$) δ 7.35 (d, *J* = 8.0 Hz, 2H), 7.27 (d, *J* = 8.0 Hz, 2H), 7.24 (s, 1H), 7.10 (t, *J* = 7.6 Hz, 1H), 6.85 (t, *J* = 7.6 Hz, 1H), 6.77 (d, *J* = 8.0 Hz, 1H), 5.41 (s, 1H), 3.97 (s, 1H), 3.70 (s, 3H), 3.68 (s, 3H), 3.37 (d, *J* = 9.2 Hz, 1H), 2.09-2.01 (m, 1H), 1.94-1.85 (m, 1H), 1.16 (t, *J* = 7.6 Hz, 3H). ^{13}C NMR (101 MHz, $CDCl_3$) δ 173.98, 142.89, 137.20, 136.94, 133.03, 129.99, 128.52, 125.06, 120.95, 118.91, 118.90, 108.84, 108.71, 57.47, 54.27, 52.18, 36.02, 29.19, 27.32, 12.53. HRMS (ESI+) Calcd. For $C_{22}H_{23}ClN_2O_2$ ($[M+H]^+$): 383.1521, found: 383.1506. The product was analyzed by HPLC to determine the enantiomeric excess: 99% ee (Chiralpak IE, *i*-propanol/hexane = 2/98, flow rate 1.0 mL/min, λ = 232 nm); t_r = 9.86 and 10.79 min.

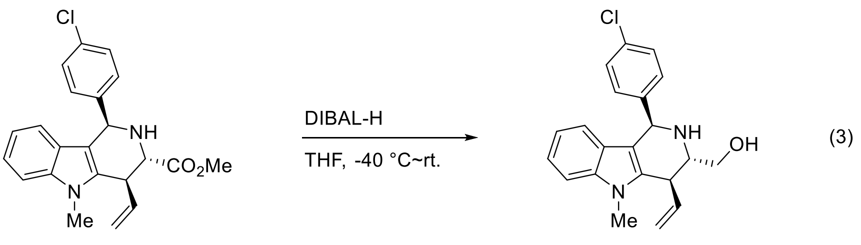

Supplementary Figure 7. Derivatization 3. Reduction of (1*R*,3*S*,4*R*)-**3a** for the synthesis of (1*R*,3*S*,4*R*)-**6**.

Under nitrogen atmosphere, to a solution of (1*R*,3*S*,4*R*)-**3a** (76.2 mg, 0.2 mmol) in anhydrous THF (2 mL) was added DIBAL-H (1.5 M, 0.4 mL, 0.6 mmol) dropwise at -40 ºC. After stirring at -40 ºC for 5 min, the reaction was then moved into room temperature and continuously stirred until

complete consumption of starting material (detected by TLC). The reaction mixture was quenched with H_2O, extracted with EA (× 3) and filtered through celite to remove the colloid. The organic layer was combined, washed with brine, dried over Na_2SO_4 before evaporation. Then the residue was purified by a flash column chromatography (pure EA) to afford the product (1*R*,3*S*,4*R*)-**6**.

((1*R*,3*S*,4*R*)-1-(4-Chlorophenyl)-5-methyl-4-vinyl-2,3,4,5-tetrahydro-1*H*-pyrido[4,3-*b*]indol-3-yl)methanol (1*R*,3*S*,4*R*-**6)**: Yield (97%); white solid; m.p. = 52 ºC. $[\alpha]^{20}_D$ = +113.0 (*c* 1.4, CH_2Cl_2); ^1H NMR (400 MHz, $CDCl_3$) δ 7.31-7.23 (m, 5H), 7.20-7.14 (m, 1H), 6.98-6.90 (m, 2H), 5.95 (ddd, *J* = 17.2, 10.0, 7.2 Hz, 1H), 5.28 (d, *J* = 10.0 Hz, 1H), 5.24 (s, 1H), 5.06 (d, *J* = 17.2 Hz, 1H), 3.70-3.64 (m, 2H), 3.60 (s, 3H), 3.47-3.40 (m, 1H), 3.13-3.04 (m, 1H). ^{13}C NMR (101 MHz, $CDCl_3$) δ 141.19, 138.60, 137.20, 134.88, 133.16, 129.85, 128.57, 124.98, 121.30, 119.16, 118.91, 118.16, 109.94, 108.82, 62.19, 57.23, 52.94, 39.12, 29.71. HRMS (ESI+) Calcd. For $C_{29}H_{28}ClN_2O_2$ ($[M+H]^+$): 353.1415, found: 353.1400. The product was analyzed by HPLC to determine the enantiomeric excess: 99% ee (Chiralpak AD-H, *i*-propanol/hexane = 15/85, flow rate 1.0 mL/min, λ = 282 nm); t_r = 12.37 and 17.49 min.

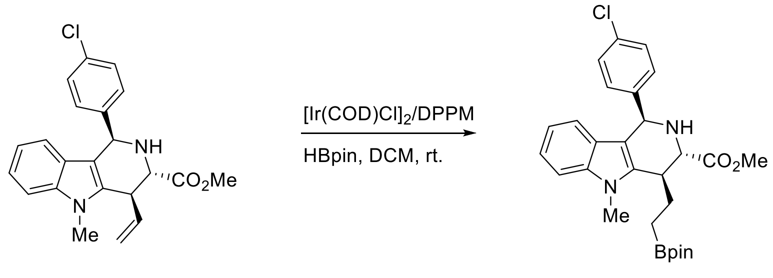

(1*R*,3*S*,4*R*)-**3a** (1*R*,3*S*,4*R*)-**7** (4)

Supplementary Figure 8. Derivatization 4. Hydroboration of (1*R*,3*S*,4*R*)-**3a** for the synthesis of (1*R*,3*S*,4*R*)-**7**[7].

To a solution of $[Ir(COD)Cl]_2$ (4.0 mg, 3 mol %) and bis(diphenylphosphino)methane (DPPM, 4.6 mg, 6 mol %) in anhydrous DCM (2 mL) was added (1*R*,3*S*,4*R*)-**3a** (76.2 g, 0.2 mmol) in one portion under a positive argon pressure. Then 4,4,5,5-tetramethyl-1,3,2-dioxaborolane (HBpin, 58 µL, 0.4 mmol) was added at room temperature and the resulting solution was stirred overnight. The reaction mixture was quenched with MeOH (1 mL) and concentrated under reduced pressure. The residue was purified by silica-gel flash column chromatography (PE/EA = 3/1, with 1% MeOH) to afford the product (1*R*,3*S*,4*R*)-**7**.

Methyl (1*R*,3*S*,4*R*)-1-(4-chlorophenyl)-5-methyl-4-(2-(4,4,5,5-tetramethyl-1,3,2-dioxaborolan-2-yl)ethyl)-2,3,4,5-tetrahydro-1*H*-pyrido[4,3-*b*]indole-3-carboxylate (1*R*,3*S*,4*R*-**7)**: Yield (86%); white solid; m.p. = 68 °C. $[\alpha]^{20}_{D}$ = -32.3 (*c* 0.9, CH_2Cl_2); ^1H NMR (400 MHz, $CDCl_3$) δ 7.38-7.33 (m, 2H), 7.28-7.25 (m, 2H), 7.24 (d, *J* = 8.0 Hz, 1H), 7.09 (ddd, *J* = 8.0, 6.8, 1.2 Hz, 1H), 6.84 (ddd, *J* = 8.0, 6.8, 1.2 Hz, 1H), 6.74 (dt, *J* = 8.0, 0.8 Hz, 1H), 5.41 (d, *J* = 1.2 Hz, 1H), 3.99 (d, *J* = 2.0 Hz, 1H), 3.75 (s, 3H), 3.66 (s, 3H), 3.45 (ddd, *J* = 8.8, 3.6, 1.6 Hz, 1H), 2.12-2.04 (m, 1H), 2.03-1.96 (m, 1H), 1.27 (s, 12H). ^{13}C NMR (101 MHz, $CDCl_3$) δ 174.02, 142.89, 137.22, 137.07, 132.99, 130.04, 128.50, 125.05, 120.86, 118.88, 118.82, 108.74, 108.68, 83.26, 57.10, 54.26, 52.10, 36.07, 29.16, 28.32, 24.94, 24.79. HRMS (ESI+) Calcd. For $C_{28}H_{35}BClN_2O_4$ ($[M+H]^+$): 509.2373, found: 509.2381. The product was analyzed by HPLC to determine the enantiomeric excess: 98% ee (Chiralpak OD-H, *i*-propanol/hexane = 5/95, flow rate 1.0 mL/min, λ = 228 nm); t_r = 9.62 and 15.53 min.

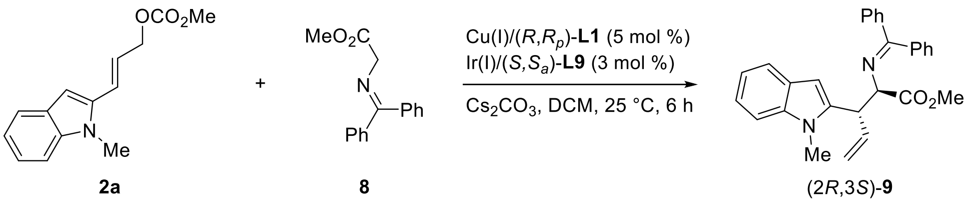

Supplementary Figure 9. Mechanism validation 1. Standard reaction of ketoimine ester **8** to synthesize (2*R*,3*S*)-**9**.

A flame dried Schlenk tube **A** was cooled to room temperature and filled with N_2. To this flask were added $[Ir(COD)Cl]_2$ (0.003 mmol, 1.5 mol %), (*S*,*S*$_a$)-Me-THQphos-**L9** (0.006 mmol, 3.0 mol %), degassed THF (0.5 mL) and degassed *n*-propylamine (0.5 mL). The reaction mixture was heated at 50 ºC for 30 min and then the volatile solvents were removed under vacuum to gain a pale yellow solid. Meanwhile, $Cu(MeCN)_4BF_4$ (0.01 mmol, 5 mol %) and (*R*,*R*$_p$)-^{i}Pr-Phosferrox-**L1** (0.011 mmol, 5.5 mol %) were dissolved in 1.0 mL of CH_2Cl_2 in a Schlenk tube **B**, and stirred at room temperature for about 30 min. Substrate **2a** (0.20 mmol), ketoimine esters **8** (0.30 mmol), CH_2Cl_2 (1.0 mL) and Cs_2CO_3 (0.40 mmol) were added into the Schlenk tube **A** and filled with N_2. The Cu/**L1** complex solution was then transferred from the Schlenk tube **B** to the Schlenk tube **A** *via* syringe. Finally, the reaction mixture was continuously stirred at room temperature under N_2 atmosphere. While the starting material was consumed (monitored by TLC), the solvent was removed by rotary evaporation to obtain a crude mixture, which was purified by flash column chromatography (PE/EA = 8/1) to give the product (2*R*,3*S*)-**9**.

Methyl (2*R*,3*S*)-2-((diphenylmethylene)amino)-3-(1-methyl-1*H*-indol-2-yl)pent-4-enoate (2*R*,3*S*-**9)**: Yield (99%); white solid; m.p. = 52 ºC. $[\alpha]^{20}_D$ = +2.0 (*c* 0.4, CH_2Cl_2); ^{1}H NMR (400 MHz, $CDCl_3$) δ 7.53-7.46 (m, 3H), 7.41-7.27 (m, 4H), 7.25-7.19 (m, 3H), 7.12 (t, *J* = 7.6 Hz, 1H), 7.04 (t, *J* = 7.6 Hz, 1H), 6.82 (d, *J* = 6.0 Hz, 2H), 6.24 (s, 1H), 5.89 (ddd, *J* = 17.6, 10.0, 8.4 Hz, 1H), 5.12 (d, *J* = 8.4 Hz, 1H), 5.08 (s, 1H), 4.55 (d, *J* = 8.4 Hz, 1H), 4.44 (t, *J* = 8.4 Hz, 1H), 3.69 (s, 3H), 3.67 (s, 3H). ^{13}C NMR (101 MHz, $CDCl_3$) δ 171.39, 171.21, 139.19, 138.71, 137.02, 136.22, 135.62, 130.32, 128.78, 128.64, 128.10, 128.07, 127.87, 127.67, 120.71, 119.99, 119.08, 117.40, 108.93, 100.41, 70.41, 52.04, 45.54, 29.90. HRMS (ESI+) Calcd. For $C_{28}H_{26}N_2O_2$ ($[M+H]^+$): 423.2067, found: 423.2060. The product was analyzed by HPLC to determine the enantiomeric excess: 99% ee (Chiralpak OD-H, *i*-propanol/hexane = 2/98, flow rate 1.0 mL/min, λ = 226 nm); t_r = 6.31 and 14.45 min.

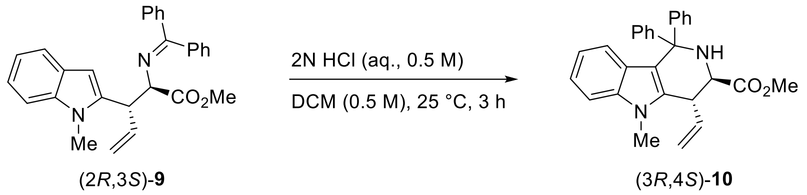

Supplementary Figure 10. Mechanism validation 2. Cyclization of (2*R*,3*S*)-**9** to synthesize (3*R*,4*S*)-**10**.

Diluted 2N HCl (aq., 2 mL) was added to a solution of (2*R*,3*S*)-**9** (84.4 mg, 0.2 mmol) in CH_2Cl_2 (2 mL) at room temperature. After vigorously stirring for 3 hours, the organic layer was separated and the aqueous layer was extracted with CH_2Cl_2. The organic layer was combined, washed with brine and dried over Na_2SO_4. After filtration and evaporation, the residue was purified by a flash column chromatography (PE/EA = 8/1) to afford the product (3*R*,4*S*)-**10**.

Methyl (3*R*,4*S*)-5-methyl-1,1-diphenyl-4-vinyl-2,3,4,5-tetrahydro-1*H*-pyrido[4,3-*b*]indole-3-carboxylate (3*R*,4*S*-**10)**: Yield (46%); white solid; m.p. = 56 ºC. $[\alpha]^{20}_D$ = -166.3 (*c* 0.9, CH_2Cl_2); ^{1}H NMR (400 MHz, $CDCl_3$) δ 7.60 (d, *J* = 7.2 Hz, 2H), 7.36-7.24 (m, 4H), 7.21-7.16 (m, 3H), 7.11 (t, *J* = 7.6 Hz, 1H), 7.09-7.03 (m, 2H), 6.81 (t, *J* = 7.6 Hz, 1H), 6.60 (d, *J* = 8.0 Hz, 1H), 5.76 (ddd, *J* = 18.0, 10.0, 8.4 Hz, 1H), 5.24-5.13 (m, 2H), 4.12 (t, *J* = 8.4 Hz, 1H), 3.70 (s, 3H), 3.64 (s, 3H), 3.61 (d, *J* = 8.4 Hz, 1H). ^{13}C NMR (101 MHz, $CDCl_3$) δ 172.69, 146.33, 144.40, 137.61, 137.06, 135.00, 128.99, 128.59, 127.99, 127.88, 127.10, 127.01, 126.20, 120.95, 120.50, 119.27, 118.54, 116.21, 108.65, 65.20, 59.94, 52.01, 41.26, 30.70. HRMS (ESI+) Calcd. For $C_{28}H_{26}N_2O_2$ ($[M+H]^+$): 423.2067, found: 423.2061. The product was analyzed by HPLC to determine the enantiomeric excess: 99% ee (Chiralpak AD-H, *i*-propanol/hexane = 2/98, flow rate 1.0 mL/min, λ = 226 nm); t_r = 5.68 and 16.35 min.

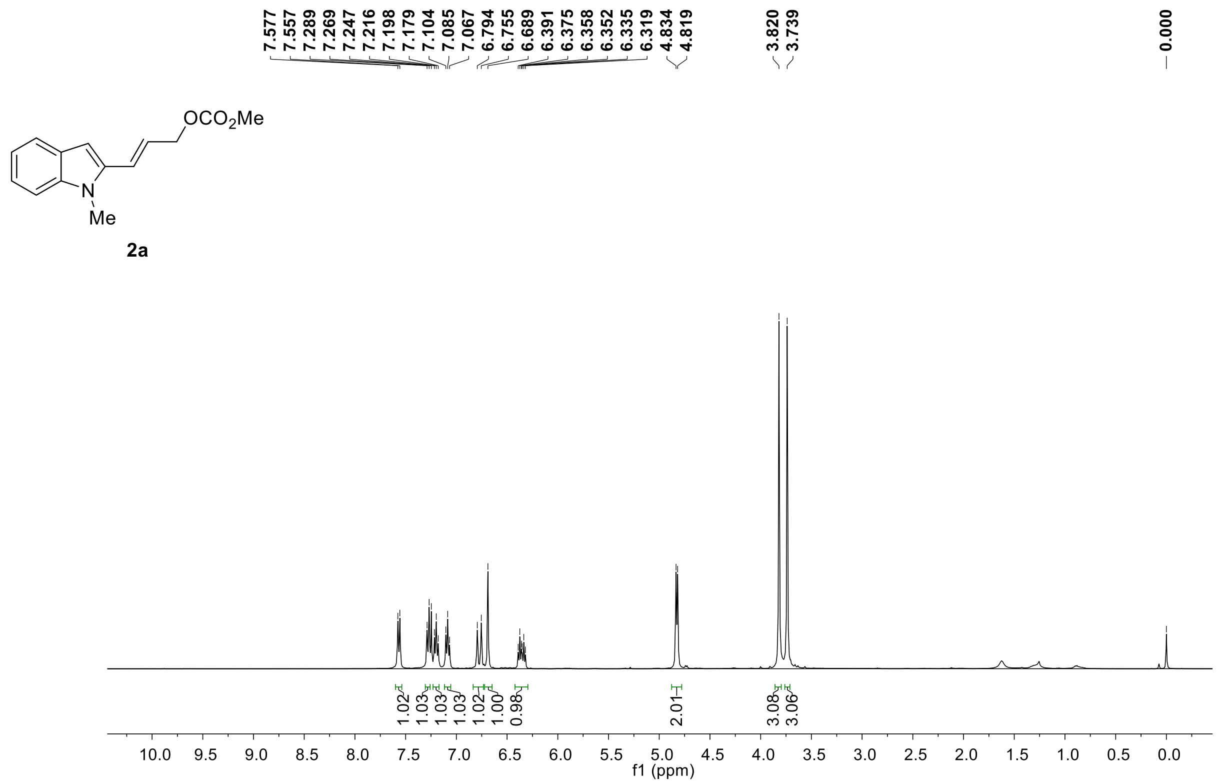

Supplementary Figure 11. ^{1}H NMR spectrum of **2a**

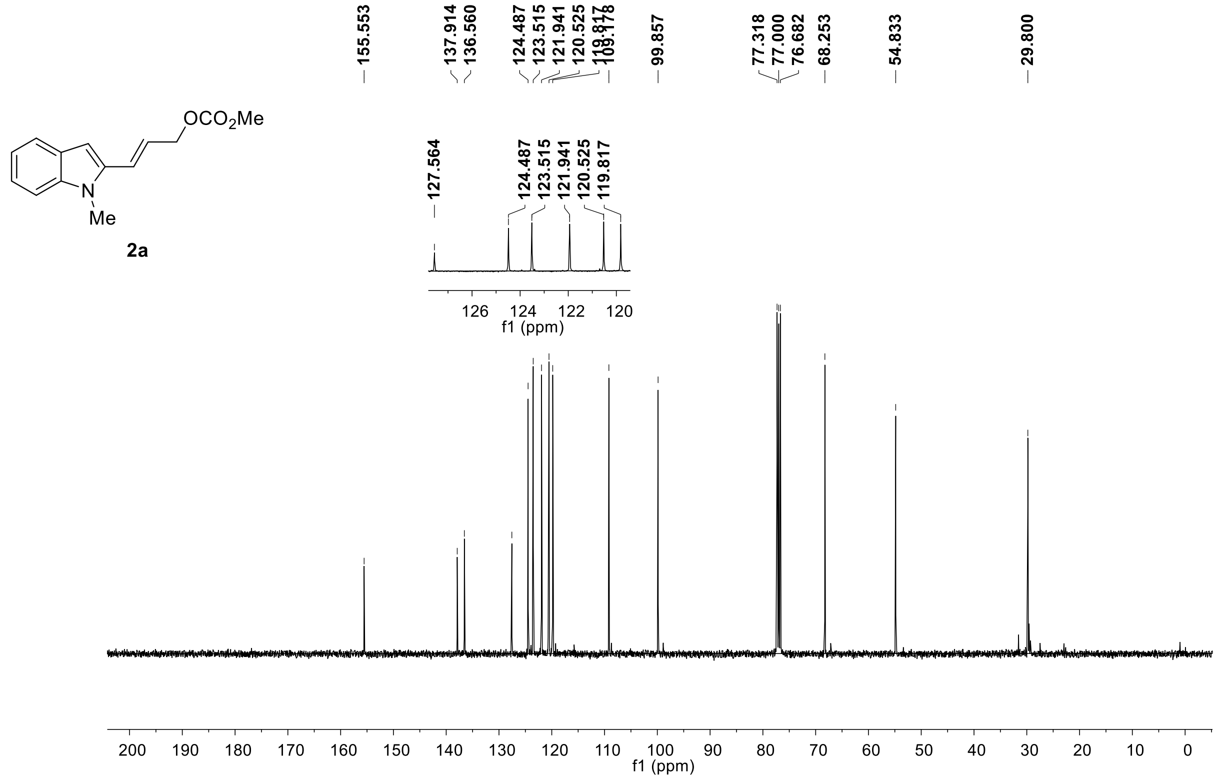

Supplementary Figure 12. ^{13}C NMR spectrum of **2a**

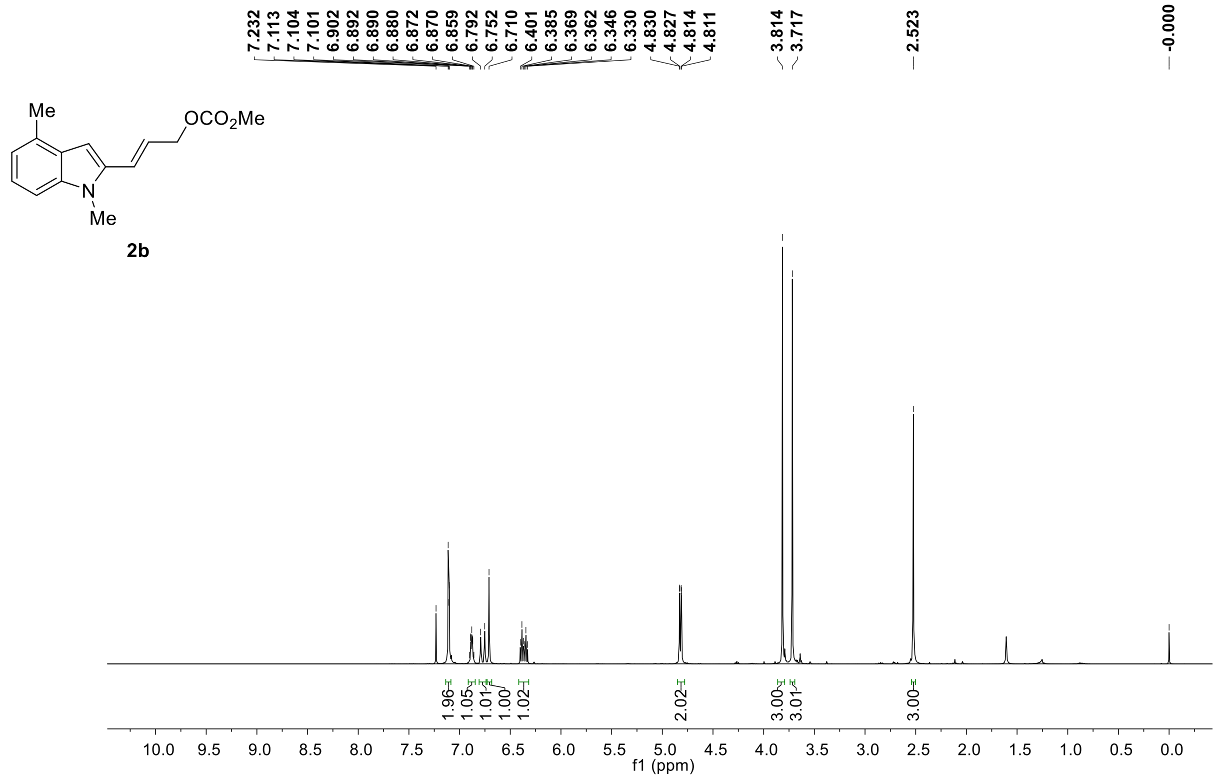

Supplementary Figure 13. ^{1}H NMR spectrum of **2b**

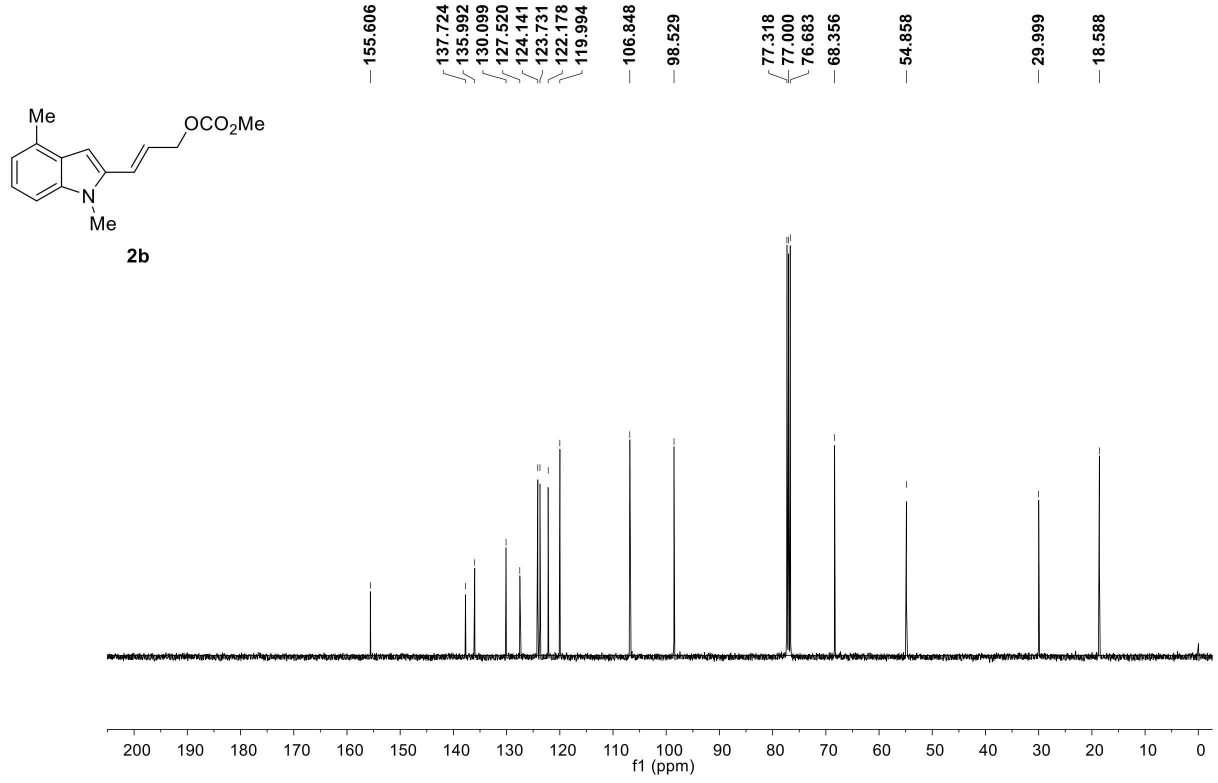

Supplementary Figure 14. ^{13}C NMR spectrum of **2b**

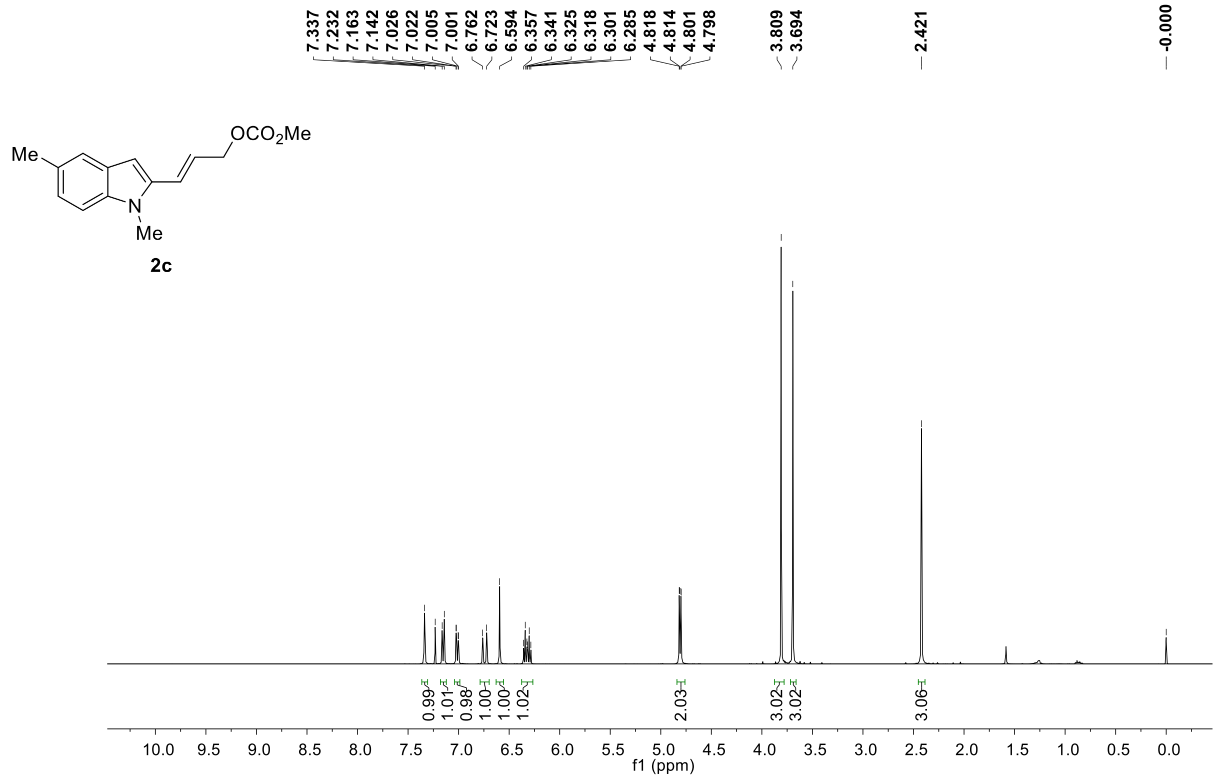

Supplementary Figure 15. ^{1}H NMR spectrum of **2c**

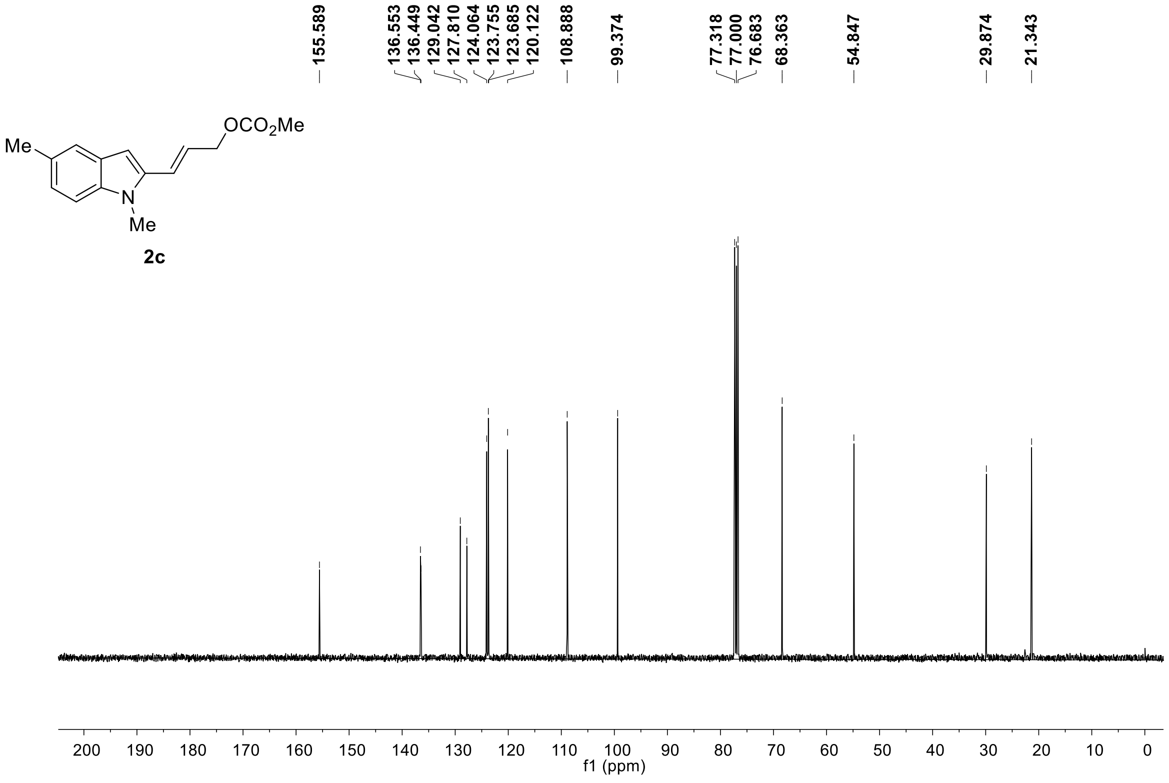

Supplementary Figure 16. ^{13}C NMR spectrum of **2c**

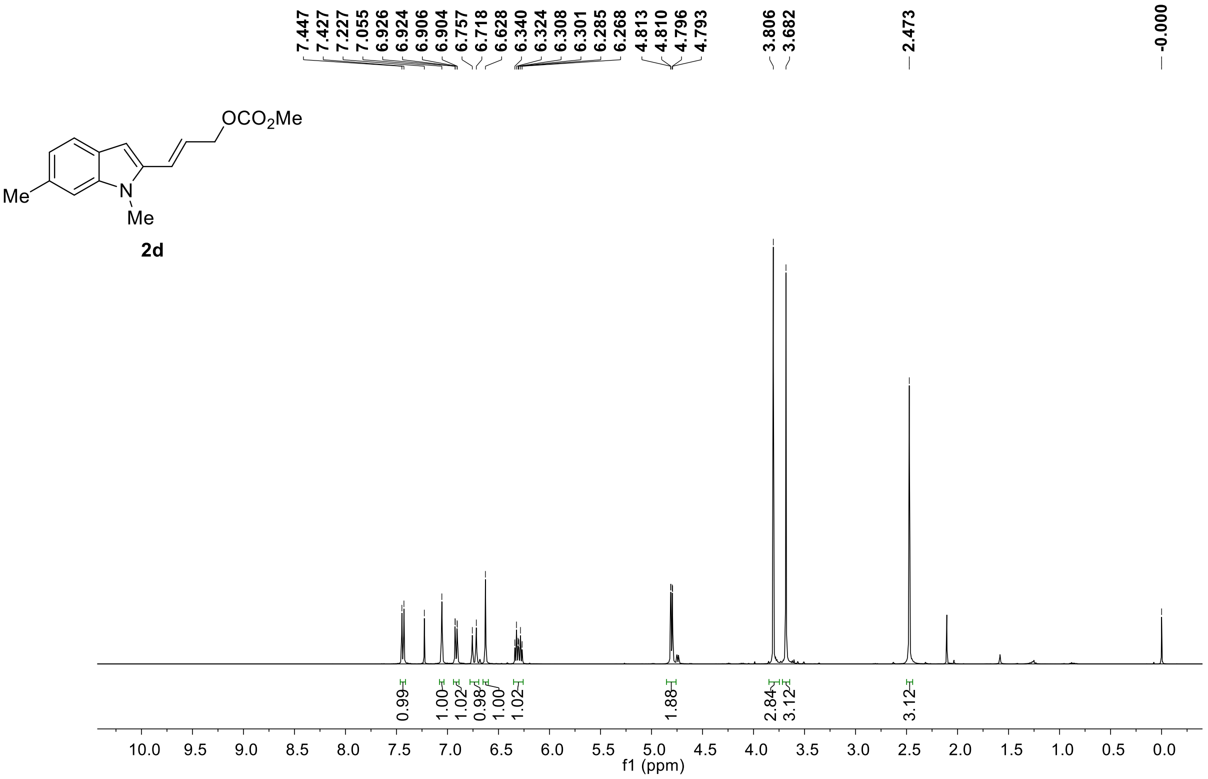

Supplementary Figure 17. ^{1}H NMR spectrum of **2d**

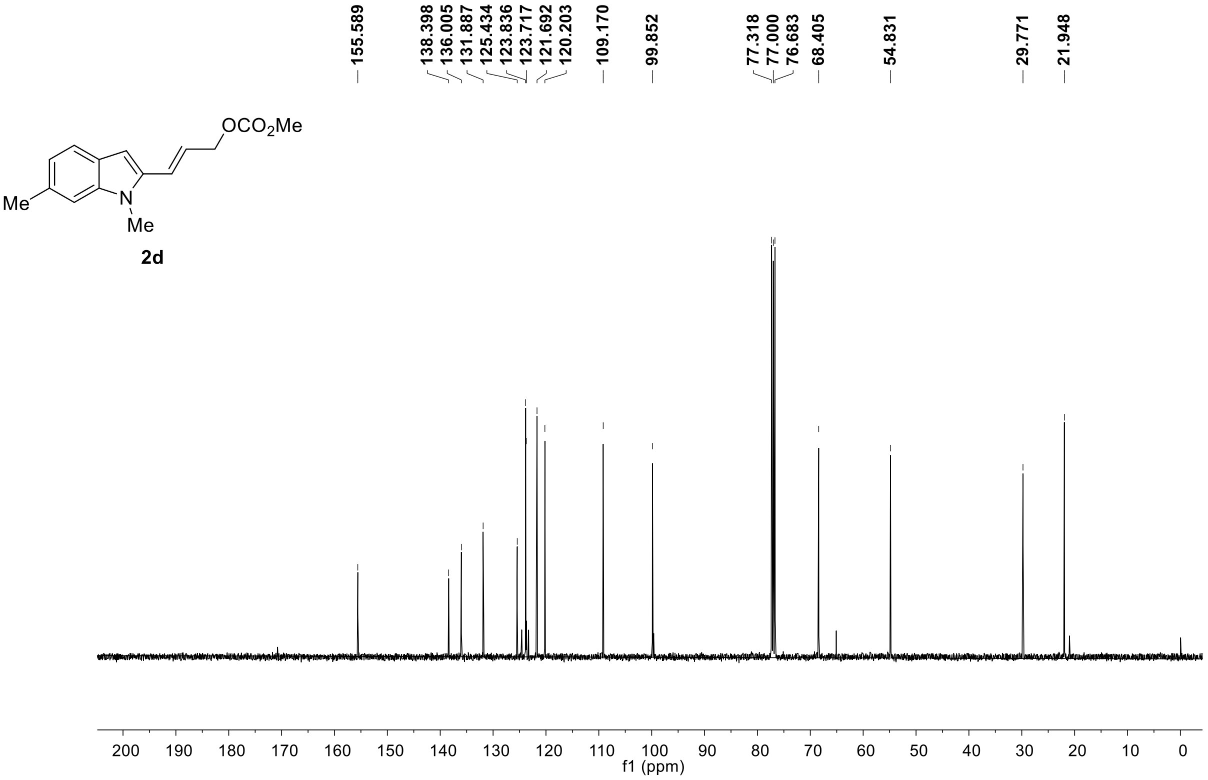

Supplementary Figure 18. ^{13}C NMR spectrum of **2d**

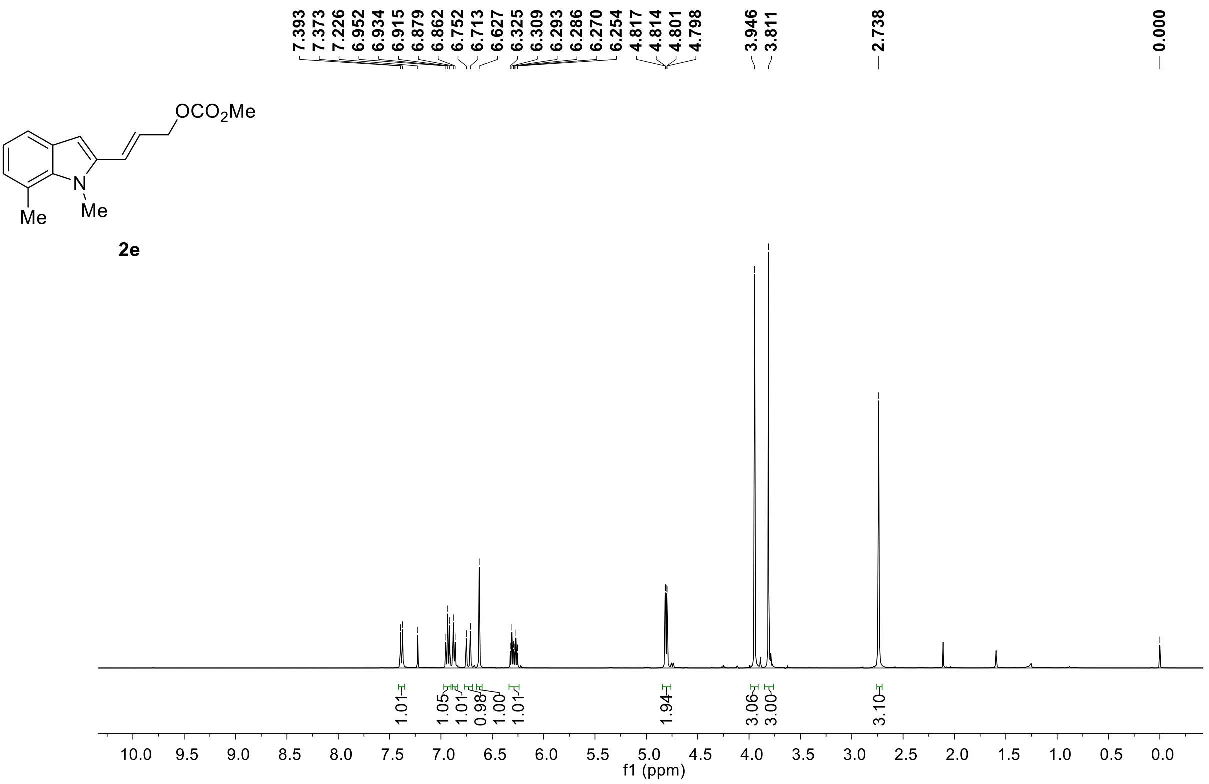

Supplementary Figure 19. ^{1}H NMR spectrum of **2e**

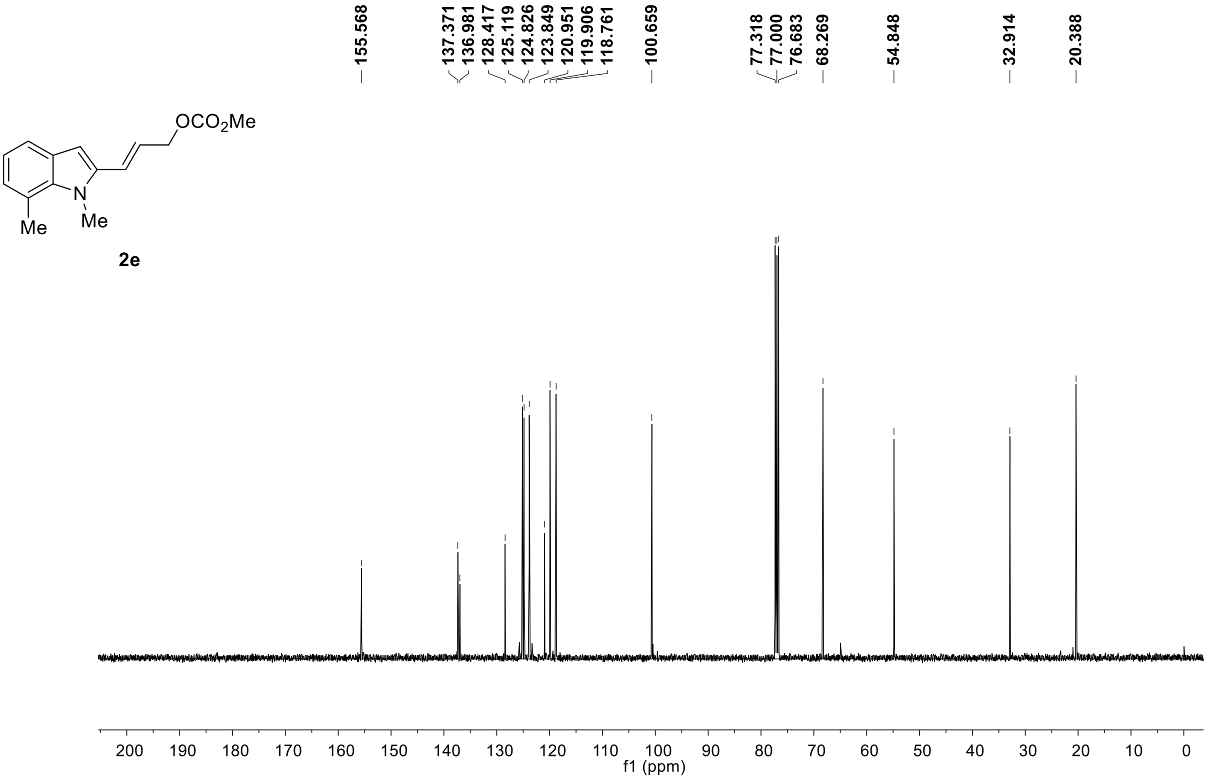

Supplementary Figure 20. ^{13}C NMR spectrum of **2e**

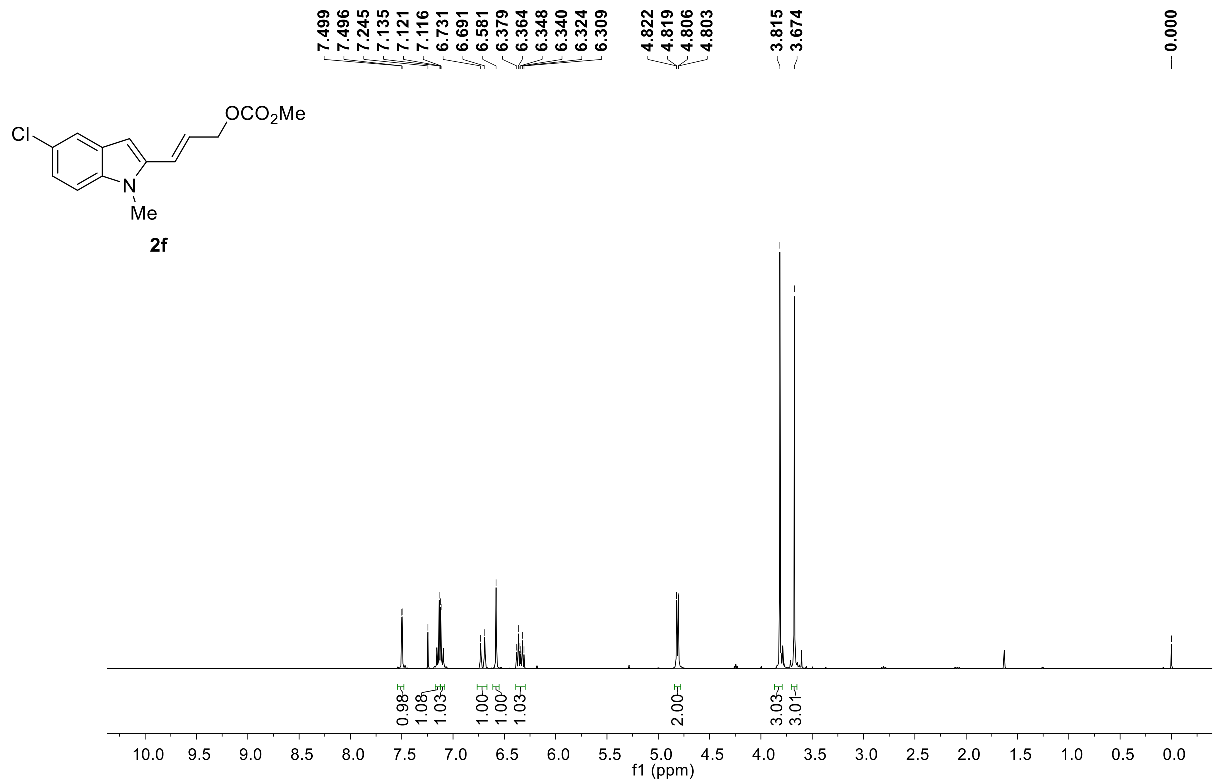

Supplementary Figure 21. ^{1}H NMR spectrum of **2f**

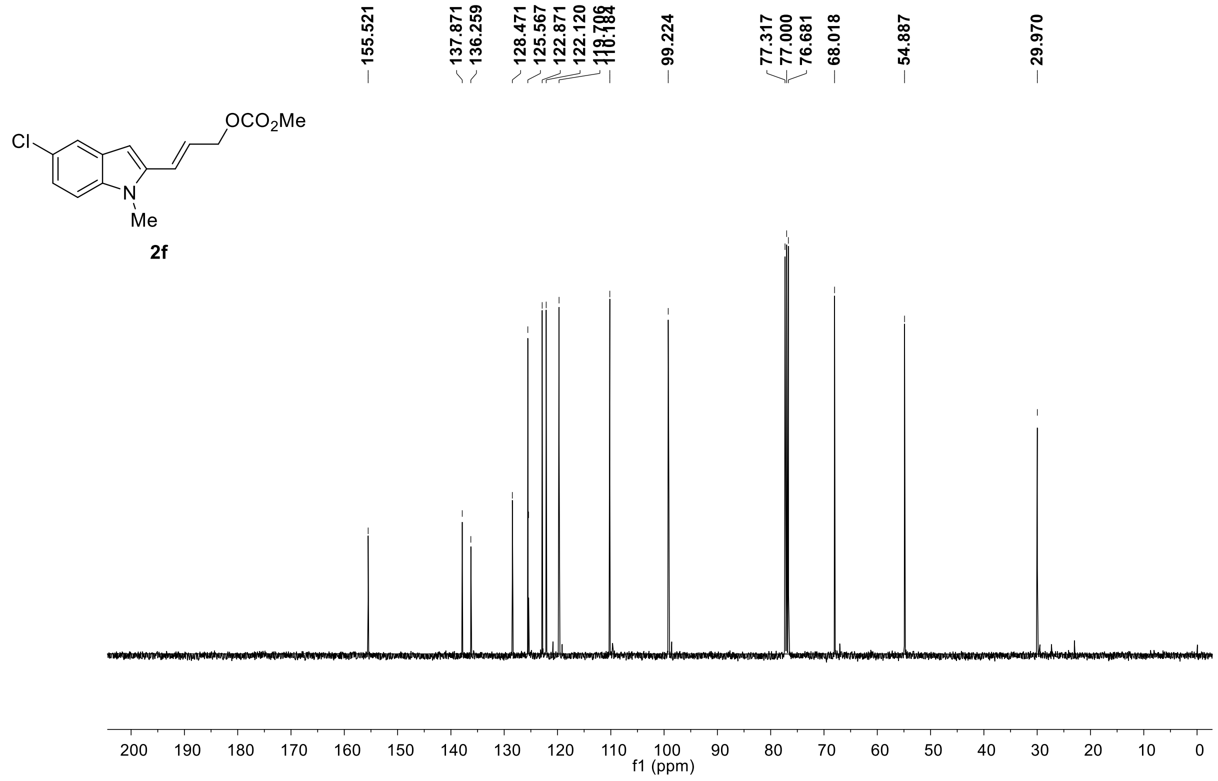

Supplementary Figure 22. ^{13}C NMR spectrum of **2f**

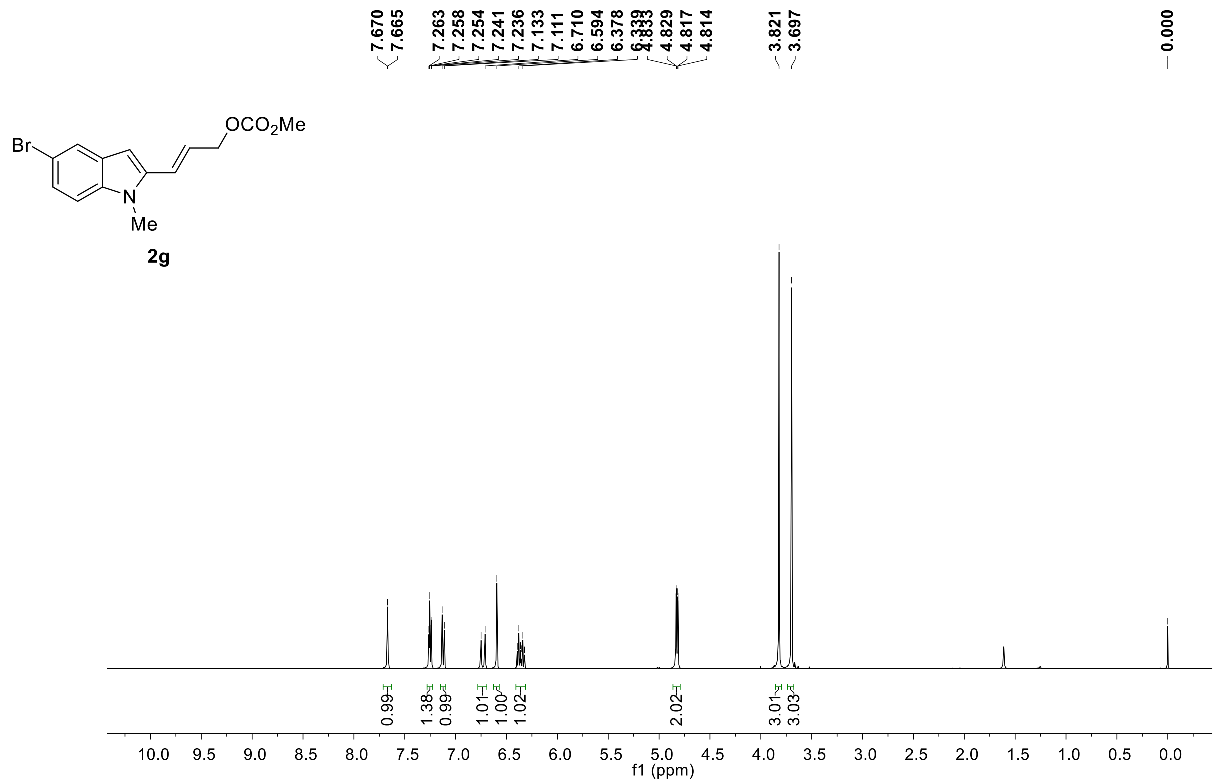

Supplementary Figure 23. ^{1}H NMR spectrum of **2g**

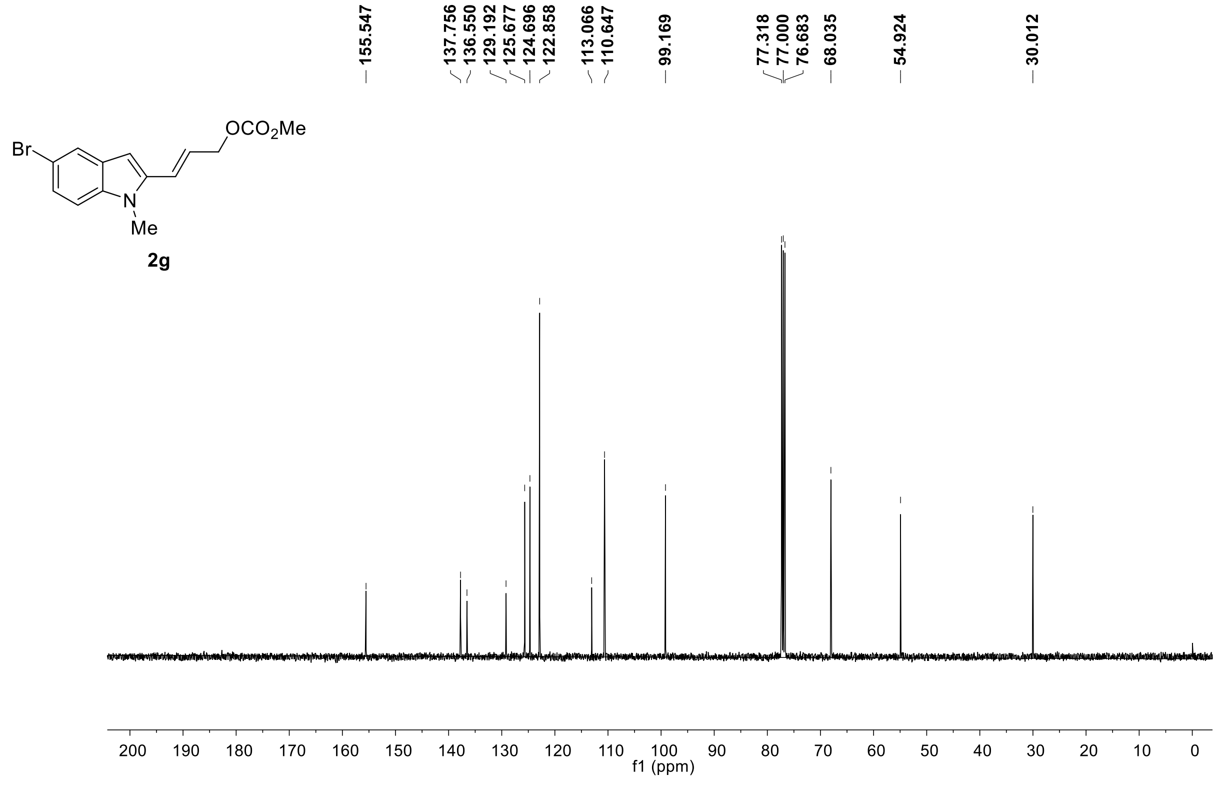

Supplementary Figure 24. ^{13}C NMR spectrum of **2g**

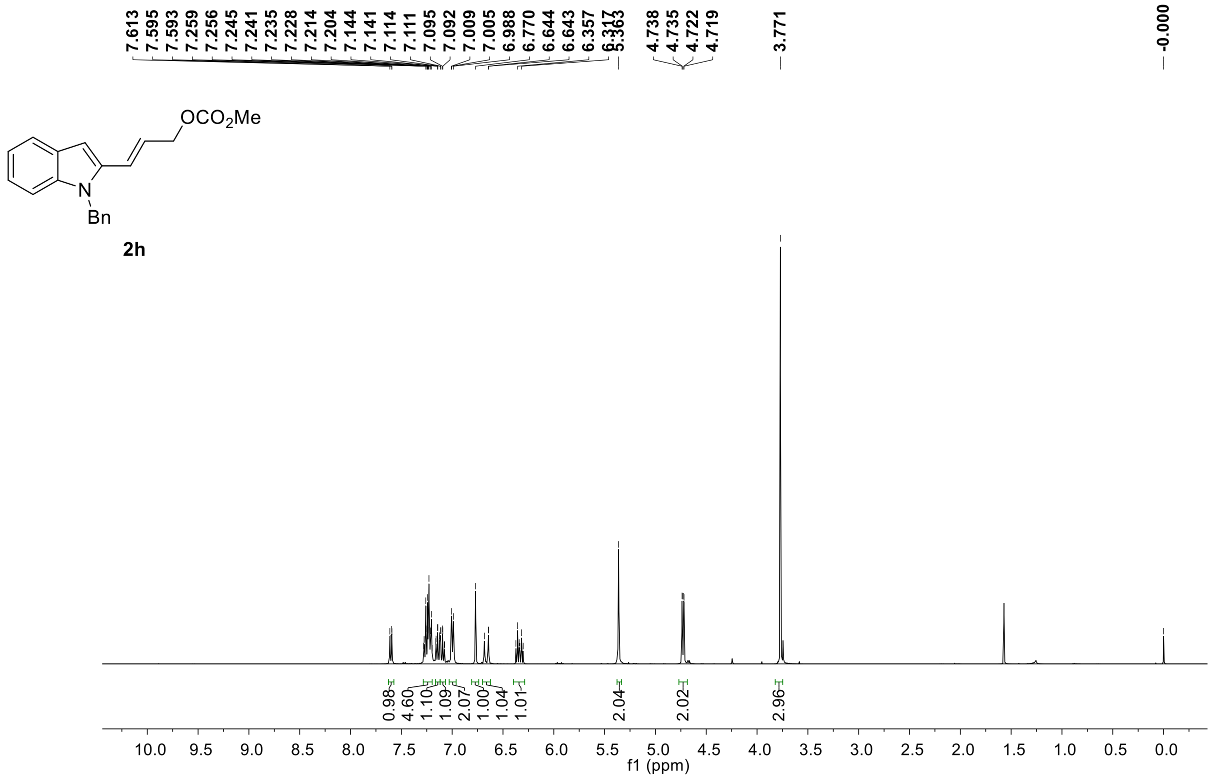

Supplementary Figure 25. ^{1}H NMR spectrum of **2h**

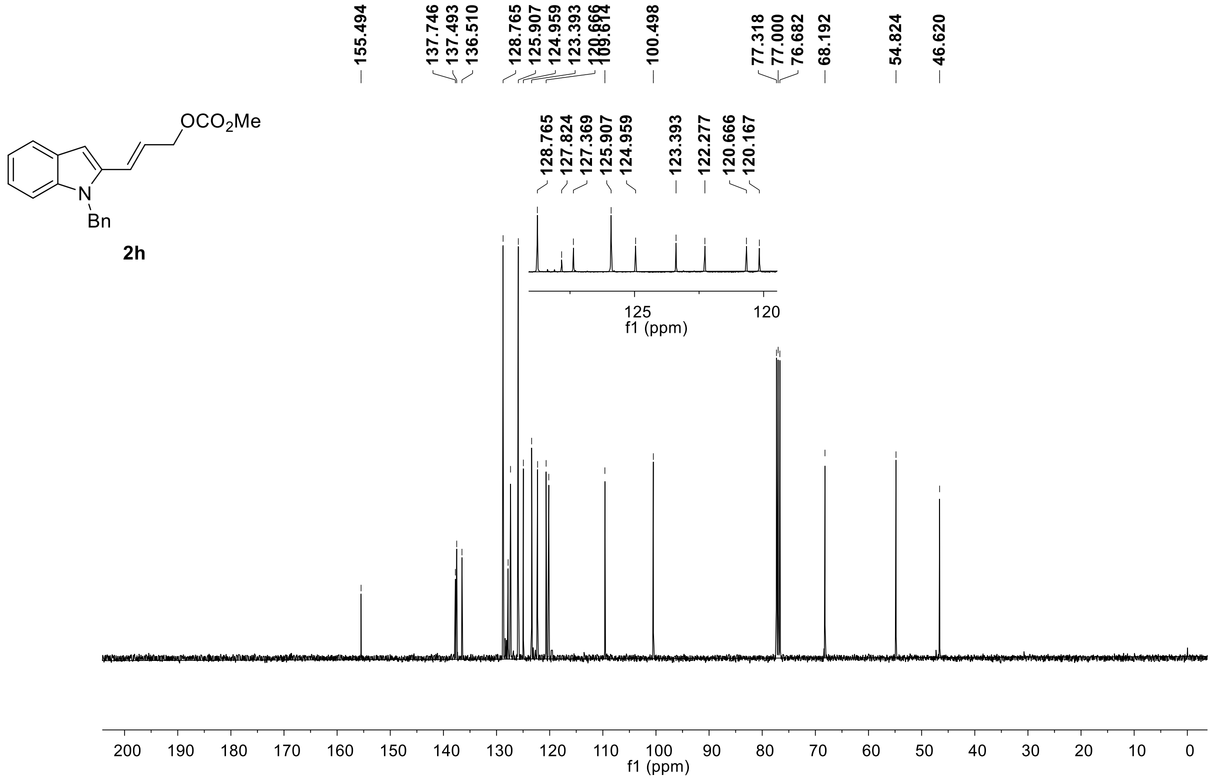

Supplementary Figure 26. ^{13}C NMR spectrum of **2h**

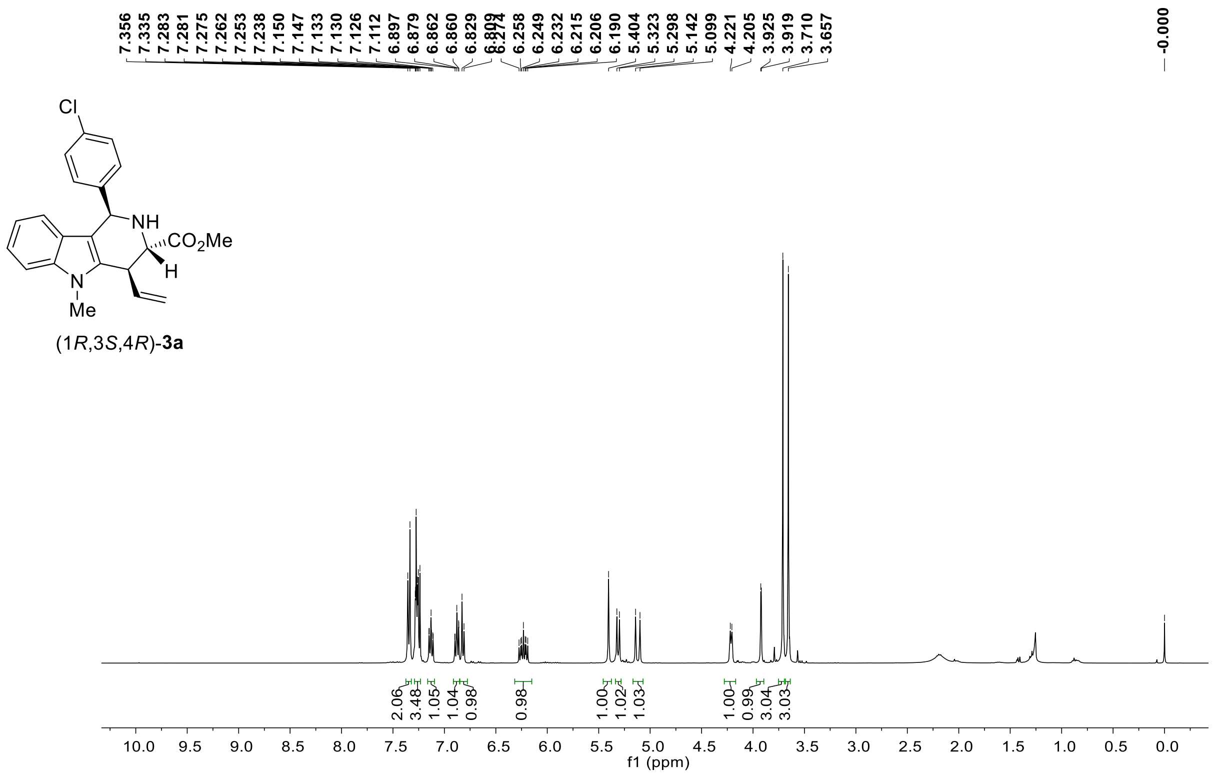

Supplementary Figure 27. ^{1}H NMR spectrum of (1*R*,3*S*,4*R*)-**3a**

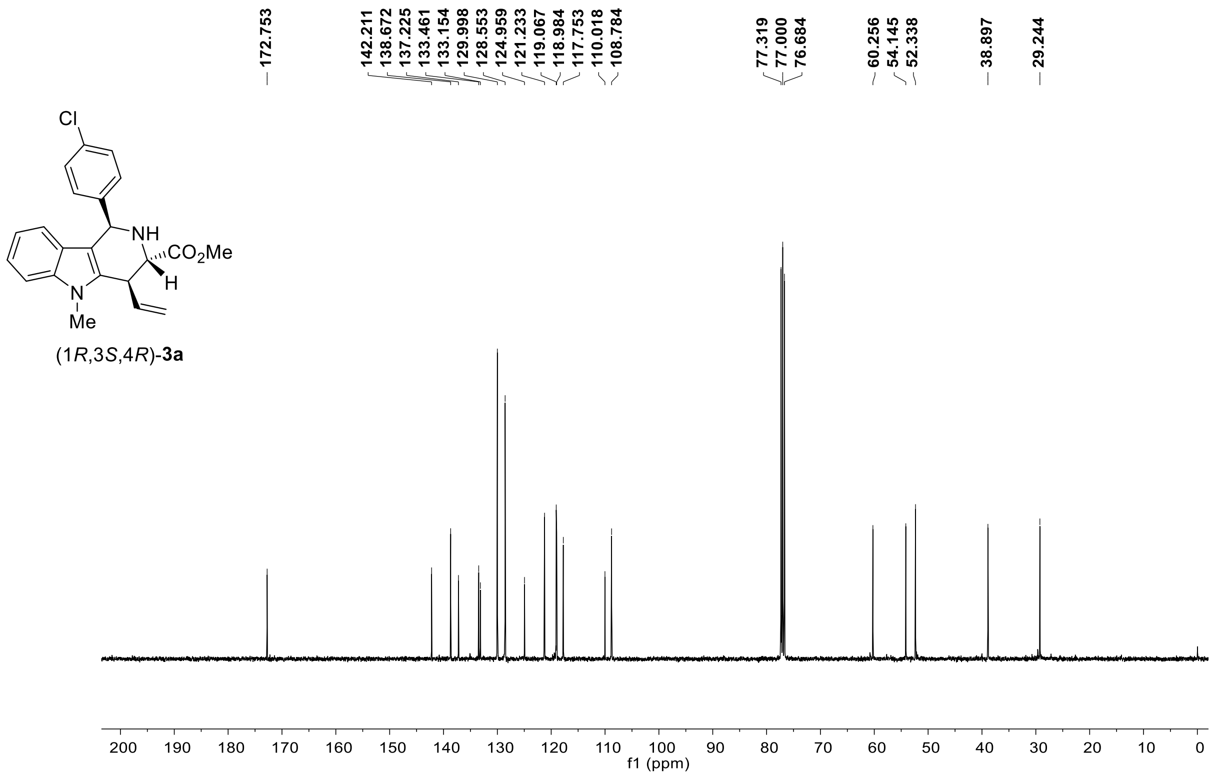

Supplementary Figure 28. ^{13}C NMR spectrum of (1*R*,3*S*,4*R*)-**3a**

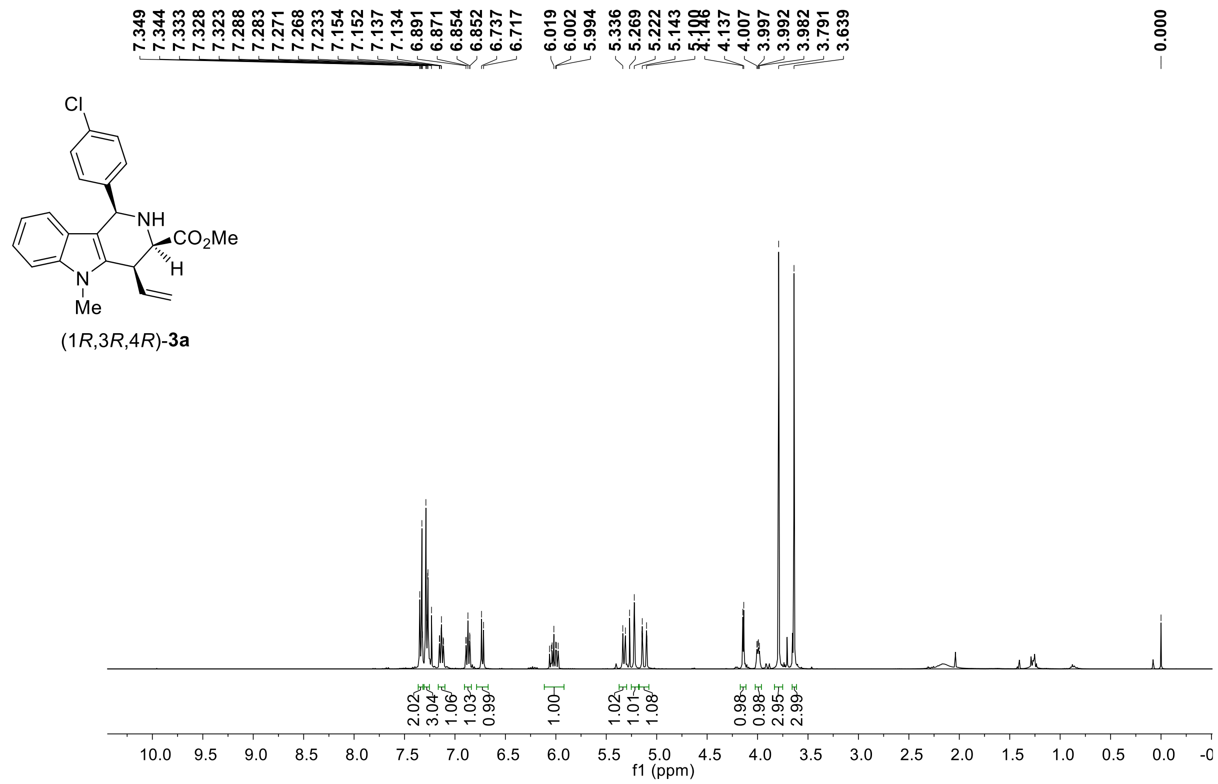

Supplementary Figure 29. ^{1}H NMR spectrum of (1*R*,3*R*,4*R*)-**3a**

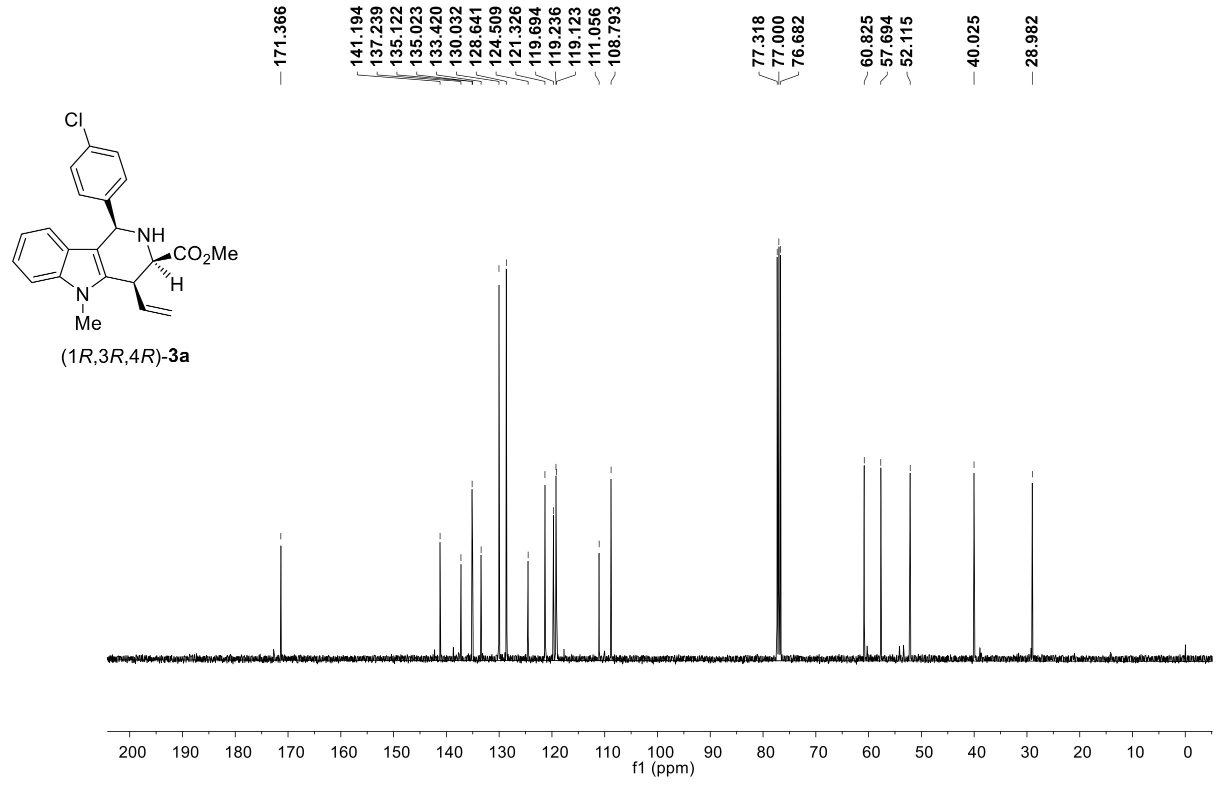

Supplementary Figure 30. ^{13}C NMR spectrum of (1*R*,3*R*,4*R*)-**3a**

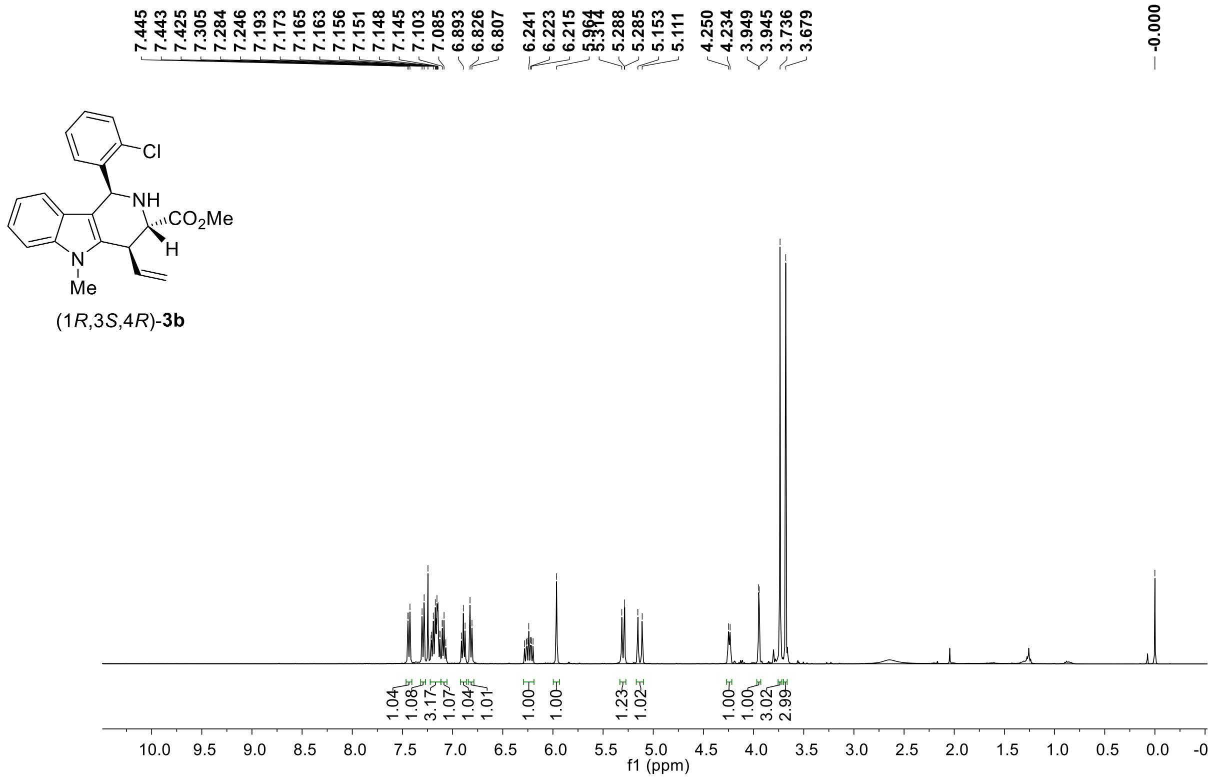

Supplementary Figure 31. ^{1}H NMR spectrum of (1*R*,3*S*,4*R*)-**3b**

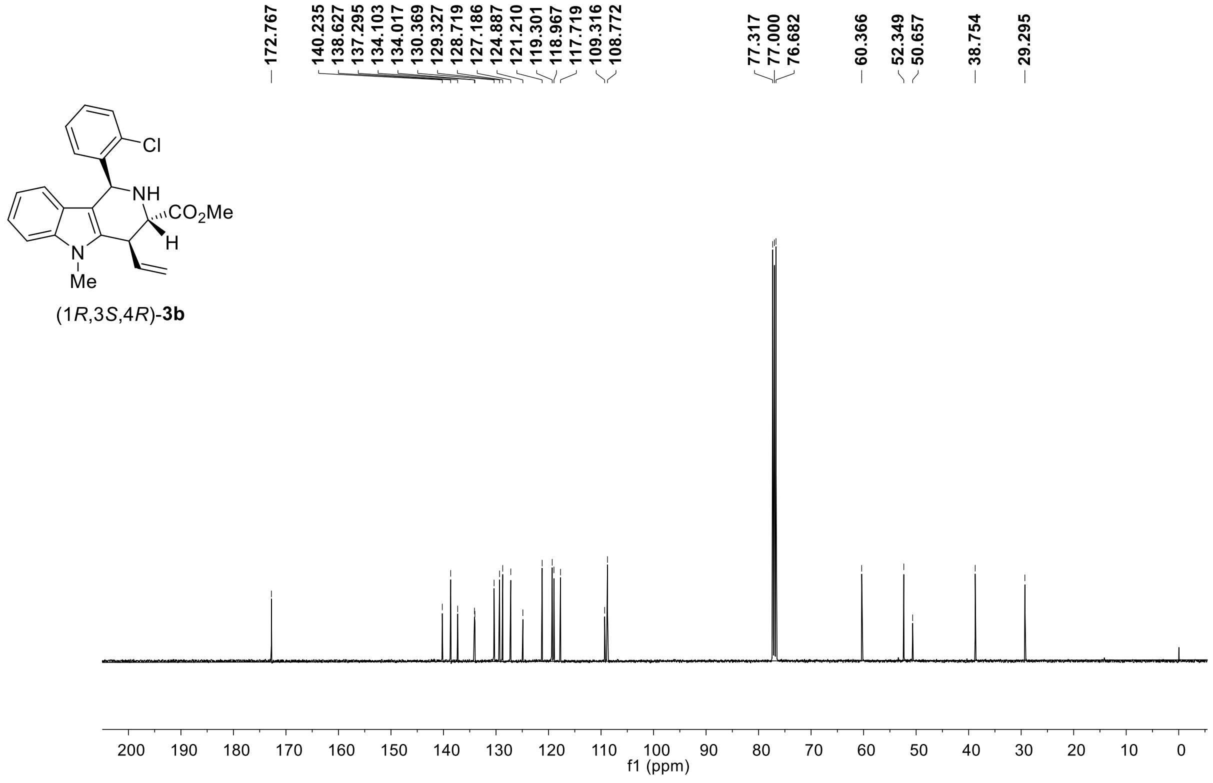

Supplementary Figure 32. ^{13}C NMR spectrum of (1*R*,3*S*,4*R*)-**3b**

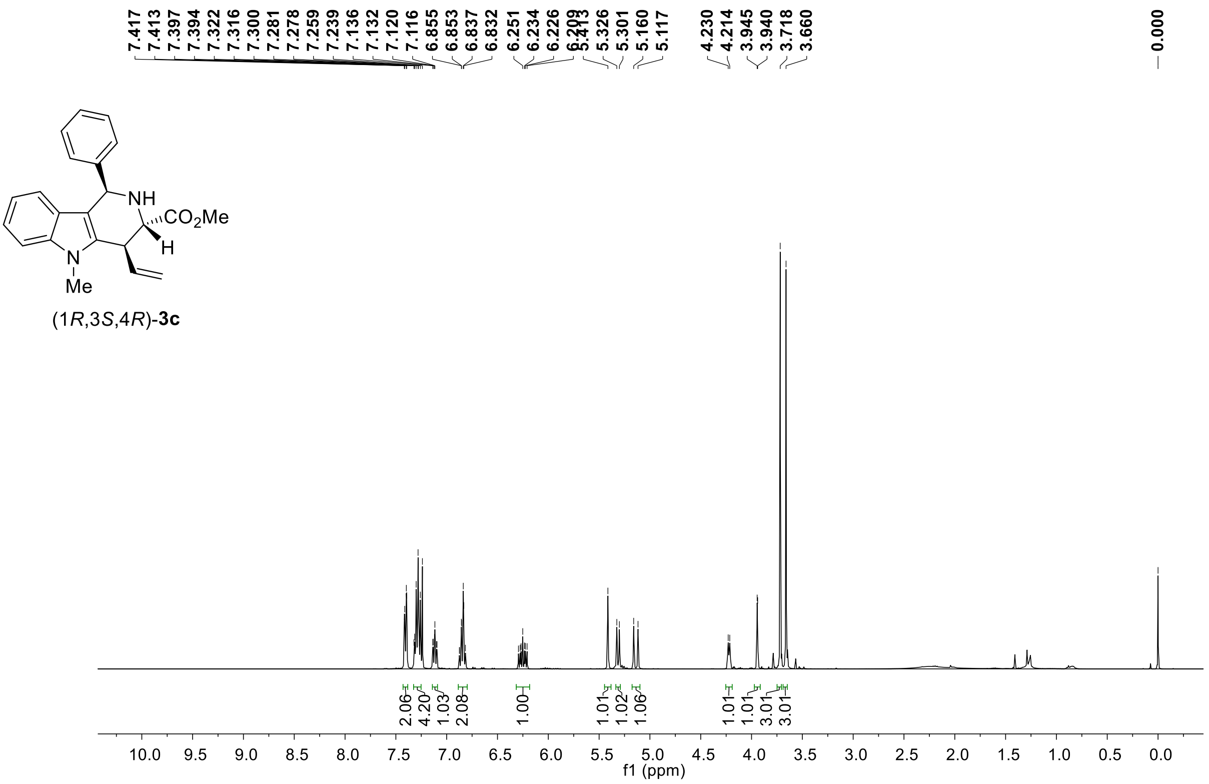

Supplementary Figure 33. ^{1}H NMR spectrum of (1*R*,3*S*,4*R*)-**3c**

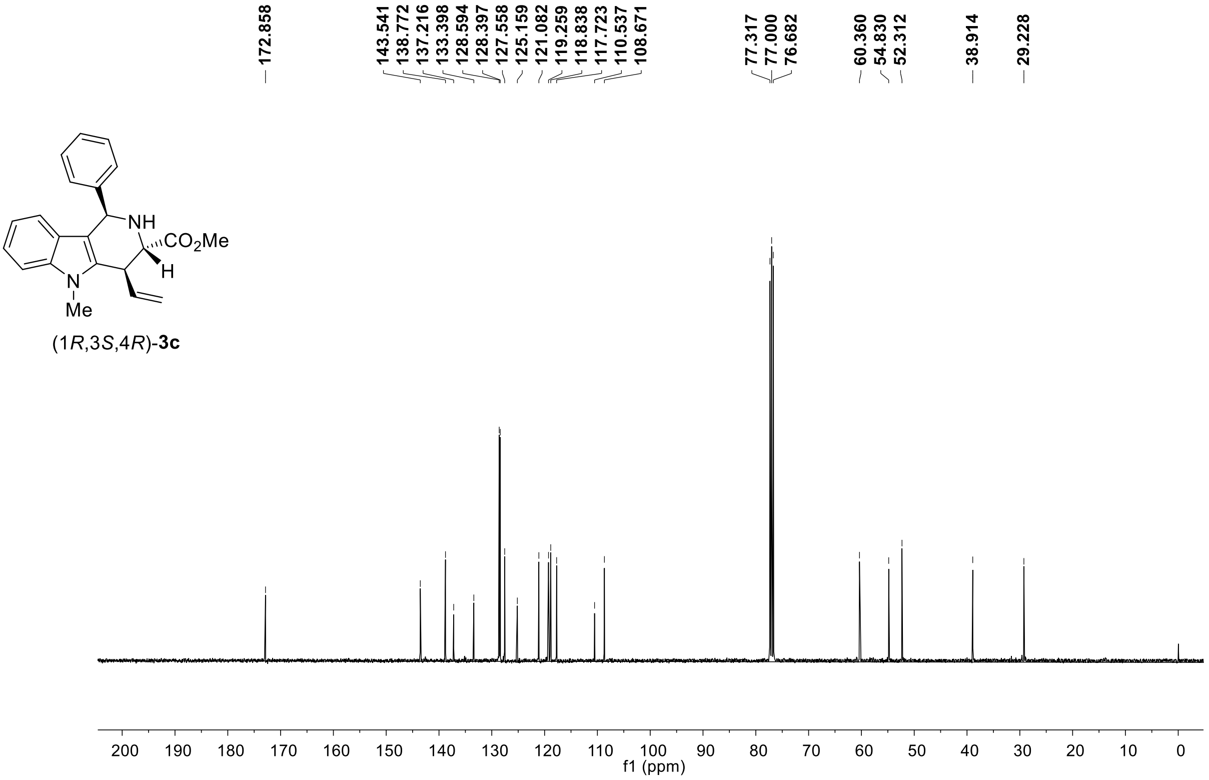

Supplementary Figure 34. ^{13}C NMR spectrum of (1*R*,3*S*,4*R*)-**3c**

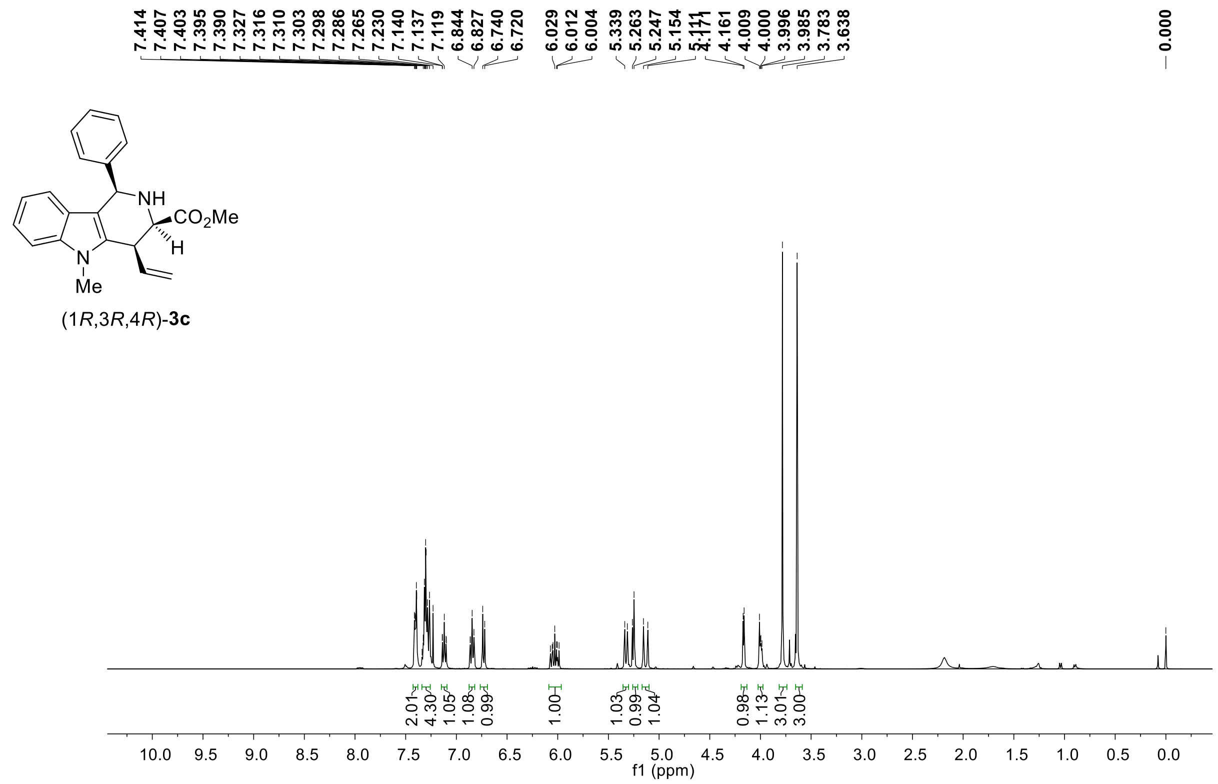

Supplementary Figure 35. ^{1}H NMR spectrum of (1*R*,3*R*,4*R*)-**3c**

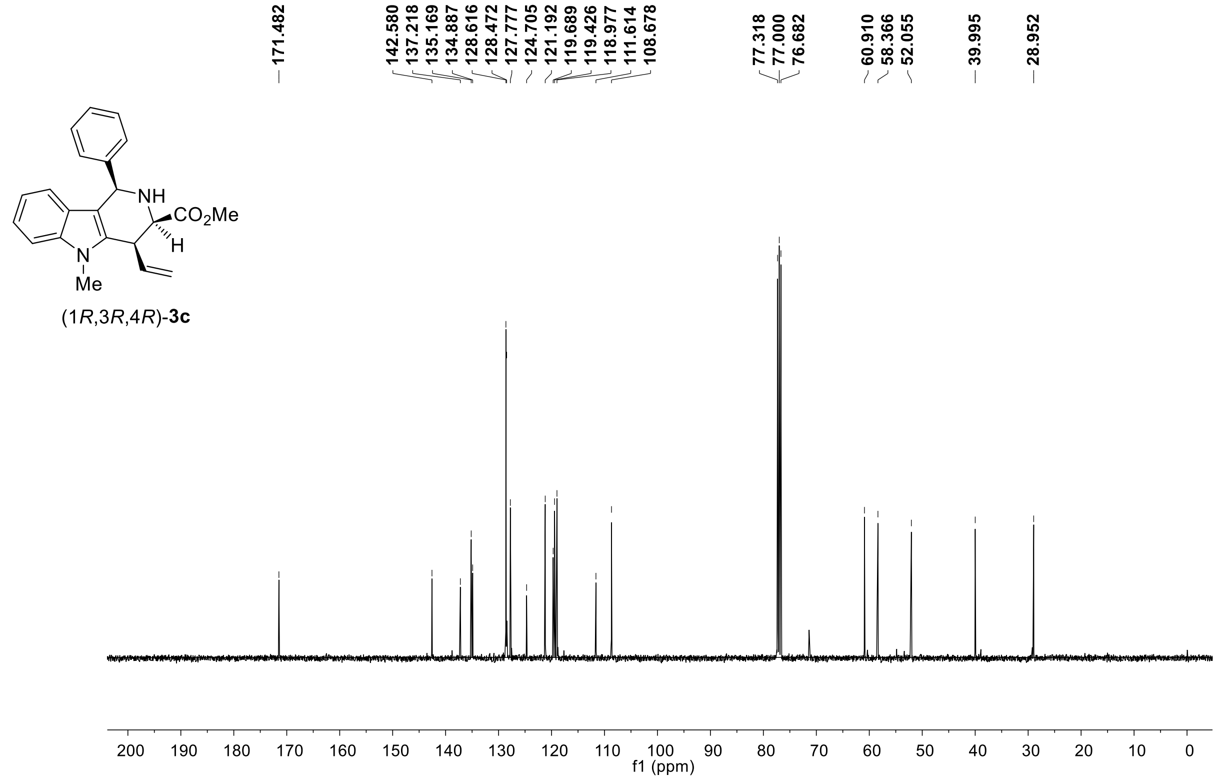

Supplementary Figure 36. ^{13}C NMR spectrum of (1*R*,3*R*,4*R*)-**3c**

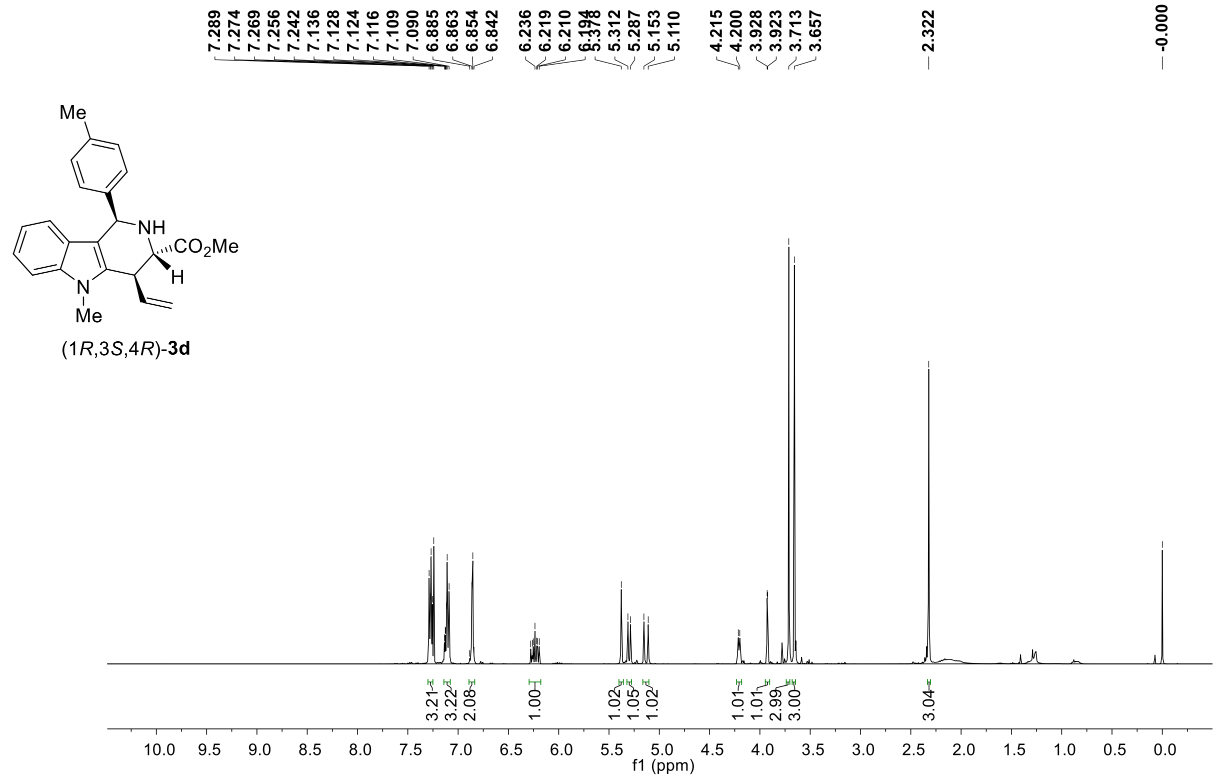

Supplementary Figure 37. ^{1}H NMR spectrum of (1*R*,3*S*,4*R*)-**3d**

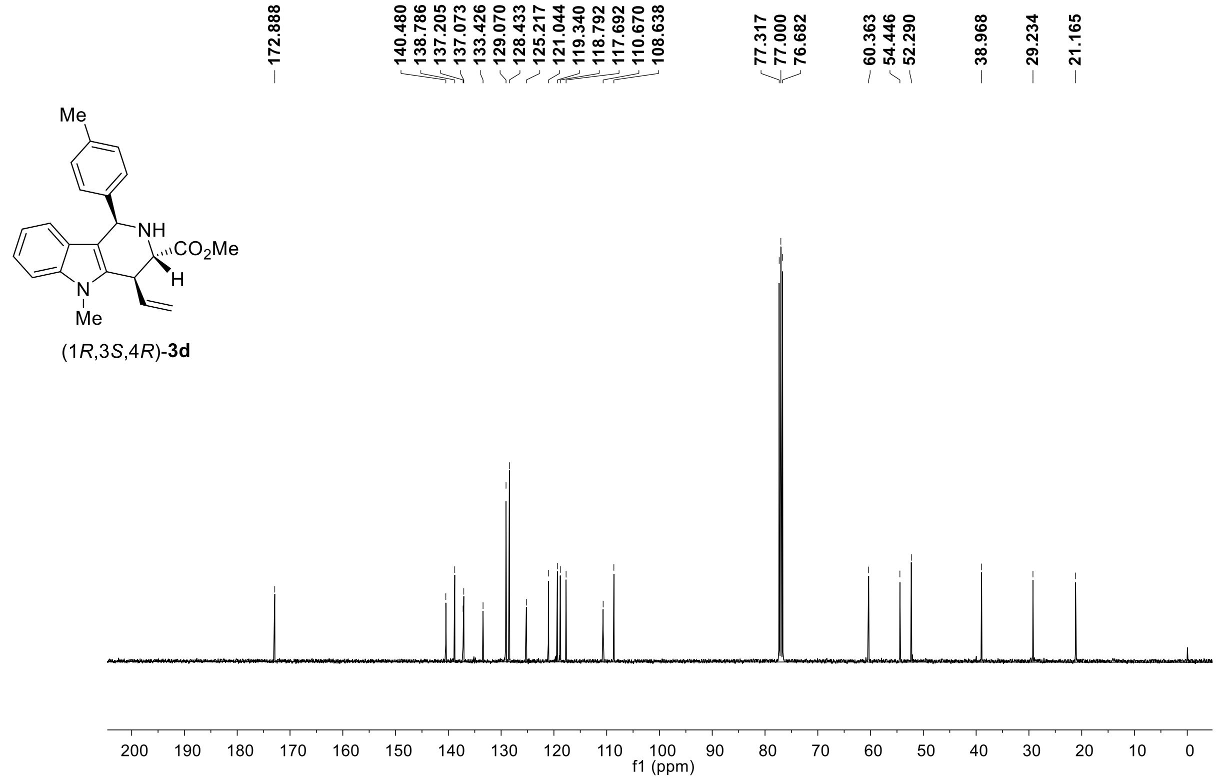

Supplementary Figure 38. ^{13}C NMR spectrum of (1*R*,3*S*,4*R*)-**3d**

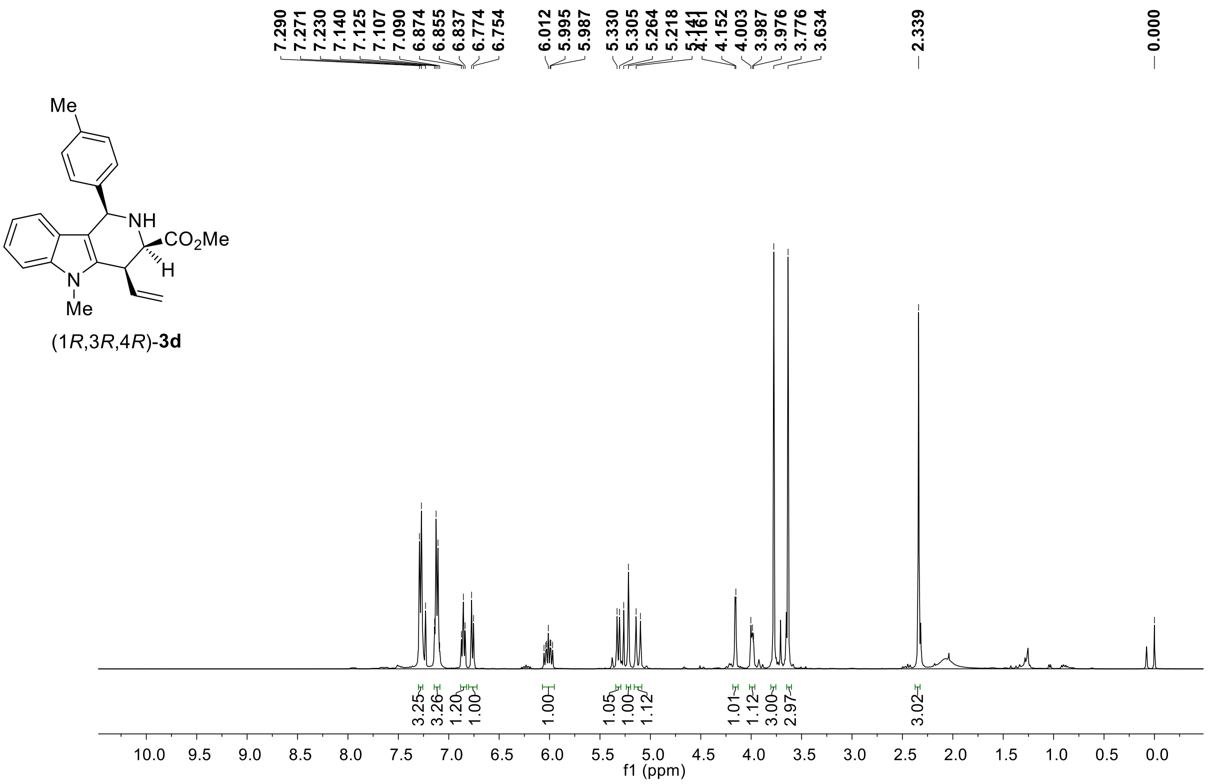

Supplementary Figure 39. ^{1}H NMR spectrum of (1*R*,3*R*,4*R*)-**3d**

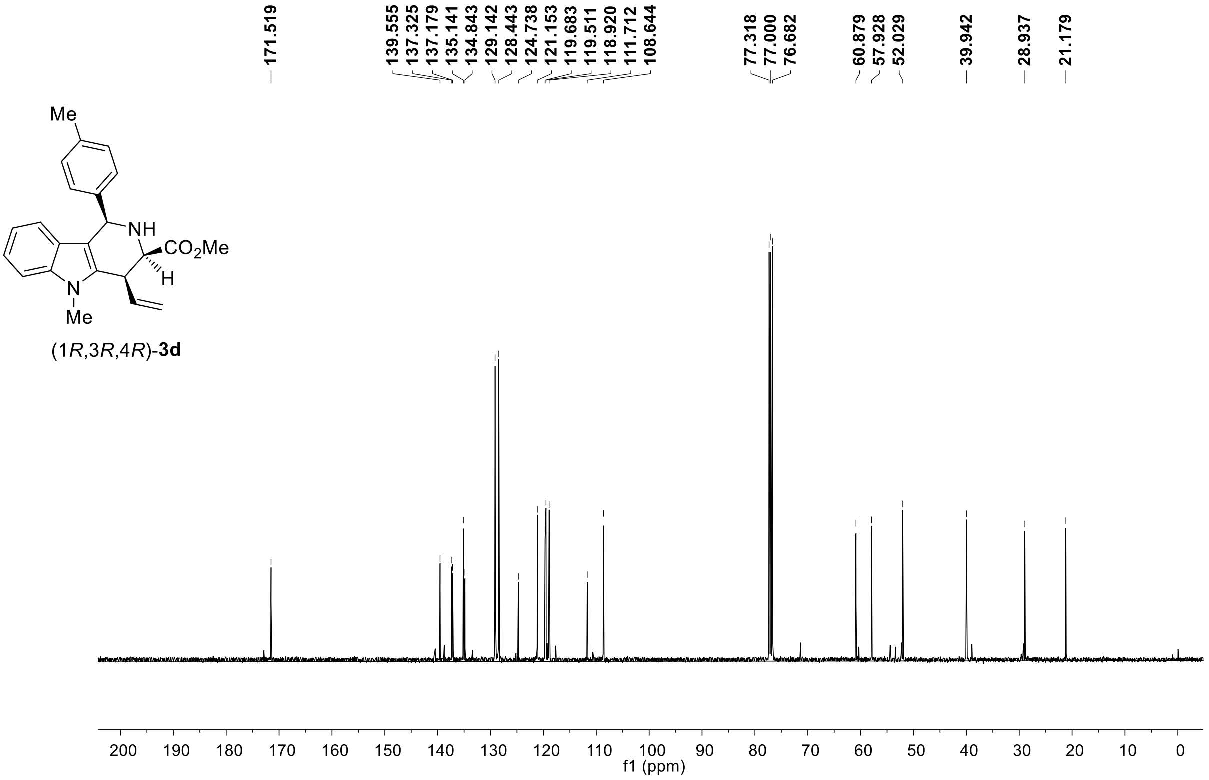

Supplementary Figure 40. ^{13}C NMR spectrum of (1*R*,3*R*,4*R*)-**3d**

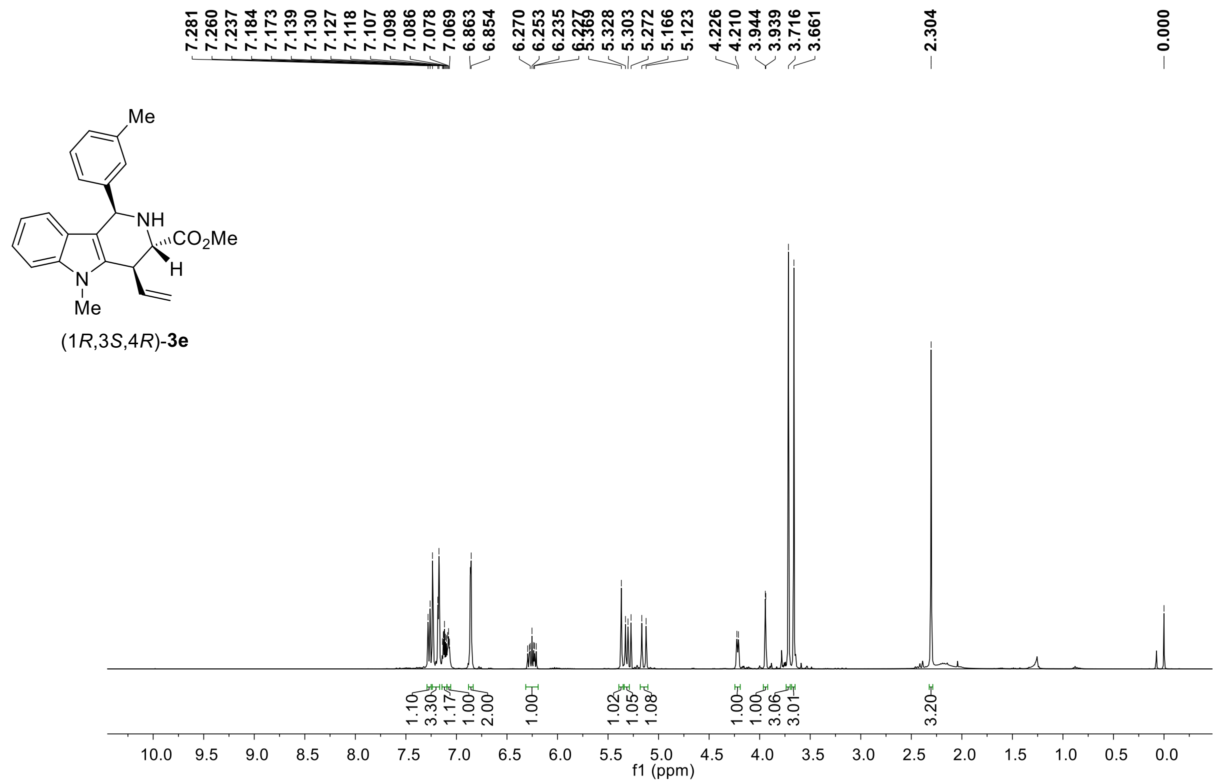

Supplementary Figure 41. ^{1}H NMR spectrum of (1*R*,3*S*,4*R*)-**3e**

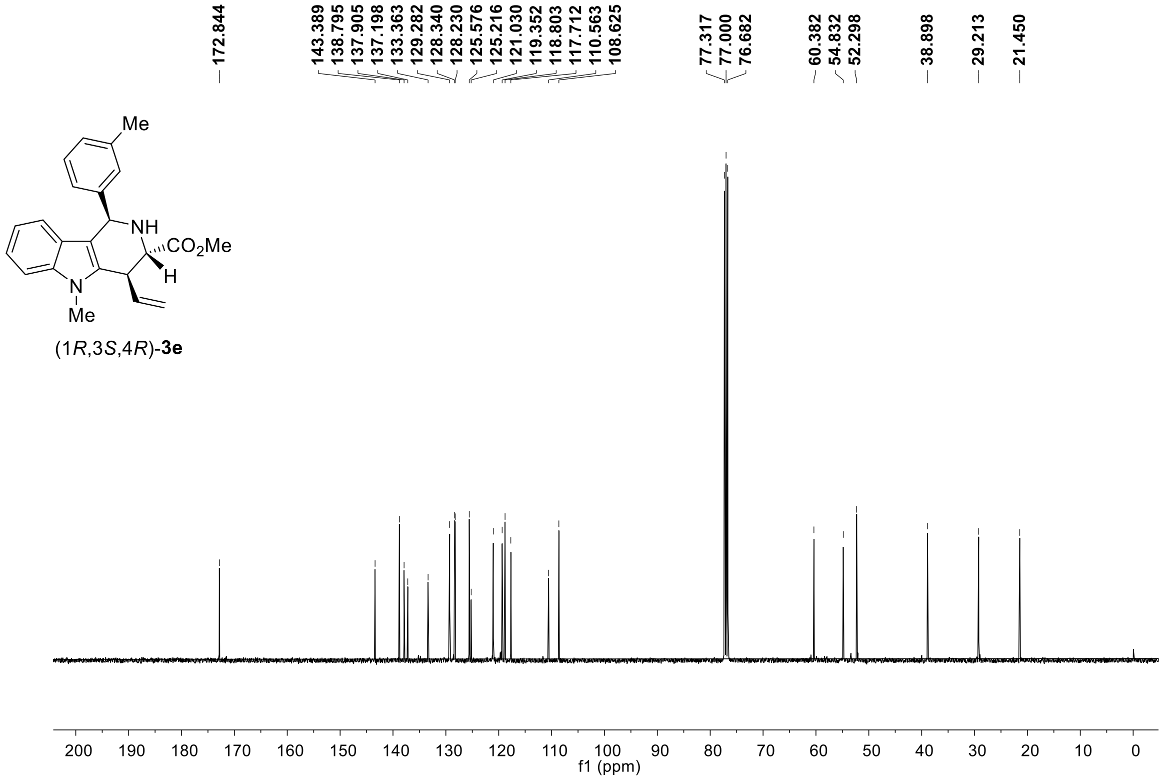

Supplementary Figure 42. ^{13}C NMR spectrum of (1*R*,3*S*,4*R*)-**3e**

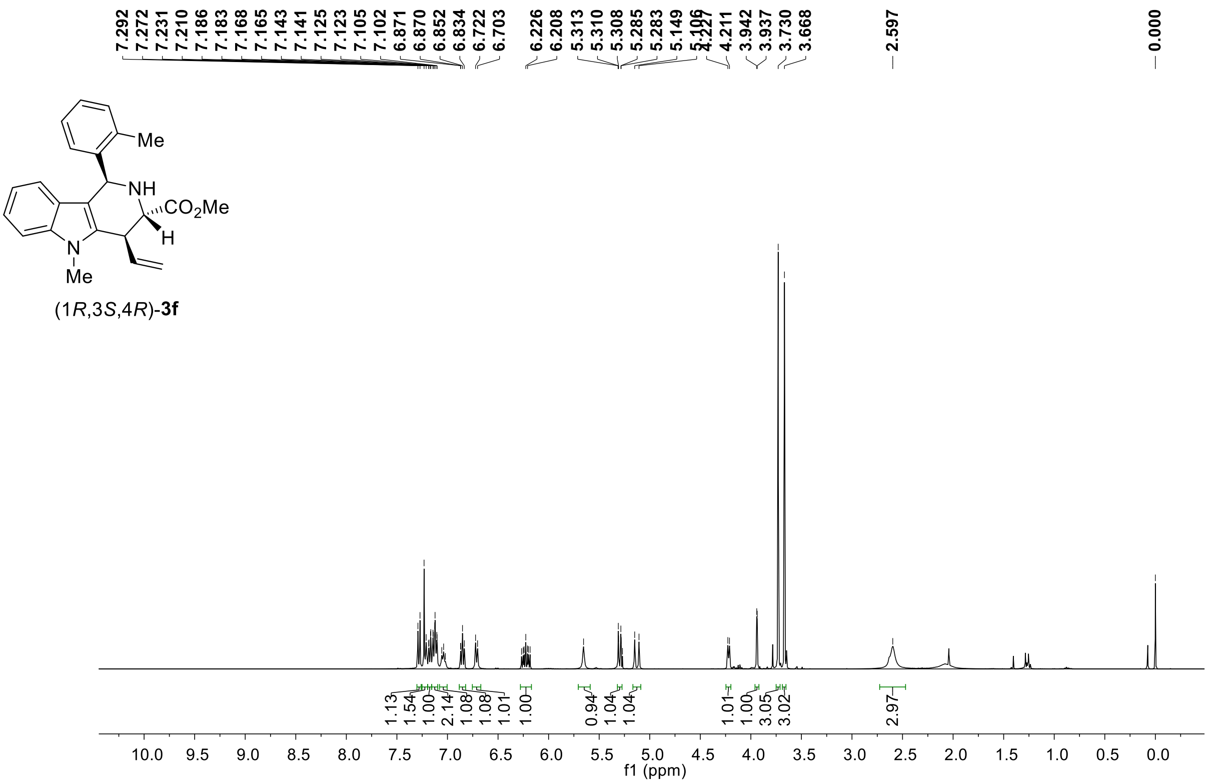

Supplementary Figure 43. ^{1}H NMR spectrum of (1*R*,3*S*,4*R*)-**3f**

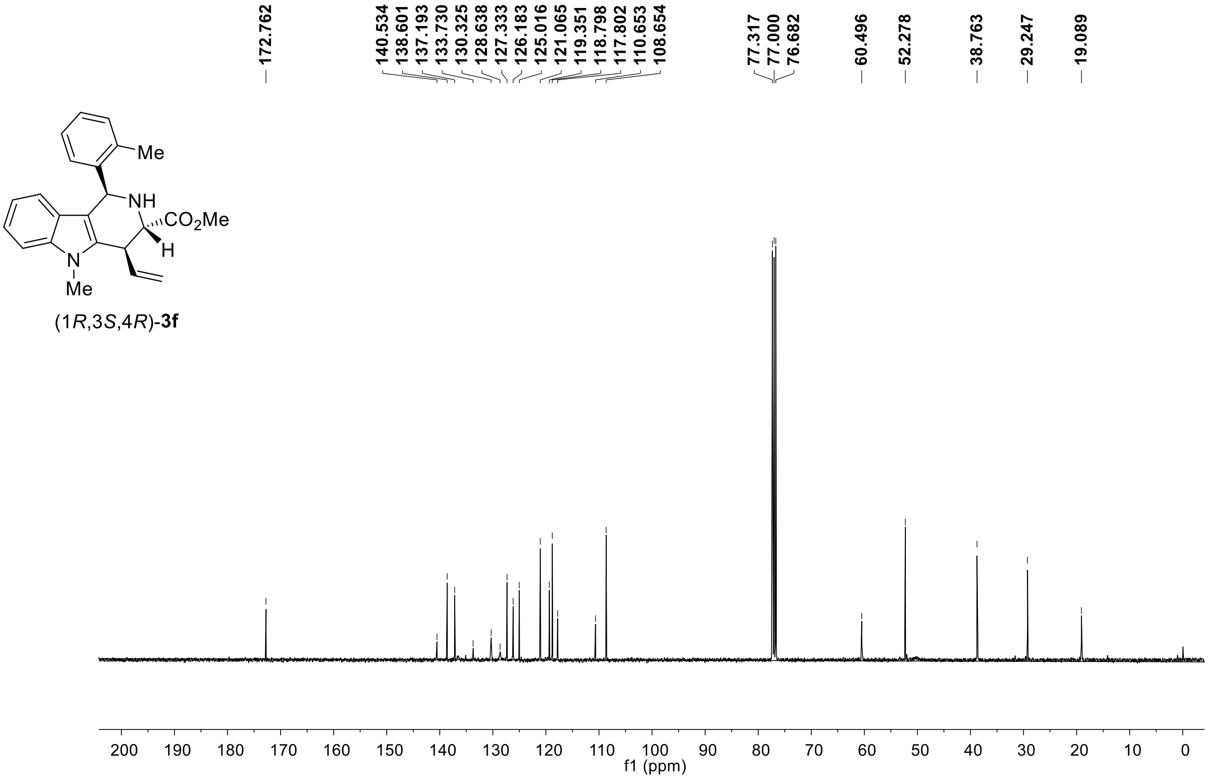

Supplementary Figure 44. ^{13}C NMR spectrum of (1*R*,3*S*,4*R*)-**3f**

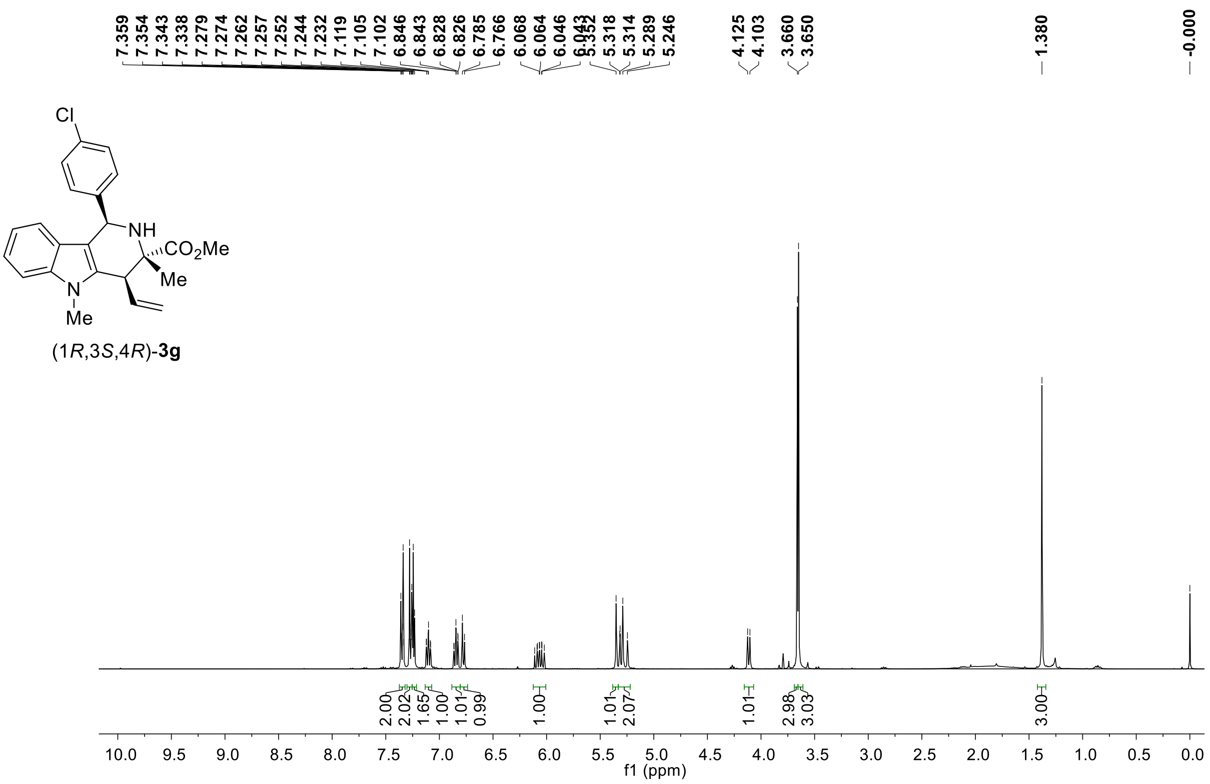

Supplementary Figure 45. ^{1}H NMR spectrum of (1*R*,3*S*,4*R*)-**3g**

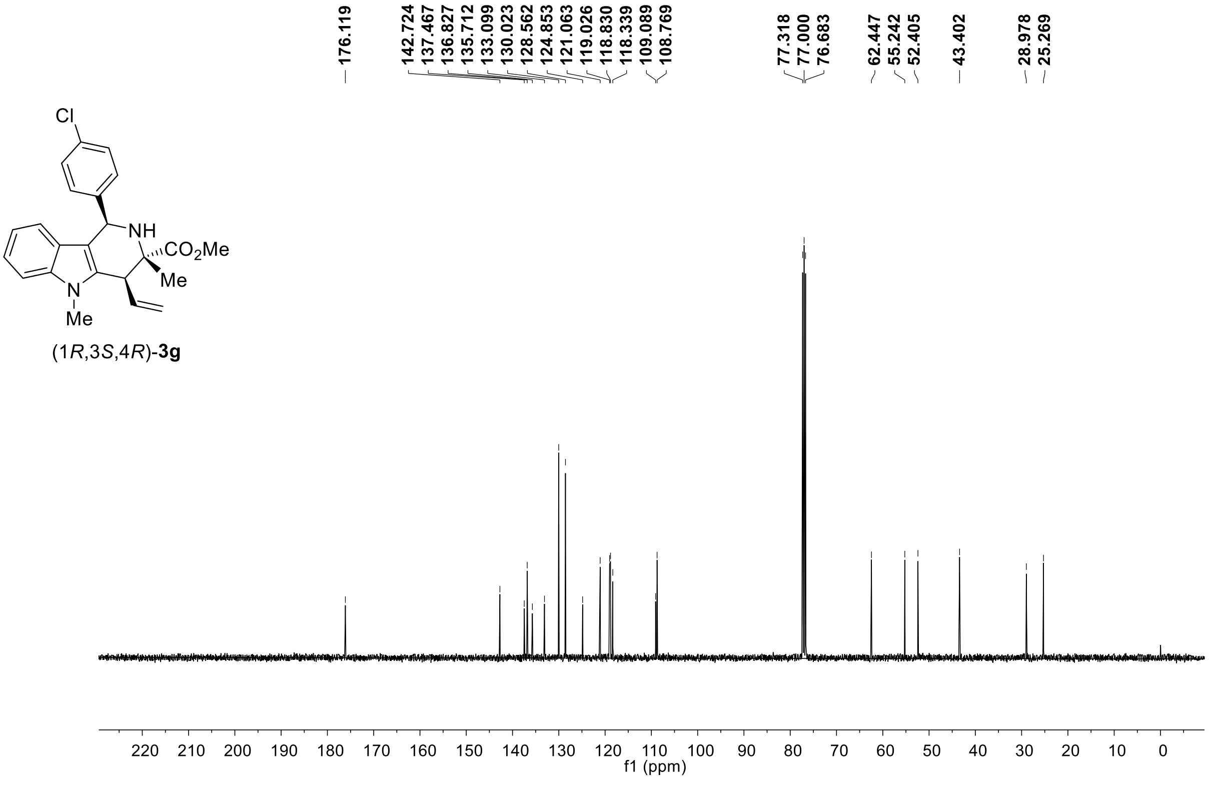

Supplementary Figure 46. ^{13}C NMR spectrum of (1*R*,3*S*,4*R*)-**3g**

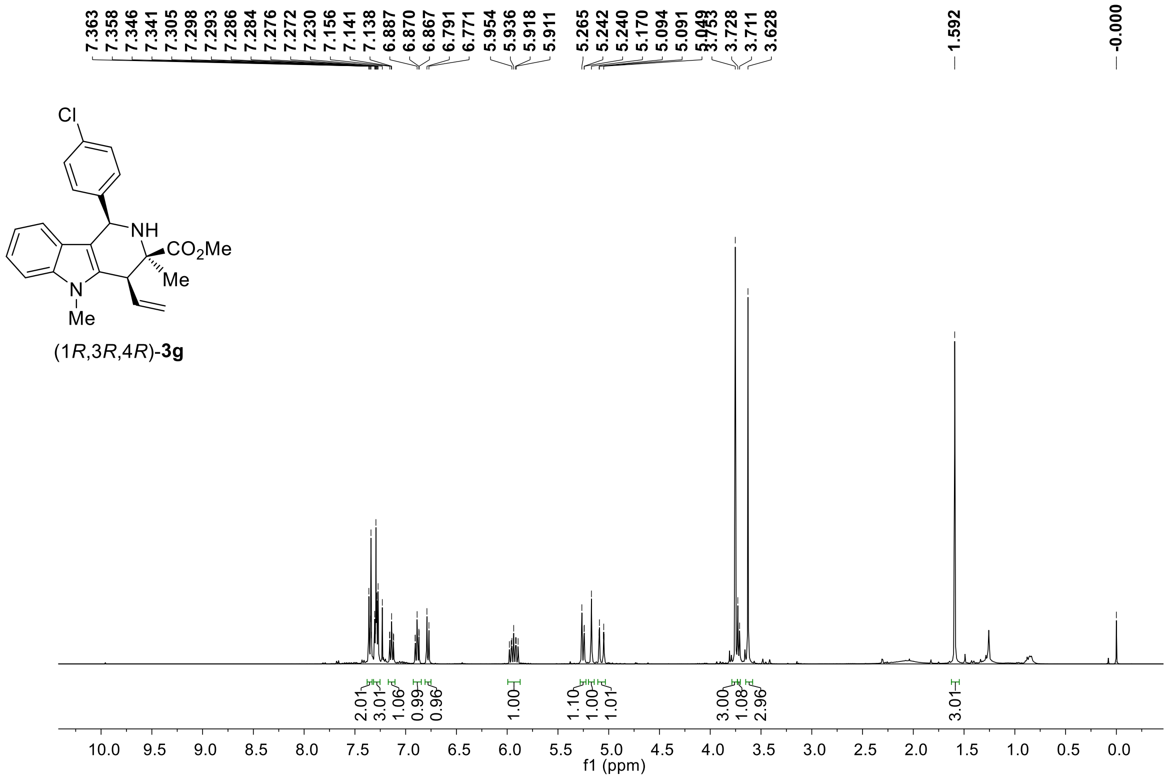

Supplementary Figure 47. ^{1}H NMR spectrum of (1*R*,3*R*,4*R*)-**3g**

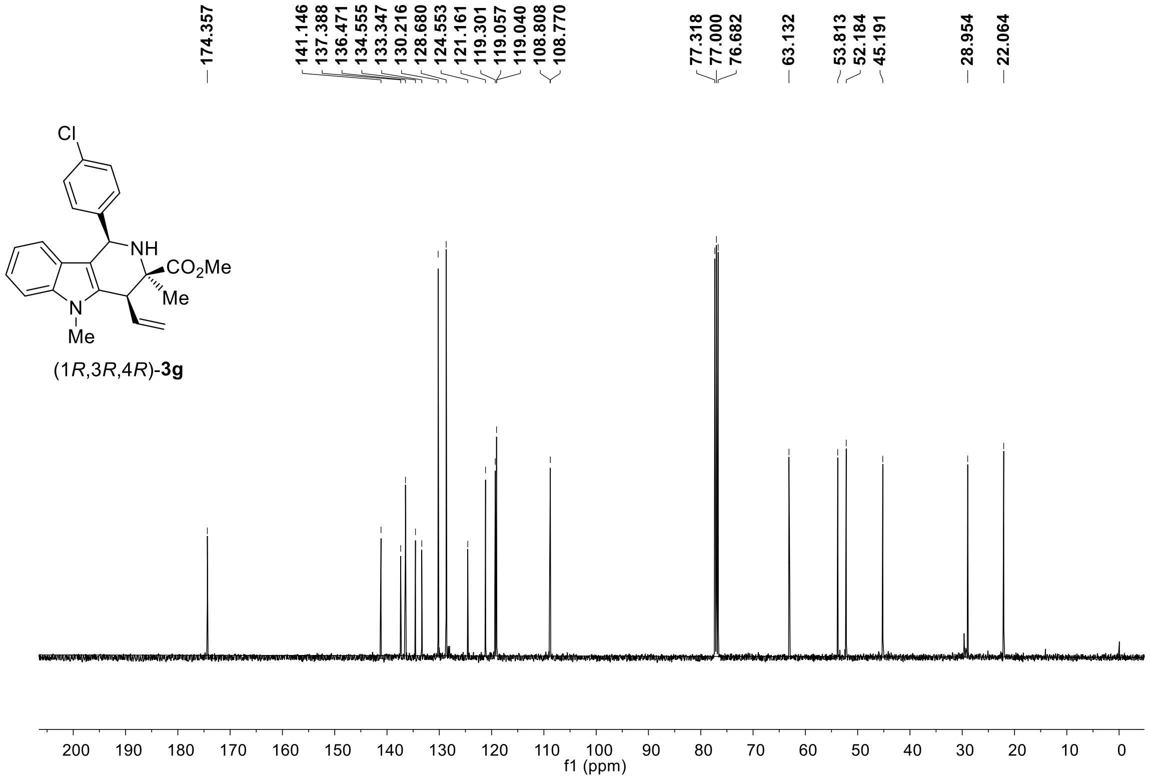

Supplementary Figure 48. ^{13}C NMR spectrum of (1*R*,3*R*,4*R*)-**3g**

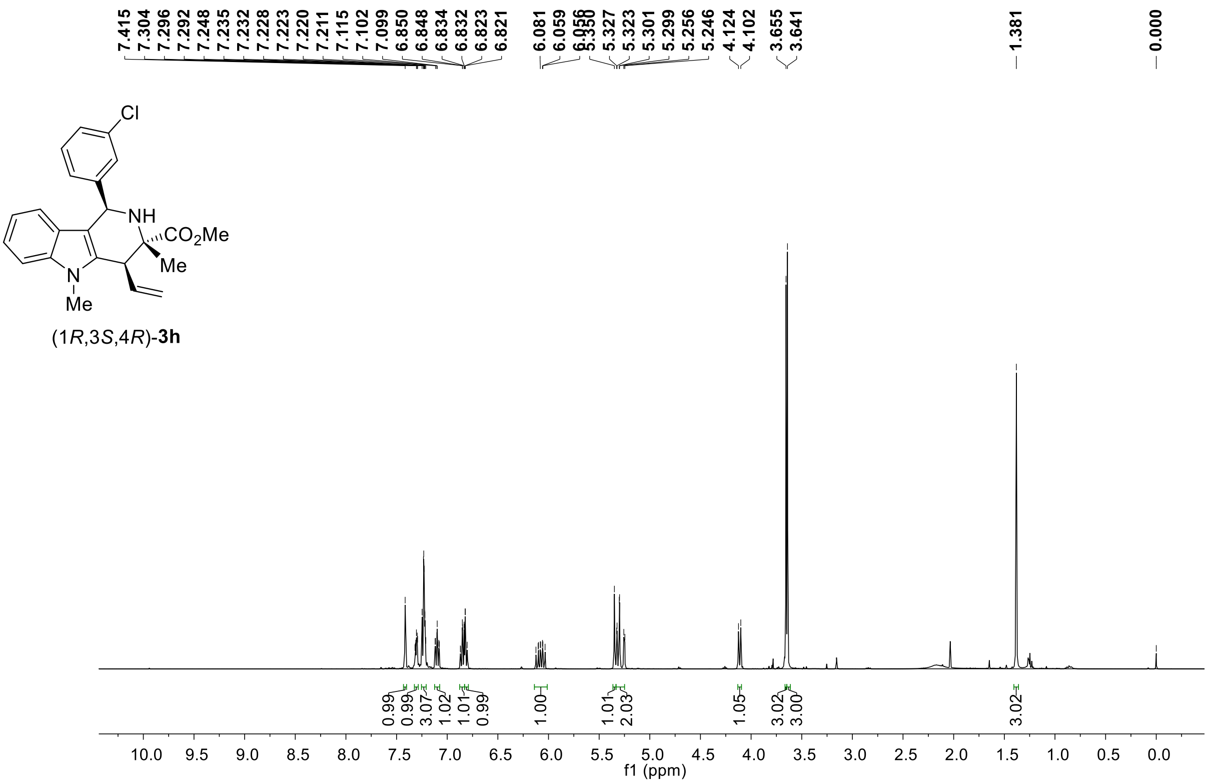

Supplementary Figure 49. ^{1}H NMR spectrum of (1*R*,3*S*,4*R*)-**3h**

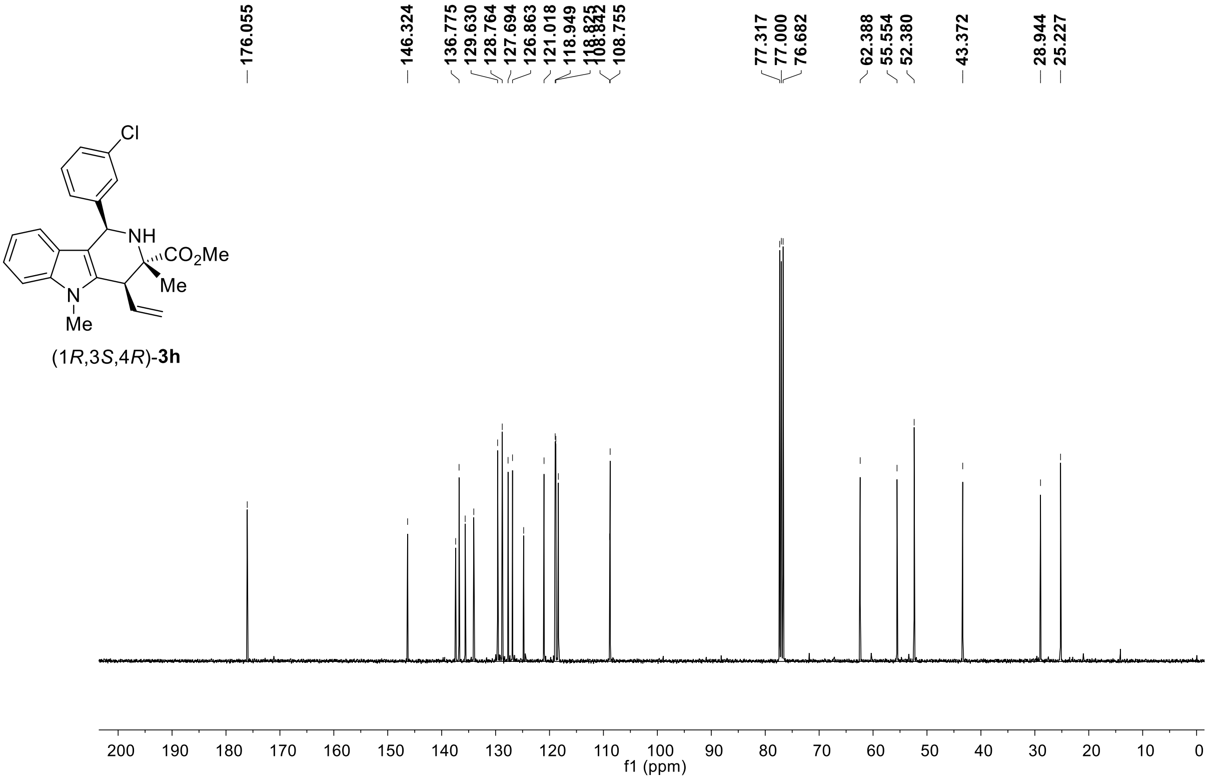

Supplementary Figure 50. ^{13}C NMR spectrum of (1*R*,3*S*,4*R*)-**3h**

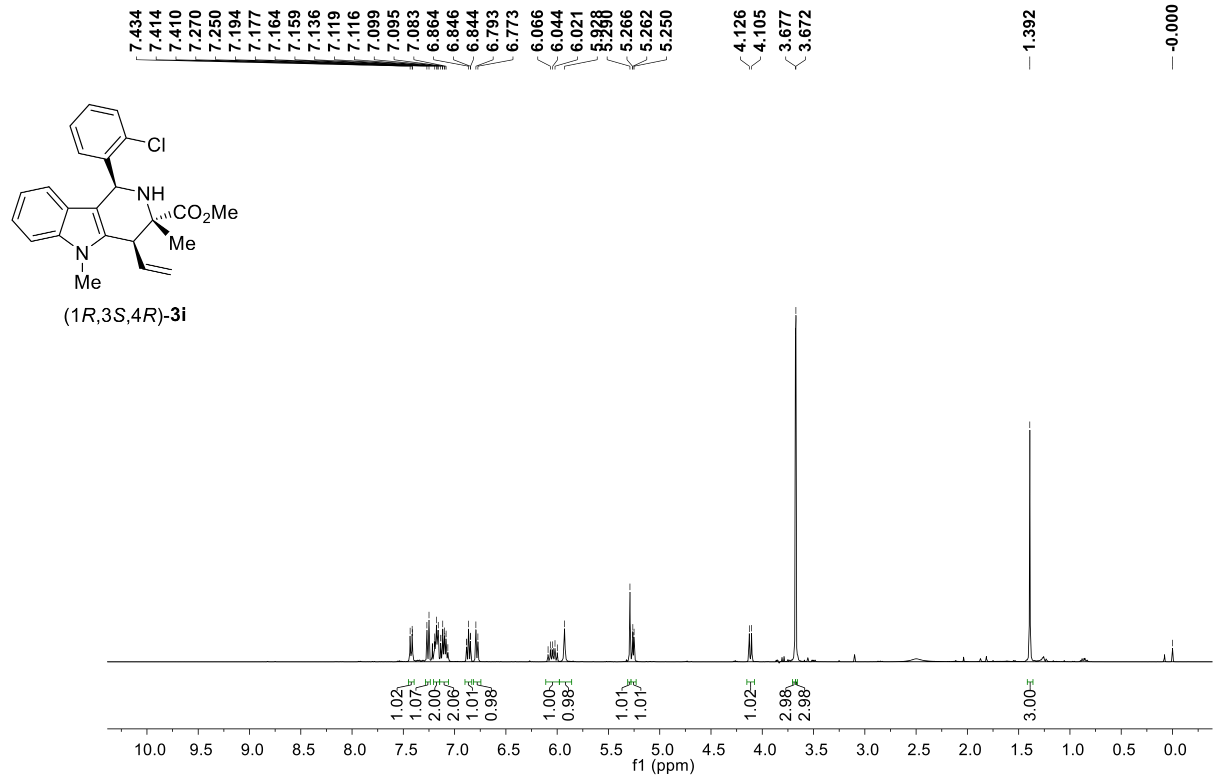

Supplementary Figure 51. ^{1}H NMR spectrum of (1*R*,3*S*,4*R*)-**3i**

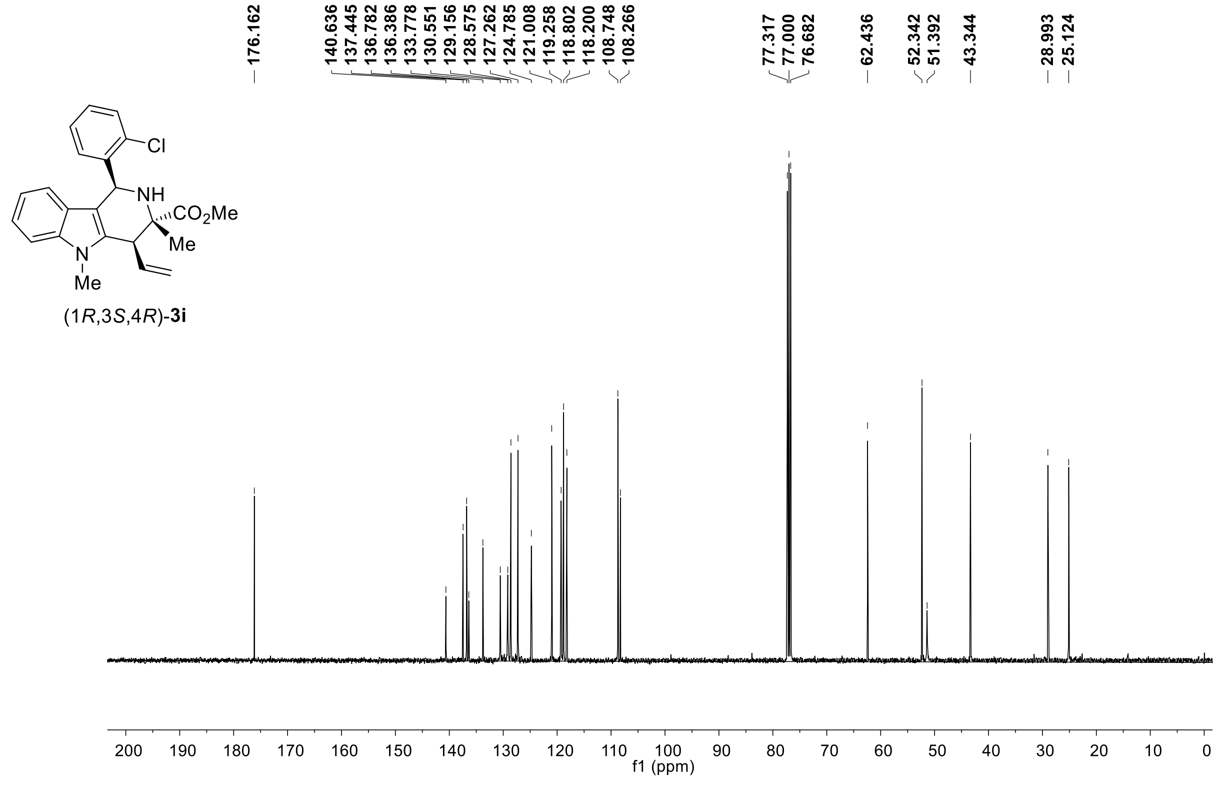

Supplementary Figure 52. ^{13}C NMR spectrum of (1*R*,3*S*,4*R*)-**3i**

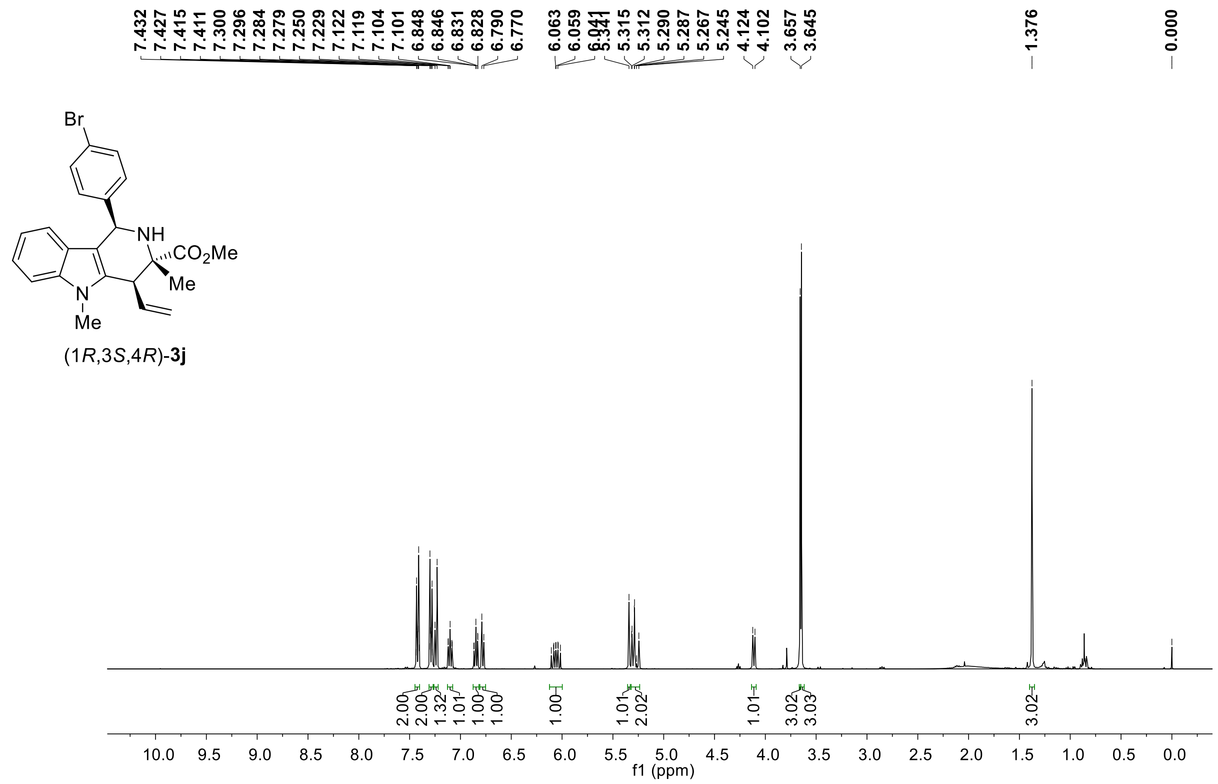

Supplementary Figure 53. ^{1}H NMR spectrum of (1*R*,3*S*,4*R*)-**3j**

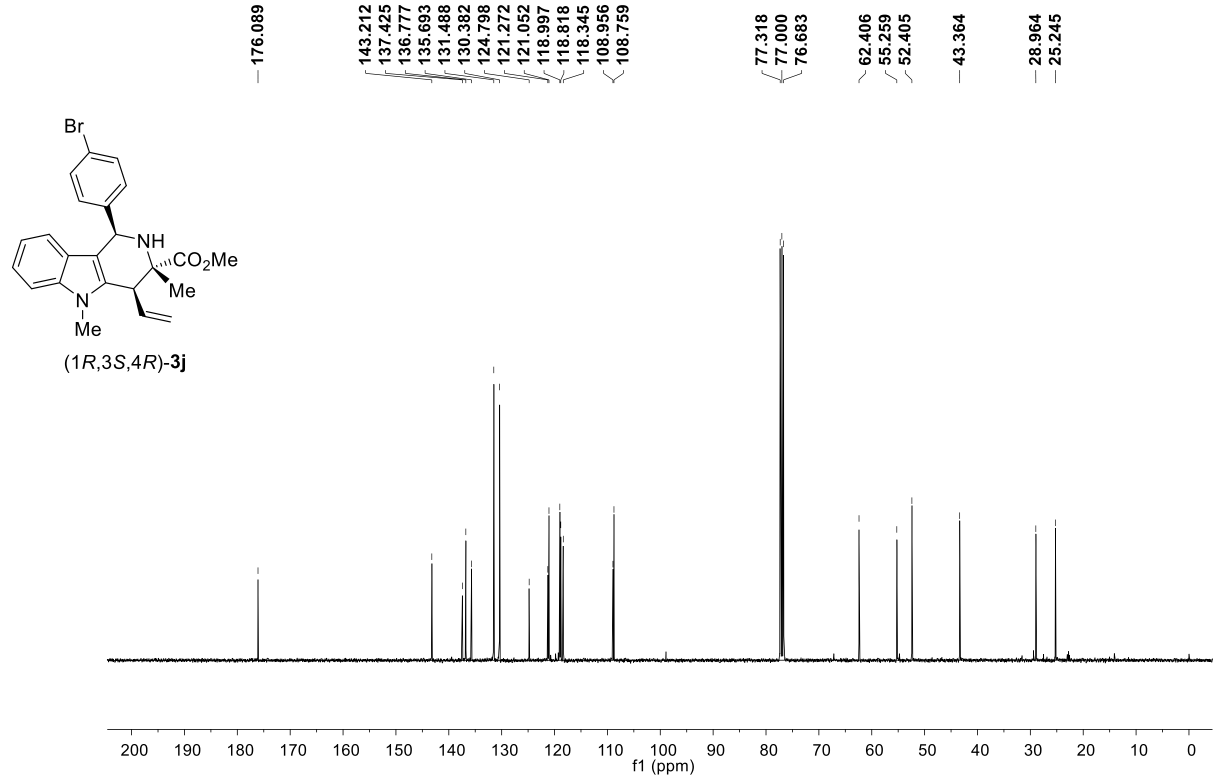

Supplementary Figure 54. ^{13}C NMR spectrum of (1*R*,3*S*,4*R*)-**3j**

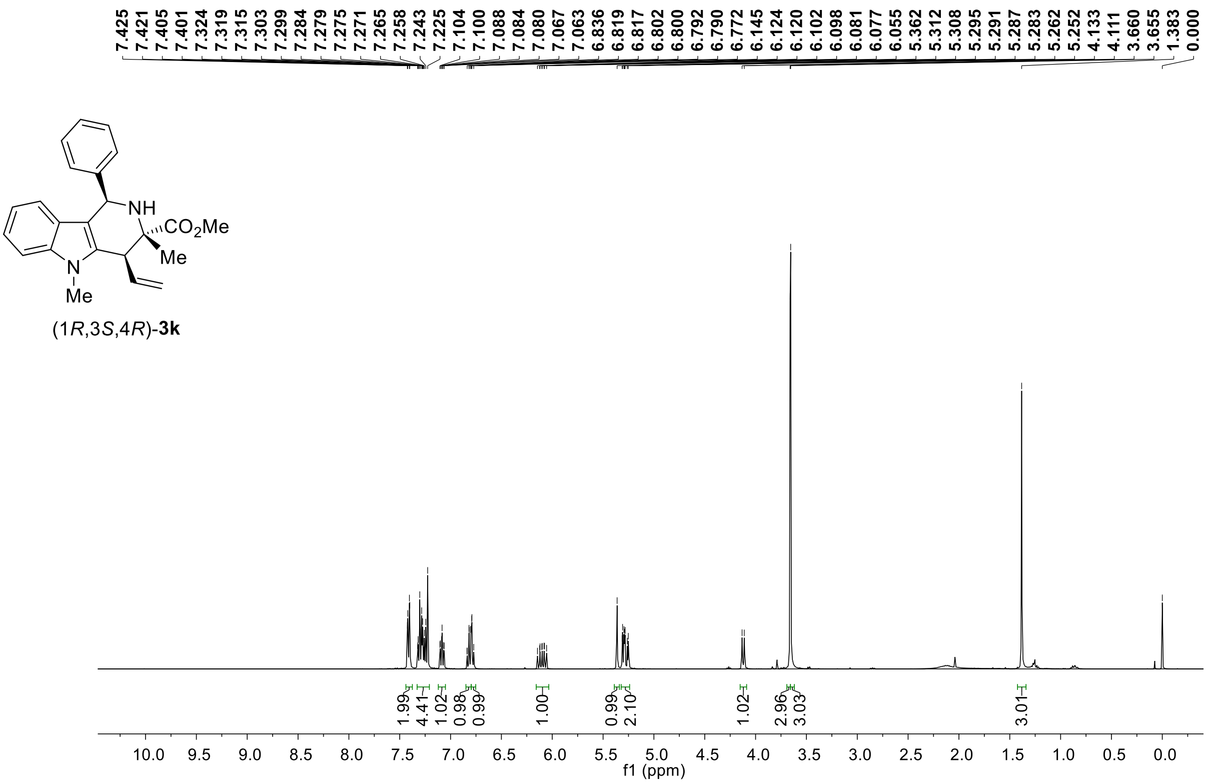

Supplementary Figure 55. ^{1}H NMR spectrum of (1*R*,3*S*,4*R*)-**3k**

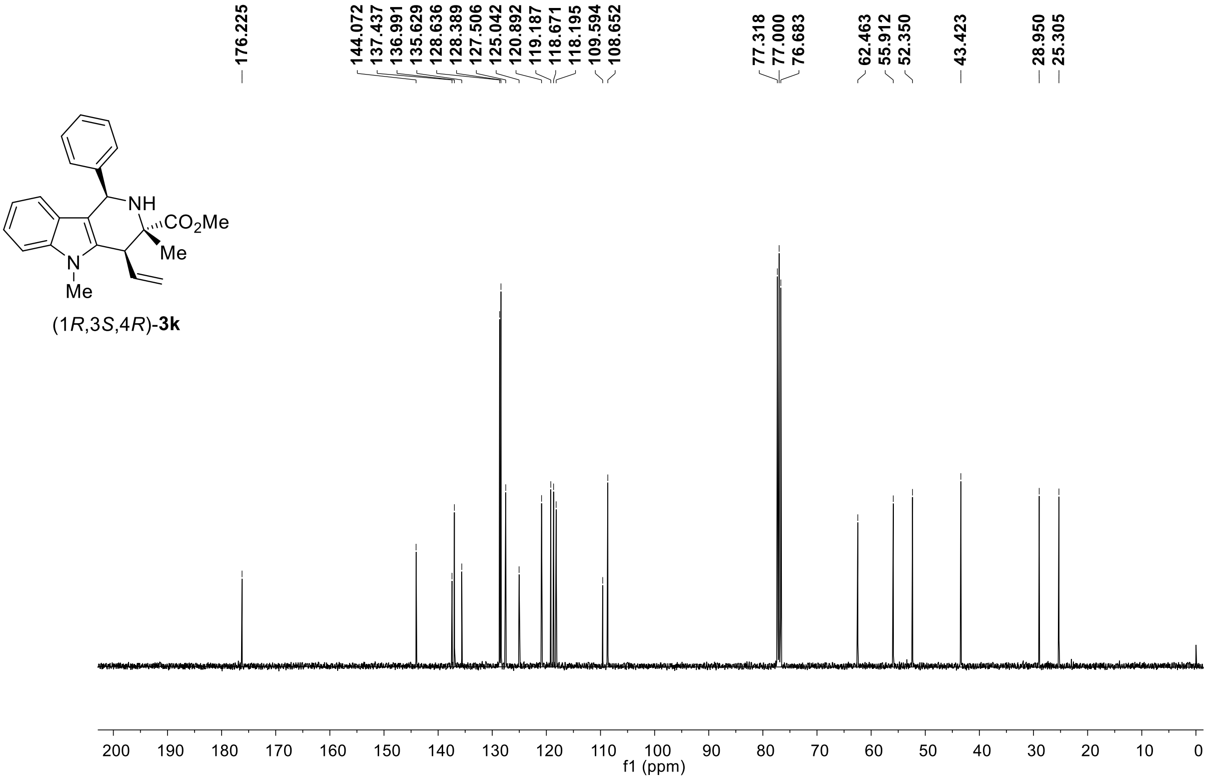

Supplementary Figure 56. ^{13}C NMR spectrum of (1*R*,3*S*,4*R*)-**3k**

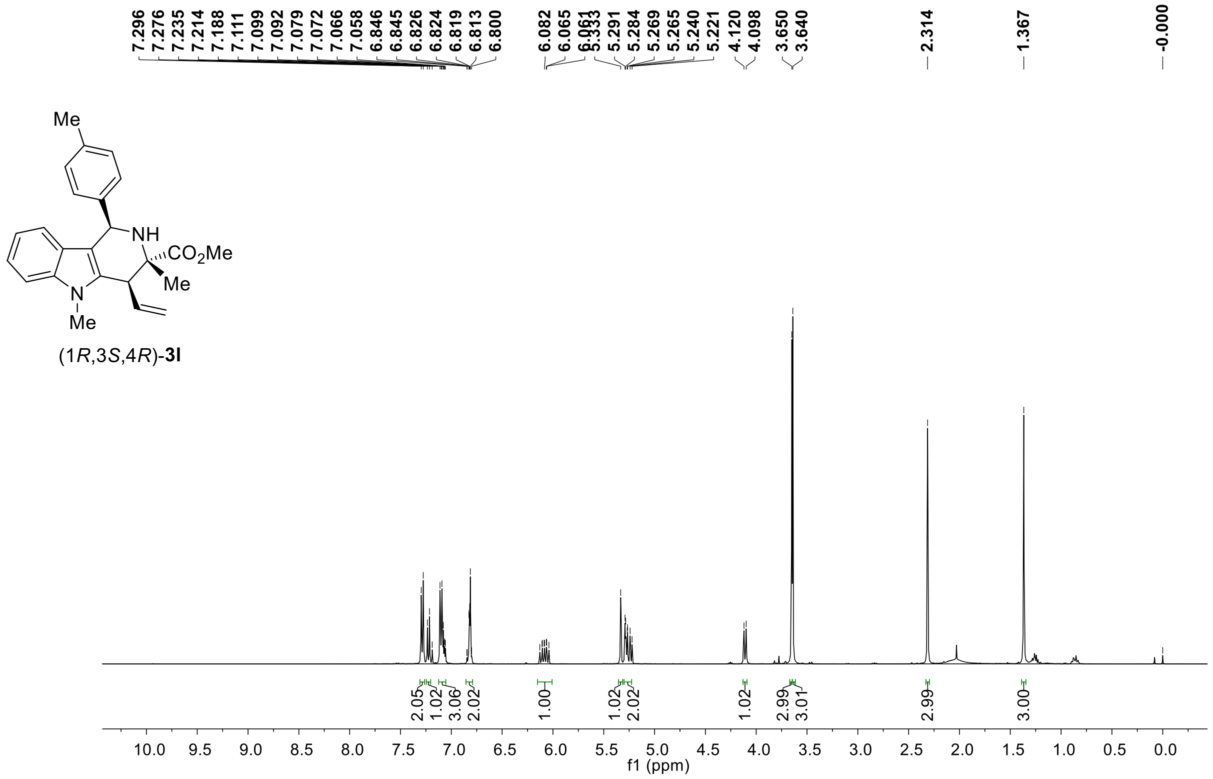

Supplementary Figure 57. ^{1}H NMR spectrum of (1*R*,3*S*,4*R*)-**3l**

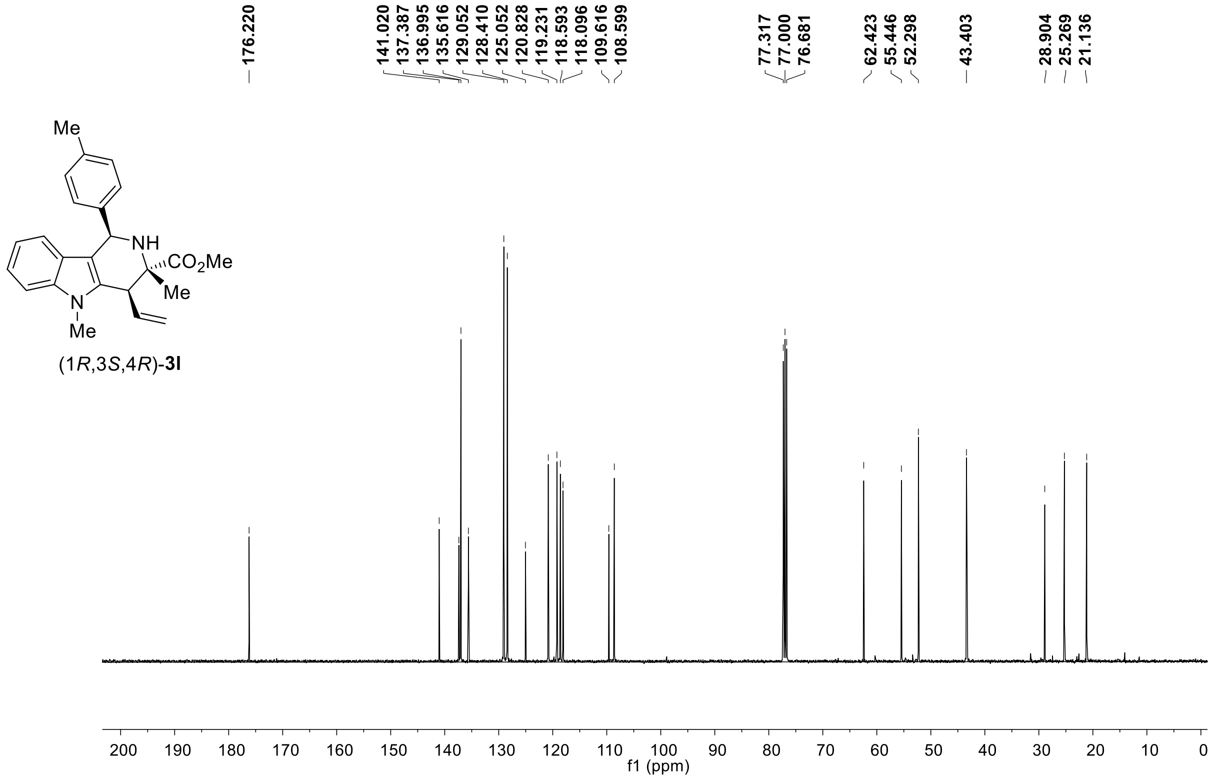

Supplementary Figure 58. ^{13}C NMR spectrum of (1*R*,3*S*,4*R*)-**3l**

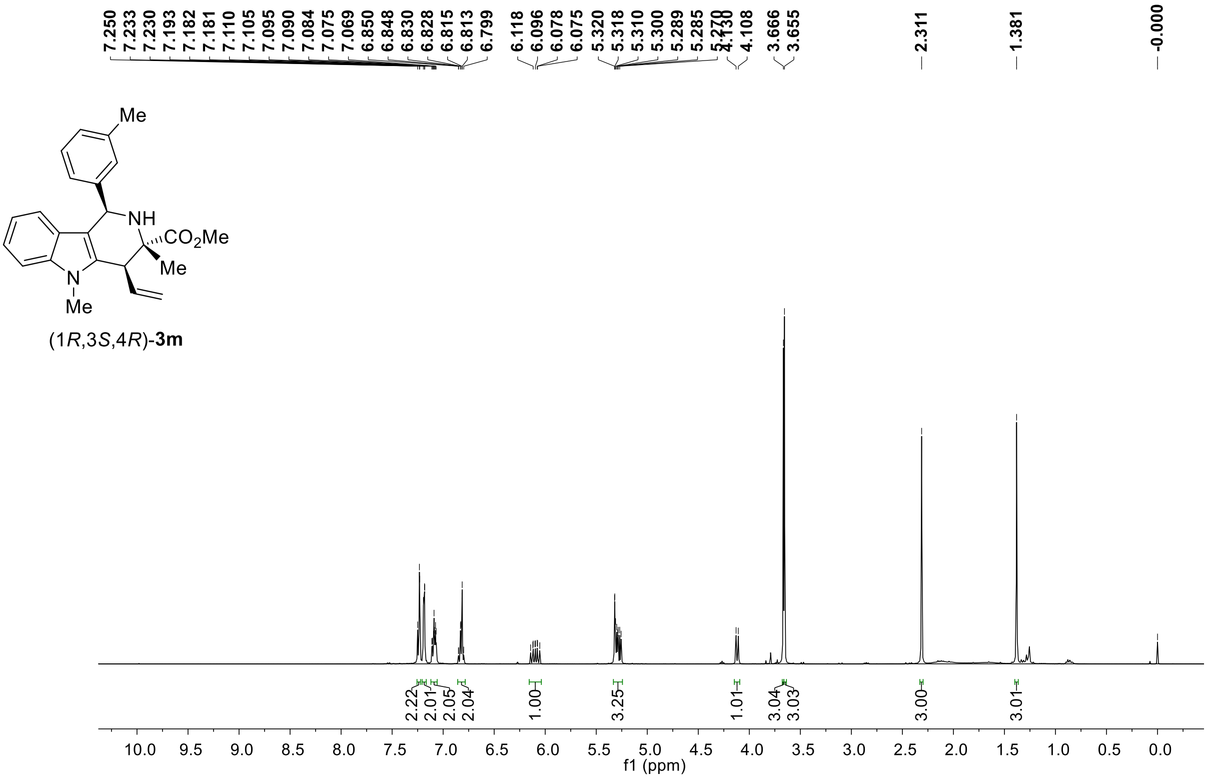

Supplementary Figure 59. ^{1}H NMR spectrum of (1*R*,3*S*,4*R*)-**3m**

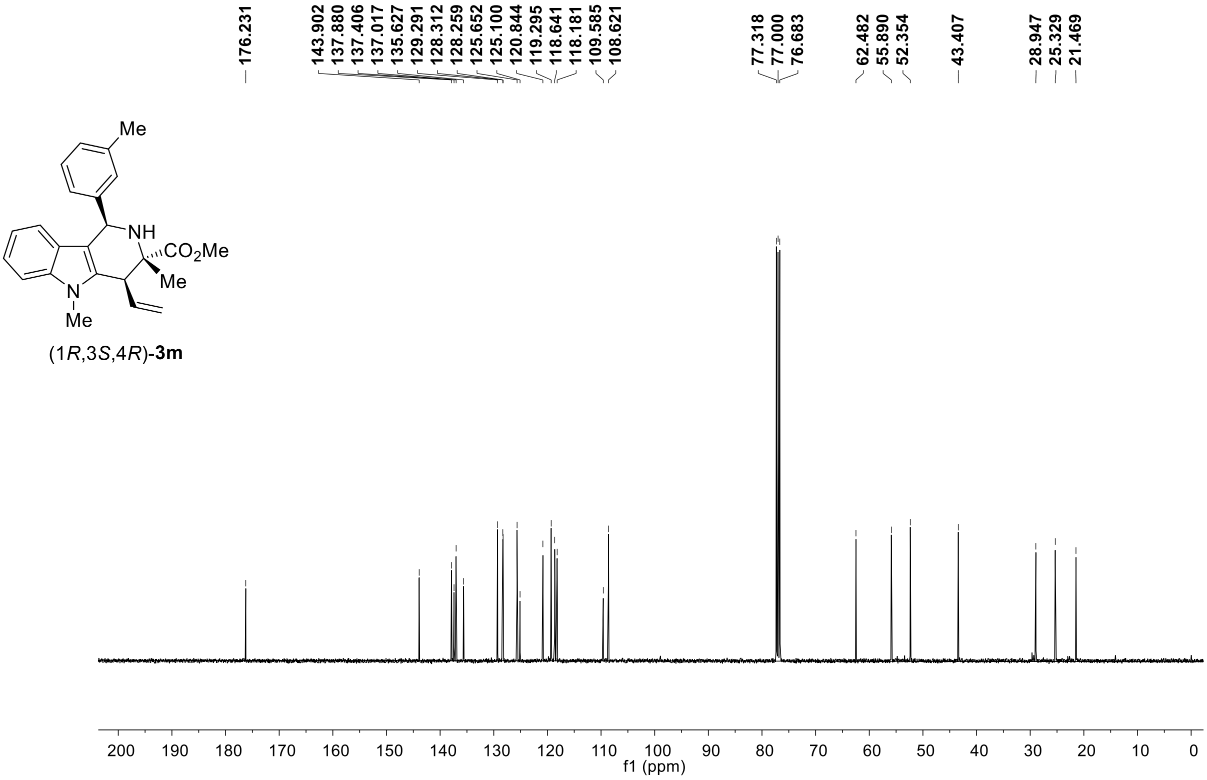

Supplementary Figure 60. ^{13}C NMR spectrum of (1*R*,3*S*,4*R*)-**3m**

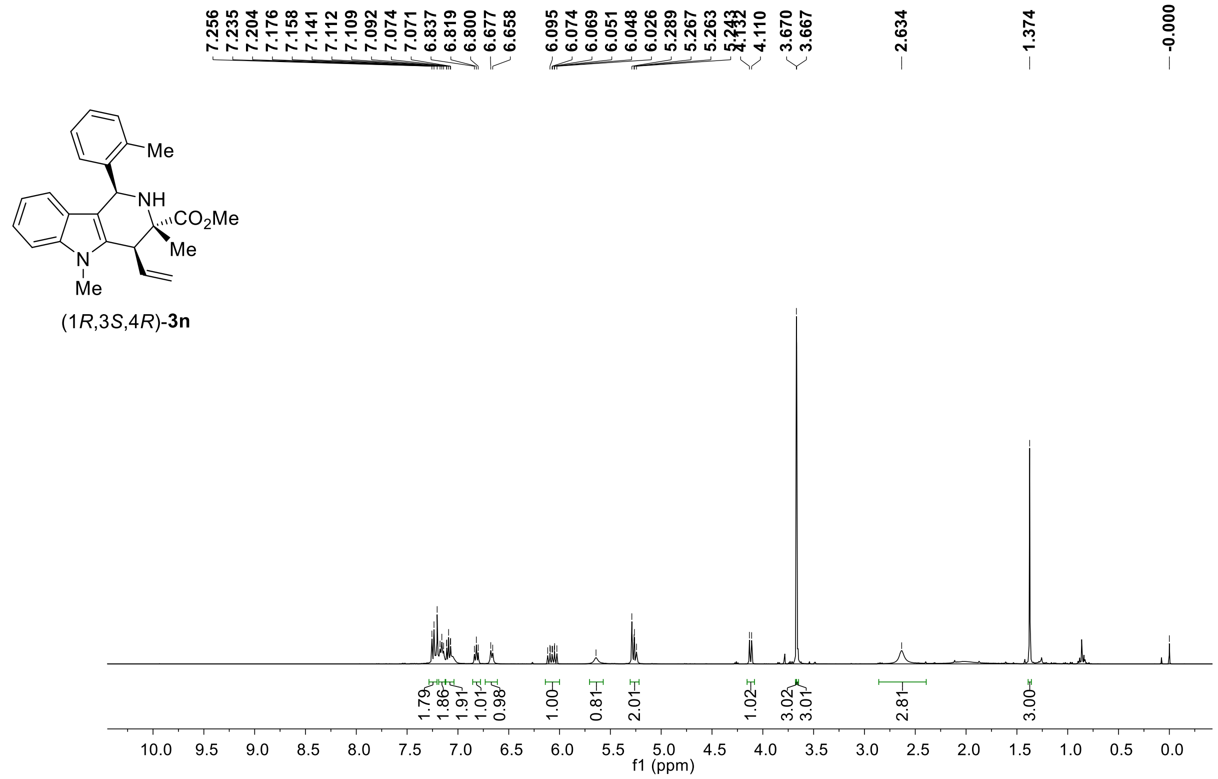

Supplementary Figure 61. ^{1}H NMR spectrum of (1*R*,3*S*,4*R*)-**3n**

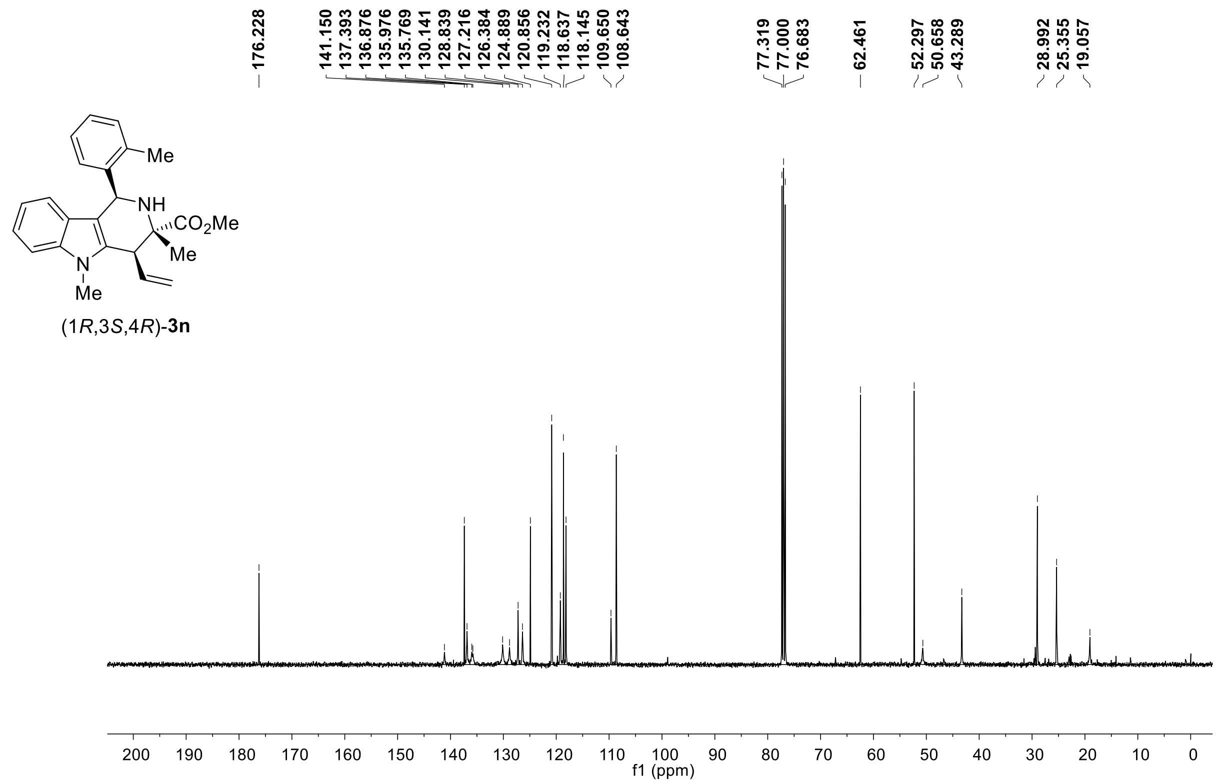

Supplementary Figure 62. ^{13}C NMR spectrum of (1*R*,3*S*,4*R*)-**3n**

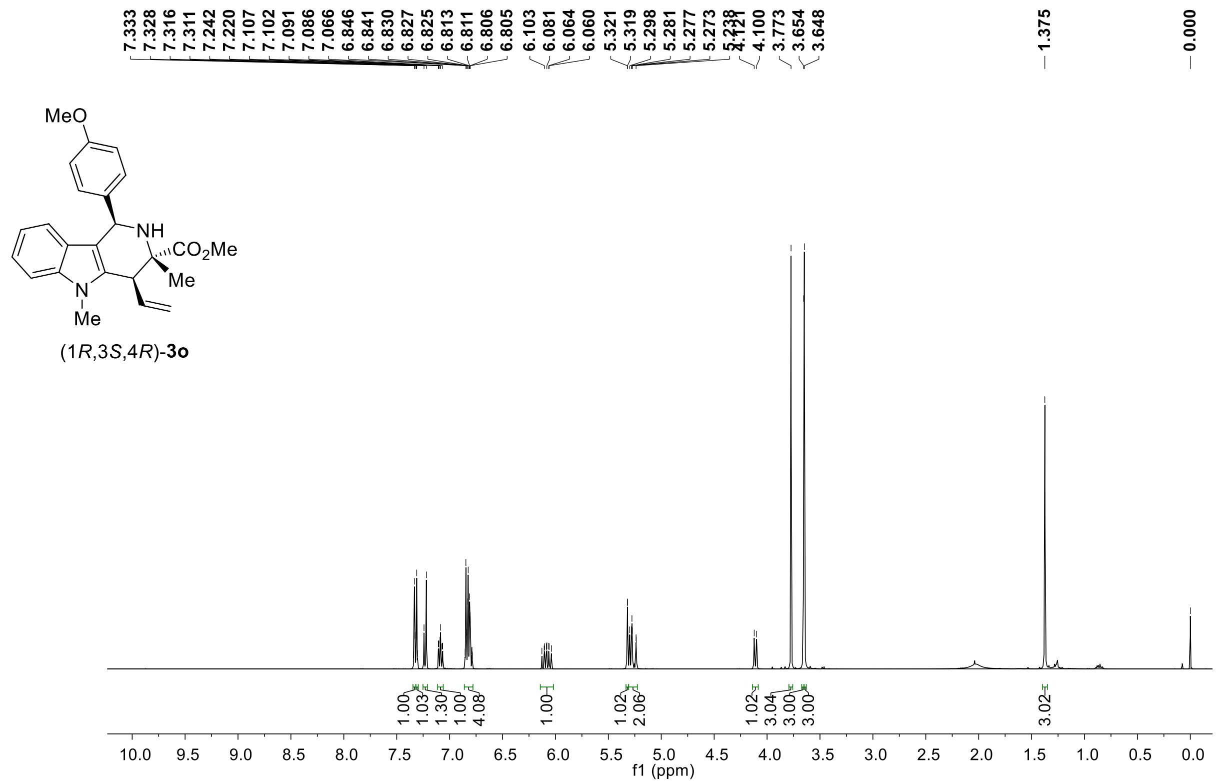

Supplementary Figure 63. ^{1}H NMR spectrum of (1*R*,3*S*,4*R*)-**3o**

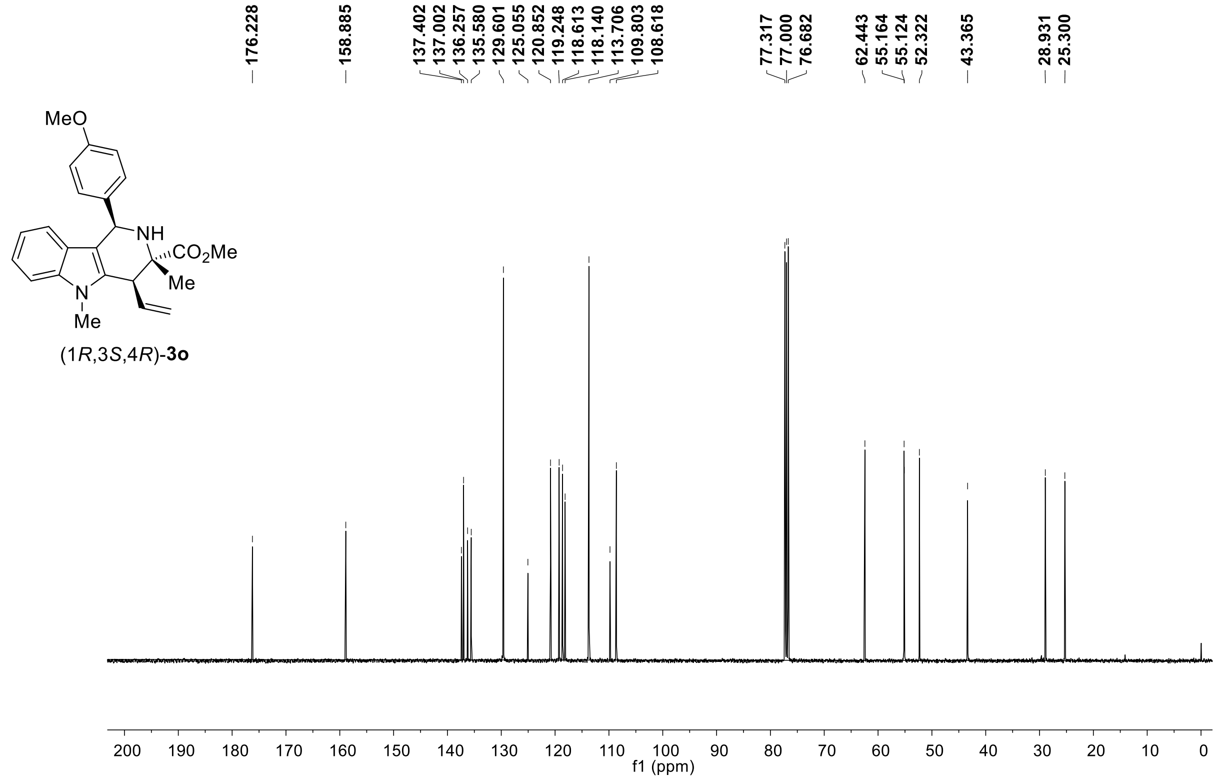

Supplementary Figure 64. ^{13}C NMR spectrum of (1*R*,3*S*,4*R*)-**3o**

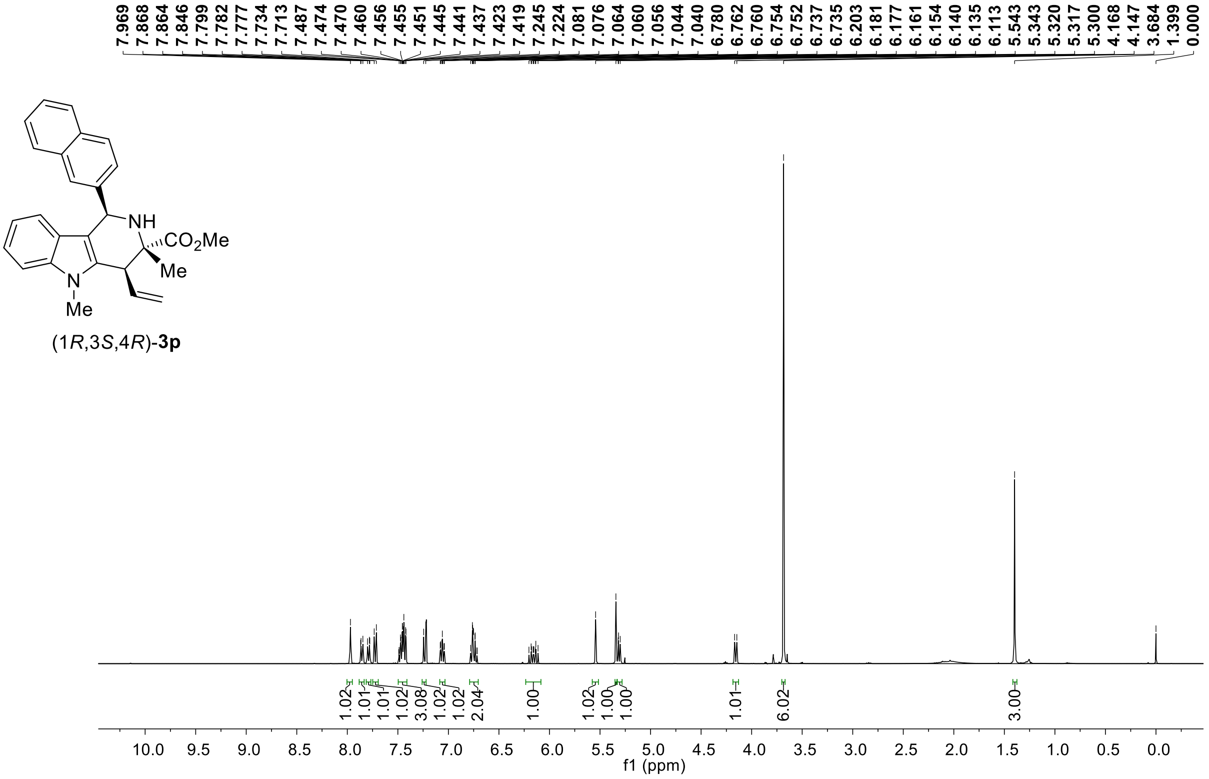

Supplementary Figure 65. ^{1}H NMR spectrum of (1*R*,3*S*,4*R*)-**3p**

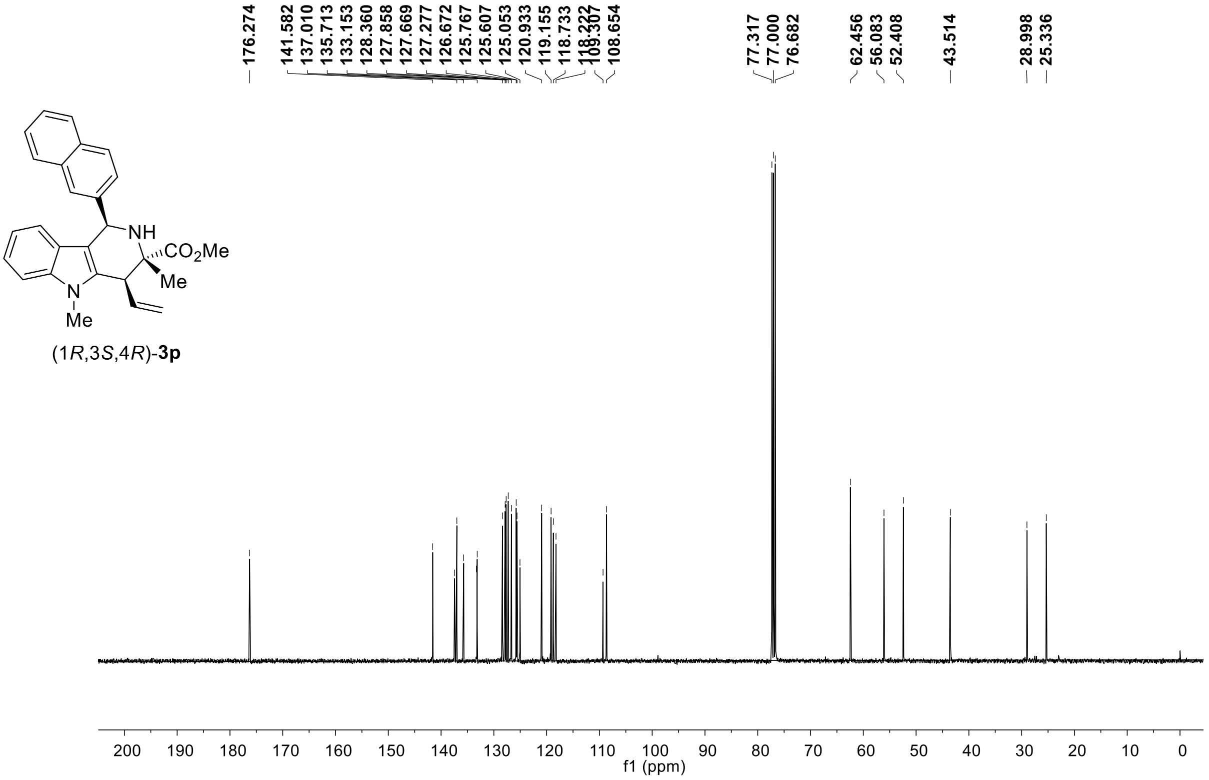

Supplementary Figure 66. ^{13}C NMR spectrum of (1*R*,3*S*,4*R*)-**3p**

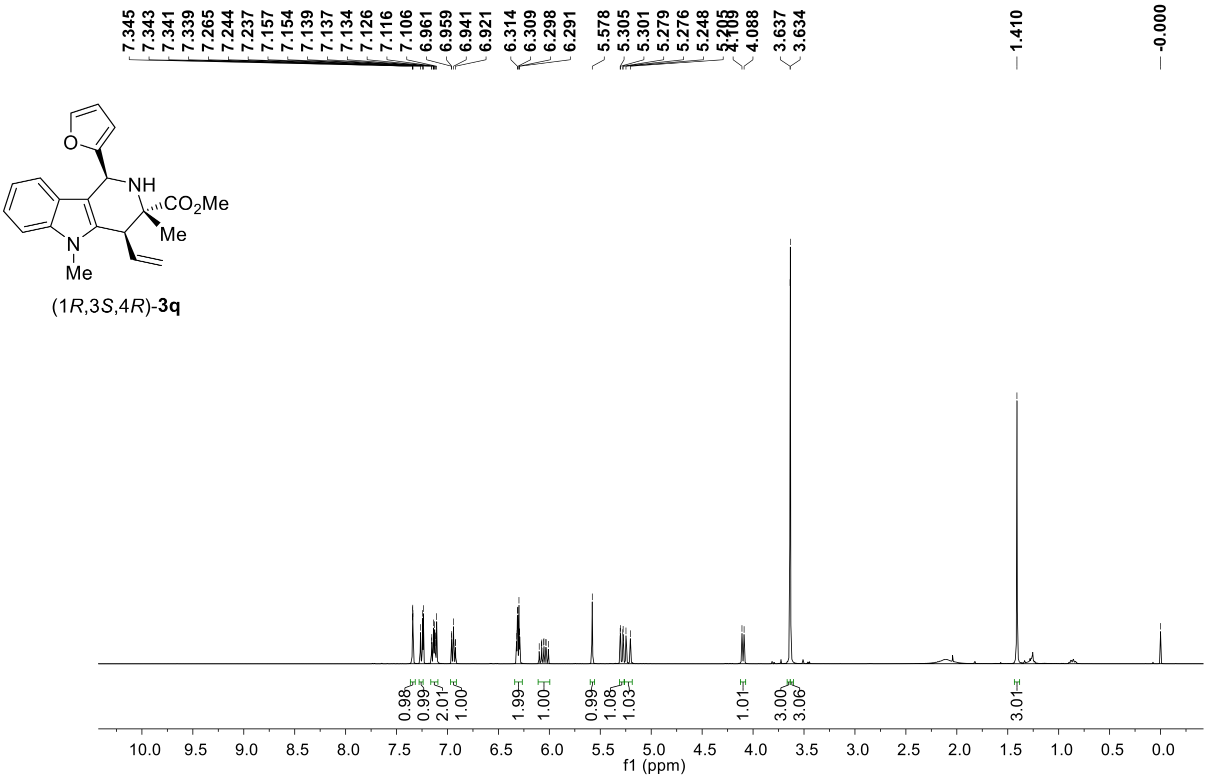

Supplementary Figure 67. ^{1}H NMR spectrum of (1*R*,3*S*,4*R*)-**3q**

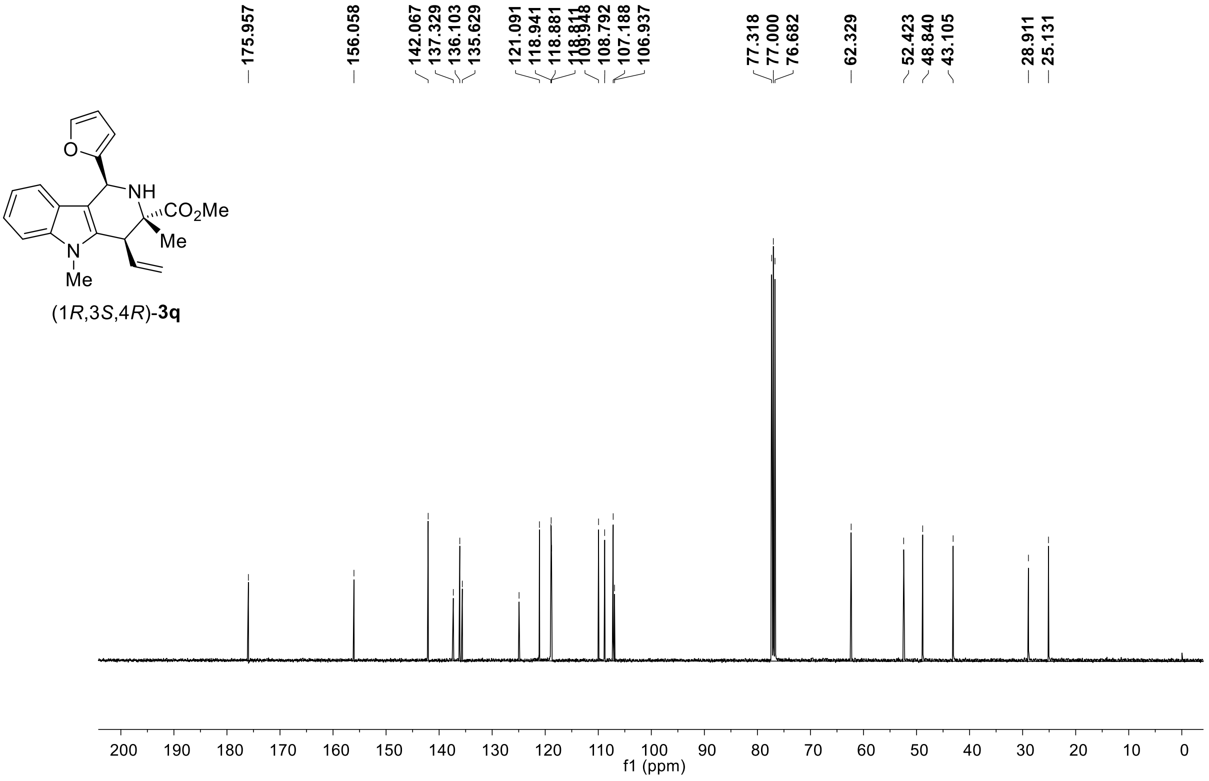

Supplementary Figure 68. ^{13}C NMR spectrum of (1*R*,3*S*,4*R*)-**3q**

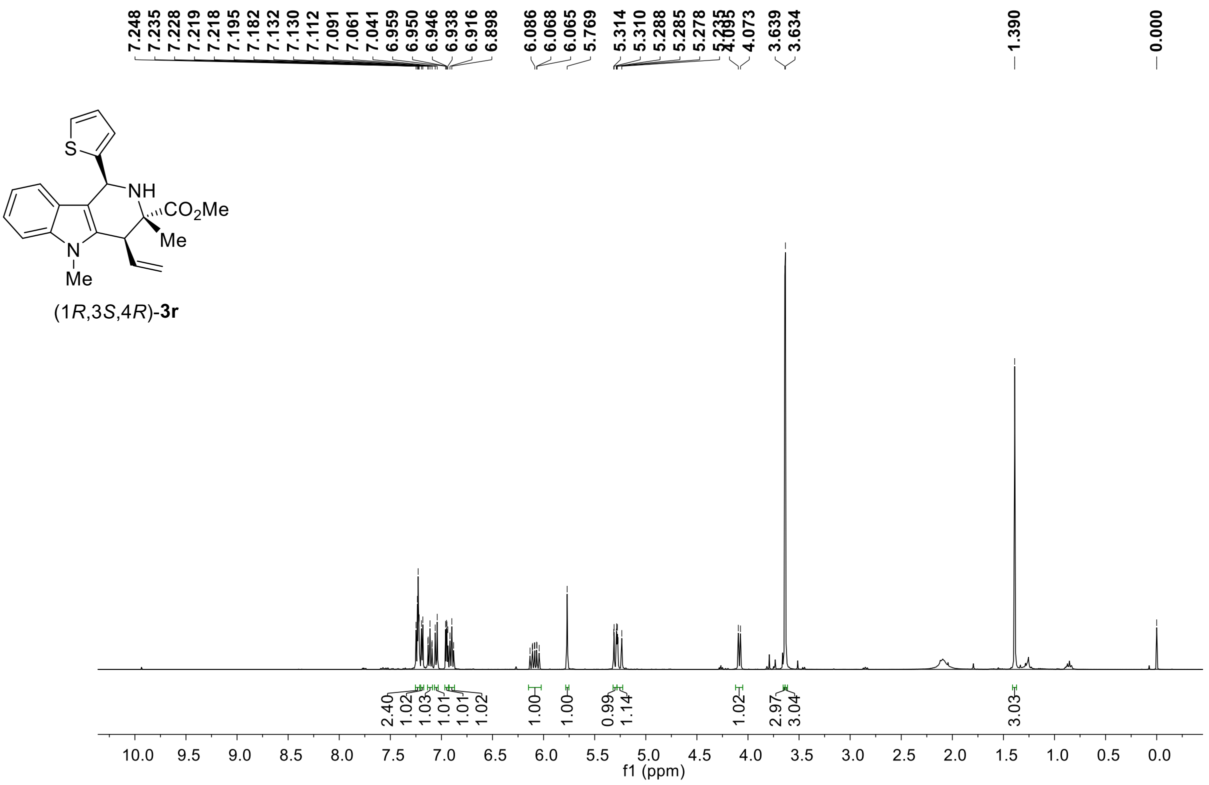

Supplementary Figure 69. ^{1}H NMR spectrum of (1*R*,3*S*,4*R*)-**3r**

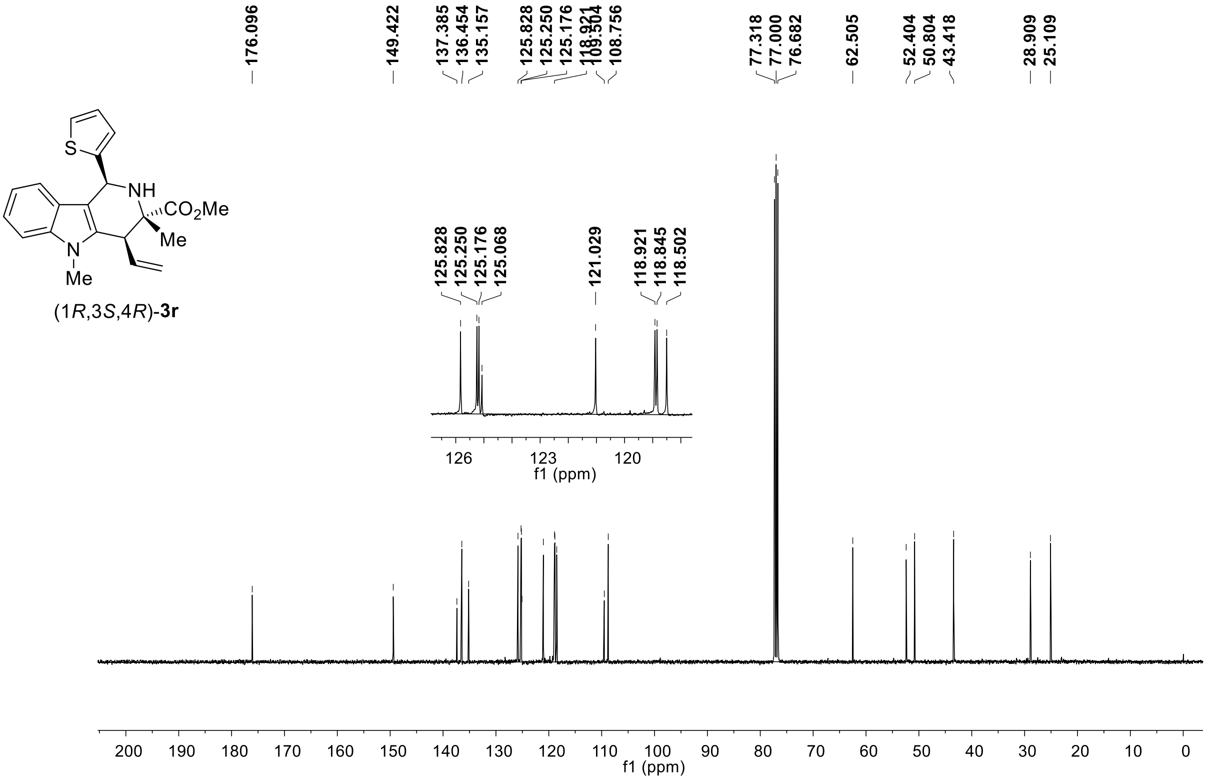

Supplementary Figure 70. ^{13}C NMR spectrum of (1*R*,3*S*,4*R*)-**3r**

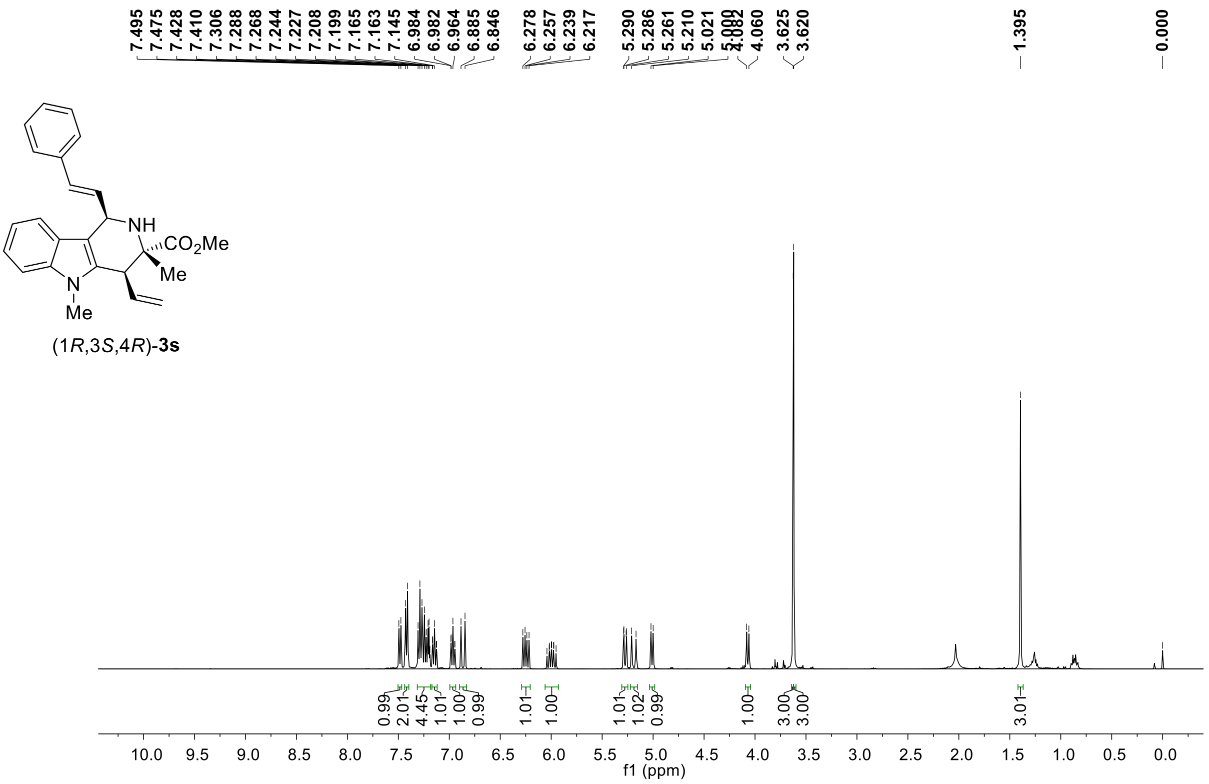

Supplementary Figure 71. ^{1}H NMR spectrum of (1*R*,3*S*,4*R*)-**3s**

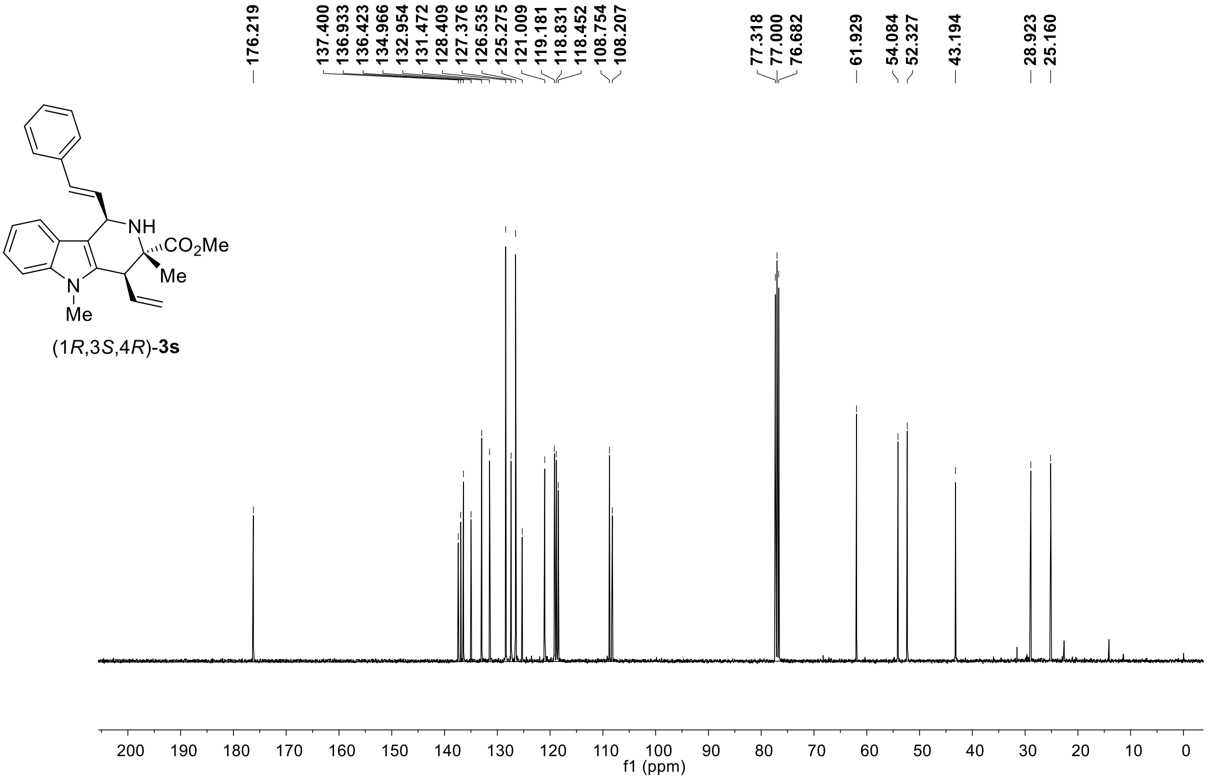

Supplementary Figure 72. ^{13}C NMR spectrum of (1*R*,3*S*,4*R*)-**3s**

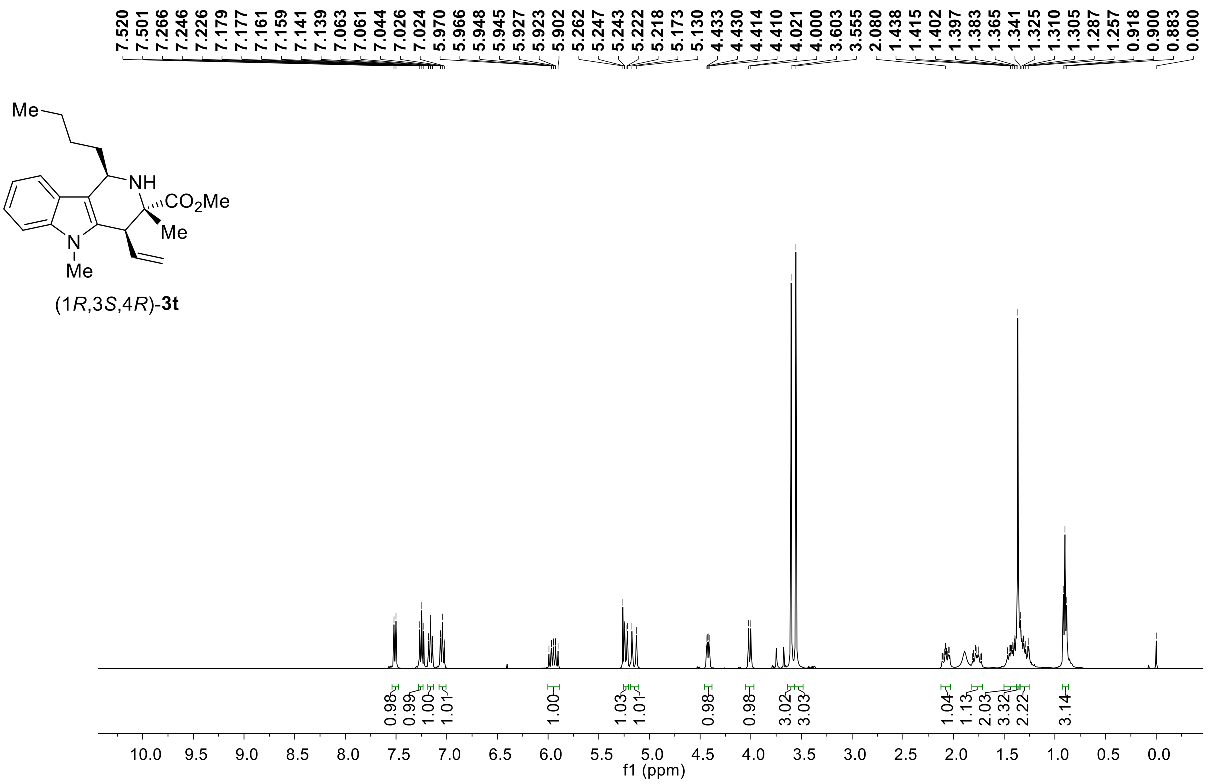

Supplementary Figure 73. ^{1}H NMR spectrum of (1*R*,3*S*,4*R*)-**3t**

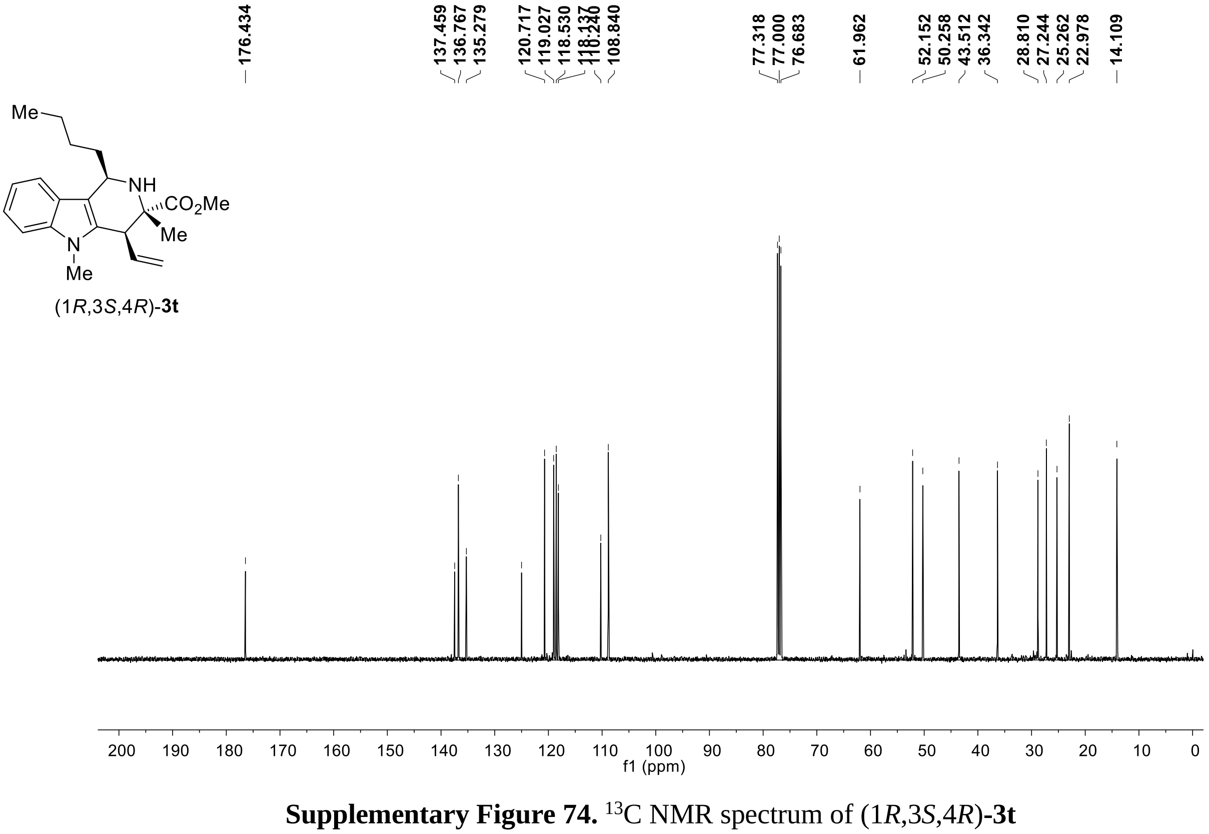

Supplementary Figure 74. ^{13}C NMR spectrum of (1*R*,3*S*,4*R*)-**3t**

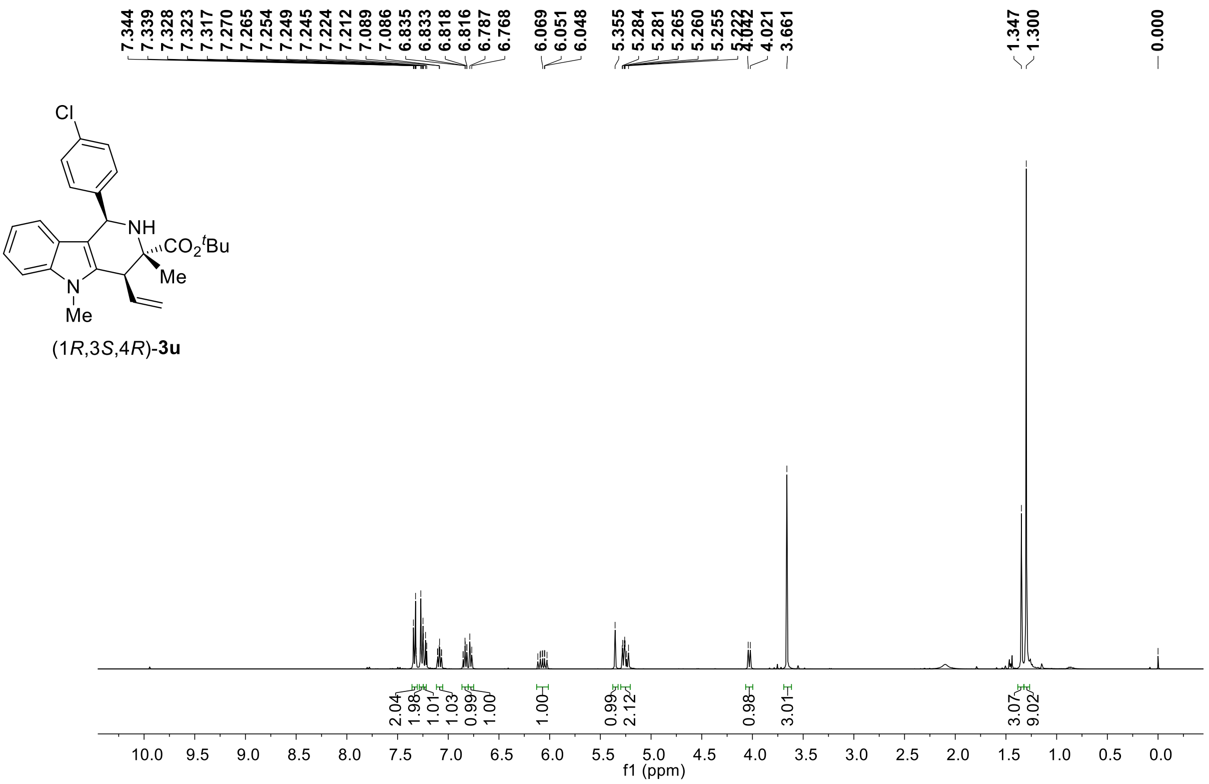

Supplementary Figure 75. ^{1}H NMR spectrum of (1*R*,3*S*,4*R*)-**3u**

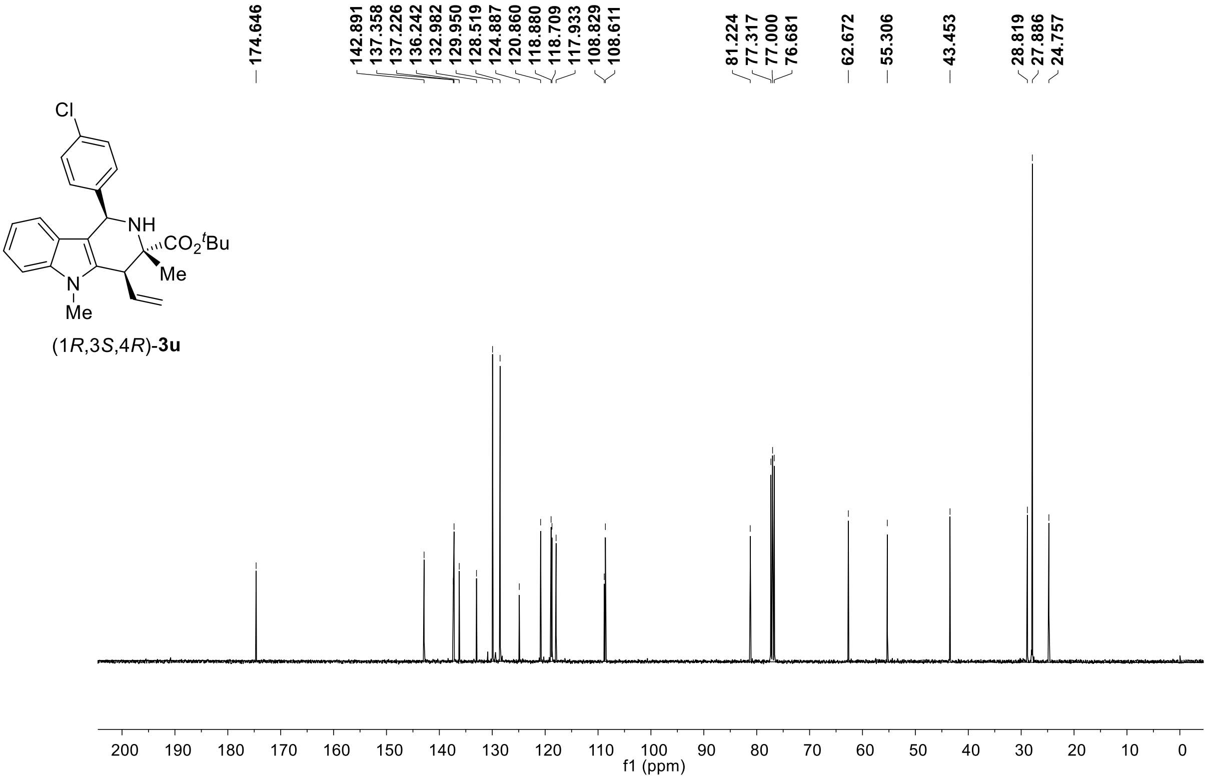

Supplementary Figure 76. ^{13}C NMR spectrum of (1*R*,3*S*,4*R*)-**3u**

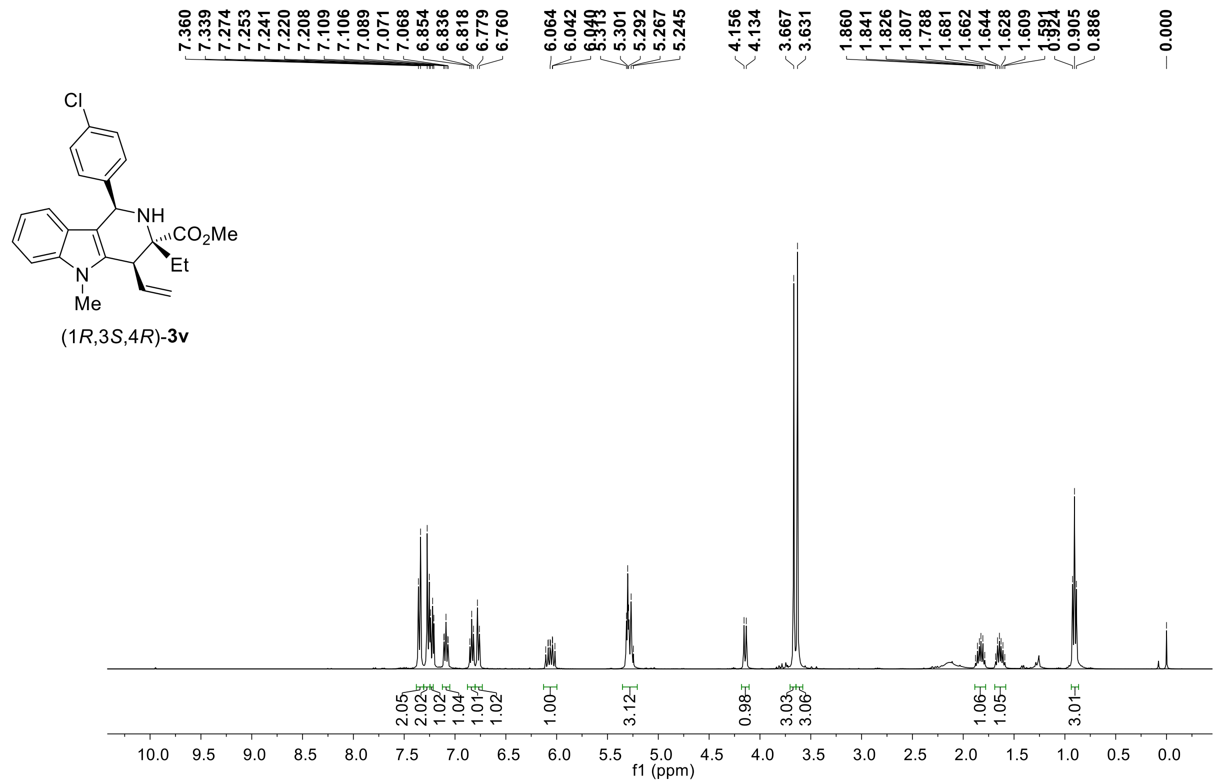

Supplementary Figure 77. ^{1}H NMR spectrum of (1*R*,3*S*,4*R*)-**3v**

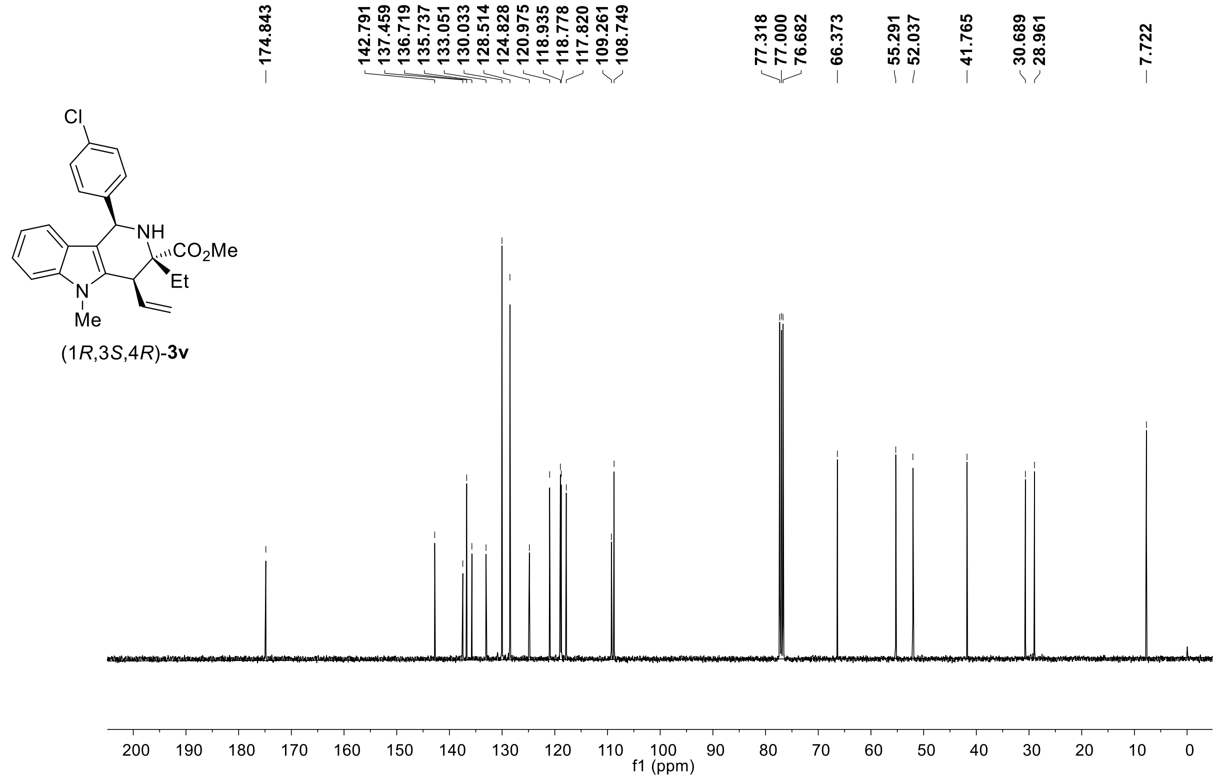

Supplementary Figure 78. ^{13}C NMR spectrum of (1*R*,3*S*,4*R*)-**3v**

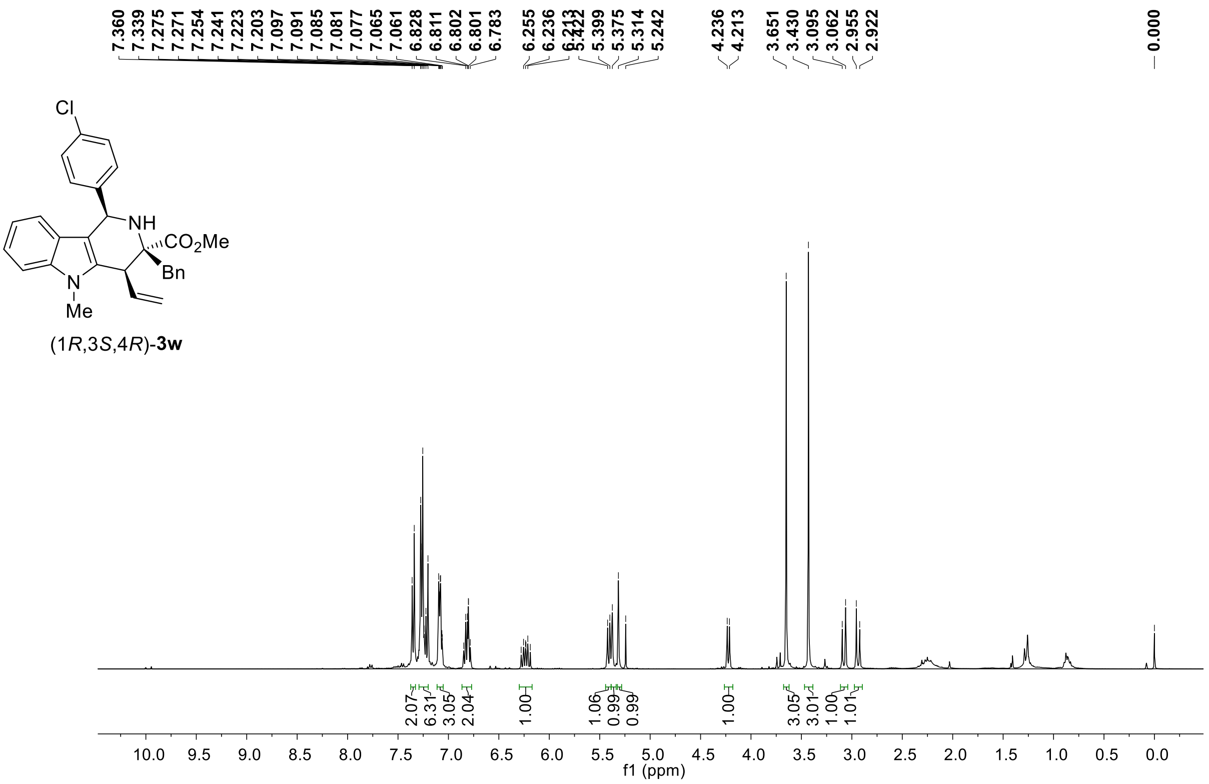

Supplementary Figure 79. ^{1}H NMR spectrum of (1*R*,3*S*,4*R*)-**3w**

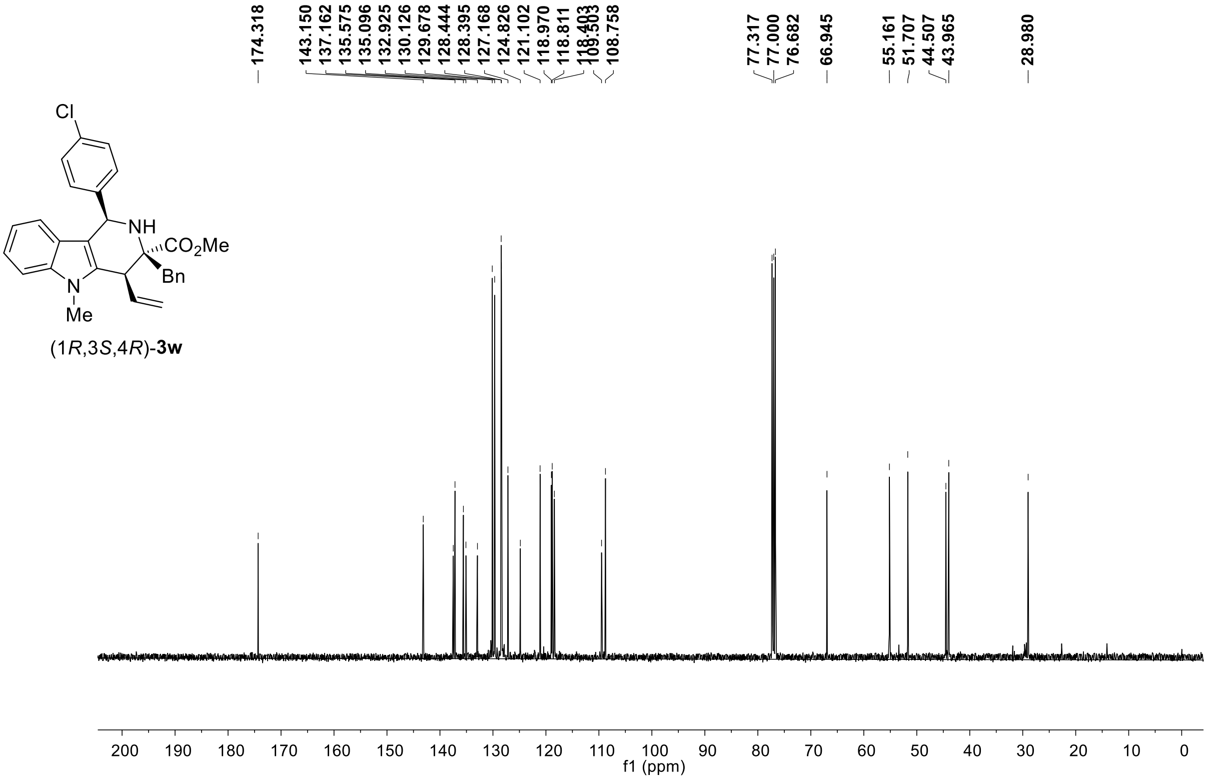

Supplementary Figure 80. ^{13}C NMR spectrum of (1*R*,3*S*,4*R*)-**3w**

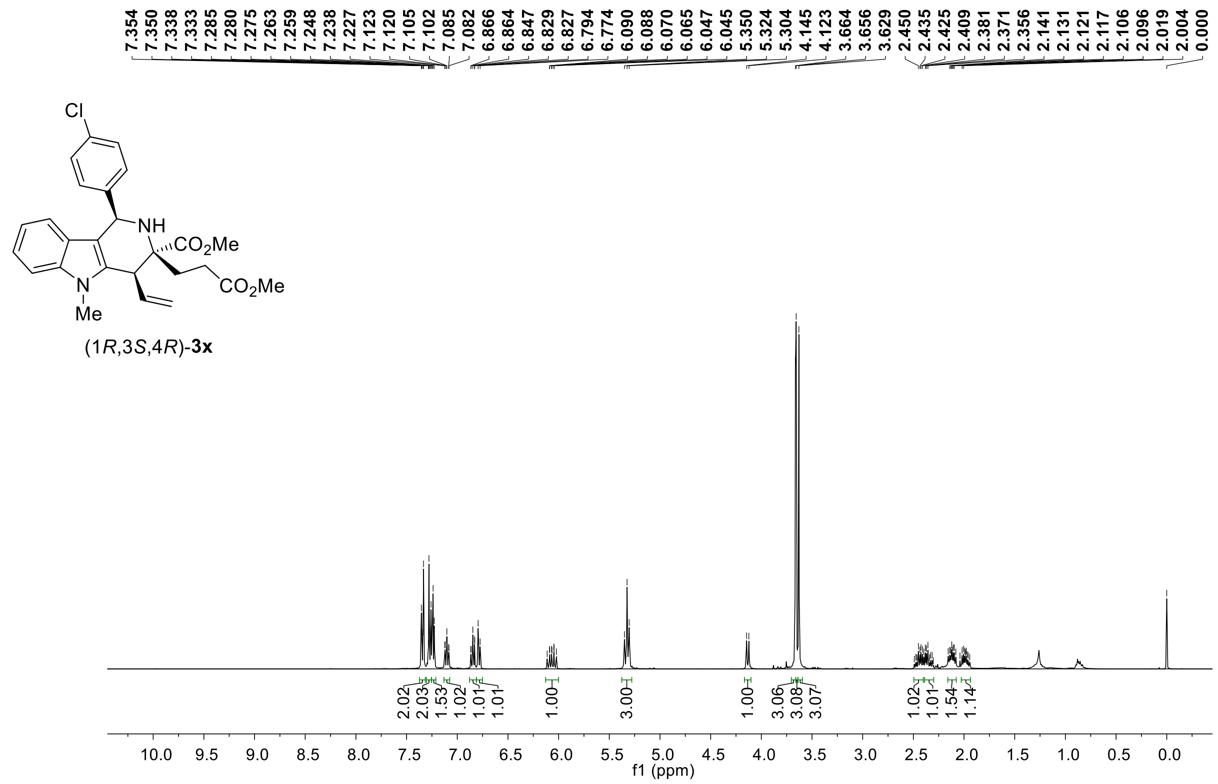

Supplementary Figure 81. ^{1}H NMR spectrum of (1*R*,3*S*,4*R*)-**3x**

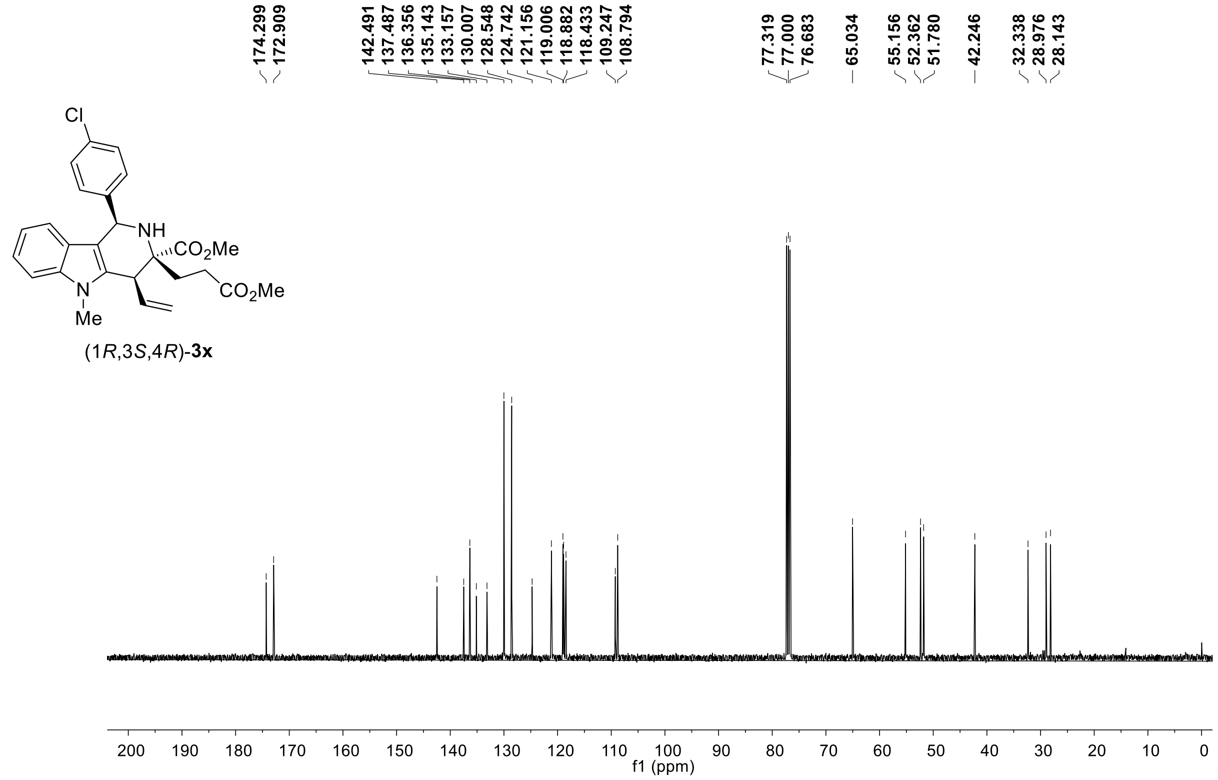

Supplementary Figure 82. ^{13}C NMR spectrum of (1*R*,3*S*,4*R*)-**3x**

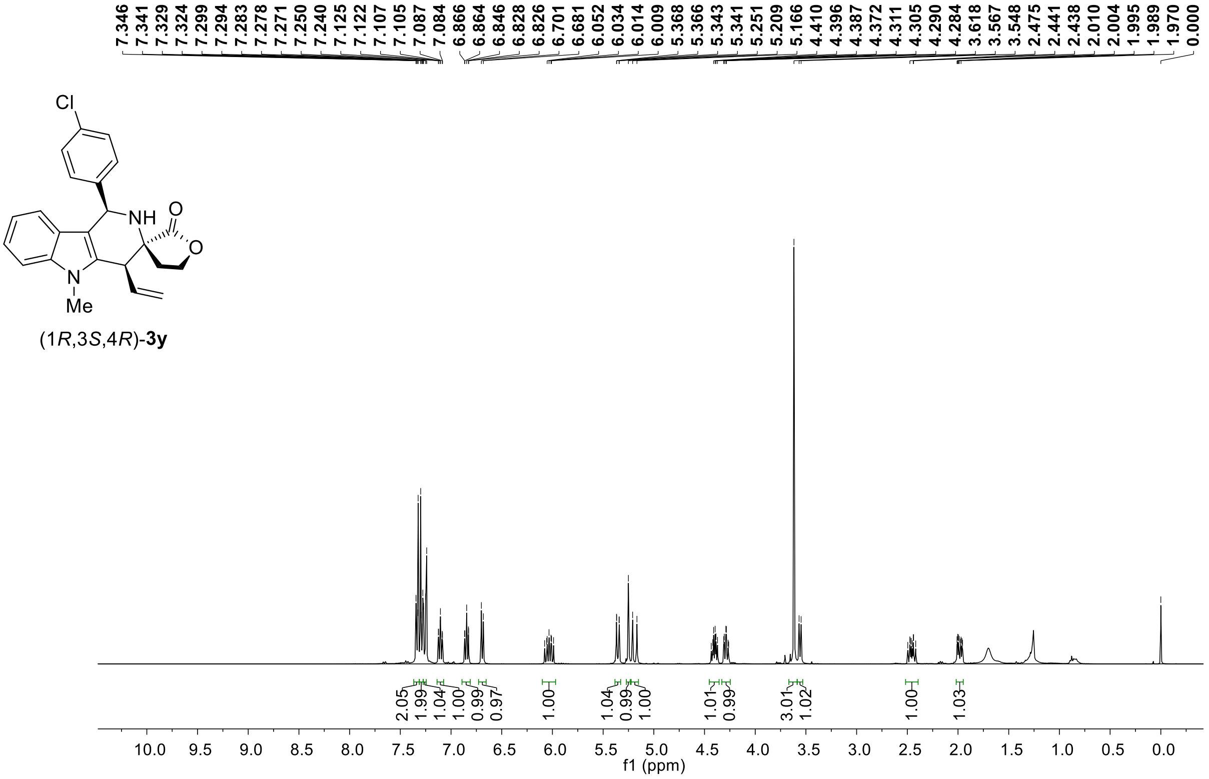

Supplementary Figure 83. ^{1}H NMR spectrum of (1*R*,3*S*,4*R*)-**3y**

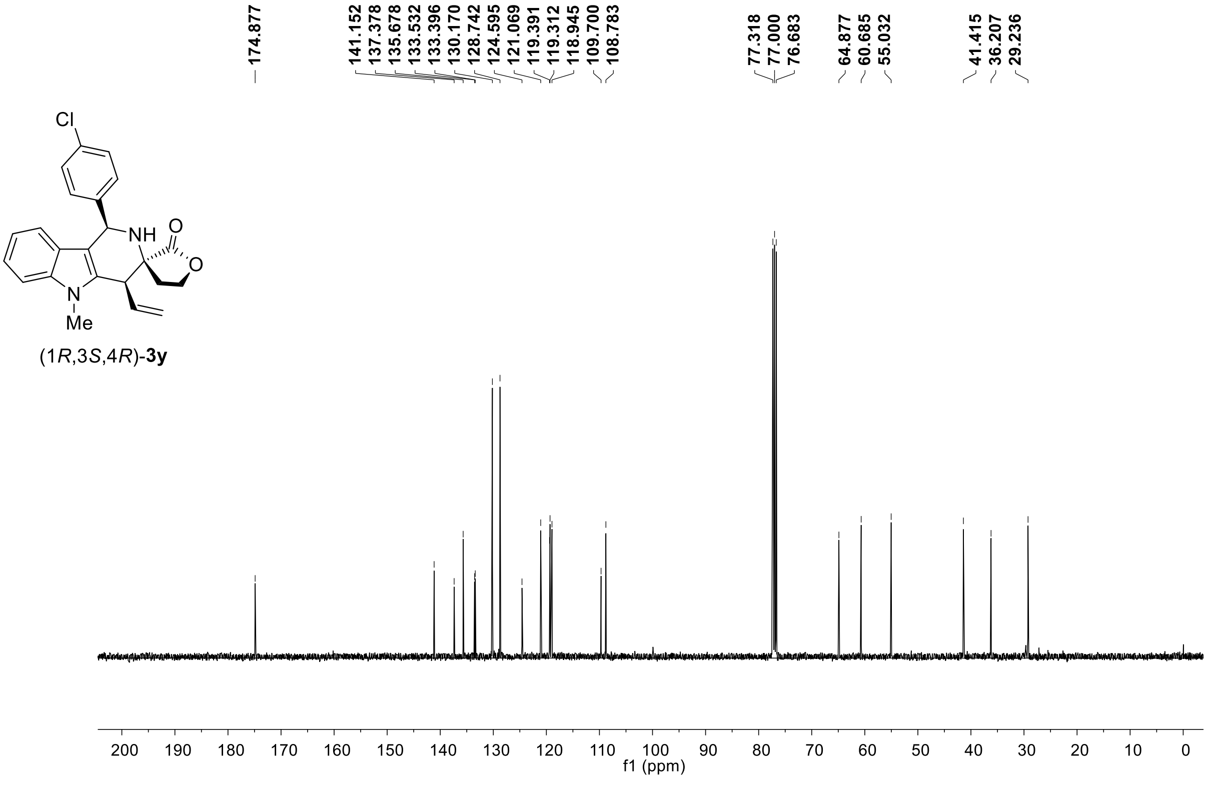

Supplementary Figure 84. ^{13}C NMR spectrum of (1*R*,3*S*,4*R*)-**3y**

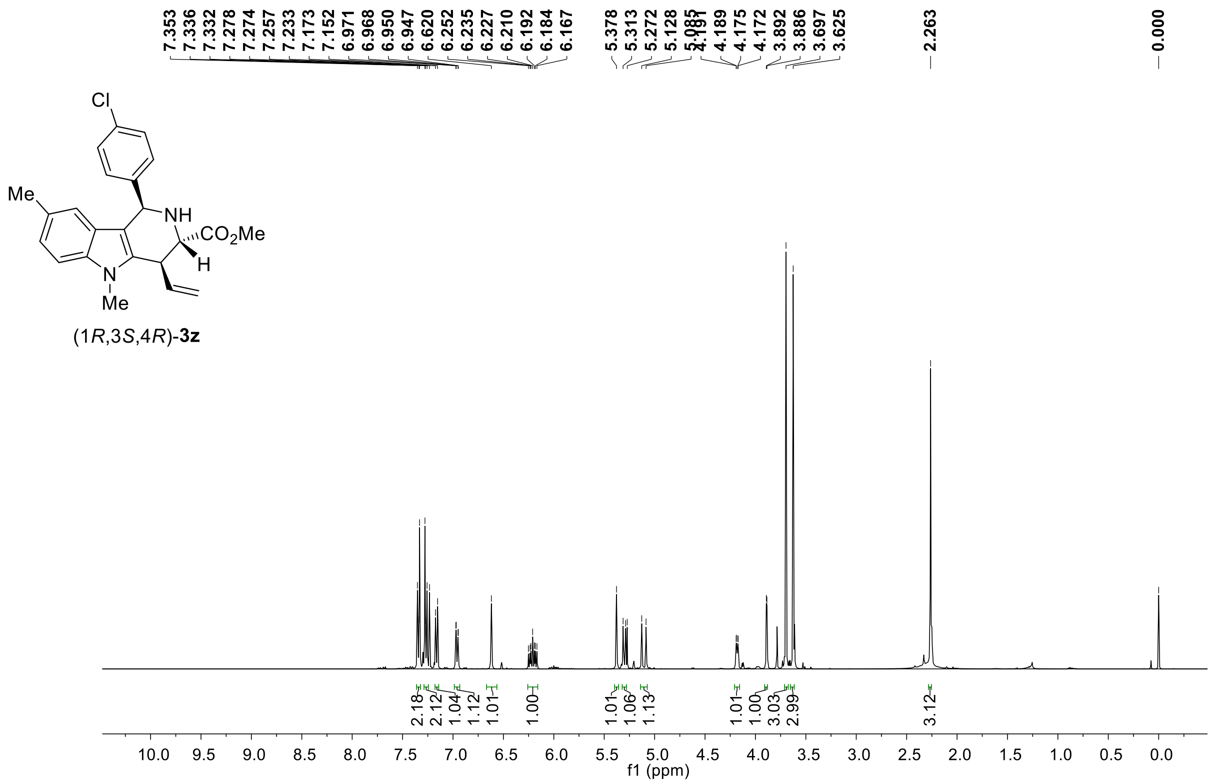

Supplementary Figure 85. ^{1}H NMR spectrum of (1*R*,3*S*,4*R*)-**3z**

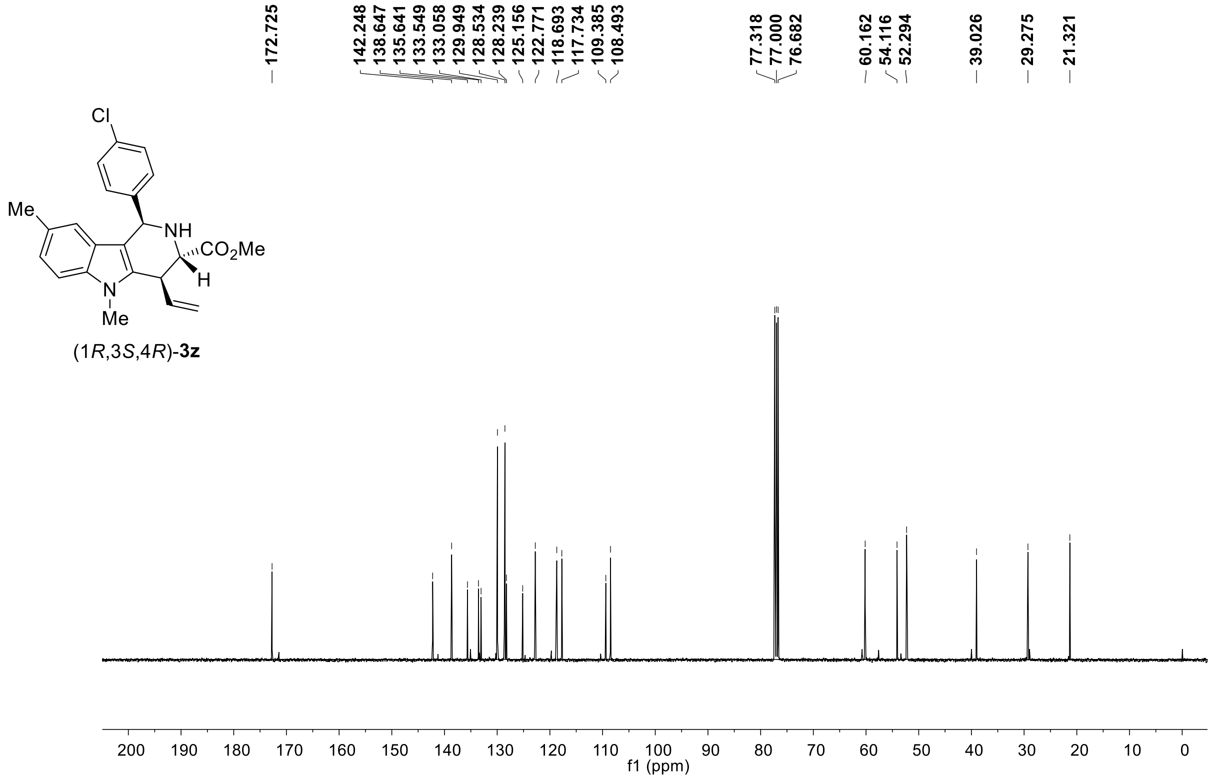

Supplementary Figure 86. ^{13}C NMR spectrum of (1*R*,3*S*,4*R*)-**3z**

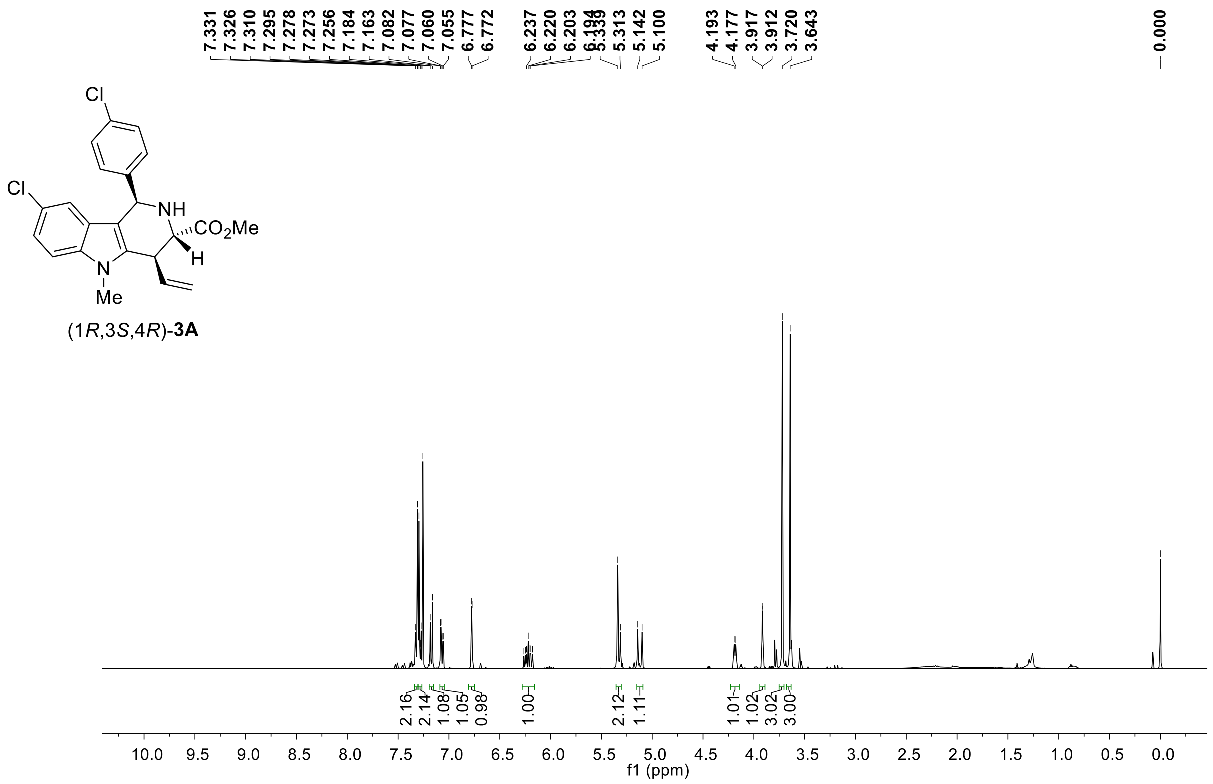

Supplementary Figure 87. ^{1}H NMR spectrum of (1*R*,3*S*,4*R*)-**3A**

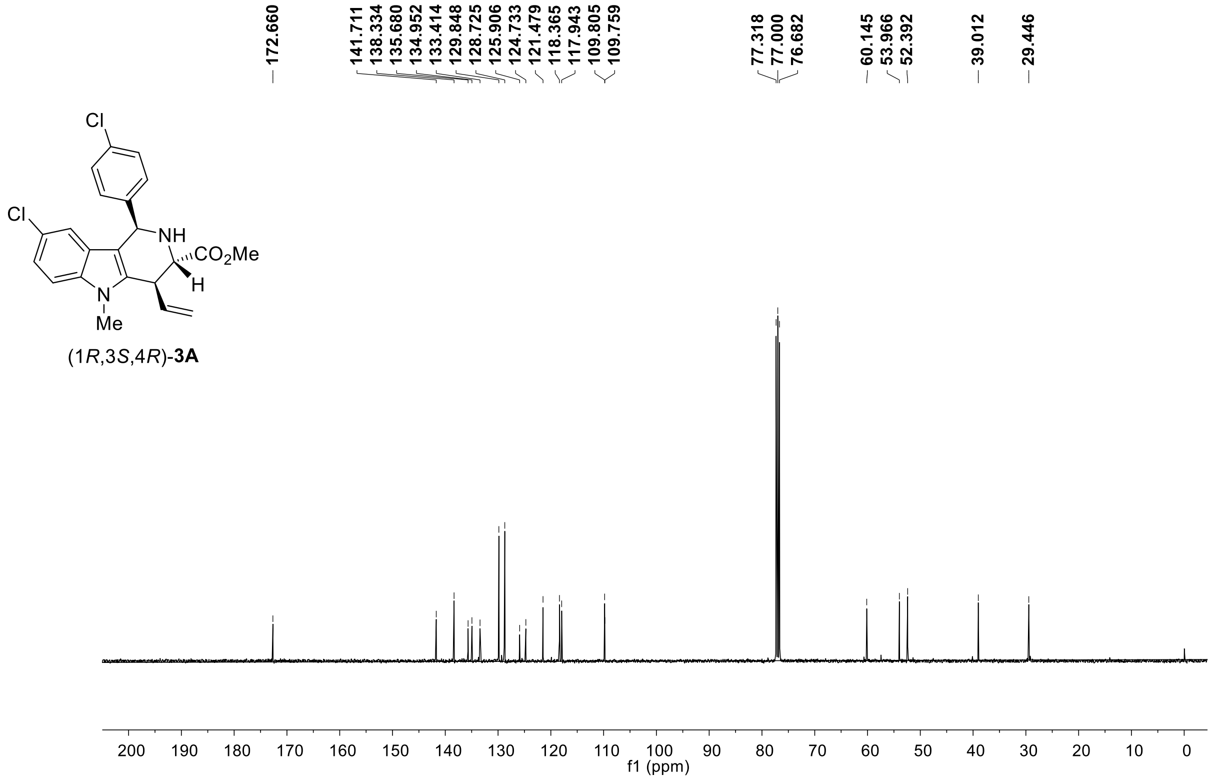

Supplementary Figure 88. ^{13}C NMR spectrum of (1*R*,3*S*,4*R*)-**3A**

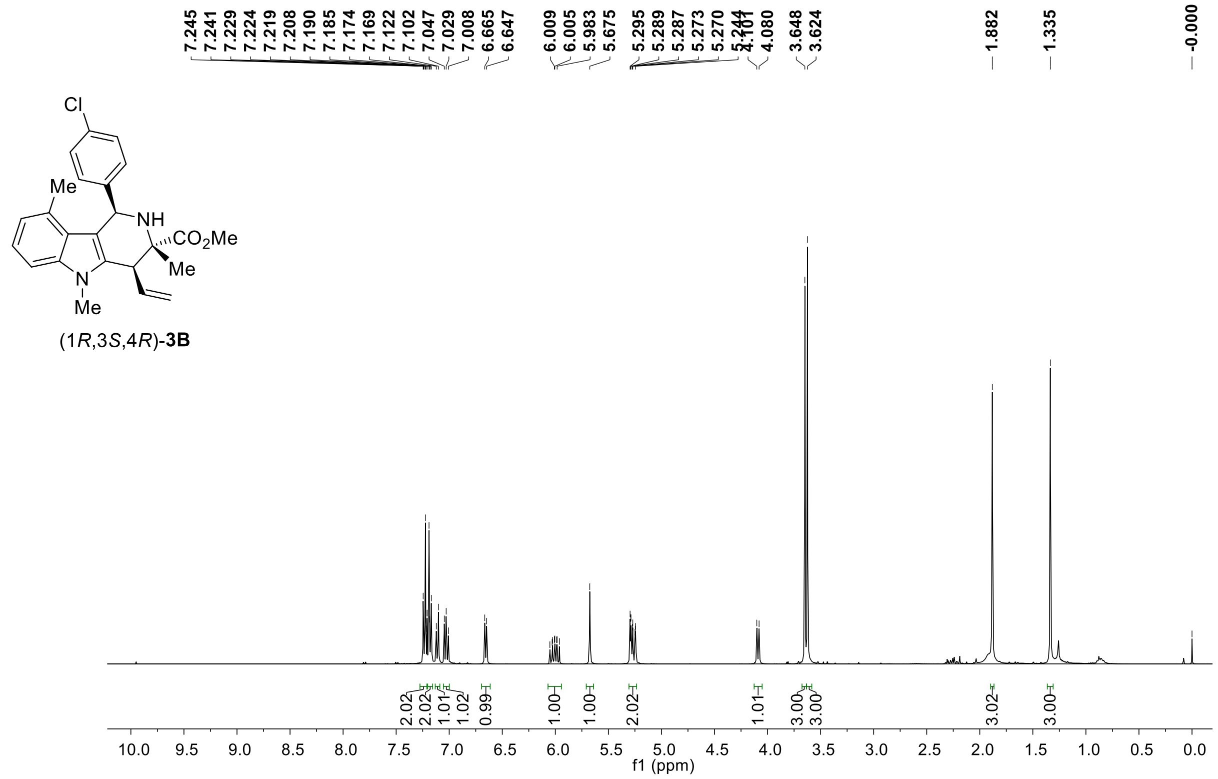

Supplementary Figure 89. ^{1}H NMR spectrum of (1*R*,3*S*,4*R*)-**3B**

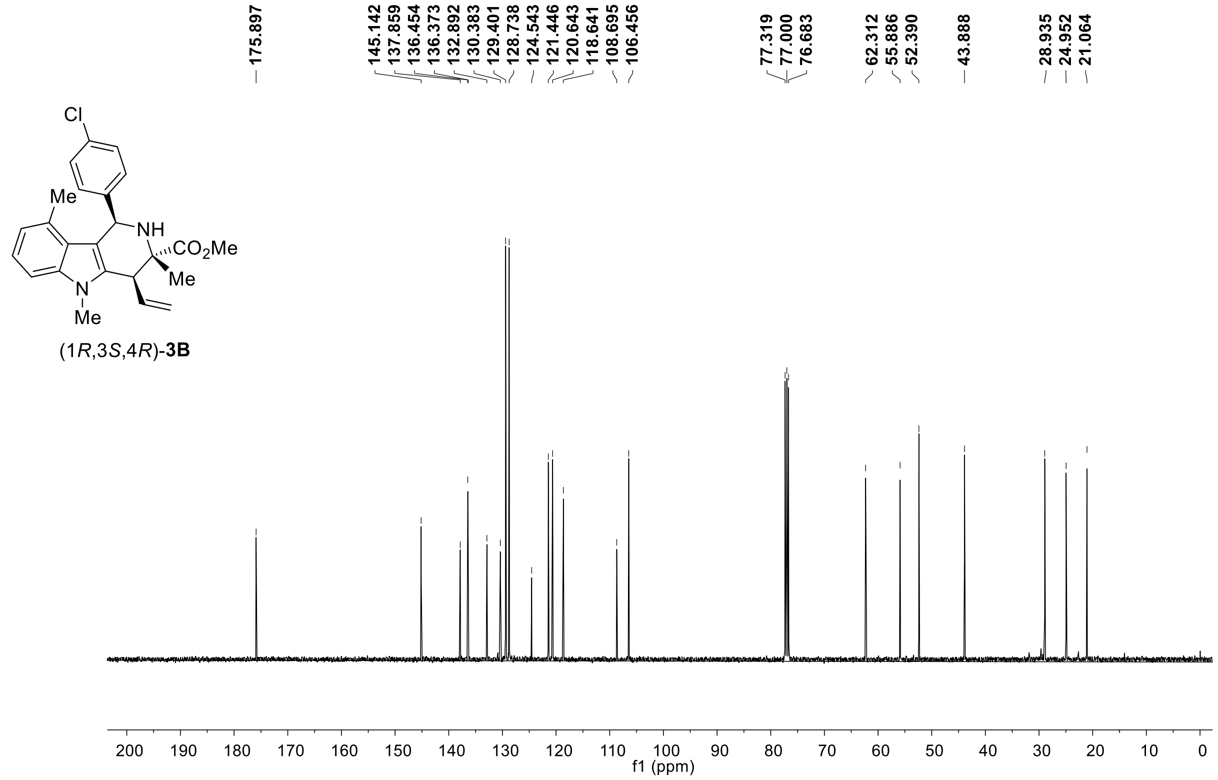

Supplementary Figure 90. ^{13}C NMR spectrum of (1*R*,3*S*,4*R*)-**3B**

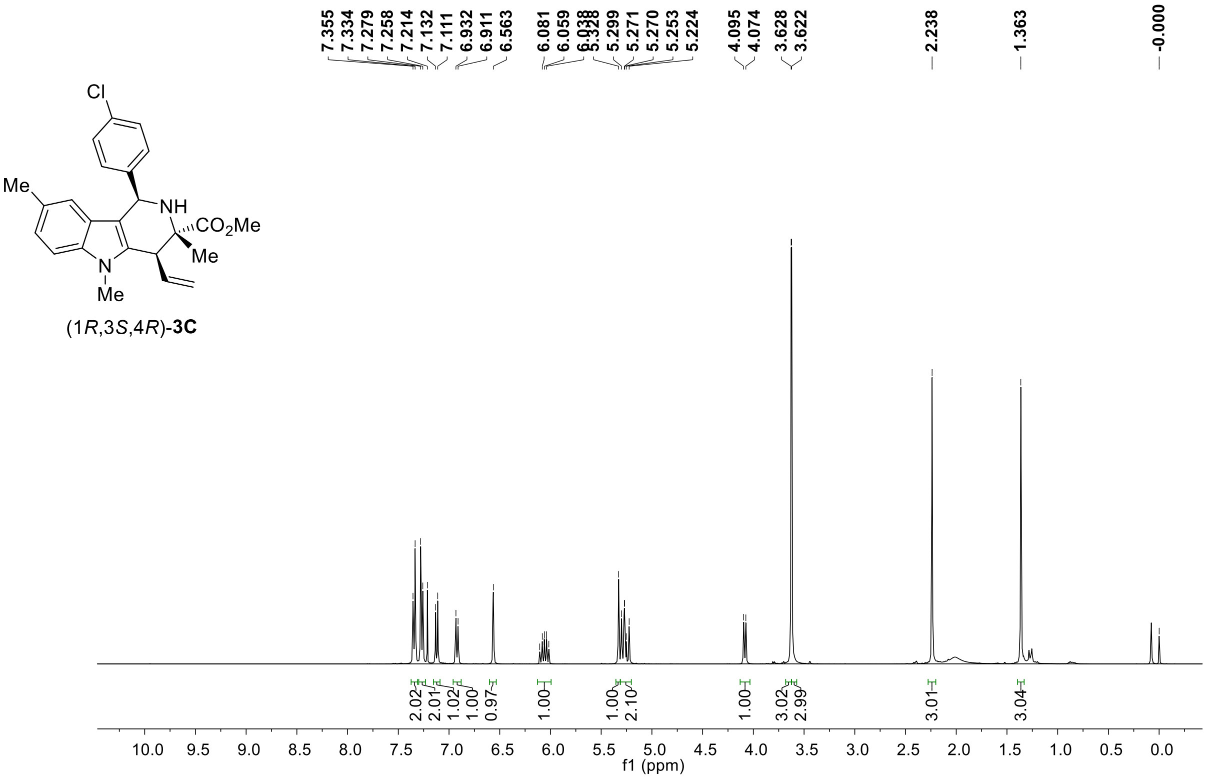

Supplementary Figure 91. ^{1}H NMR spectrum of (1*R*,3*S*,4*R*)-**3C**

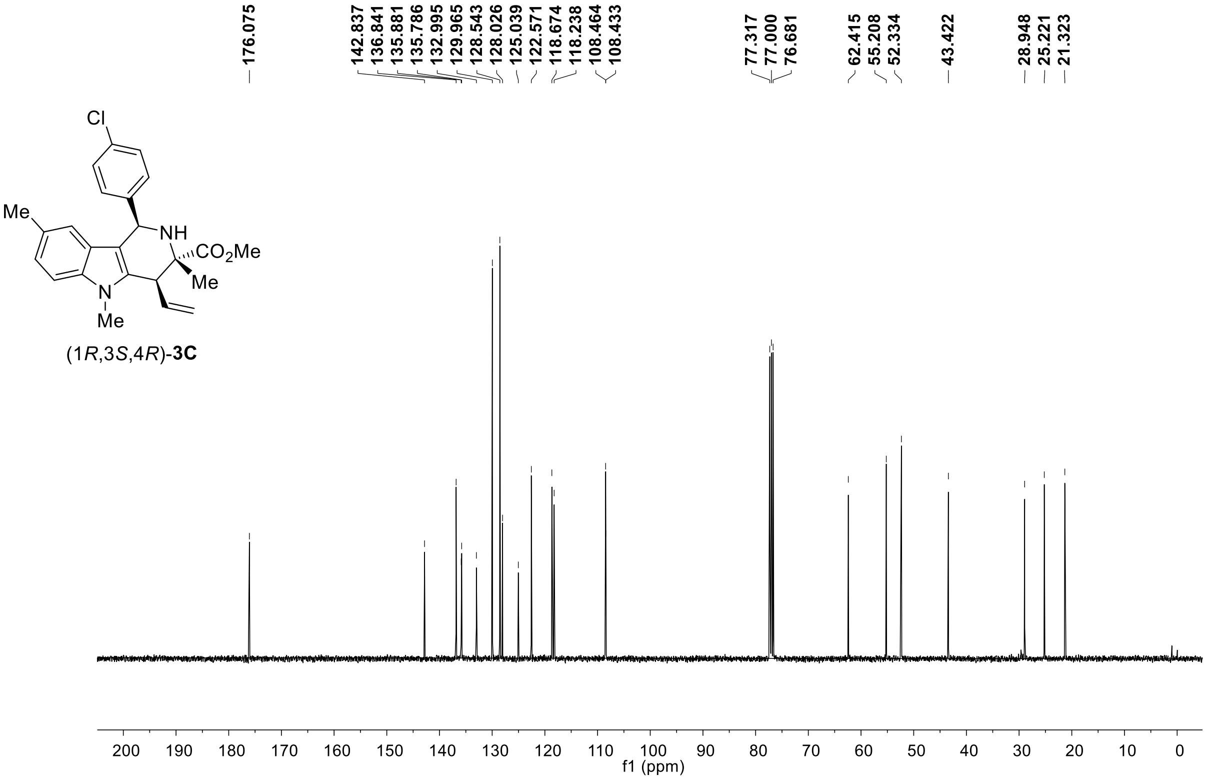

Supplementary Figure 92. ^{13}C NMR spectrum of (1*R*,3*S*,4*R*)-**3C**

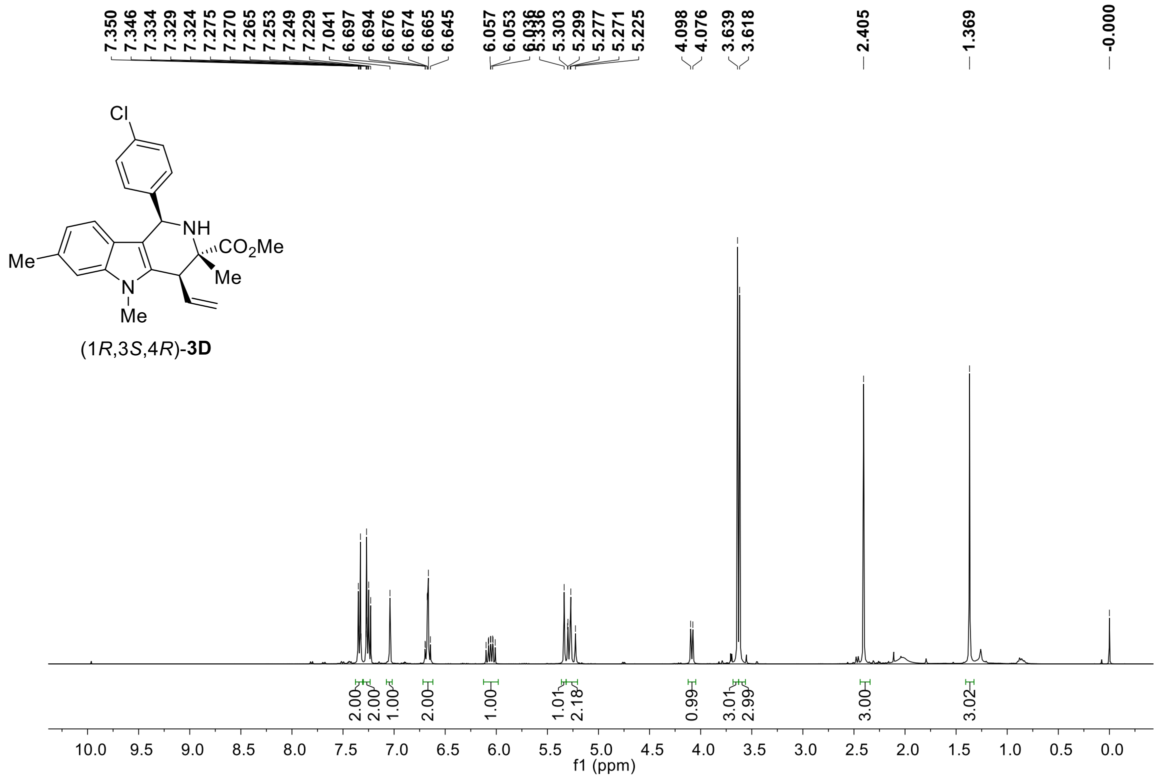

Supplementary Figure 93. ^{1}H NMR spectrum of (1*R*,3*S*,4*R*)-**3D**

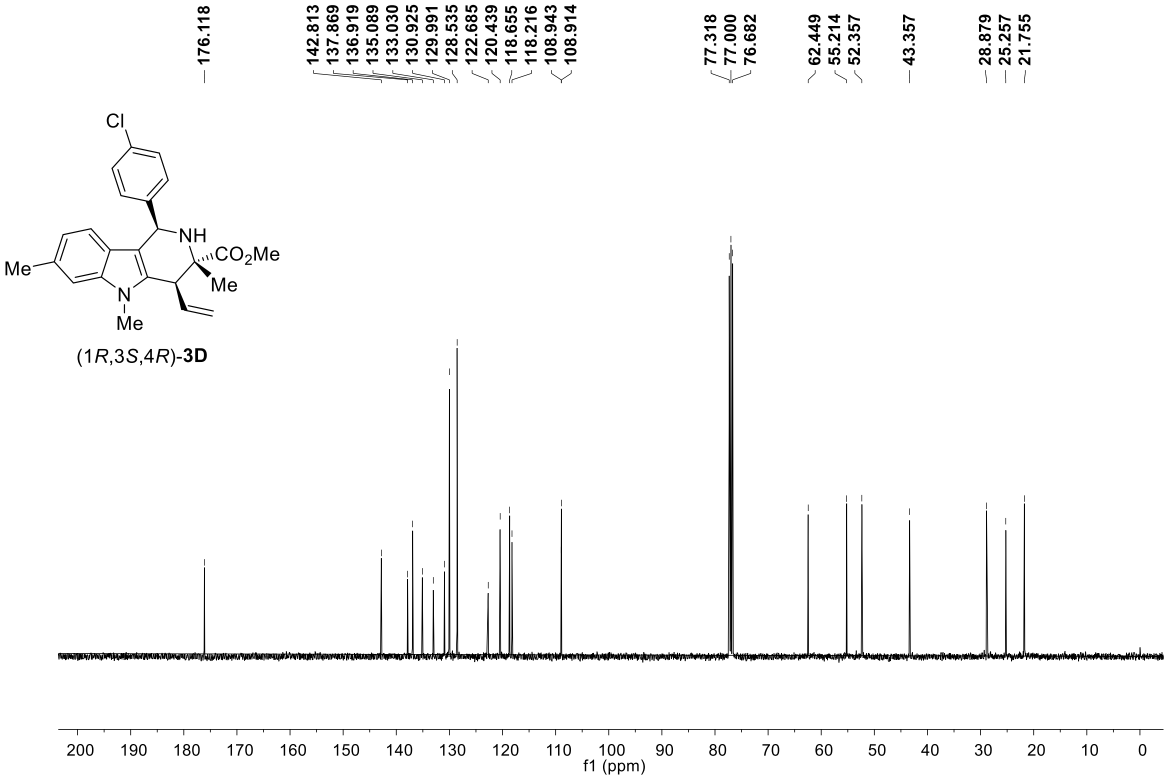

Supplementary Figure 94. ^{13}C NMR spectrum of (1*R*,3*S*,4*R*)-**3D**

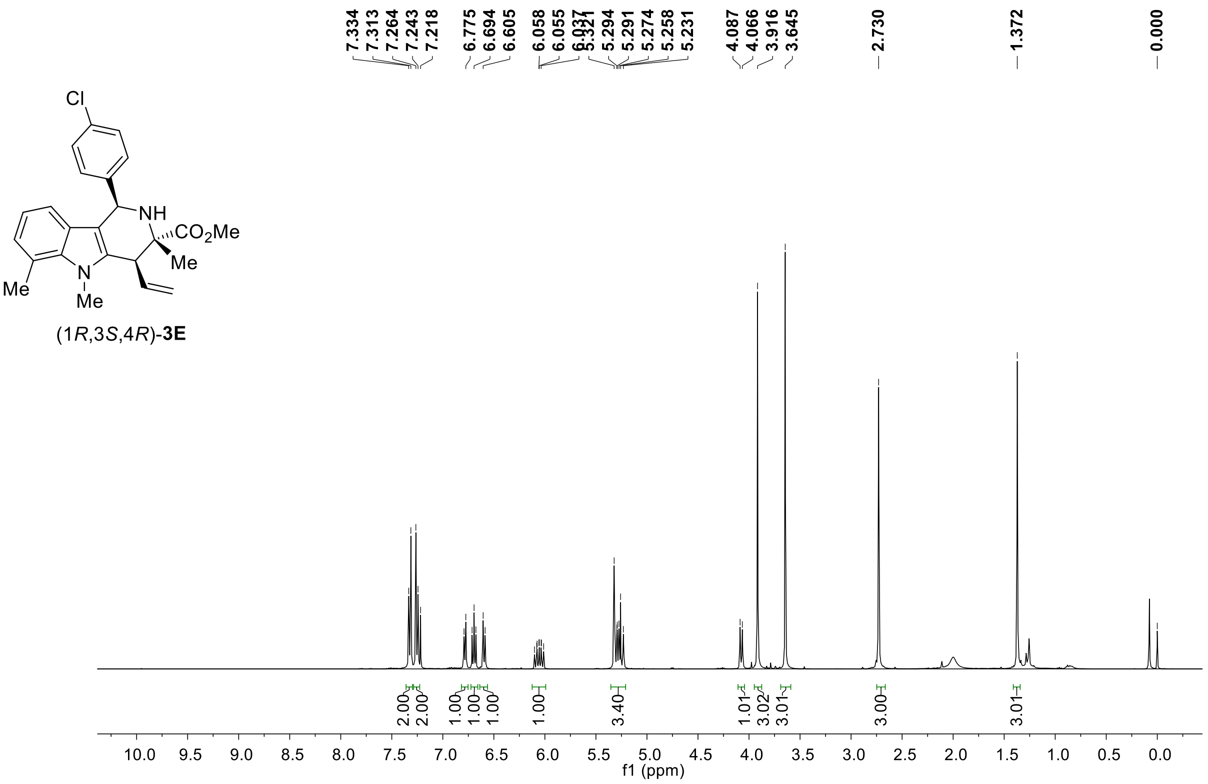

Supplementary Figure 95. ^{1}H NMR spectrum of (1*R*,3*S*,4*R*)-**3E**

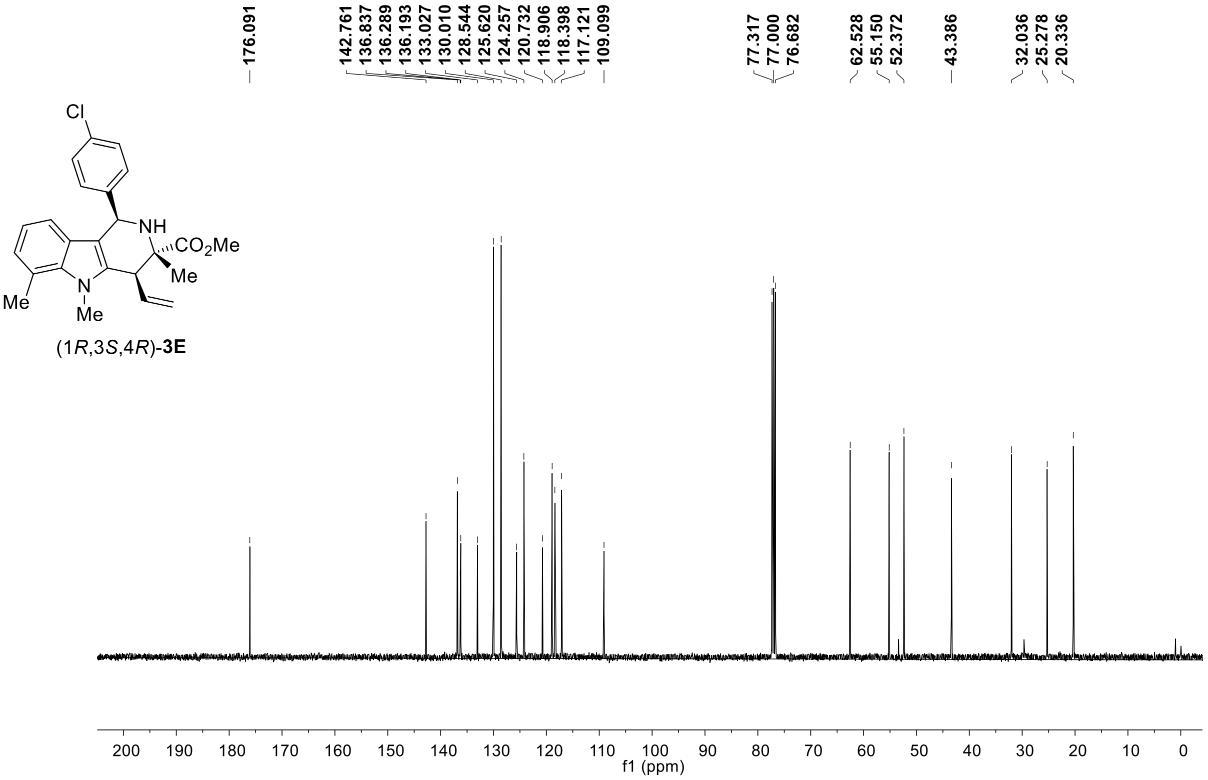

Supplementary Figure 96. ^{13}C NMR spectrum of (1*R*,3*S*,4*R*)-**3E**

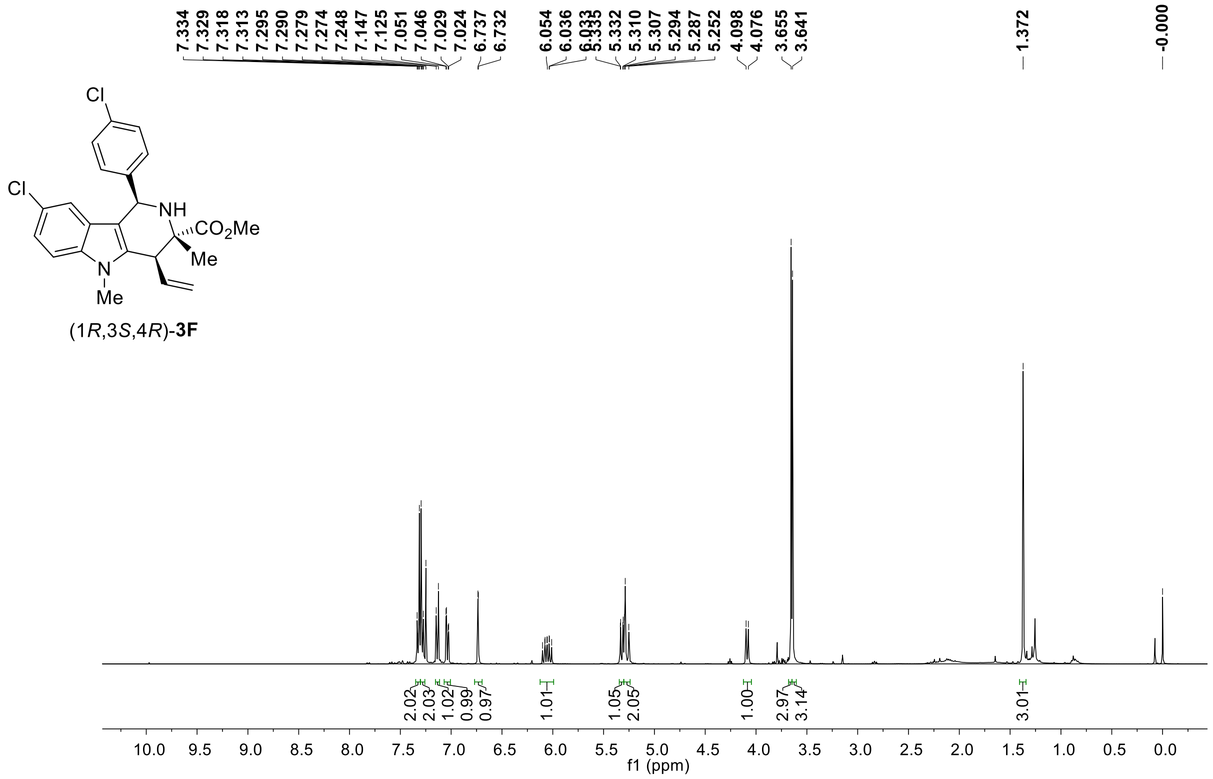

Supplementary Figure 97. ^{1}H NMR spectrum of (1*R*,3*S*,4*R*)-**3F**

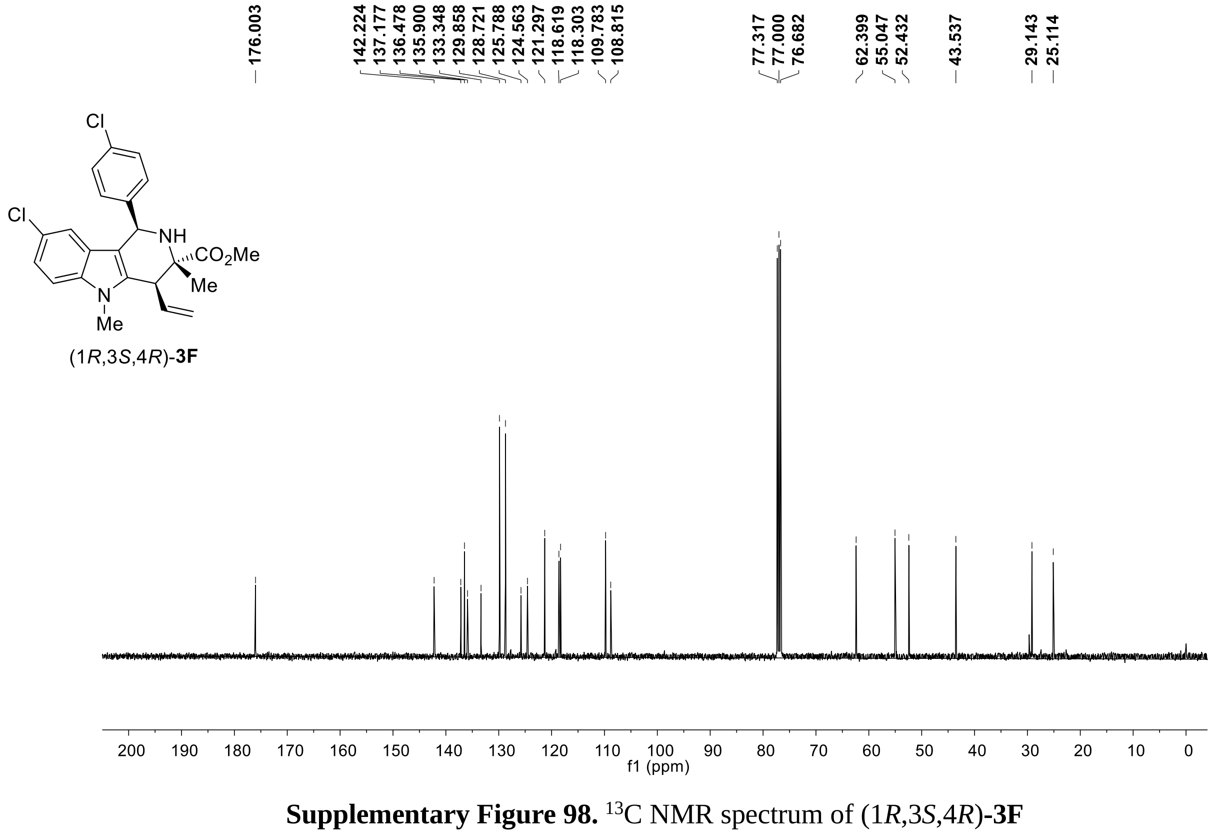

Supplementary Figure 98. ^{13}C NMR spectrum of (1*R*,3*S*,4*R*)-**3F**

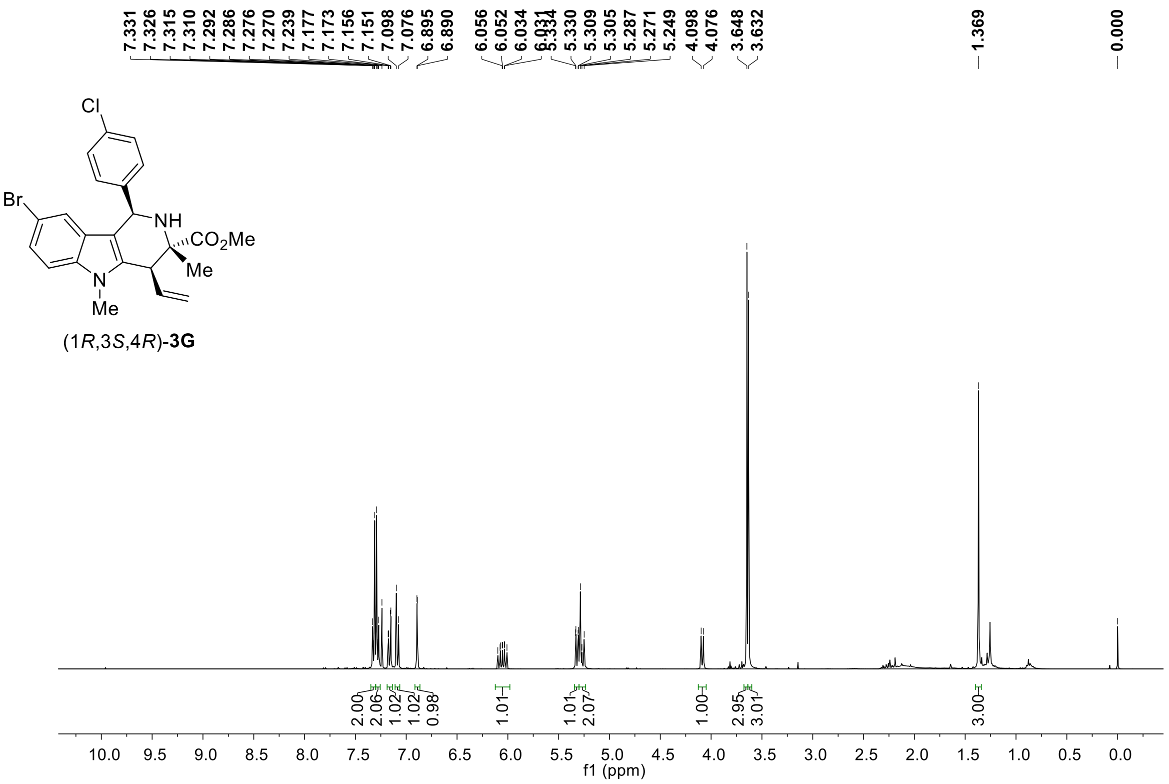

Supplementary Figure 99. ^{1}H NMR spectrum of (1*R*,3*S*,4*R*)-**3G**

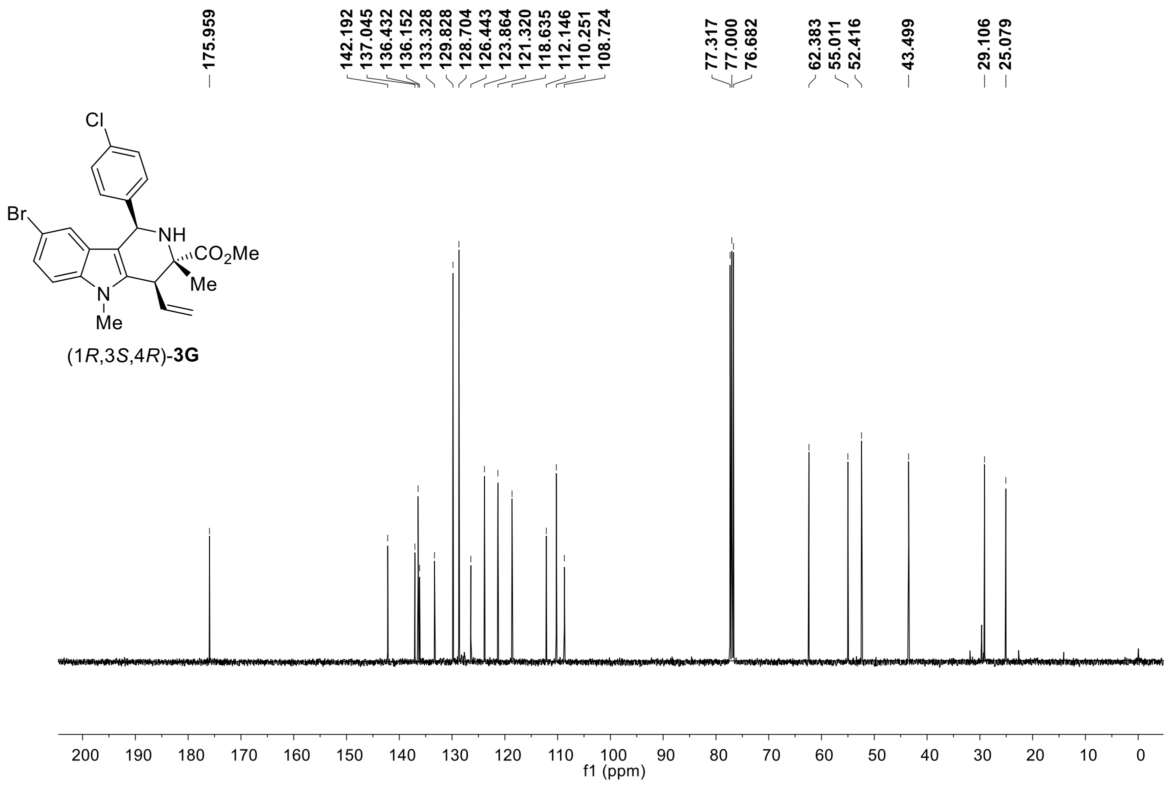

Supplementary Figure 100. ^{13}C NMR spectrum of (1*R*,3*S*,4*R*)-**3G**

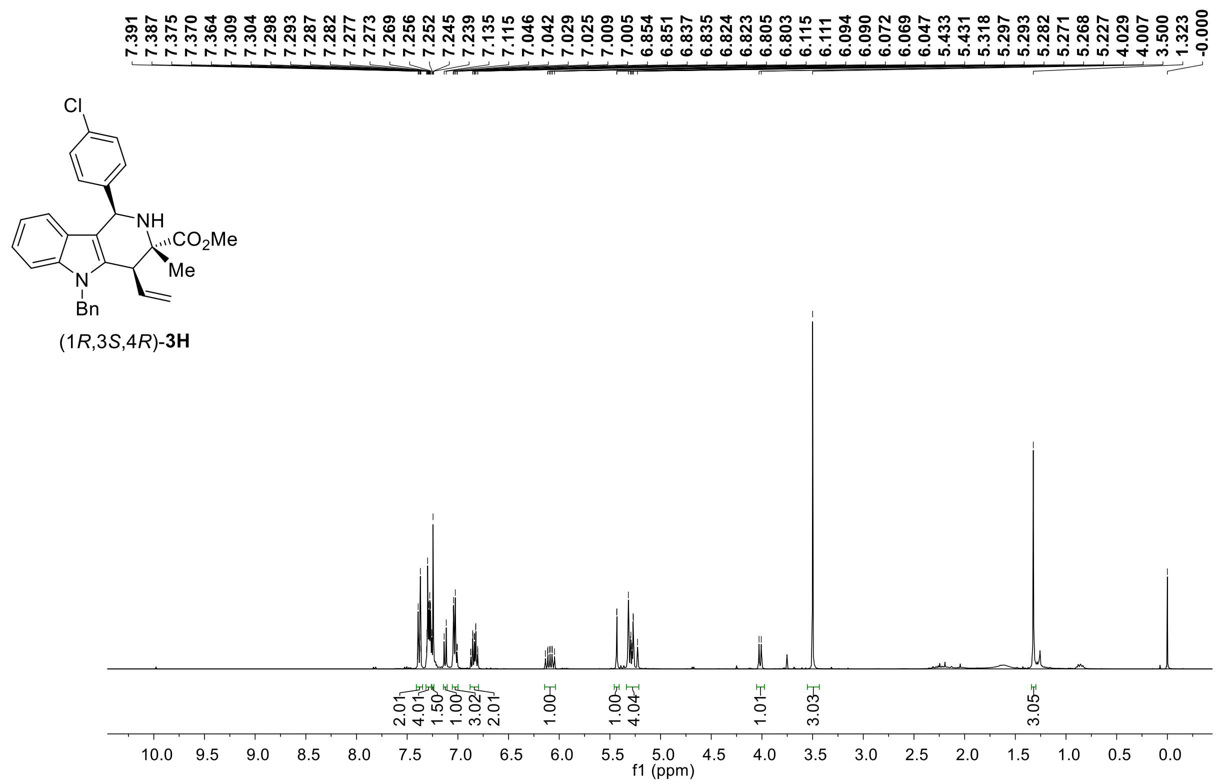

Supplementary Figure 101. ^{1}H NMR spectrum of (1*R*,3*S*,4*R*)**-3H**

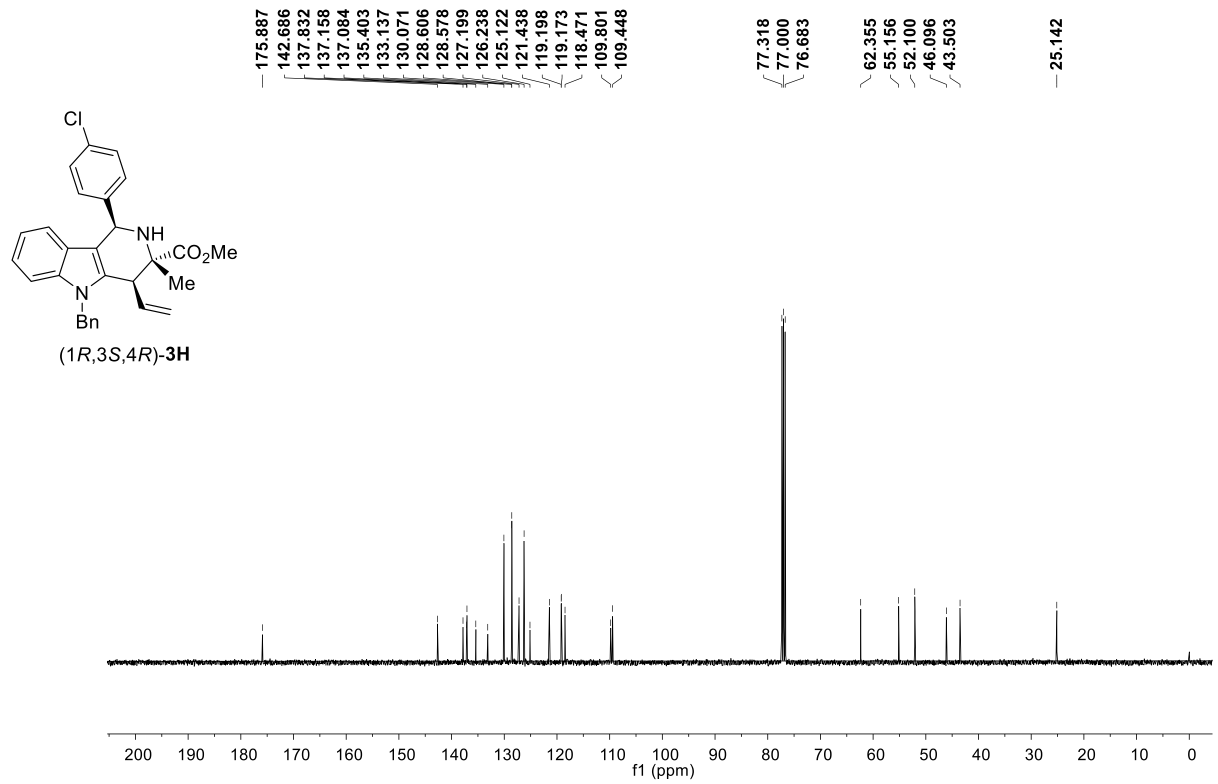

Supplementary Figure 102. ^{13}C NMR spectrum of (1*R*,3*S*,4*R*)-**3H**

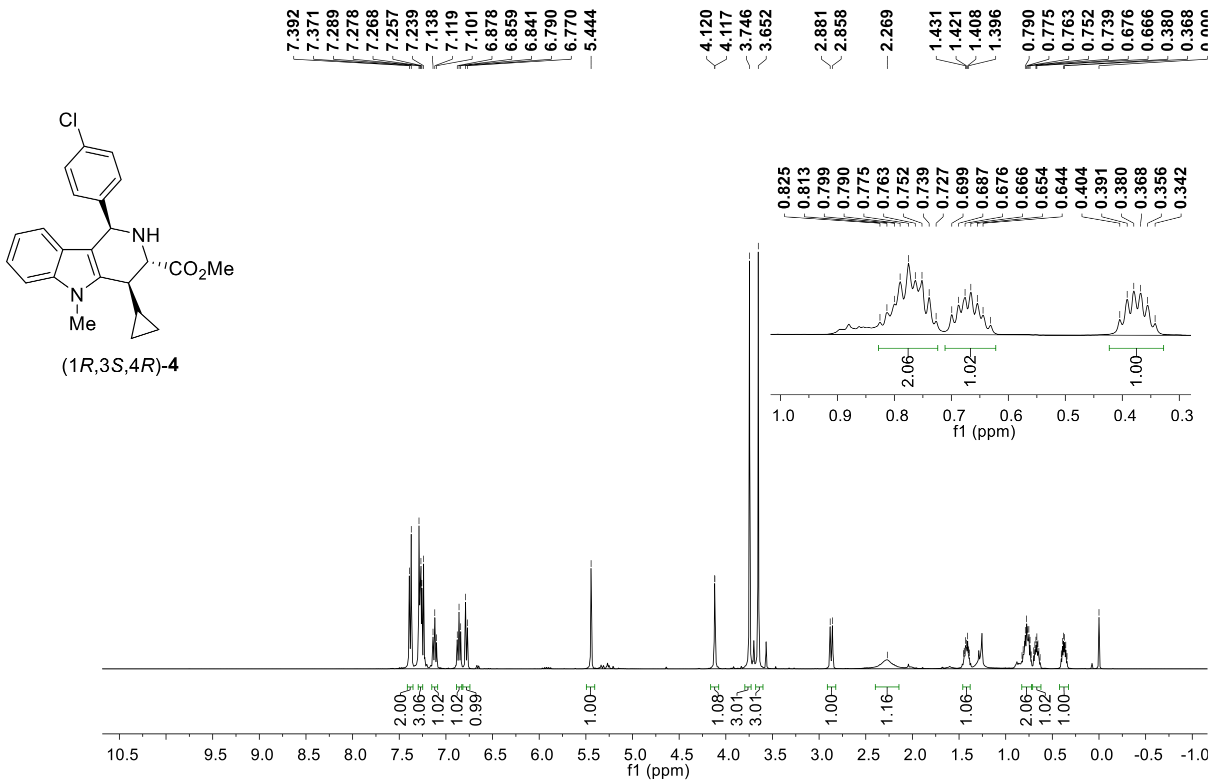

Supplementary Figure 103. ^{1}H NMR spectrum of (1*R*,3*S*,4*R*)-**4**

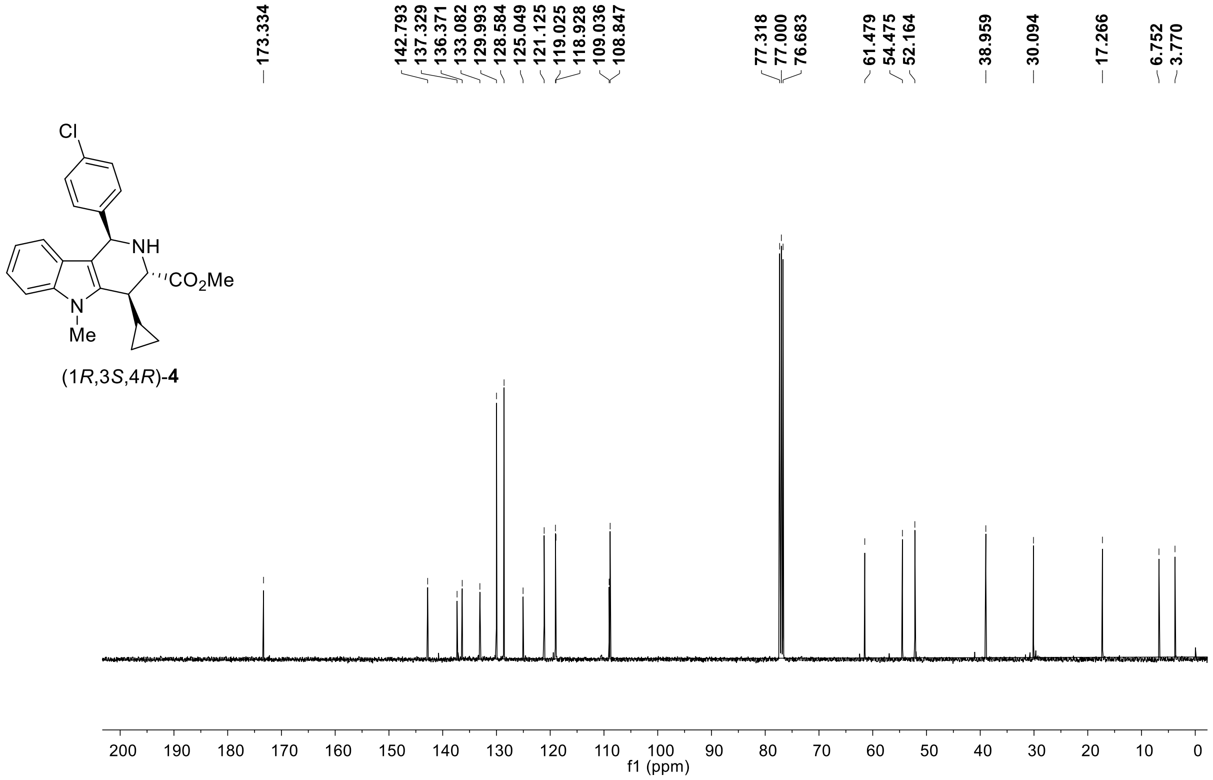

Supplementary Figure 104. ^{13}C NMR spectrum of (1*R*,3*S*,4*R*)-**4**

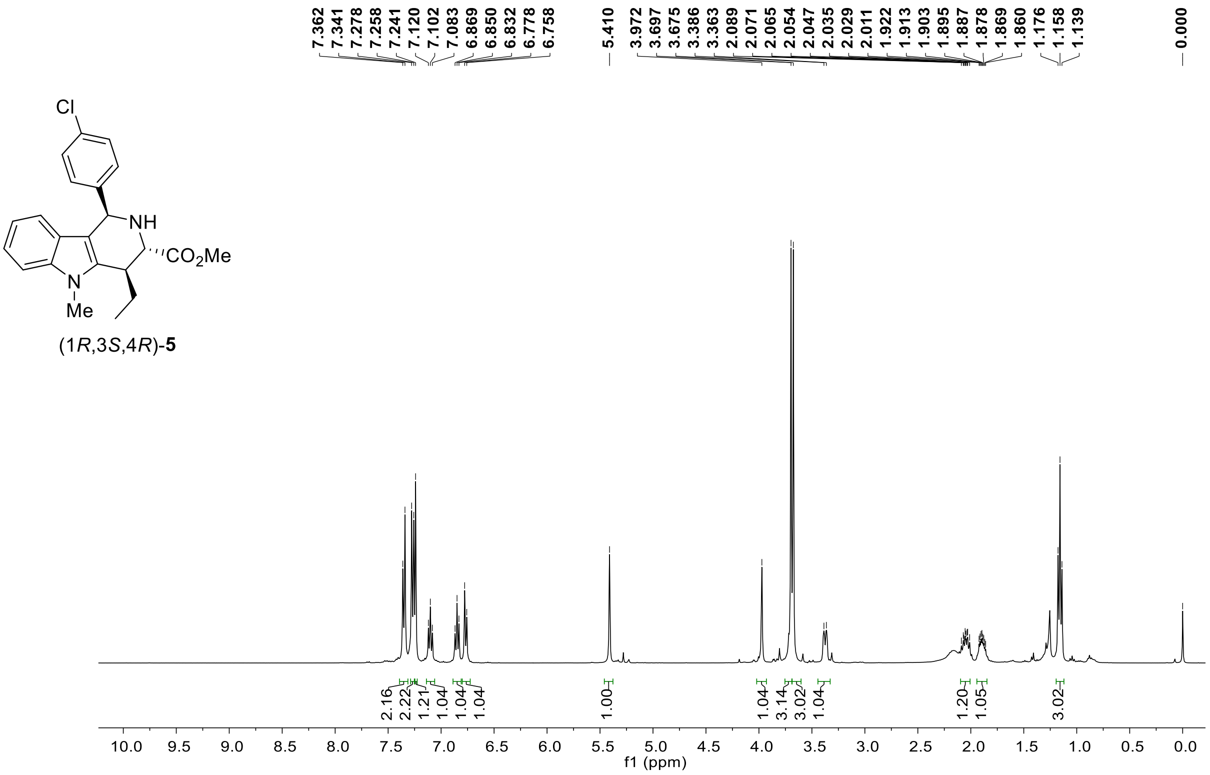

Supplementary Figure 105. ^{1}H NMR spectrum of (1*R*,3*S*,4*R*)-**5**

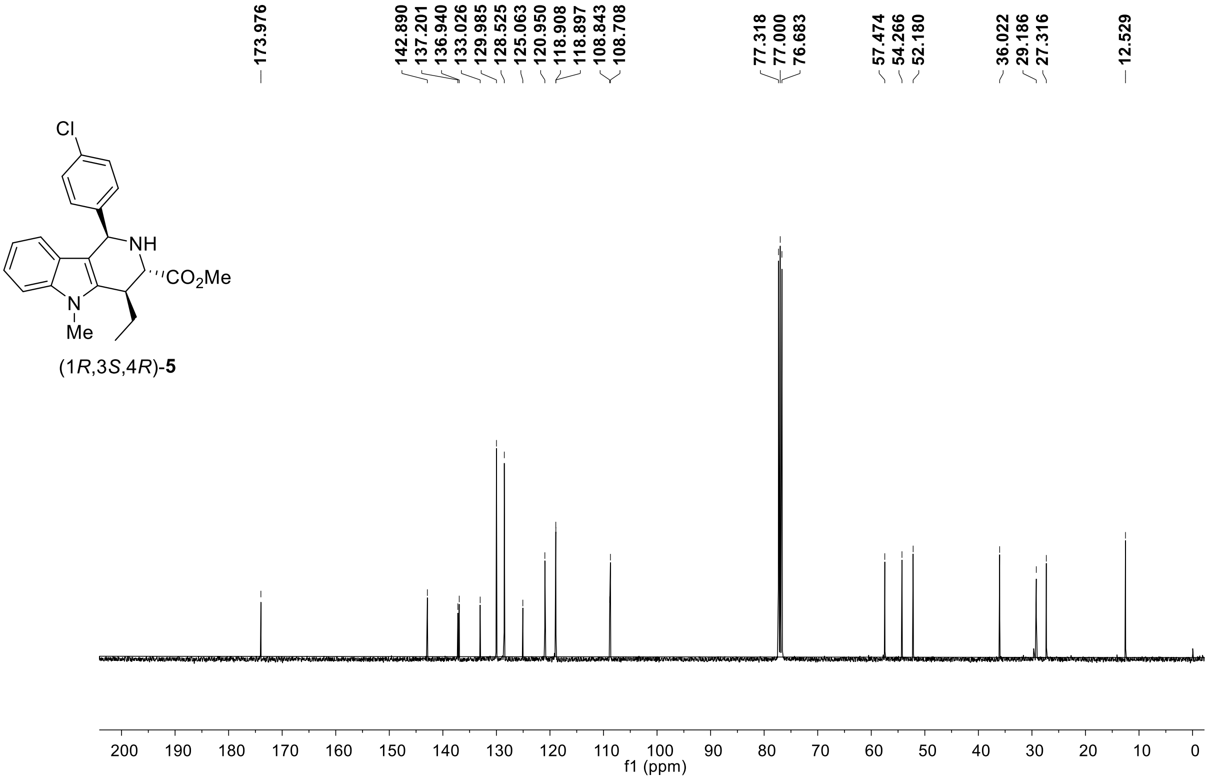

Supplementary Figure 106. ^{13}C NMR spectrum of (1*R*,3*S*,4*R*)-**5**

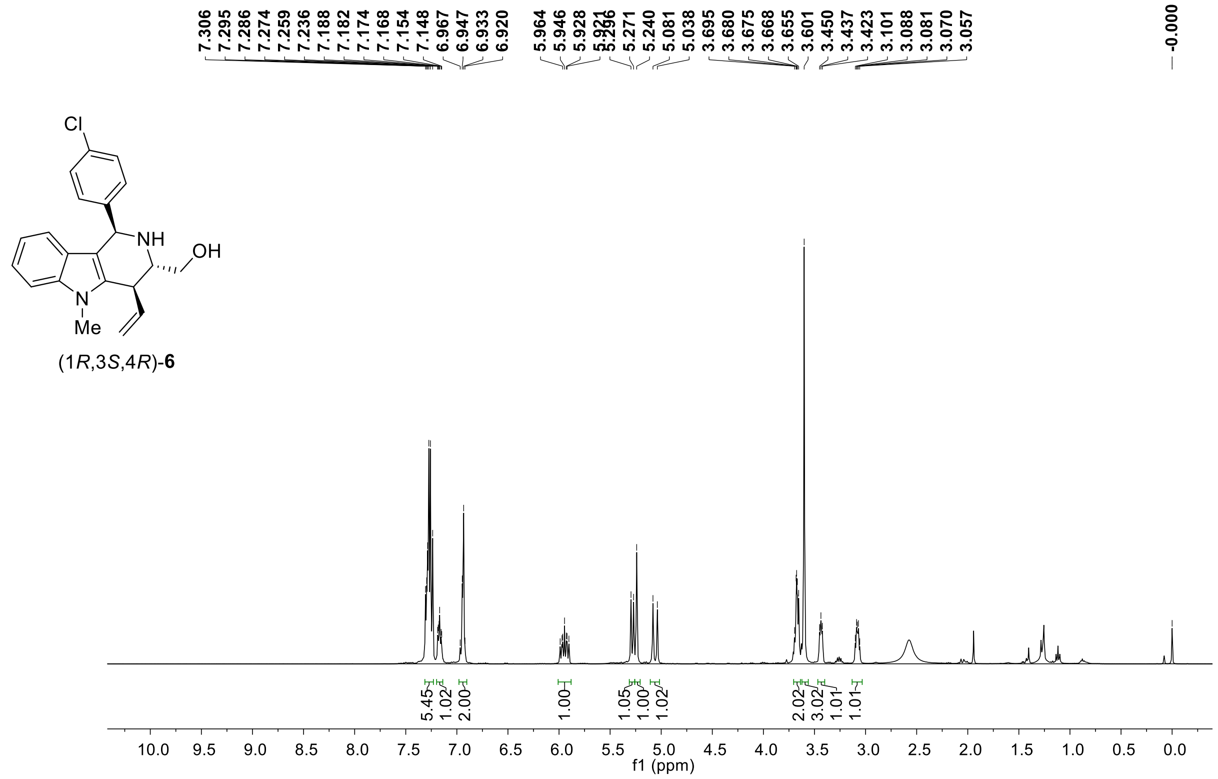

Supplementary Figure 107. ^{1}H NMR spectrum of (1*R*,3*S*,4*R*)-**6**

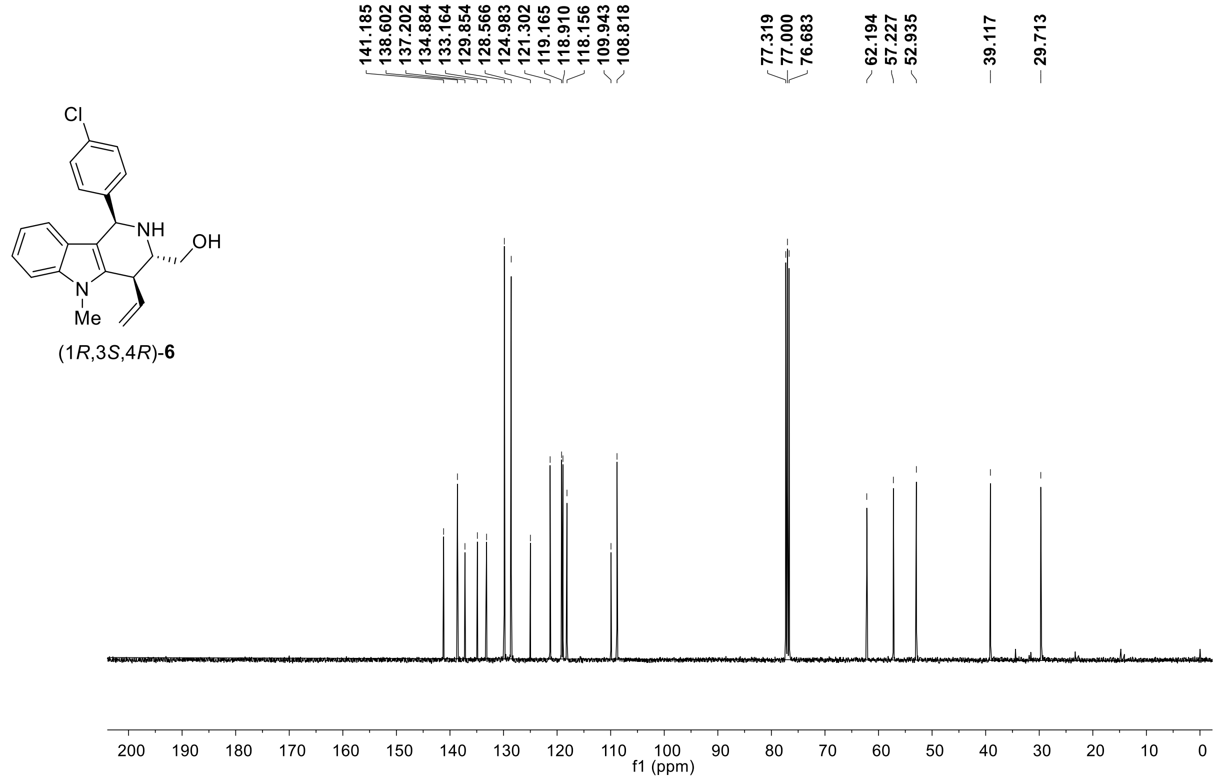

Supplementary Figure 108. ^{13}C NMR spectrum of (1*R*,3*S*,4*R*)-**6**

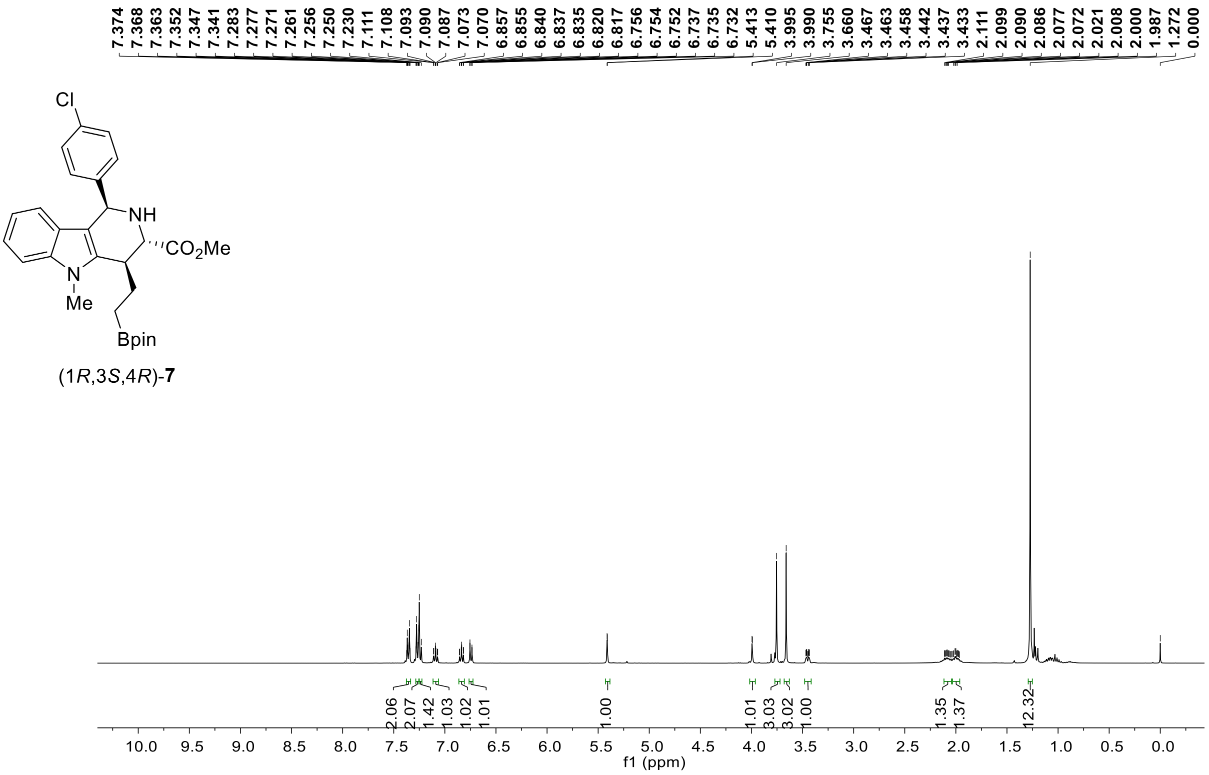

Supplementary Figure 109. ^{1}H NMR spectrum of (1*R*,3*S*,4*R*)-**7**

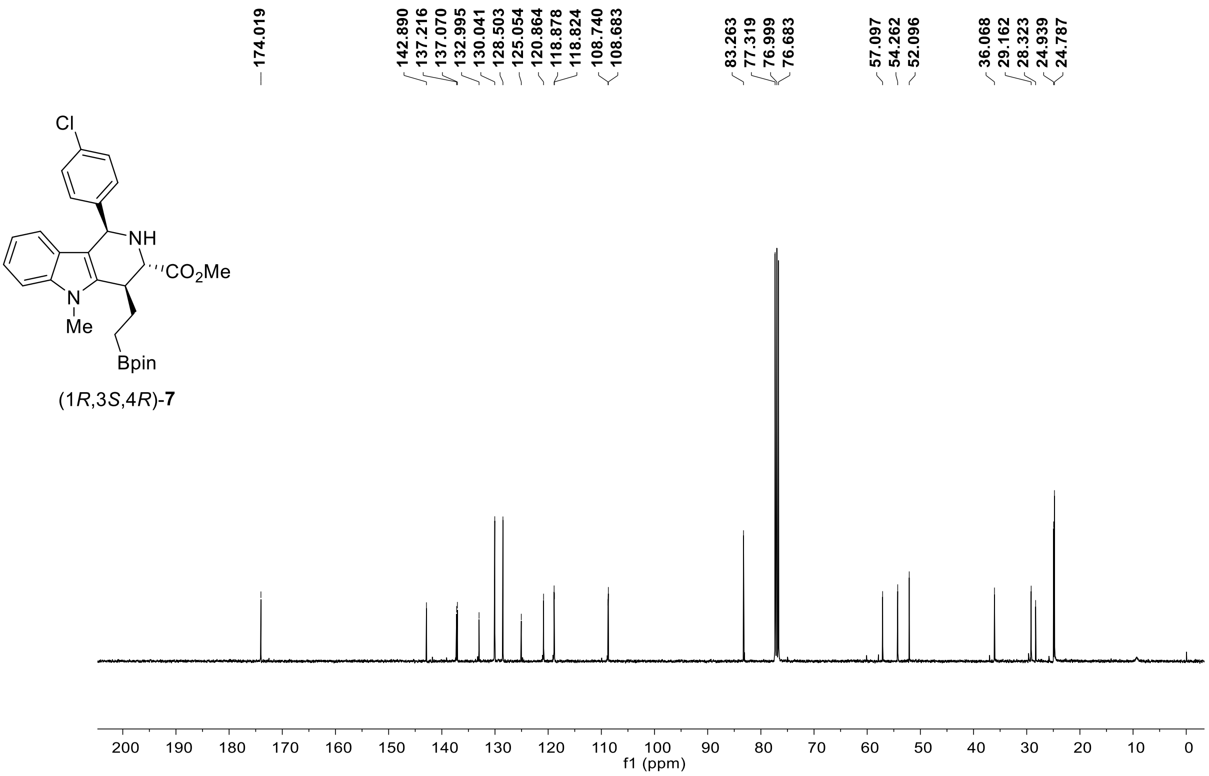

Supplementary Figure 110. ^{1}H NMR spectrum of (1*R*,3*S*,4*R*)-**7**

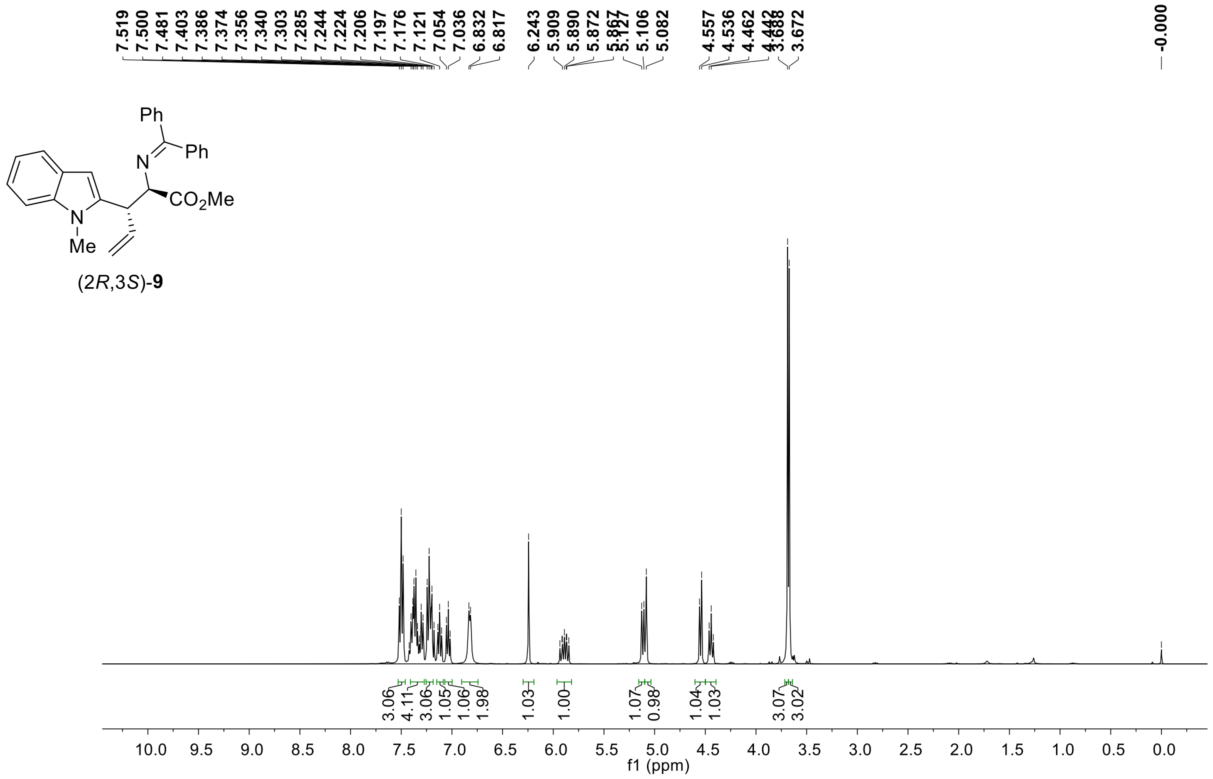

Supplementary Figure 111. ^{1}H NMR spectrum of (2*R*,3*S*)-**9**

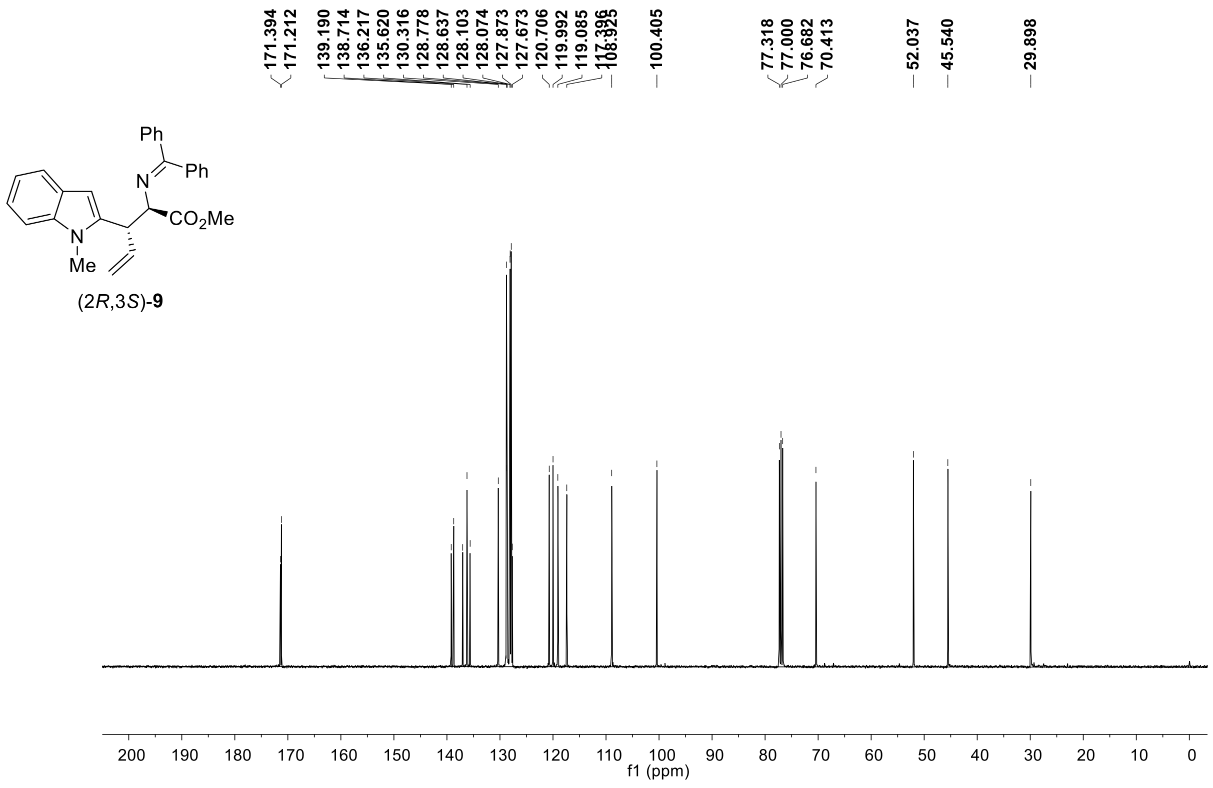

Supplementary Figure 112. ^{13}C NMR spectrum of (2*R*,3*S*)-**9**

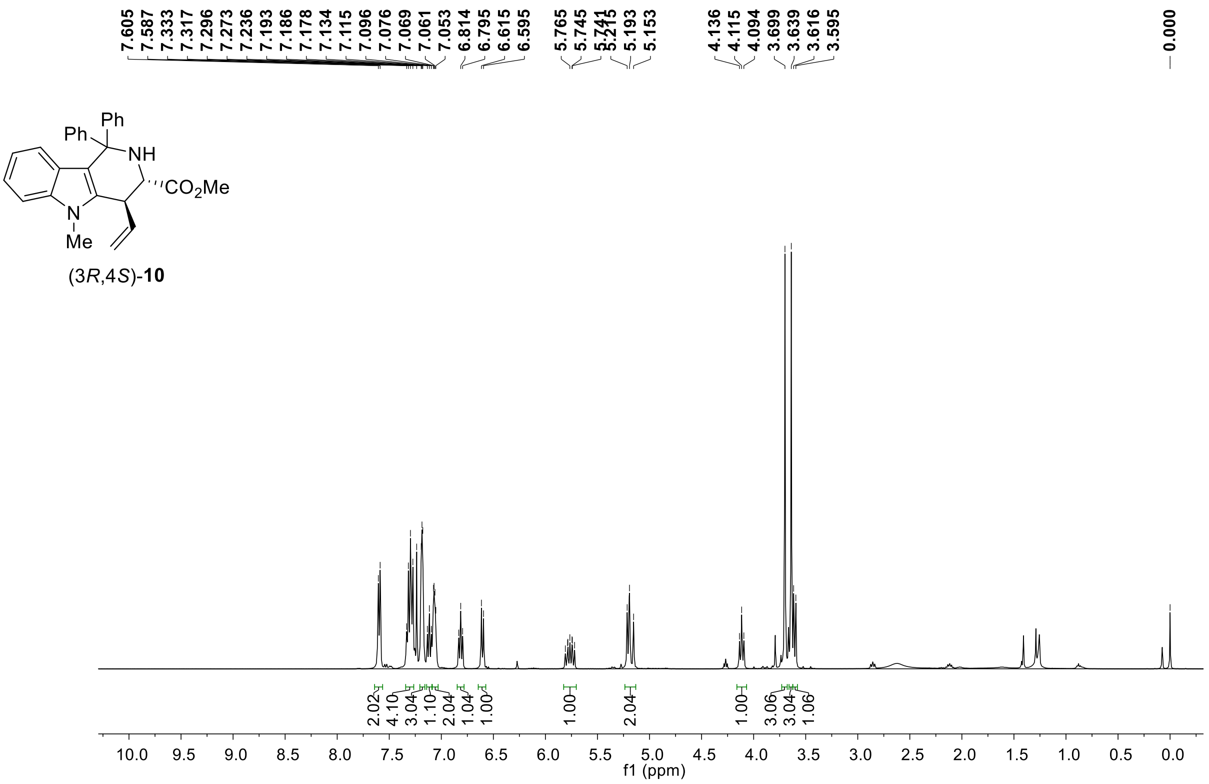

Supplementary Figure 113. ^{1}H NMR spectrum of (3*R*,4*S*)-**10**

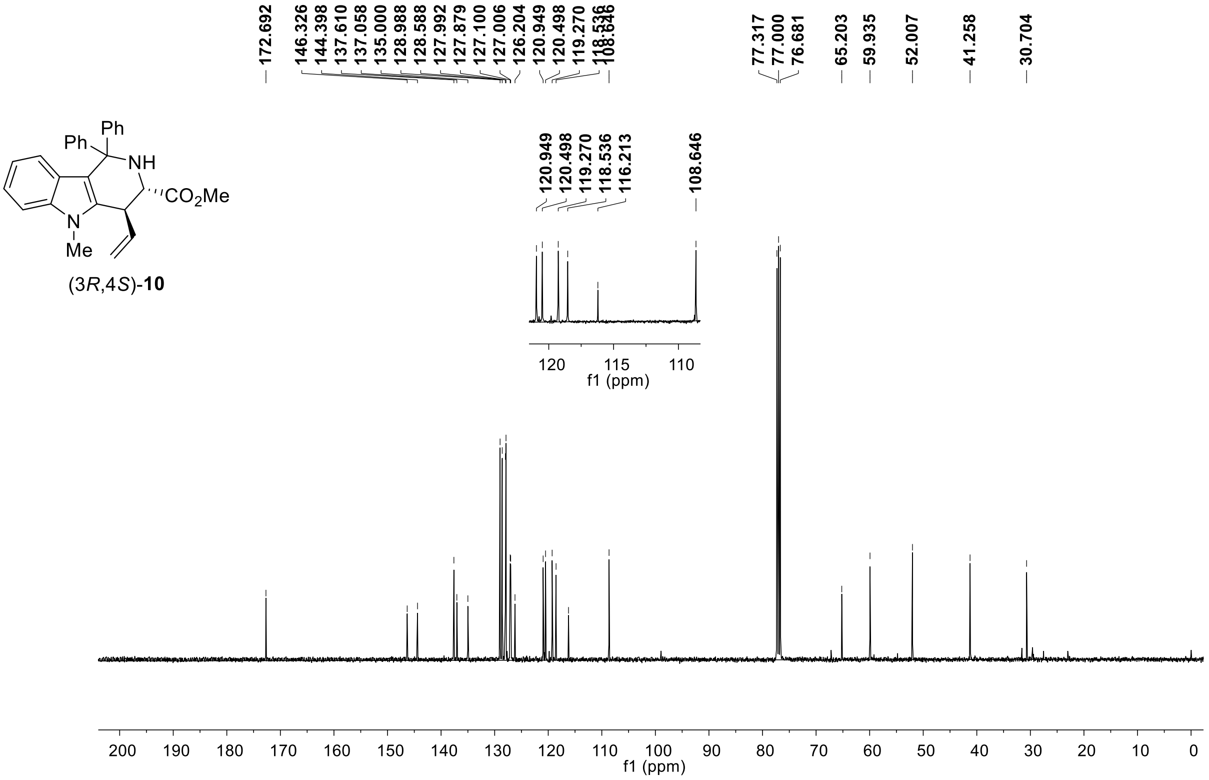

Supplementary Figure 114. ^{13}C NMR spectrum of (3*R*,4*S*)-**10**

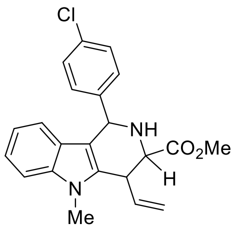

(1R*,3S*,4R*)-**3a** and (1R*,3R*,4R*)-**3a**

Data File E:\DATA\XS...109 03-150 4-Cl-aH div 2018-11-09 11-51-40\XSM 181109 4-Cl-aH div.D
Sample Name: In33 03-132 4-Cl-aH rac 4peak

===

Acq. Operator : SYSTEM Seq. Line : 1
Acq. Instrument : 1260 Location : 70
Injection Date : 11/9/2018 11:53:13 AM Inj : 1
Inj Volume : 5.000 µl
Acq. Method : E:\DATA\XSM-In33\In33 181109 03-150 4-Cl-aH div 2018-11-09 11-51-40\In33 AD -98-40min-282nm 4-Cl-aH div.M
Last changed : 11/9/2018 11:51:40 AM by SYSTEM
Analysis Method : E:\DATA\XSM-In33\In33 181109 03-150 4-Cl-aH div 2018-11-09 11-51-40\In33 AD -98-40min-282nm 4-Cl-aH div.M (Sequence Method)
Last changed : 12/15/2018 4:00:28 PM by SYSTEM
(modified after loading)
Additional Info : Peak(s) manually integrated

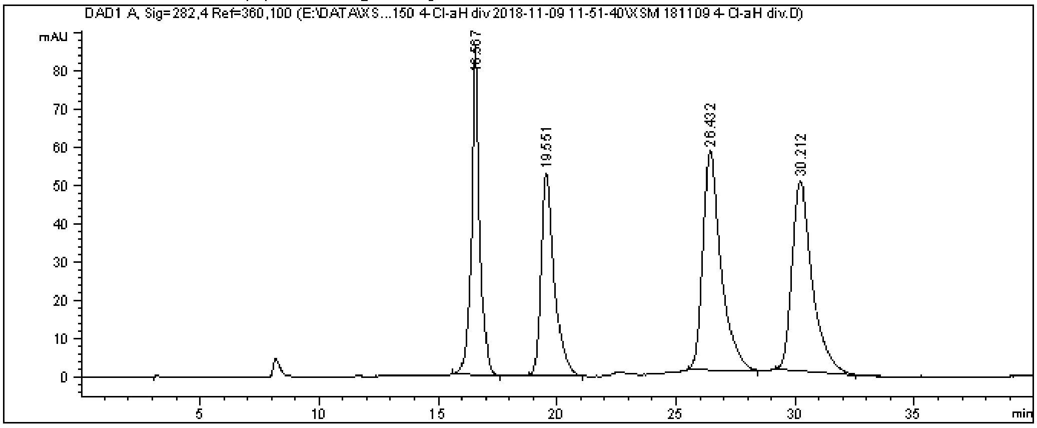

===

Area Percent Report

===

Sorted By : Signal
Multiplier : 1.0000
Dilution : 1.0000
Do not use Multiplier & Dilution Factor with ISTDs

Signal 1: DAD1 A, Sig=282,4 Ref=360,100

Peak #	RetTime [min]	Type	Width [min]	Area [mAU*s]	Height [mAU]	Area %
1	16.567	BB	0.3229	2027.34363	85.74043	20.2577
2	19.551	BB	0.5368	2050.67627	52.83966	20.4908
3	26.432	BB	0.6670	3013.78735	57.26770	30.1145
4	30.212	BB	0.7057	2915.95898	49.61451	29.1370

Totals : 1.00078e4 245.46230

===

*** End of Report ***

1260 12/15/2018 4:00:31 PM SYSTEM Page 1 of 1

Supplementary Figure 115. HPLC spectrum of (1R*,3S*,4R*)-**3a** and (1R*,3R*,4R*)-**3a**

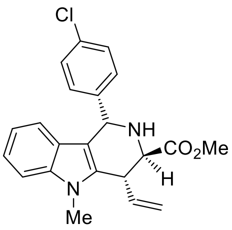

(1*S*,3*R*,4*S*)-**3a**

```
Data File E:\DATA\XS...0316 04-48 4-Cl-aH div 1p and 3p 2019-03-16 20-35-15\XSM 20190316.D
Sample Name: In33 04-48 4-Cl-aH S+D div

    =====================================================================
    Acq. Operator   : SYSTEM                         Seq. Line :    1
    Acq. Instrument : 1260                            Location :   69
    Injection Date  : 3/16/2019 8:36:45 PM                 Inj :    1
                                                  Inj Volume : 5.000 µl
    Acq. Method     : E:\DATA\XSM\In33 190316 04-48 4-Cl-aH div 1p and 3p 2019-03-16 20-35-15
                      \In33 AD-98-40min-282nm 4-Cl-aH div.M
    Last changed    : 3/16/2019 8:35:15 PM by SYSTEM
    Analysis Method : E:\DATA\XSM\In33 190316 04-48 4-Cl-aH div 1p and 3p 2019-03-16 20-35-15
                      \In33 AD-98-40min-282nm 4-Cl-aH div.M (Sequence Method)
    Last changed    : 3/16/2019 10:23:57 PM by SYSTEM
                      (modified after loading)
    Additional Info : Peak(s) manually integrated
```

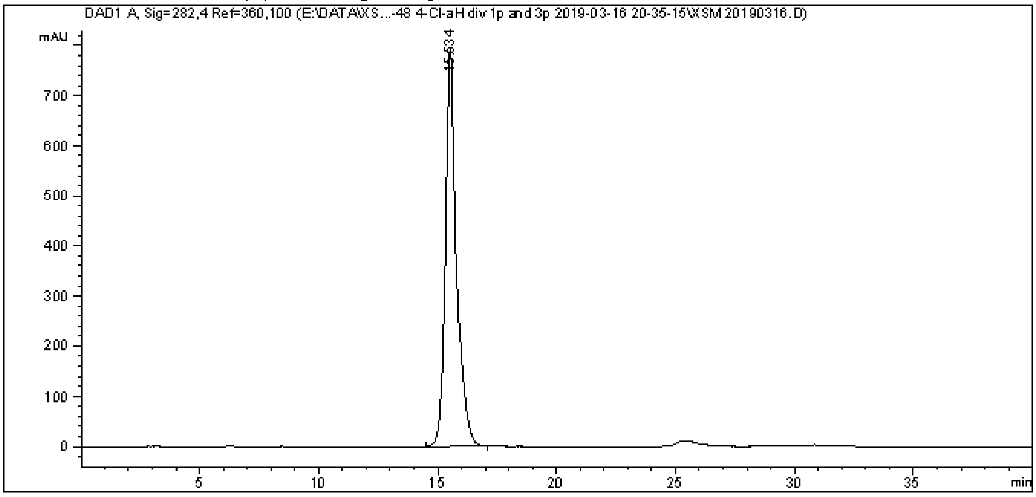


```
    =====================================================================
                             Area Percent Report
    =====================================================================

    Sorted By             :      Signal
    Multiplier            :      1.0000
    Dilution              :      1.0000
    Do not use Multiplier & Dilution Factor with ISTDs


    Signal 1: DAD1 A, Sig=282,4 Ref=360,100

    Peak RetTime Type  Width     Area      Height     Area
      #   [min]        [min]   [mAU*s]     [mAU]        %
    ----|-------|----|-------|----------|----------|--------|
       1  15.534 BB    0.4477 2.55505e4   791.22150 100.0000

    Totals :                  2.55505e4   791.22150


    =====================================================================
                              *** End of Report ***

1260 3/16/2019 10:24:23 PM SYSTEM                                    Page 1 of 1
```

Supplementary Figure 116. HPLC spectrum of (1*S*,3*R*,4*S*)-**3a**

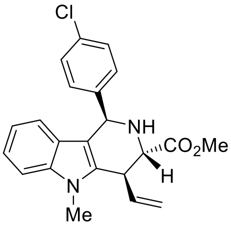

(1*R*,3*S*,4*R*)-**3a**

Data File E:\DATA\XS...09 03-150 4-Cl-aH div 2018-11-09 11-51-40\XSM 181109 4-Cl-aH div1.D
Sample Name: In33 03-146 4-Cl-aH R+L opt

===

Acq. Operator : SYSTEM Seq. Line : 2
Acq. Instrument : 1260 Location : 66
Injection Date : 11/9/2018 12:34:43 PM Inj : 1
Inj Volume : 5.000 µl
Acq. Method : E:\DATA\XSM-In33\In33 181109 03-150 4-Cl-aH div 2018-11-09 11-51-40\In33 AD -98-40min-282nm 4-Cl-aH div.M
Last changed : 11/9/2018 11:51:40 AM by SYSTEM
Analysis Method : E:\DATA\XSM-In33\In33 181109 03-150 4-Cl-aH div 2018-11-09 11-51-40\In33 AD -98-40min-282nm 4-Cl-aH div.M (Sequence Method)
Last changed : 1/2/2019 4:46:24 PM by SYSTEM
(modified after loading)

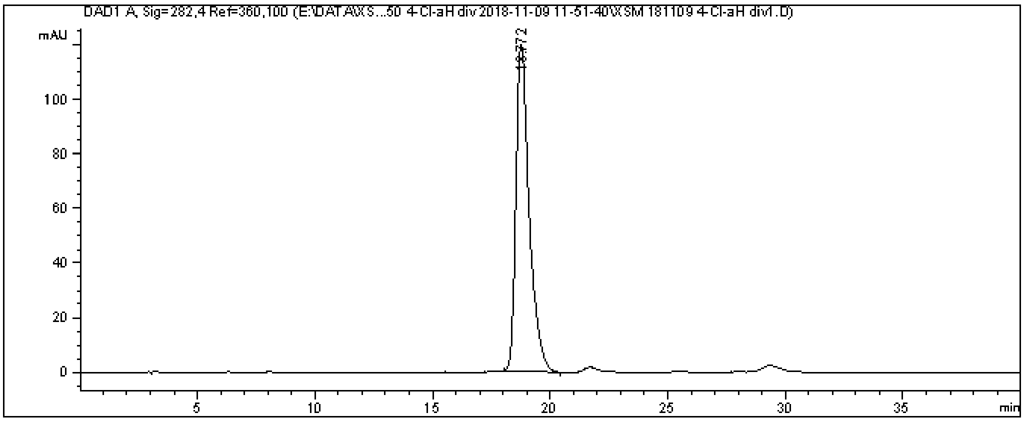

===

Area Percent Report

===

Sorted By : Signal
Multiplier : 1.0000
Dilution : 1.0000
Do not use Multiplier & Dilution Factor with ISTDs

Signal 1: DAD1 A, Sig=282,4 Ref=360,100

Peak #	RetTime [min]	Type	Width [min]	Area [mAU*s]	Height [mAU]	Area %
1	18.772	BB	0.5548	4565.13135	119.72671	100.0000
Totals :				4565.13135	119.72671	

===

*** End of Report ***

1260 1/2/2019 4:50:48 PM SYSTEM Page 1 of 1

Supplementary Figure 117. HPLC spectrum of (1*R*,3*S*,4*R*)-**3a**

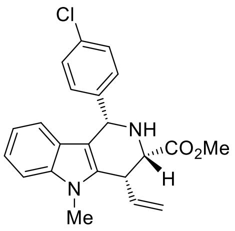

(1*S*,3*S*,4*S*)-**3a**

```
Data File E:\DATA\XS...316 04-48 4-Cl-aH div 1p and 3p 2019-03-16 20-35-15\XSM 201903161.D
Sample Name: In33 04-48 4-Cl-aH S+L div

    =====================================================================
    Acq. Operator   : SYSTEM                          Seq. Line :   2
    Acq. Instrument : 1260                             Location :   70
    Injection Date  : 3/16/2019 9:18:12 PM                  Inj :   1
                                                    Inj Volume : 5.000 µl
    Acq. Method     : E:\DATA\XSM\In33 190316 04-48 4-Cl-aH div 1p and 3p 2019-03-16 20-35-15
                      \In33 AD-98-40min-282nm 4-Cl-aH div.M
    Last changed    : 3/16/2019 8:35:15 PM by SYSTEM
    Analysis Method : E:\DATA\XSM\In33 190316 04-48 4-Cl-aH div 1p and 3p 2019-03-16 20-35-15
                      \In33 AD-98-40min-282nm 4-Cl-aH div.M (Sequence Method)
    Last changed    : 3/16/2019 10:23:57 PM by SYSTEM
                      (modified after loading)
    Additional Info : Peak(s) manually integrated
```

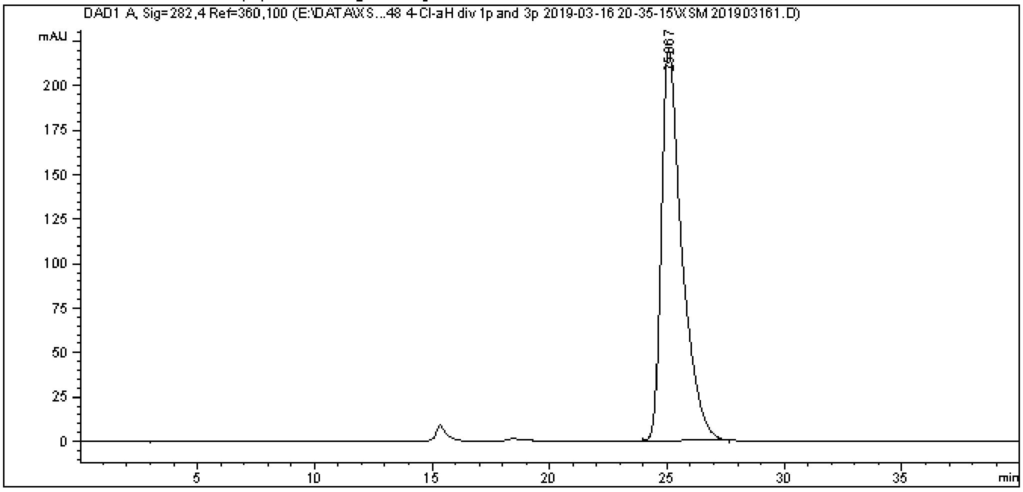


```
    =====================================================================
                             Area Percent Report
    =====================================================================

    Sorted By             :      Signal
    Multiplier            :      1.0000
    Dilution              :      1.0000
    Do not use Multiplier & Dilution Factor with ISTDs


    Signal 1: DAD1 A, Sig=282,4 Ref=360,100

    Peak RetTime Type  Width     Area      Height     Area
      #   [min]        [min]   [mAU*s]     [mAU]        %
    ----|-------|----|-------|----------|----------|--------|
       1  25.067 BB    0.7977 1.34526e4   220.50945 100.0000

    Totals :                  1.34526e4   220.50945


    =====================================================================
                              *** End of Report ***

1260 3/16/2019 10:24:02 PM SYSTEM                                        Page 1 of 1
```

Supplementary Figure 118. HPLC spectrum of (1*S*,3*S*,4*S*)-**3a**

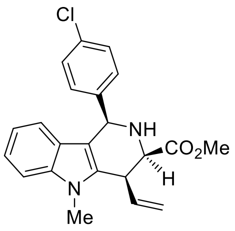

(1*R*,3*R*,4*R*)-**3a**

```
Data File E:\DATA\XS...0110 03-04 4-Cl 4-Cl-aH 23Me-aH 2019-01-10 22-19-30\XSM 201901103.D
Sample Name: In33 03-150 4-Cl-aH R+D opt

    =====================================================================
    Acq. Operator   : SYSTEM                        Seq. Line :    4
    Acq. Instrument : 1260                           Location :   60
    Injection Date  : 1/10/2019 11:39:18 PM               Inj :    1
                                                   Inj Volume : 5.000 µl
    Acq. Method     : E:\DATA\XSM\In33 190110 03-04 4-Cl 4-Cl-aH 23Me-aH 2019-01-10 22-19-30\In33
                      AD-98-40min-282nm 4-Cl-aH div.M
    Last changed    : 1/10/2019 10:19:31 PM by SYSTEM
    Analysis Method : E:\DATA\XSM\In33 190110 03-04 4-Cl 4-Cl-aH 23Me-aH 2019-01-10 22-19-30\In33
                      AD-98-40min-282nm 4-Cl-aH div.M (Sequence Method)
    Last changed    : 1/11/2019 11:57:28 AM by SYSTEM
                      (modified after loading)
    Additional Info : Peak(s) manually integrated
```

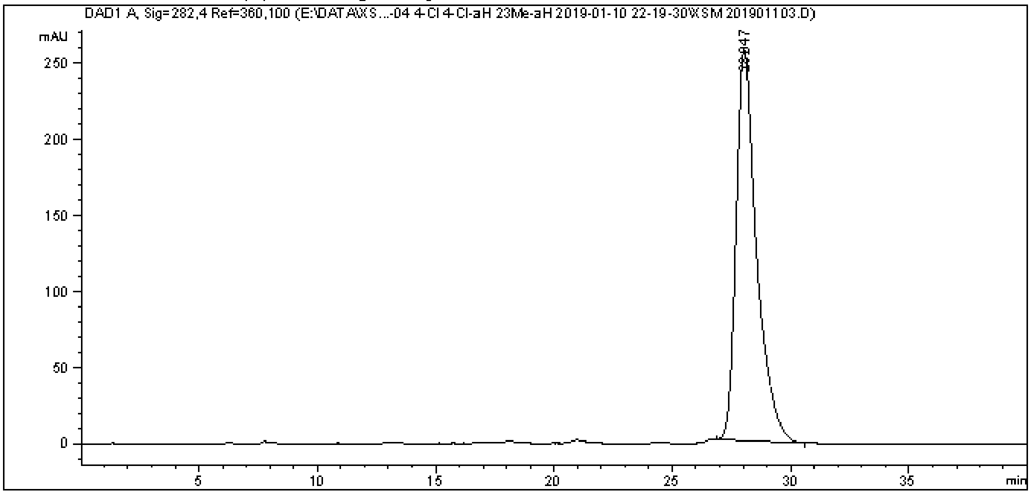


```
    =====================================================================
                             Area Percent Report
    =====================================================================

    Sorted By             :      Signal
    Multiplier            :      1.0000
    Dilution              :      1.0000
    Do not use Multiplier & Dilution Factor with ISTDs


    Signal 1: DAD1 A, Sig=282,4 Ref=360,100

    Peak RetTime Type  Width     Area      Height     Area
      #   [min]        [min]   [mAU*s]     [mAU]        %
    ----|-------|----|-------|----------|----------|--------|
       1  28.047 BV R  0.8224 1.53992e4   257.24072 100.0000

    Totals :                  1.53992e4   257.24072


    =====================================================================
                             *** End of Report ***
```


Supplementary Figure 119. HPLC spectrum of (1*R*,3*R*,4*R*)-**3a**

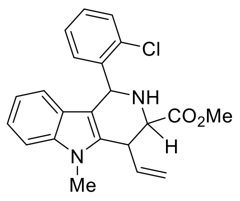

(1*R**,3*S**,4*R**)-**3b**

Data File E:\DATA\XS...230 04-12 2Cl-23Me-5Mein-aH 1st 2018-12-30 17-30-14\XSM 201812303.D
Sample Name: In33 04-12 2-Cl-aH 1st rac

```
=====================================================================
Acq. Operator   : SYSTEM                         Seq. Line :   4
Acq. Instrument : 1260                            Location :   70
Injection Date  : 12/30/2018 7:04:04 PM                Inj :   1
                                                Inj Volume : 5.000 µl
Acq. Method     : E:\DATA\XSM\In33 181230 04-12 2Cl-23Me-5Mein-aH 1st 2018-12-30 17-30-14
                  \In33 AD-98-234nm-30min 2Cl-23Me-aH.M
Last changed    : 12/30/2018 5:30:15 PM by SYSTEM
Analysis Method : E:\DATA\XSM\In33 181230 04-12 2Cl-23Me-5Mein-aH 1st 2018-12-30 17-30-14
                  \In33 AD-98-234nm-30min 2Cl-23Me-aH.M (Sequence Method)
Last changed    : 1/2/2019 11:08:05 AM by SYSTEM
                  (modified after loading)
Additional Info : Peak(s) manually integrated
```

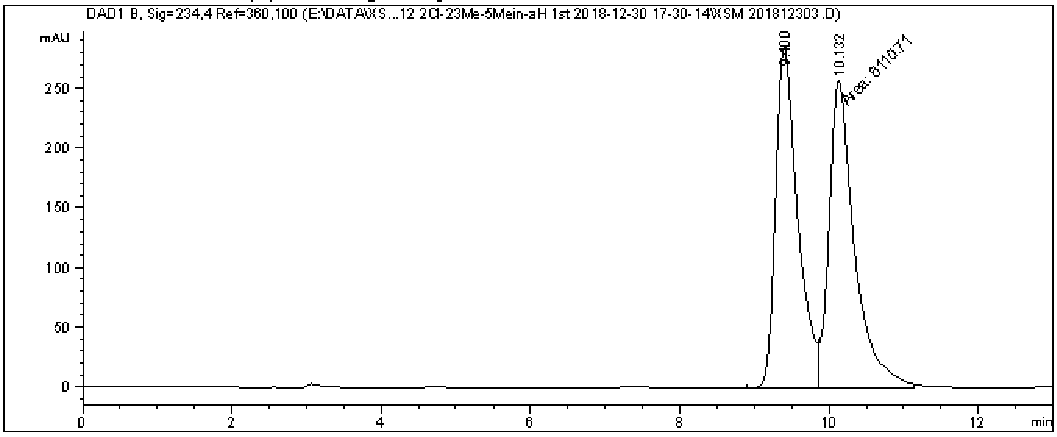


```
=====================================================================
                         Area Percent Report
=====================================================================

Sorted By             :      Signal
Multiplier            :      1.0000
Dilution              :      1.0000
Do not use Multiplier & Dilution Factor with ISTDs


Signal 1: DAD1 B, Sig=234,4 Ref=360,100

Peak RetTime Type  Width     Area      Height     Area
  #   [min]        [min]   [mAU*s]     [mAU]        %
----|-------|----|-------|----------|----------|--------|
   1   9.400 BV    0.3089 5894.23633  285.41483  49.0984
   2  10.132 MF    0.3953 6110.70654  257.61252  50.9016

Totals :                 1.20049e4   543.02734


=====================================================================
                          *** End of Report ***
```

1260 1/2/2019 11:08:09 AM SYSTEM Page 1 of 1

Supplementary Figure 120. HPLC spectrum of (1*R**,3*S**,4*R**)-**3b**

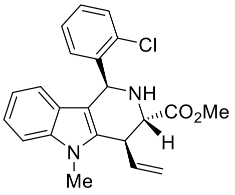

(1*R*,3*S*,4*R*)-**3b**

```
Data File E:\DATA\XS...230 04-12 2Cl-23Me-5Mein-aH 1st 2018-12-30 17-30-14\XSM 201812302.D
Sample Name: In33 04-12 2-Cl-aH R+L opt

=====================================================================
Acq. Operator   : SYSTEM                        Seq. Line :   3
Acq. Instrument : 1260                           Location :   69
Injection Date  : 12/30/2018 6:33:08 PM               Inj :   1
                                               Inj Volume : 5.000 µl
Acq. Method     : E:\DATA\XSM\In33 181230 04-12 2Cl-23Me-5Mein-aH 1st 2018-12-30 17-30-14
                  \In33 AD-98-234nm-30min 2Cl-23Me-aH.M
Last changed    : 12/30/2018 5:30:15 PM by SYSTEM
Analysis Method : E:\DATA\XSM\In33 181230 04-12 2Cl-23Me-5Mein-aH 1st 2018-12-30 17-30-14
                  \In33 AD-98-234nm-30min 2Cl-23Me-aH.M (Sequence Method)
Last changed    : 1/2/2019 11:08:05 AM by SYSTEM
                  (modified after loading)
Additional Info : Peak(s) manually integrated
```

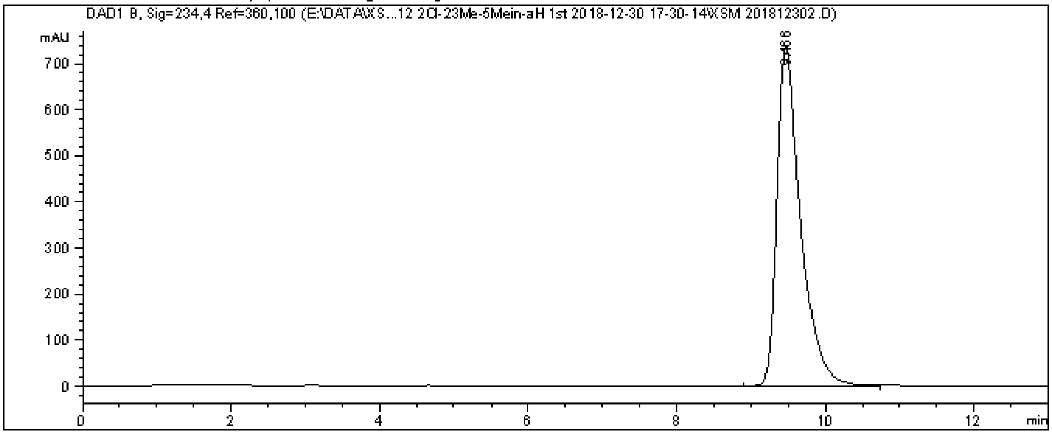


```
=====================================================================
                         Area Percent Report
=====================================================================

Sorted By             :      Signal
Multiplier            :      1.0000
Dilution              :      1.0000
Do not use Multiplier & Dilution Factor with ISTDs


Signal 1: DAD1 B, Sig=234,4 Ref=360,100

Peak RetTime Type  Width     Area      Height     Area
  #   [min]        [min]   [mAU*s]     [mAU]        %
----|-------|----|-------|----------|----------|--------|
   1   9.466 BB    0.3260 1.62327e4  734.15625 100.0000

Totals :                  1.62327e4  734.15625


=====================================================================
                          *** End of Report ***
```


Supplementary Figure 121. HPLC spectrum of (1*R*,3*S*,4*R*)-**3b**

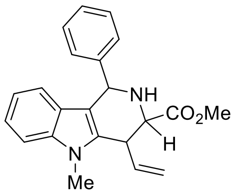

(1*R**,3*S**,4*R**)-**3c** and (1*R**,3*R**,4*R**)-**3c**

```
Data File E:\DATA\XS...3 181123 03-150 XSM Ph-aH 4p div 2018-11-17 20-06-32\XSM 20181123.D
Sample Name: In33 03-151 Ph-aH rac 4peak

    =====================================================================
    Acq. Operator   : SYSTEM                        Seq. Line :   1
    Acq. Instrument : 1260                           Location :   70
    Injection Date  : 11/17/2018 8:08:06 PM               Inj :   1
                                                   Inj Volume : 5.000 µl
    Acq. Method     : E:\DATA\XSM-In33\In33 181123 03-150 XSM Ph-aH 4p div 2018-11-17 20-06-32
                      \In33 1st AD-98-40min-232nm Ph-aH 4p.M
    Last changed    : 11/17/2018 8:06:32 PM by SYSTEM
    Analysis Method : E:\DATA\XSM-In33\In33 181123 03-150 XSM Ph-aH 4p div 2018-11-17 20-06-32
                      \In33 1st AD-98-40min-232nm Ph-aH 4p.M (Sequence Method)
    Last changed    : 12/15/2018 4:06:38 PM by SYSTEM
                      (modified after loading)
    Additional Info : Peak(s) manually integrated
```

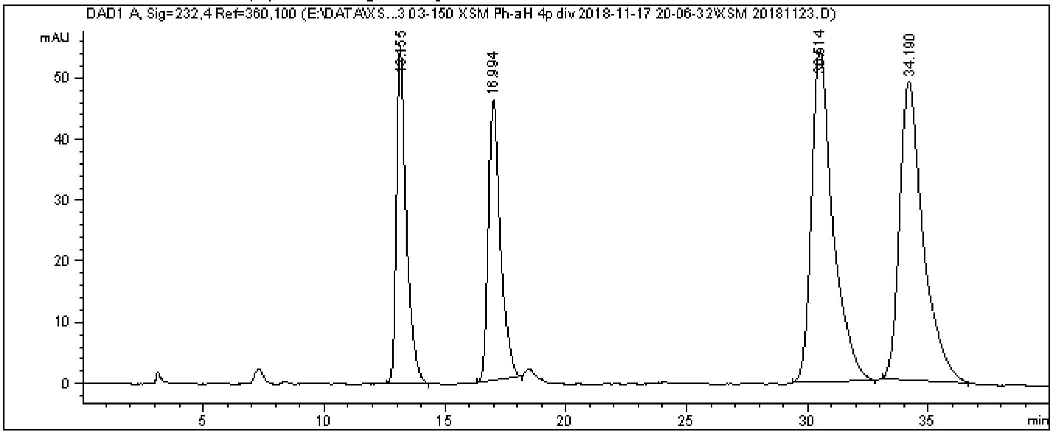


```
    =====================================================================
                             Area Percent Report
    =====================================================================

    Sorted By             :      Signal
    Multiplier            :      1.0000
    Dilution              :      1.0000
    Do not use Multiplier & Dilution Factor with ISTDs


    Signal 1: DAD1 A, Sig=232,4 Ref=360,100

    Peak RetTime Type  Width     Area      Height     Area
      #   [min]        [min]   [mAU*s]     [mAU]        %
    ----|-------|----|-------|----------|----------|--------|
       1  13.155 BB    0.4108 1564.10522   54.99319  15.9201
       2  16.994 BB    0.4881 1607.36499   45.83415  16.3604
       3  30.514 BB    0.7361 3354.70581   54.20038  34.1455
       4  34.190 BB    0.7916 3298.55615   48.92328  33.5740

    Totals :                  9824.73218  203.95100


    =====================================================================
                             *** End of Report ***

1260 12/15/2018 4:06:42 PM SYSTEM                                    Page 1 of 1
```

Supplementary Figure 122. HPLC spectrum of (1*R**,3*S**,4*R**)-**3c** and (1*R**,3*R**,4*R**)-**3c**

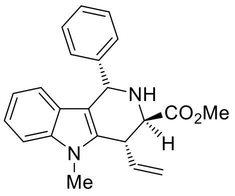

(1*S*,3*R*,4*S*)-**3c**

```
Data File E:\DATA\XS... 181123 03-150 XSM Ph-aH 4p div 2018-11-17 20-06-32\XSM 201811231.D
Sample Name: In33 03-150 Ph-aH S+D opt

    =====================================================================
    Acq. Operator   : SYSTEM                        Seq. Line :   2
    Acq. Instrument : 1260                           Location :   66
    Injection Date  : 11/17/2018 8:49:36 PM               Inj :   1
                                                 Inj Volume : 5.000 µl
    Acq. Method     : E:\DATA\XSM-In33\In33 181123 03-150 XSM Ph-aH 4p div 2018-11-17 20-06-32
                      \In33 1st AD-98-40min-232nm Ph-aH 4p.M
    Last changed    : 11/17/2018 8:06:32 PM by SYSTEM
    Analysis Method : E:\DATA\XSM-In33\In33 181123 03-150 XSM Ph-aH 4p div 2018-11-17 20-06-32
                      \In33 1st AD-98-40min-232nm Ph-aH 4p.M (Sequence Method)
    Last changed    : 1/2/2019 3:43:23 PM by SYSTEM
                      (modified after loading)
    Additional Info : Peak(s) manually integrated
```

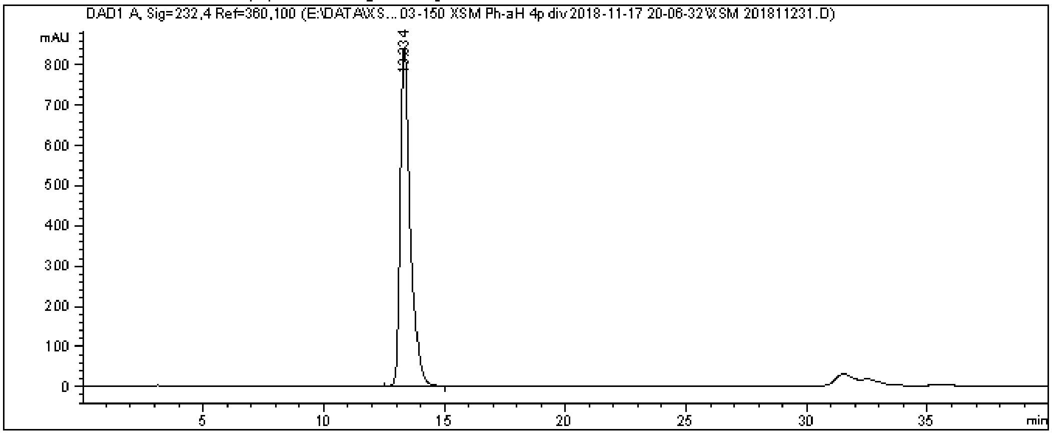


```
    =====================================================================
                             Area Percent Report
    =====================================================================

    Sorted By             :      Signal
    Multiplier            :      1.0000
    Dilution              :      1.0000
    Do not use Multiplier & Dilution Factor with ISTDs


    Signal 1: DAD1 A, Sig=232,4 Ref=360,100

    Peak RetTime Type  Width     Area      Height     Area
      #   [min]        [min]   [mAU*s]     [mAU]        %
    ----|-------|----|-------|----------|----------|--------|
       1  13.334 BB    0.4151 2.39905e4  842.88348 100.0000

    Totals :                  2.39905e4  842.88348


    =====================================================================
                              *** End of Report ***


1260 1/2/2019 3:43:31 PM SYSTEM                                           Page 1 of 1
```

Supplementary Figure 123. HPLC spectrum of (1*S*,3*R*,4*S*)-**3c**

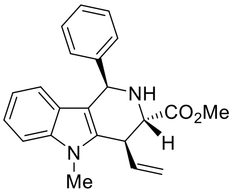

(1*R*,3*S*,4*R*)-**3c**

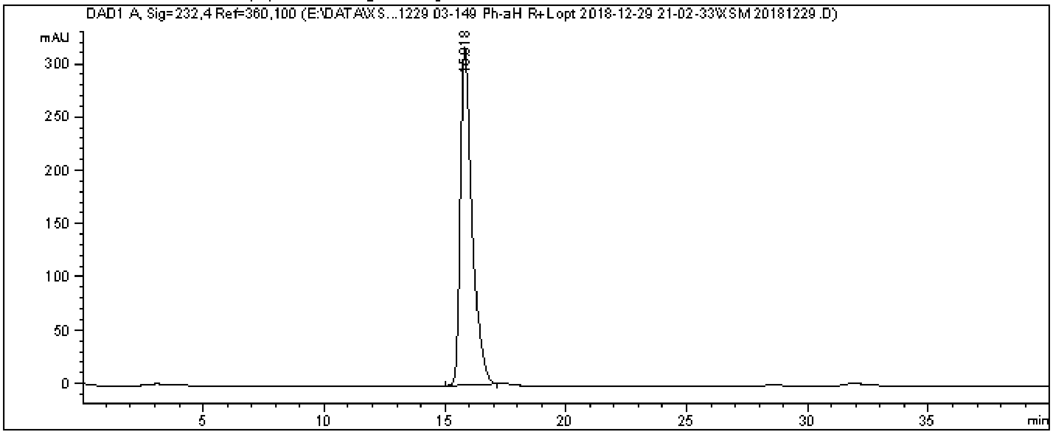


```
Data File E:\DATA\XSM\In33 181229 03-149 Ph-aH R+L opt 2018-12-29 21-02-33\XSM 20181229.D
Sample Name: In33 03-149 Ph-aH R+L opt

=====================================================================
Acq. Operator   : SYSTEM                        Seq. Line :   1
Acq. Instrument : 1260                           Location :   70
Injection Date  : 12/29/2018 9:04:07 PM              Inj :   1
                                               Inj Volume : 5.000 µl
Acq. Method     : E:\DATA\XSM\CX-10-139-DCE-2 2018-12-29 21-02-33\In33 1st AD-98-40min-232nm
                  Ph-aH 4p.M
Last changed    : 12/29/2018 9:02:33 PM by SYSTEM
Analysis Method : E:\DATA\XSM\In33 181229 03-149 Ph-aH R+L opt 2018-12-29 21-02-33\In33 1st
                  AD-98-40min-232nm Ph-aH 4p.M (Sequence Method)
Last changed    : 1/2/2019 3:41:15 PM by SYSTEM
                  (modified after loading)
Additional Info : Peak(s) manually integrated

=====================================================================
                        Area Percent Report
=====================================================================

Sorted By             :      Signal
Multiplier            :      1.0000
Dilution              :      1.0000
Do not use Multiplier & Dilution Factor with ISTDs


Signal 1: DAD1 A, Sig=232,4 Ref=360,100

Peak RetTime Type  Width     Area      Height     Area
  #   [min]        [min]   [mAU*s]     [mAU]        %
----|-------|----|-------|----------|----------|--------|
   1  15.818 BB    0.4923 1.06552e4   316.86420 100.0000

Totals :                  1.06552e4   316.86420


=====================================================================
                          *** End of Report ***
```


Supplementary Figure 124. HPLC spectrum of (1*R*,3*S*,4*R*)-**3c**

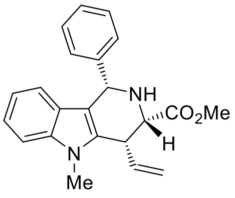

(1*S*,3*S*,4*S*)-**3c**

```
Data File E:\DATA\XSM\In33 181228 03-149 Ph-aH opt 2018-12-28 21-23-57\XSM 20181228.D
Sample Name: In33 03-149 Ph-aH S+L opt

    =====================================================================
    Acq. Operator   : SYSTEM                        Seq. Line :   1
    Acq. Instrument : 1260                           Location :   70
    Injection Date  : 12/28/2018 9:25:36 PM              Inj :   1
                                                 Inj Volume : 5.000 µl
    Acq. Method     : E:\DATA\XSM\In33 181228 03-149 Ph-aH opt 2018-12-28 21-23-57\In33 1st AD-98
                      -40min-232nm Ph-aH 4p.M
    Last changed    : 12/28/2018 9:23:58 PM by SYSTEM
    Analysis Method : E:\DATA\XSM\In33 181228 03-149 Ph-aH opt 2018-12-28 21-23-57\In33 1st AD-98
                      -40min-232nm Ph-aH 4p.M (Sequence Method)
    Last changed    : 1/2/2019 2:43:34 PM by SYSTEM
                      (modified after loading)
    Additional Info : Peak(s) manually integrated
```

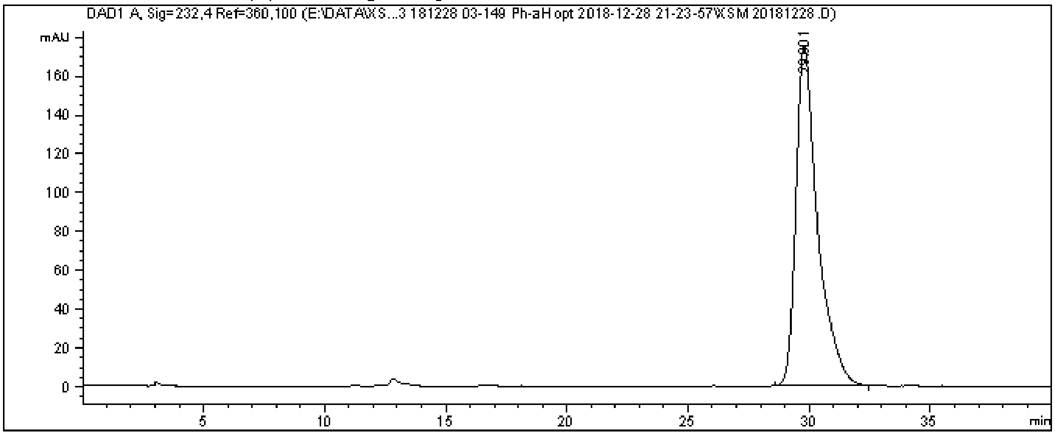


```
    =====================================================================
                             Area Percent Report
    =====================================================================

    Sorted By           :      Signal
    Multiplier          :      1.0000
    Dilution            :      1.0000
    Do not use Multiplier & Dilution Factor with ISTDs


    Signal 1: DAD1 A, Sig=232,4 Ref=360,100

    Peak RetTime Type  Width     Area      Height     Area
      #   [min]        [min]   [mAU*s]     [mAU]        %
    ----|-------|----|-------|----------|----------|--------|
       1  29.801 BB    0.8268 1.09322e4  174.52565 100.0000

    Totals :                  1.09322e4  174.52565


    =====================================================================
                              *** End of Report ***
```


Supplementary Figure 125. HPLC spectrum of (1*S*,3*S*,4*S*)-**3c**

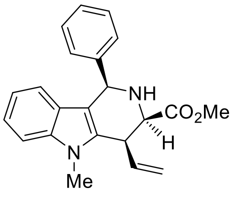

(1*R*,3*R*,4*R*)-**3c**

Data File E:\DATA\XS... 181123 03-150 XSM Ph-aH 4p div 2018-11-17 20-06-32\XSM 201811234.D
Sample Name: In33 03-149 Ph-aH R+D opt

===

```
Acq. Operator   : SYSTEM                         Seq. Line :    5
Acq. Instrument : 1260                            Location :   69
Injection Date  : 11/17/2018 10:54:13 PM               Inj :    1
                                                Inj Volume : 5.000 µl
Acq. Method     : E:\DATA\XSM-In33\In33 181123 03-150 XSM Ph-aH 4p div 2018-11-17 20-06-32
                  \In33 1st AD-98-40min-232nm Ph-aH 4p.M
Last changed    : 11/17/2018 8:06:32 PM by SYSTEM
Analysis Method : E:\DATA\XSM-In33\In33 181123 03-150 XSM Ph-aH 4p div 2018-11-17 20-06-32
                  \In33 1st AD-98-40min-232nm Ph-aH 4p.M (Sequence Method)
Last changed    : 12/15/2018 4:06:38 PM by SYSTEM
                  (modified after loading)
Additional Info : Peak(s) manually integrated
```

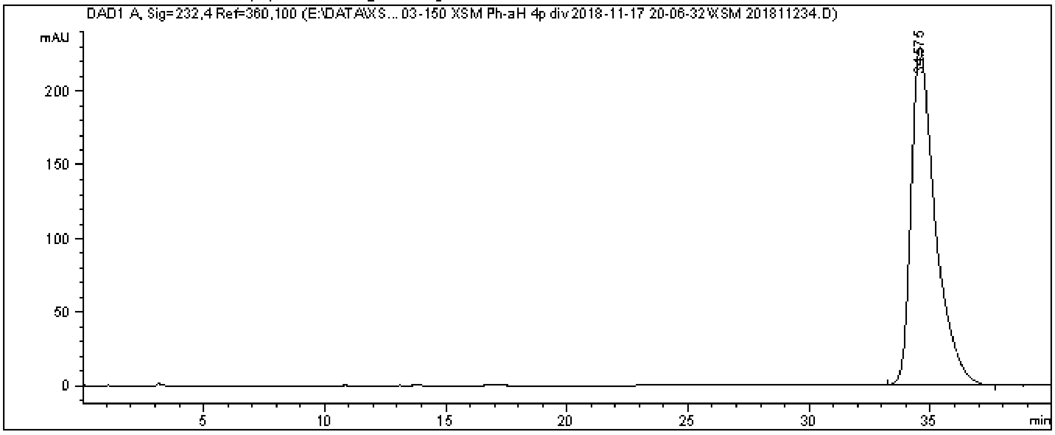

===
Area Percent Report
===

```
Sorted By             :      Signal
Multiplier            :      1.0000
Dilution              :      1.0000
Do not use Multiplier & Dilution Factor with ISTDs
```

Signal 1: DAD1 A, Sig=232,4 Ref=360,100

Peak #	RetTime [min]	Type	Width [min]	Area [mAU*s]	Height [mAU]	Area %
1	34.575	BB	0.9312	1.59828e4	229.29974	100.0000

Totals : 1.59828e4 229.29974

===
*** End of Report ***

1260 12/15/2018 4:10:17 PM SYSTEM Page 1 of 1

Supplementary Figure 126. HPLC spectrum of (1*R*,3*R*,4*R*)-**3c**

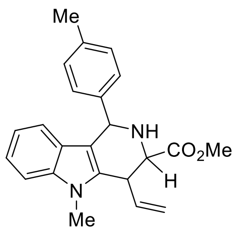

(1*R**,3*S**,4*R**)-**3d** and (1*R**,3*R**,4*R**)-**3d**

Data File E:\DATA\XS... 181121 03-151 4-Me-aH 4p 2c div 2018-11-21 21-15-03\XSM-20181121.D
Sample Name: In33 03-151 4-Me-aH rac 4peak 2column

===

Acq. Operator : SYSTEM Seq. Line : 1
Acq. Instrument : 1260 Location : 60
Injection Date : 11/21/2018 9:16:37 PM Inj : 1
Inj Volume : 20.000 µl
Acq. Method : E:\DATA\XSM\In33 181121 03-151 4-Me-aH 4p 2c div 2018-11-21 21-15-03\In33 AD-98-60min-238 4Me-aH 4p 2c div.M
Last changed : 11/21/2018 9:15:04 PM by SYSTEM
Analysis Method : E:\DATA\XSM\In33 181121 03-151 4-Me-aH 4p 2c div 2018-11-21 21-15-03\In33 AD-98-60min-238 4Me-aH 4p 2c div.M (Sequence Method)
Last changed : 12/15/2018 3:54:28 PM by SYSTEM
(modified after loading)
Additional Info : Peak(s) manually integrated

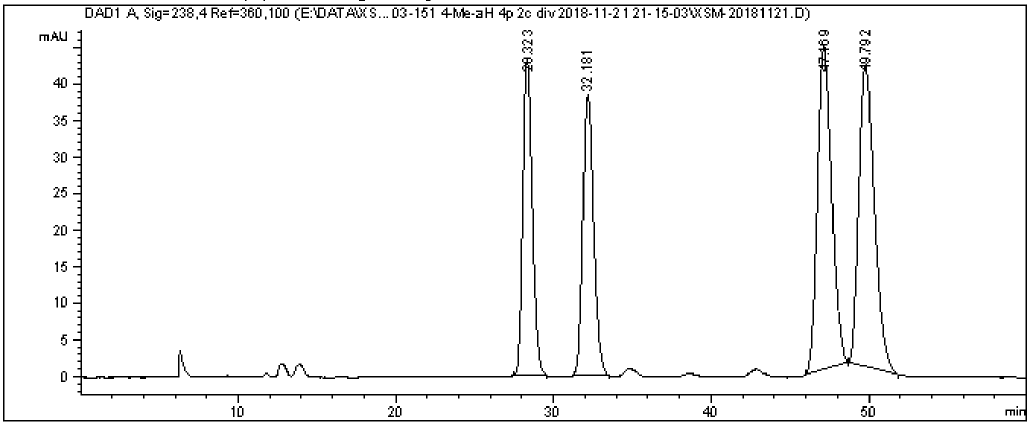

===

Area Percent Report

===

Sorted By : Signal
Multiplier : 1.0000
Dilution : 1.0000
Do not use Multiplier & Dilution Factor with ISTDs

Signal 1: DAD1 A, Sig=238,4 Ref=360,100

Peak #	RetTime [min]	Type	Width [min]	Area [mAU*s]	Height [mAU]	Area %
1	28.323	BB	0.4936	1810.04150	43.39978	19.6433
2	32.181	BB	0.5757	1817.97803	38.30616	19.7294
3	47.169	BB	0.7524	2784.21045	44.16994	30.2153
4	49.792	BB	0.7995	2802.34229	41.10054	30.4121

Totals : 9214.57227 166.97642

===

*** End of Report ***

1260 12/15/2018 3:54:31 PM SYSTEM Page 1 of 1

Supplementary Figure 127. HPLC spectrum of (1*R**,3*S**,4*R**)-**3d** and (1*R**,3*R**,4*R**)-**3d**

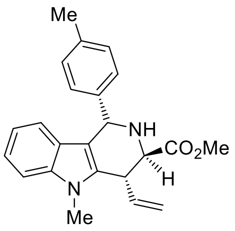

(1*S*,3*R*,4*S*)-**3d**

Data File E:\DATA\XS...181121 03-151 4-Me-aH 4p 2c div 2018-11-21 21-15-03\XSM-201811211.D
Sample Name: In33 03-151 4-Me-aH S+D opt

===

Acq. Operator : SYSTEM Seq. Line : 2
Acq. Instrument : 1260 Location : 56
Injection Date : 11/21/2018 10:18:10 PM Inj : 1
Inj Volume : 20.000 µl
Acq. Method : E:\DATA\XSM\In33 181121 03-151 4-Me-aH 4p 2c div 2018-11-21 21-15-03\In33 AD-98-60min-238 4Me-aH 4p 2c div.M
Last changed : 11/21/2018 9:15:04 PM by SYSTEM
Analysis Method : E:\DATA\XSM\In33 181121 03-151 4-Me-aH 4p 2c div 2018-11-21 21-15-03\In33 AD-98-60min-238 4Me-aH 4p 2c div.M (Sequence Method)
Last changed : 1/2/2019 3:50:26 PM by SYSTEM
(modified after loading)
Additional Info : Peak(s) manually integrated

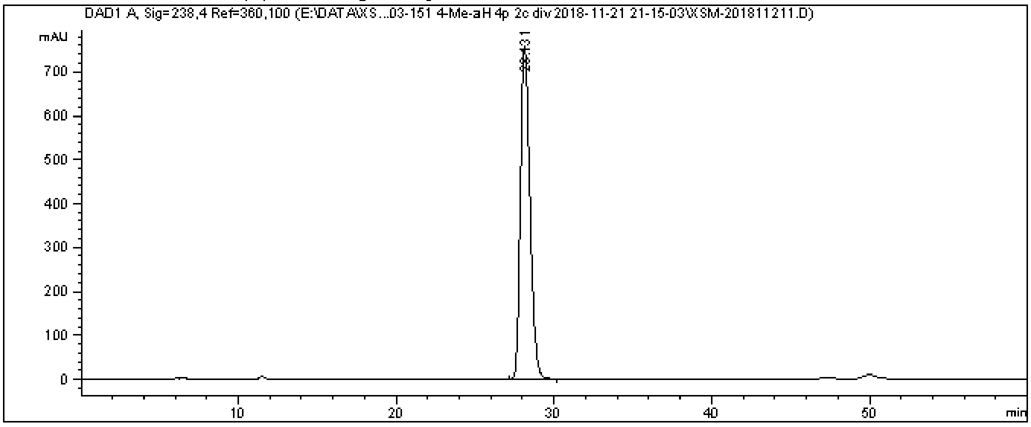

===

Area Percent Report

===

Sorted By : Signal
Multiplier : 1.0000
Dilution : 1.0000
Do not use Multiplier & Dilution Factor with ISTDs

Signal 1: DAD1 A, Sig=238,4 Ref=360,100

Peak #	RetTime [min]	Type	Width [min]	Area [mAU*s]	Height [mAU]	Area %
1	28.131	BB	0.6401	3.20875e4	757.69623	100.0000

Totals : 3.20875e4 757.69623

===

*** End of Report ***

Supplementary Figure 128. HPLC spectrum of (1*S*,3*R*,4*S*)-**3d**

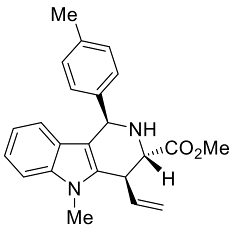

(1*R*,3*S*,4*R*)-**3d**

```
Data File E:\DATA\XSM\In33 181227 03-151 4-Me-aH 2c div 2018-12-27 22-40-15\XSM 20181227.D
Sample Name: In33 03-151 4-Me-aH R+L-1

    =====================================================================
    Acq. Operator   : SYSTEM                        Seq. Line :    1
    Acq. Instrument : 1260                           Location :   68
    Injection Date  : 12/27/2018 10:41:52 PM              Inj :    1
                                                   Inj Volume : 20.000 µl
    Acq. Method     : E:\DATA\XSM\In33 181227 03-151 4-Me-aH 2c div 2018-12-27 22-40-15\In33 AD-
                      98-60min-238 4Me-aH 4p 2c div.M
    Last changed    : 12/27/2018 10:40:16 PM by SYSTEM
    Analysis Method : E:\DATA\XSM\In33 181227 03-151 4-Me-aH 2c div 2018-12-27 22-40-15\In33 AD-
                      98-60min-238 4Me-aH 4p 2c div.M (Sequence Method)
    Last changed    : 1/2/2019 3:55:22 PM by SYSTEM
                      (modified after loading)
    Additional Info : Peak(s) manually integrated
```

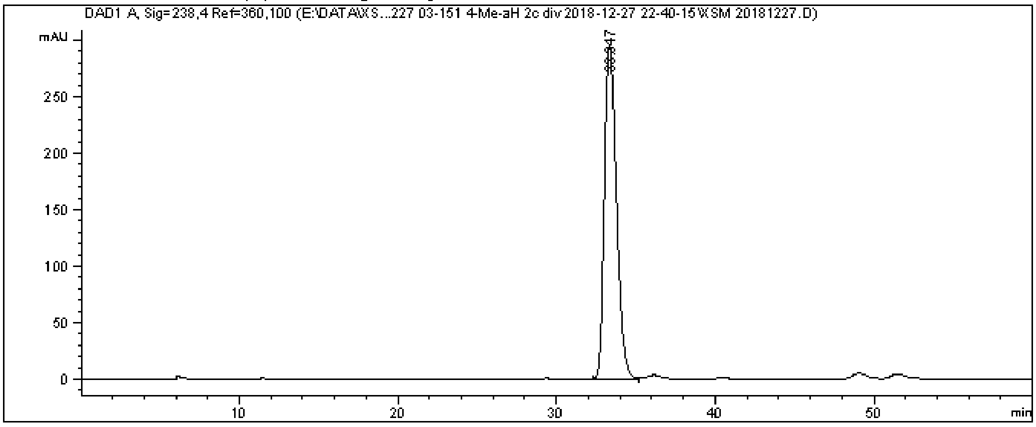


```
    =====================================================================
                             Area Percent Report
    =====================================================================

    Sorted By             :      Signal
    Multiplier            :      1.0000
    Dilution              :      1.0000
    Do not use Multiplier & Dilution Factor with ISTDs


    Signal 1: DAD1 A, Sig=238,4 Ref=360,100

    Peak RetTime Type  Width     Area      Height     Area
      #   [min]        [min]   [mAU*s]     [mAU]        %
    ----|-------|----|-------|----------|----------|--------|
       1  33.347 BB    0.7472 1.53247e4   294.44476 100.0000

    Totals :                  1.53247e4   294.44476


    =====================================================================
                              *** End of Report ***
```

1260 1/2/2019 3:55:25 PM SYSTEM Page 1 of 1

Supplementary Figure 129. HPLC spectrum of (1*R*,3*S*,4*R*)-**3d**

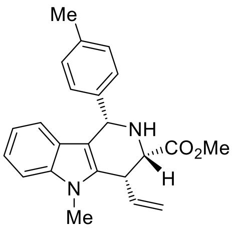

(1*S*,3*S*,4*S*)-**3d**

```
Data File E:\DATA\XS...181121 03-151 4-Me-aH 4p 2c div 2018-11-21 21-15-03\XSM-201811213.D
Sample Name: In33 03-151 4-Me-aH S+L opt

    =====================================================================
    Acq. Operator   : SYSTEM                          Seq. Line :   4
    Acq. Instrument : 1260                             Location :   58
    Injection Date  : 11/22/2018 12:21:37 AM                Inj :   1
                                                     Inj Volume : 20.000 µl
    Acq. Method     : E:\DATA\XSM\In33 181121 03-151 4-Me-aH 4p 2c div 2018-11-21 21-15-03\In33
                      AD-98-60min-238 4Me-aH 4p 2c div.M
    Last changed    : 11/21/2018 9:15:04 PM by SYSTEM
    Analysis Method : E:\DATA\XSM\In33 181121 03-151 4-Me-aH 4p 2c div 2018-11-21 21-15-03\In33
                      AD-98-60min-238 4Me-aH 4p 2c div.M (Sequence Method)
    Last changed    : 1/2/2019 3:50:26 PM by SYSTEM
                      (modified after loading)
    Additional Info : Peak(s) manually integrated
```

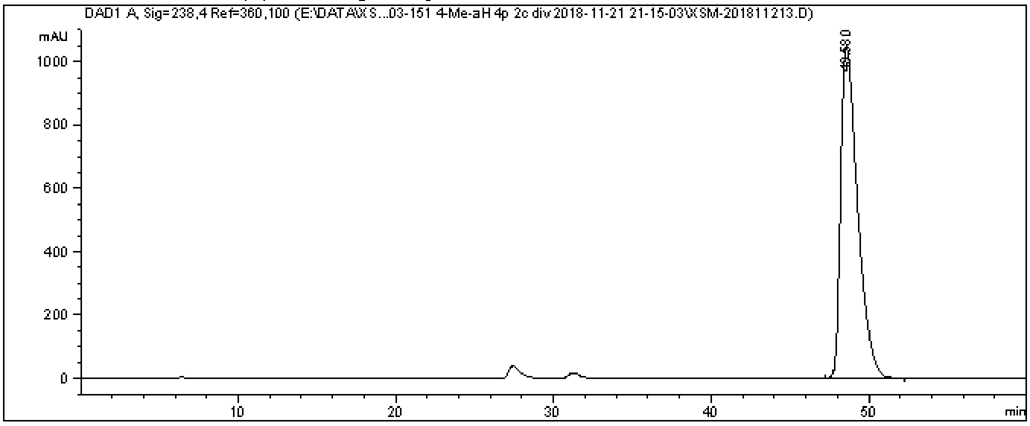


```
    =====================================================================
                             Area Percent Report
    =====================================================================

    Sorted By             :      Signal
    Multiplier            :      1.0000
    Dilution              :      1.0000
    Do not use Multiplier & Dilution Factor with ISTDs


    Signal 1: DAD1 A, Sig=238,4 Ref=360,100

    Peak RetTime Type  Width     Area      Height     Area
      #   [min]        [min]   [mAU*s]     [mAU]        %
    ----|-------|----|-------|----------|----------|--------|
       1  48.580 BB    1.0054 7.90543e4  1048.80359 100.0000

    Totals :                  7.90543e4  1048.80359


    =====================================================================
                              *** End of Report ***
```


Supplementary Figure 130. HPLC spectrum of (1*S*,3*S*,4*S*)-**3d**

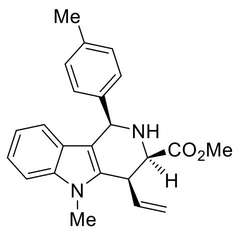

(1*R*,3*R*,4*R*)-**3d**

```
Data File E:\DATA\XSM\In33 181228 03-151 4-Me-aH opt 2018-12-28 17-43-05\XSM 20181228.D
Sample Name: In33 03-151 4-Me-aH R+D opt

    =====================================================================
    Acq. Operator   : SYSTEM                         Seq. Line :    1
    Acq. Instrument : 1260                            Location :   70
    Injection Date  : 12/28/2018 5:44:38 PM                Inj :    1
                                                    Inj Volume : 20.000 µl
    Acq. Method     : E:\DATA\XSM\In33 181228 03-151 4-Me-aH opt 2018-12-28 17-43-05\In33 AD-98-
                      60min-238 4Me-aH 4p 2c div.M
    Last changed    : 12/28/2018 5:43:06 PM by SYSTEM
    Analysis Method : E:\DATA\XSM\In33 181228 03-151 4-Me-aH opt 2018-12-28 17-43-05\In33 AD-98-
                      60min-238 4Me-aH 4p 2c div.M (Sequence Method)
    Last changed    : 1/2/2019 2:46:23 PM by SYSTEM
                      (modified after loading)
    Additional Info : Peak(s) manually integrated
```

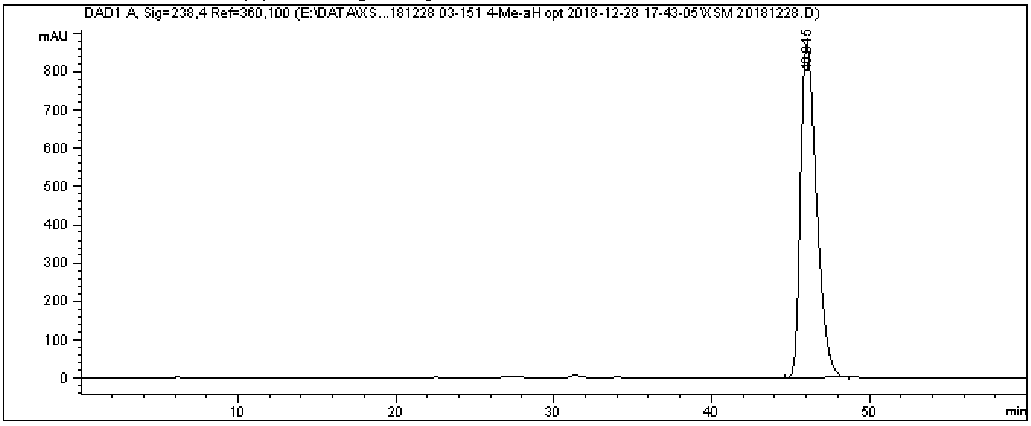


```
    =====================================================================
                             Area Percent Report
    =====================================================================

    Sorted By             :      Signal
    Multiplier            :      1.0000
    Dilution              :      1.0000
    Do not use Multiplier & Dilution Factor with ISTDs


    Signal 1: DAD1 A, Sig=238,4 Ref=360,100

    Peak RetTime Type  Width     Area      Height     Area
      #   [min]        [min]   [mAU*s]     [mAU]        %
    ----|-------|----|-------|----------|----------|--------|
       1  46.045 BB    0.8462 6.10427e4   866.19482 100.0000

    Totals :                  6.10427e4   866.19482


    =====================================================================
                              *** End of Report ***
```

1260 1/2/2019 2:46:26 PM SYSTEM   Page 1 of 1

Supplementary Figure 131. HPLC spectrum of (1*R*,3*R*,4*R*)-**3d**

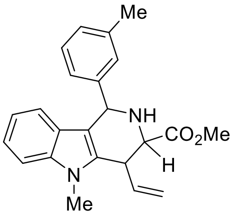

(1*R**,3*S**,4*R**)-**3e**

Data File E:\DATA\XSM\In33 190112 04-12 23Me-aH 2col 2019-01-12 11-06-02\XSM 201901125.D
Sample Name: In33 04-12 3-Me-aH rac

===

Acq. Operator : SYSTEM Seq. Line : 6
Acq. Instrument : 1260 Location : 80
Injection Date : 1/12/2019 1:54:02 PM Inj : 1
Inj Volume : 5.000 µl
Acq. Method : E:\DATA\XSM\In33 190112 04-12 23Me-aH 2col 2019-01-12 11-06-02\In33 AD-98-234nm-40min 23Me-aH 2col.M
Last changed : 1/12/2019 2:56:50 PM by SYSTEM
(modified after loading)
Analysis Method : E:\DATA\XSM\In33 190112 04-12 23Me-aH 2col 2019-01-12 11-06-02\In33 AD-98-234nm-40min 23Me-aH 2col.M (Sequence Method)
Last changed : 1/12/2019 5:19:13 PM by SYSTEM
(modified after loading)
Additional Info : Peak(s) manually integrated

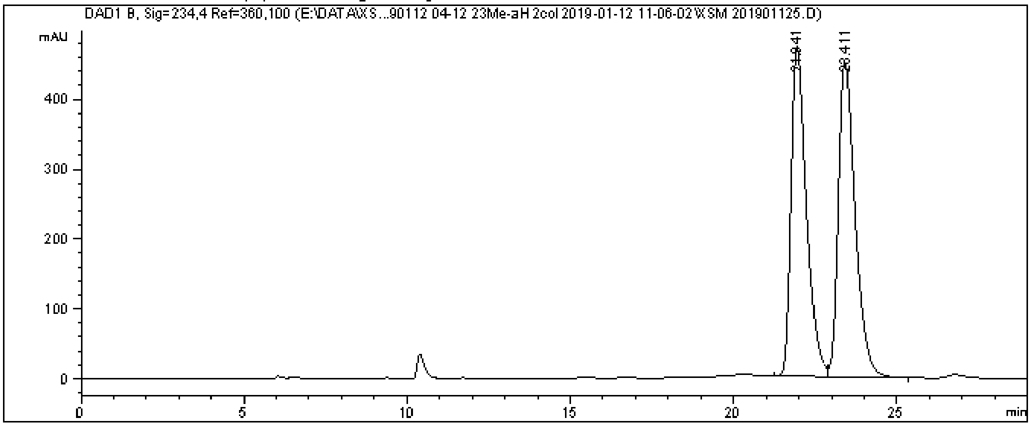

===

Area Percent Report

===

Sorted By : Signal
Multiplier : 1.0000
Dilution : 1.0000
Do not use Multiplier & Dilution Factor with ISTDs

Signal 1: DAD1 B, Sig=234,4 Ref=360,100

Peak #	RetTime [min]	Type	Width [min]	Area [mAU*s]	Height [mAU]	Area %
1	21.941	BV	0.4924	1.55540e4	472.09314	49.3371
2	23.411	VB	0.5293	1.59720e4	450.89838	50.6629

Totals : 3.15260e4 922.99152

===

*** End of Report ***

1260 1/12/2019 5:19:18 PM SYSTEM Page 1 of 1

Supplementary Figure 132. HPLC spectrum of (1*R**,3*S**,4*R**)-**3e**

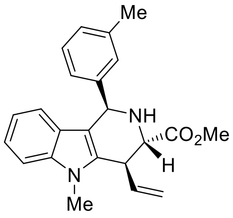

(1*R*,3*S*,4*R*)-**3e**

```
Data File E:\DATA\XSM\In33 190112 04-12 23Me-aH 2col 2019-01-12 11-06-02\XSM 201901124.D
Sample Name: In33 04-12 3-Me-aH R+L opt

    =====================================================================
    Acq. Operator   : SYSTEM                          Seq. Line :   5
    Acq. Instrument : 1260                             Location :   79
    Injection Date  : 1/12/2019 1:13:05 PM                   Inj :   1
                                                    Inj Volume : 5.000 µl
    Acq. Method     : E:\DATA\XSM\In33 190112 04-12 23Me-aH 2col 2019-01-12 11-06-02\In33 AD-98-
                      234nm-40min 23Me-aH 2col.M
    Last changed    : 1/12/2019 11:06:03 AM by SYSTEM
    Analysis Method : E:\DATA\XSM\In33 190112 04-12 23Me-aH 2col 2019-01-12 11-06-02\In33 AD-98-
                      234nm-40min 23Me-aH 2col.M (Sequence Method)
    Last changed    : 1/12/2019 5:16:07 PM by SYSTEM
                      (modified after loading)
    Additional Info : Peak(s) manually integrated
```

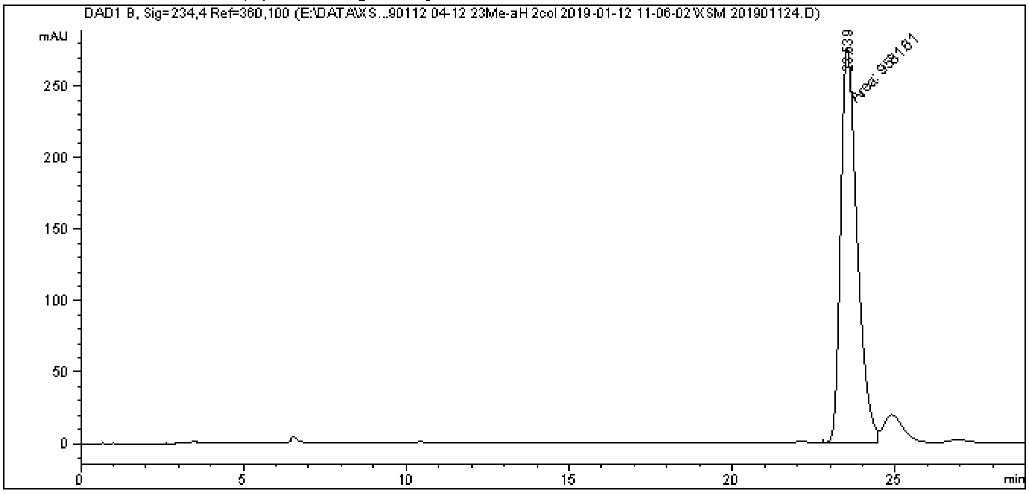


```
    =====================================================================
                         Area Percent Report
    =====================================================================

    Sorted By             :      Signal
    Multiplier            :      1.0000
    Dilution              :      1.0000
    Do not use Multiplier & Dilution Factor with ISTDs


    Signal 1: DAD1 B, Sig=234,4 Ref=360,100

    Peak RetTime Type  Width     Area      Height     Area
      #   [min]        [min]   [mAU*s]     [mAU]        %
    ----|-------|----|-------|----------|----------|--------|
       1  23.539 MF    0.5797 9581.61426  275.49536 100.0000

    Totals :                  9581.61426  275.49536


    =====================================================================
                          *** End of Report ***
```

1260 1/12/2019 5:16:14 PM SYSTEM — Page 1 of 1

Supplementary Figure 133. HPLC spectrum of (1*R*,3*S*,4*R*)-**3e**

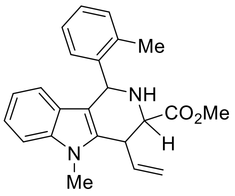

(1*R**,3*S**,4*R**)-**3f**

Data File E:\DATA\XSM\In33 190112 04-12 23Me-aH 2col 2019-01-12 11-06-02\XSM 201901121.D
Sample Name: In33 04-12 2-Me-aH rac

===

Acq. Operator : SYSTEM   Seq. Line : 2
Acq. Instrument : 1260   Location : 78
Injection Date : 1/12/2019 11:48:00 AM   Inj : 1
Inj Volume : 5.000 µl
Acq. Method : E:\DATA\XSM\In33 190112 04-12 23Me-aH 2col 2019-01-12 11-06-02\In33 AD-98-234nm-40min 23Me-aH 2col.M
Last changed : 1/12/2019 11:06:03 AM by SYSTEM
Analysis Method : E:\DATA\XSM\In33 190112 04-12 23Me-aH 2col 2019-01-12 11-06-02\In33 AD-98-234nm-40min 23Me-aH 2col.M (Sequence Method)
Last changed : 1/12/2019 5:16:07 PM by SYSTEM
(modified after loading)
Additional Info : Peak(s) manually integrated

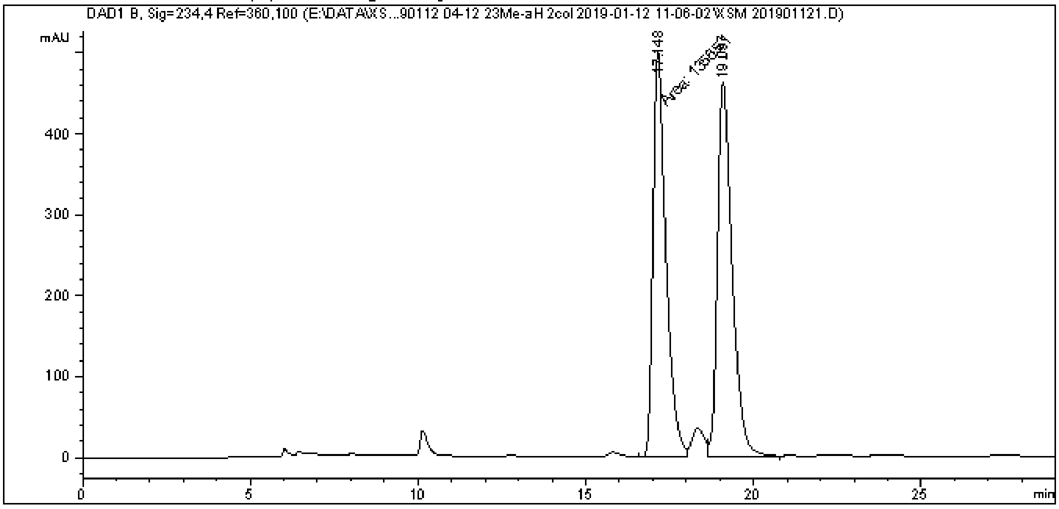

===

Area Percent Report

===

Sorted By : Signal
Multiplier : 1.0000
Dilution : 1.0000
Do not use Multiplier & Dilution Factor with ISTDs

Signal 1: DAD1 B, Sig=234,4 Ref=360,100

Peak #	RetTime [min]	Type	Width [min]	Area [mAU*s]	Height [mAU]	Area %
1	17.148	MF	0.4503	1.35651e4	502.08762	49.3089
2	19.097	VB	0.4477	1.39453e4	462.64755	50.6911

Totals : 2.75104e4 964.73517

===

*** End of Report ***

1260 1/12/2019 5:16:57 PM SYSTEM   Page 1 of 1

Supplementary Figure 134. HPLC spectrum of (1*R**,3*S**,4*R**)-**3f**

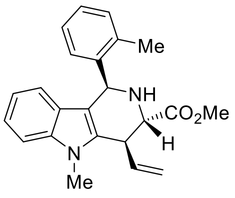

(1*R*,3*S*,4*R*)-**3f**

Data File E:\DATA\XSM\In33 190112 04-12 23Me-aH 2col 2019-01-12 11-06-02\XSM 201901122.D
Sample Name: In33 04-12 2-Me-aH R+L opt

```
=====================================================================
Acq. Operator   : SYSTEM                        Seq. Line :    3
Acq. Instrument : 1260                           Location :   77
Injection Date  : 1/12/2019 12:28:59 PM               Inj :    1
                                               Inj Volume : 5.000 µl
Acq. Method     : E:\DATA\XSM\In33 190112 04-12 23Me-aH 2col 2019-01-12 11-06-02\In33 AD-98-
                  234nm-40min 23Me-aH 2col.M
Last changed    : 1/12/2019 11:06:03 AM by SYSTEM
Analysis Method : E:\DATA\XSM\In33 190112 04-12 23Me-aH 2col 2019-01-12 11-06-02\In33 AD-98-
                  234nm-40min 23Me-aH 2col.M (Sequence Method)
Last changed    : 1/12/2019 5:16:07 PM by SYSTEM
                  (modified after loading)
Additional Info : Peak(s) manually integrated
```

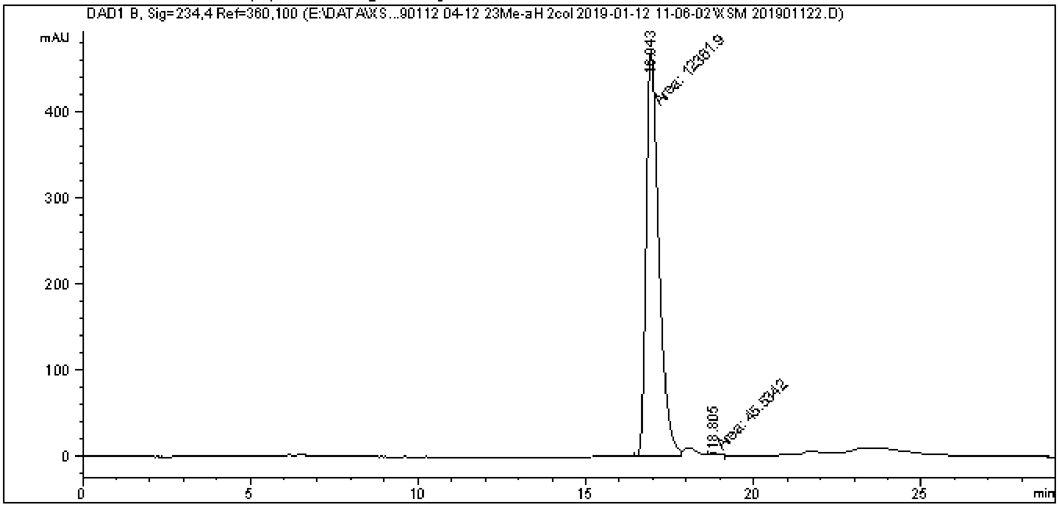


```
=====================================================================
                         Area Percent Report
=====================================================================

Sorted By             :      Signal
Multiplier            :      1.0000
Dilution              :      1.0000
Do not use Multiplier & Dilution Factor with ISTDs


Signal 1: DAD1 B, Sig=234,4 Ref=360,100

Peak RetTime Type  Width     Area      Height     Area
  #   [min]        [min]   [mAU*s]     [mAU]        %
----|-------|----|-------|----------|----------|--------|
   1  16.943 MF    0.4377 1.23619e4  470.70593  99.6330
   2  18.805 MM    0.3073   45.53419    2.46981   0.3670

Totals :                  1.24075e4  473.17574


=====================================================================
                          *** End of Report ***
```

1260 1/12/2019 5:17:07 PM SYSTEM   Page 1 of 1

Supplementary Figure 135. HPLC spectrum of (1*R*,3*S*,4*R*)-**3f**

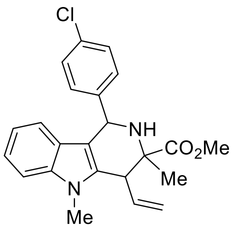

(1*R**,3*S**,4*R**)-**3g**

Data File E:\DATA\XS...\XSM 20180225 03-39 4-Cl 1st div 2018-12-25 19-09-33\XSM 20181225.D
Sample Name: In33 03-39 4-Cl 1st -rac

```
=====================================================================
Acq. Operator   : SYSTEM                         Seq. Line :   1
Acq. Instrument : 1260                            Location :   68
Injection Date  : 12/25/2018 7:10:29 PM                Inj :   1
                                                Inj Volume : 10.000 µl
Acq. Method     : E:\DATA\XSM-In33\XSM 20180225 03-39 4-Cl 1st div 2018-12-25 19-09-33\In33
                  AS-98-0.5mL-234-82nm-10uL 4-Cl 1st.M
Last changed    : 12/25/2018 7:09:33 PM by SYSTEM
Analysis Method : E:\DATA\XSM-In33\XSM 20180225 03-39 4-Cl 1st div 2018-12-25 19-09-33\In33
                  AS-98-0.5mL-234-82nm-10uL 4-Cl 1st.M (Sequence Method)
Last changed    : 1/2/2019 3:32:35 PM by SYSTEM
                  (modified after loading)
Additional Info : Peak(s) manually integrated
```

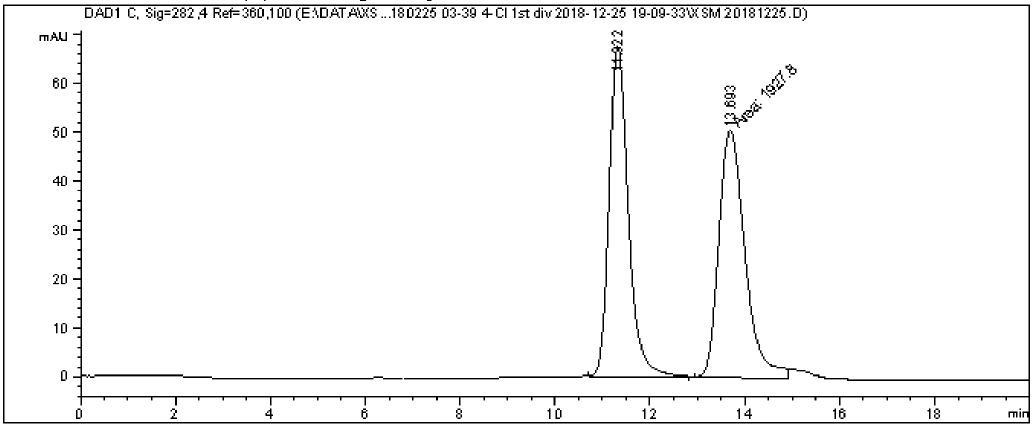


```
=====================================================================
                         Area Percent Report
=====================================================================

Sorted By             :      Signal
Multiplier            :      1.0000
Dilution              :      1.0000
Do not use Multiplier & Dilution Factor with ISTDs


Signal 1: DAD1 C, Sig=282,4 Ref=360,100

Peak RetTime Type  Width     Area      Height     Area
  #   [min]        [min]   [mAU*s]     [mAU]        %
----|-------|----|-------|----------|----------|--------|
   1  11.322 BB    0.4265 1888.88147   67.50159  49.4902
   2  13.693 MF    0.6373 1927.79944   50.41568  50.5098

Totals :                  3816.68091  117.91727


=====================================================================
                          *** End of Report ***
```

1260 1/2/2019 3:32:39 PM SYSTEM Page 1 of 1

Supplementary Figure 136. HPLC spectrum of (1*R**,3*S**,4*R**)-**3g**

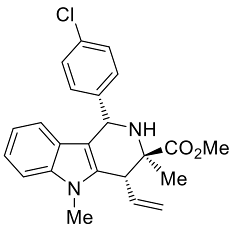

(1*S*,3*R*,4*S*)-**3g**

```
Data File E:\DATA\XSM\In33 190111 03-39 4-Cl 1st 2019-01-11 12-04-05\XSM 20190111.D
Sample Name: In33 03-39 4-Cl S+D opt

    =====================================================================
    Acq. Operator   : SYSTEM                          Seq. Line :    1
    Acq. Instrument : 1260                             Location :   68
    Injection Date  : 1/11/2019 12:05:06 PM                 Inj :    1
                                                     Inj Volume : 5.000 µl
    Acq. Method     : E:\DATA\XSM\In33 190111 03-39 4-Cl 1st 2019-01-11 12-04-05\In33 AS-98-0.5mL
                      -282nm-25min 4-Cl 1st.M
    Last changed    : 1/11/2019 12:04:06 PM by SYSTEM
    Analysis Method : E:\DATA\XSM\In33 190111 03-39 4-Cl 1st 2019-01-11 12-04-05\In33 AS-98-0.5mL
                      -282nm-25min 4-Cl 1st.M (Sequence Method)
    Last changed    : 1/11/2019 2:59:44 PM by SYSTEM
                      (modified after loading)
    Additional Info : Peak(s) manually integrated
```

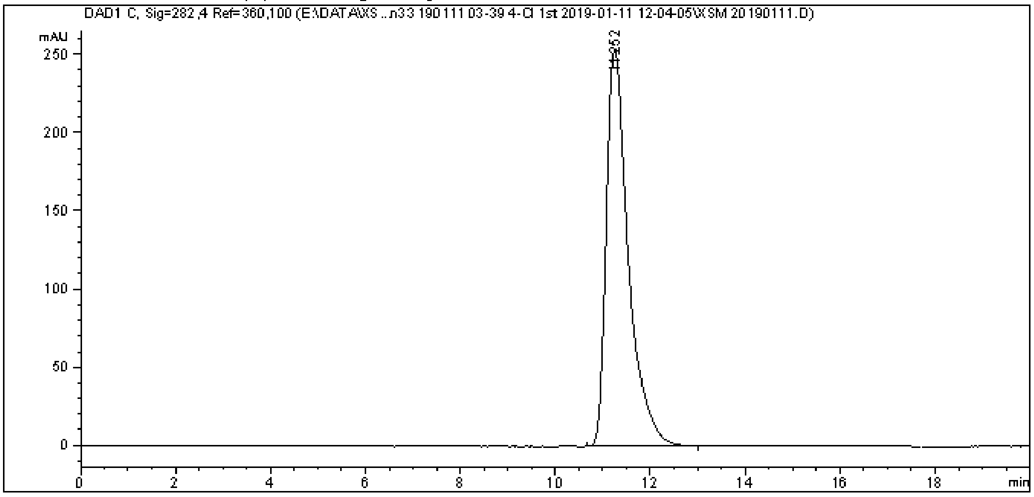


```
    =====================================================================
                             Area Percent Report
    =====================================================================

    Sorted By             :      Signal
    Multiplier            :      1.0000
    Dilution              :      1.0000
    Do not use Multiplier & Dilution Factor with ISTDs


    Signal 1: DAD1 C, Sig=282,4 Ref=360,100

    Peak RetTime Type  Width     Area      Height     Area
      #   [min]        [min]   [mAU*s]     [mAU]        %
    ----|-------|----|-------|----------|----------|--------|
       1  11.252 BB    0.4821 8172.99854  253.55905 100.0000

    Totals :                  8172.99854  253.55905


    =====================================================================
                              *** End of Report ***
```

1260 1/11/2019 3:00:30 PM SYSTEM — Page 1 of 1

Supplementary Figure 137. HPLC spectrum of (1*S*,3*R*,4*S*)-**3g**

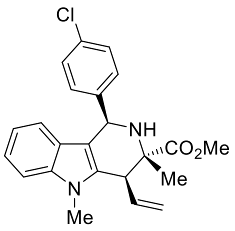

(1*R*,3*S*,4*R*)-**3g**

```
Data File E:\DATA\XSM\In33 190111 03-39 4-Cl 1st 2019-01-11 12-04-05\XSM 201901111.D
Sample Name: In33 03-39 4-Cl R+L opt

    =====================================================================
    Acq. Operator   : SYSTEM                        Seq. Line :    2
    Acq. Instrument : 1260                           Location :   69
    Injection Date  : 1/11/2019 12:31:05 PM               Inj :    1
                                                   Inj Volume : 5.000 µl
    Acq. Method     : E:\DATA\XSM\In33 190111 03-39 4-Cl 1st 2019-01-11 12-04-05\In33 AS-98-0.5mL
                      -282nm-25min 4-Cl 1st.M
    Last changed    : 1/11/2019 12:04:06 PM by SYSTEM
    Analysis Method : E:\DATA\XSM\In33 190111 03-39 4-Cl 1st 2019-01-11 12-04-05\In33 AS-98-0.5mL
                      -282nm-25min 4-Cl 1st.M (Sequence Method)
    Last changed    : 1/11/2019 2:59:44 PM by SYSTEM
                      (modified after loading)
    Additional Info : Peak(s) manually integrated
```

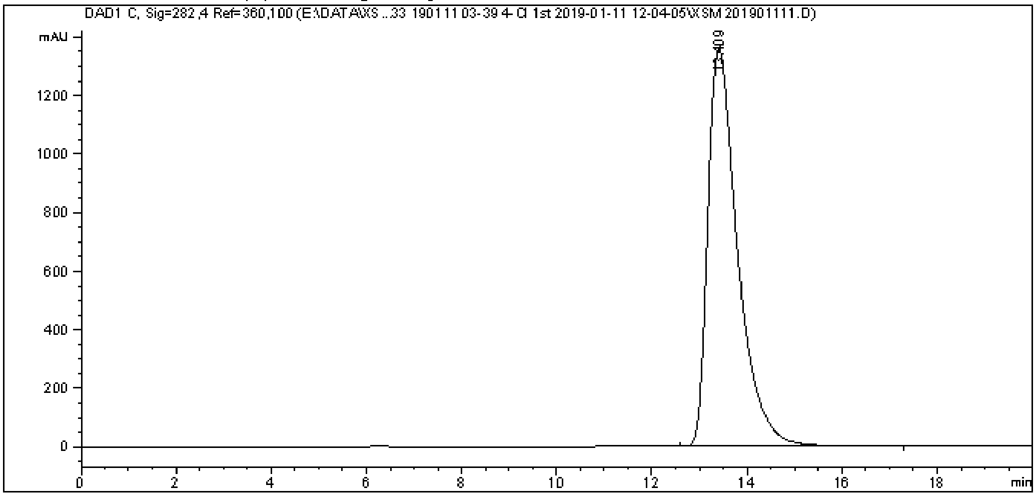


```
    =====================================================================
                             Area Percent Report
    =====================================================================

    Sorted By             :      Signal
    Multiplier            :      1.0000
    Dilution              :      1.0000
    Do not use Multiplier & Dilution Factor with ISTDs


    Signal 1: DAD1 C, Sig=282,4 Ref=360,100

    Peak RetTime Type  Width     Area      Height     Area
      #   [min]        [min]   [mAU*s]     [mAU]        %
    ----|-------|----|-------|----------|----------|--------|
       1  13.409 BB    0.6533 5.92288e4  1356.78369 100.0000

    Totals :                  5.92288e4  1356.78369


    =====================================================================
                             *** End of Report ***
```


Supplementary Figure 138. HPLC spectrum of (1*R*,3*S*,4*R*)-**3g**

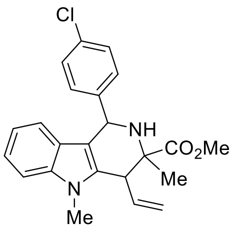

(1*R**,3*R**,4*R**)-**3g**

Data File E:\DATA\XSM\In33 181217 03-39 4-Cl 2nd 2018-12-17 13-36-21\XSM 201812172.D
Sample Name: In33 03-39 4-Cl 2nd rac

===

Acq. Operator : SYSTEM   Seq. Line : 3
Acq. Instrument : 1260   Location : 70
Injection Date : 12/17/2018 2:19:19 PM   Inj : 1
Inj Volume : 5.000 µl
Acq. Method : E:\DATA\XSM\In33 181217 03-39 4-Cl 2nd 2018-12-17 13-36-21\In33 AS-98-1ml-20min-260-82nm 4-Cl 2nd.M
Last changed : 12/17/2018 1:36:22 PM by SYSTEM
Analysis Method : E:\DATA\XSM\In33 181217 03-39 4-Cl 2nd 2018-12-17 13-36-21\In33 AS-98-1ml-20min-260-82nm 4-Cl 2nd.M (Sequence Method)
Last changed : 1/2/2019 3:23:48 PM by SYSTEM
(modified after loading)

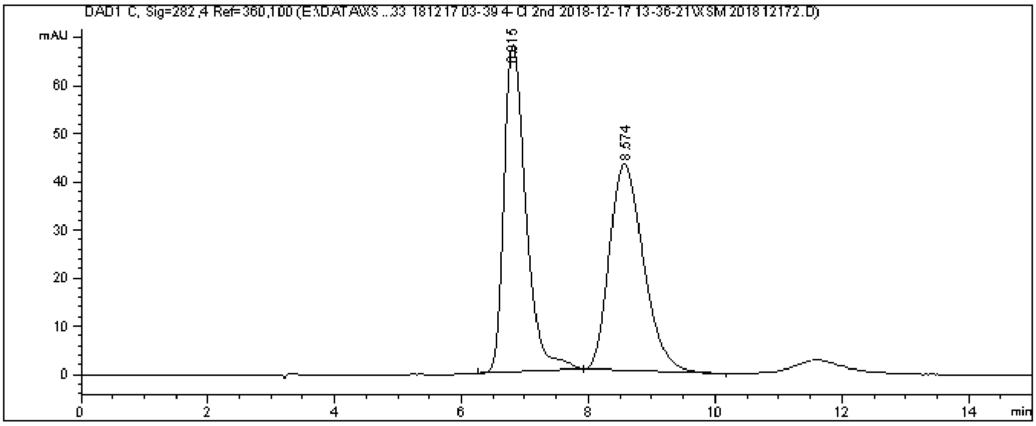

===

Area Percent Report

===

Sorted By : Signal
Multiplier : 1.0000
Dilution : 1.0000
Do not use Multiplier & Dilution Factor with ISTDs

Signal 1: DAD1 C, Sig=282,4 Ref=360,100

Peak #	RetTime [min]	Type	Width [min]	Area [mAU*s]	Height [mAU]	Area %
1	6.815	BB	0.3631	1625.36267	67.92990	50.3685
2	8.574	BB	0.5701	1601.57800	42.84415	49.6315

Totals : 3226.94067 110.77405

===

*** End of Report ***

1260 1/2/2019 3:23:51 PM SYSTEM   Page 1 of 1

Supplementary Figure 139. HPLC spectrum of (1*R**,3*R**,4*R**)-**3g**

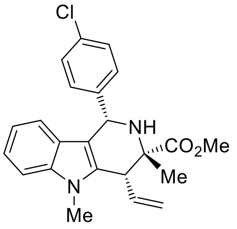

(1*S*,3*S*,4*S*)-**3g**

```
Data File E:\DATA\XSM\In33 181217 03-39 4-Cl 2nd 2018-12-17 13-36-21\XSM 20181217.D
Sample Name: In33 03-39 4-Cl S+L opt

    =====================================================================
    Acq. Operator   : SYSTEM                          Seq. Line :   1
    Acq. Instrument : 1260                             Location :   68
    Injection Date  : 12/17/2018 1:37:25 PM                 Inj :   1
                                                     Inj Volume : 5.000 µl
    Acq. Method     : E:\DATA\XSM\In33 181217 03-39 4-Cl 2nd 2018-12-17 13-36-21\In33 AS-98-1ml-
                      20min-260-82nm 4-Cl 2nd.M
    Last changed    : 12/17/2018 1:36:22 PM by SYSTEM
    Analysis Method : E:\DATA\XSM\In33 181217 03-39 4-Cl 2nd 2018-12-17 13-36-21\In33 AS-98-1ml-
                      20min-260-82nm 4-Cl 2nd.M (Sequence Method)
    Last changed    : 1/2/2019 3:23:48 PM by SYSTEM
                      (modified after loading)
```

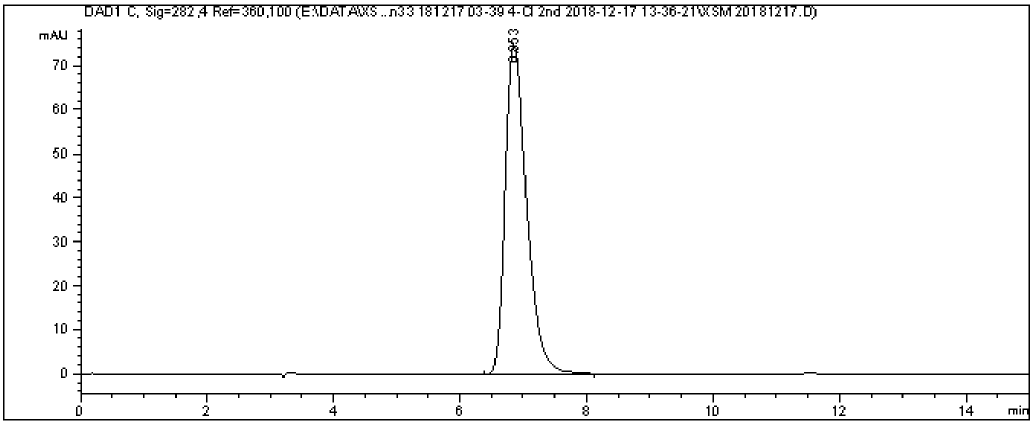


```
    =====================================================================
                             Area Percent Report
    =====================================================================

    Sorted By             :      Signal
    Multiplier            :      1.0000
    Dilution              :      1.0000
    Do not use Multiplier & Dilution Factor with ISTDs


    Signal 1: DAD1 C, Sig=282,4 Ref=360,100

    Peak RetTime Type  Width     Area      Height     Area
      #   [min]        [min]   [mAU*s]     [mAU]        %
    ----|-------|----|-------|----------|----------|--------|
       1   6.853 BB    0.3644 1772.95496   74.83467 100.0000

    Totals :                  1772.95496   74.83467


    =====================================================================
                              *** End of Report ***
```


Supplementary Figure 140. HPLC spectrum of (1*S*,3*S*,4*S*)-**3g**

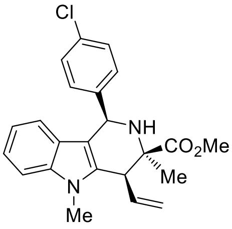

(1*R*,3*R*,4*R*)-**3g**

Data File E:\DATA\XSM\In33 181217 03-39 4-Cl 2nd 2018-12-17 13-36-21\XSM 201812171.D
Sample Name: In33 03-39 4-Cl R+D opt

===

Acq. Operator : SYSTEM Seq. Line : 2
Acq. Instrument : 1260 Location : 69
Injection Date : 12/17/2018 1:58:21 PM Inj : 1
Inj Volume : 5.000 µl
Acq. Method : E:\DATA\XSM\In33 181217 03-39 4-Cl 2nd 2018-12-17 13-36-21\In33 AS-98-1ml-20min-260-82nm 4-Cl 2nd.M
Last changed : 12/17/2018 1:36:22 PM by SYSTEM
Analysis Method : E:\DATA\XSM\In33 181217 03-39 4-Cl 2nd 2018-12-17 13-36-21\In33 AS-98-1ml-20min-260-82nm 4-Cl 2nd.M (Sequence Method)
Last changed : 1/2/2019 3:23:48 PM by SYSTEM
(modified after loading)

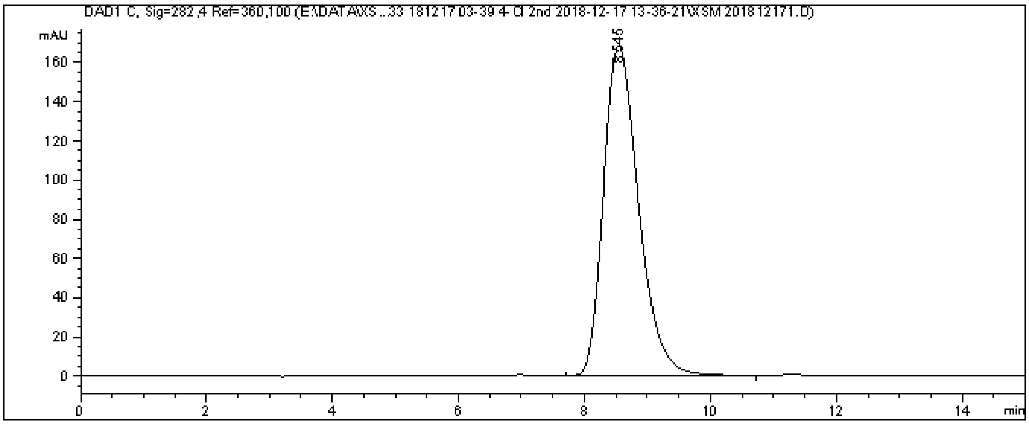

===
Area Percent Report
===

Sorted By : Signal
Multiplier : 1.0000
Dilution : 1.0000
Do not use Multiplier & Dilution Factor with ISTDs

Signal 1: DAD1 C, Sig=282,4 Ref=360,100

Peak #	RetTime [min]	Type	Width [min]	Area [mAU*s]	Height [mAU]	Area %
1	8.545	BB	0.5928	6517.98730	168.69238	100.0000

Totals : 6517.98730 168.69238

===
*** End of Report ***

1260 1/2/2019 3:23:54 PM SYSTEM Page 1 of 1

Supplementary Figure 141. HPLC spectrum of (1*R*,3*R*,4*R*)-**3g**

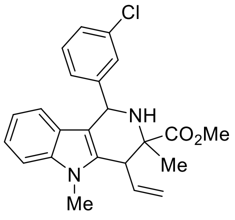

(1*R**,3*S**,4*R**)-**3h**

Data File E:\DATA\XSM-In33\In33 181205 04-1 234-Cl 2018-12-05 16-44-00\XSM 201812054.D
Sample Name: In33 04-1 3-Cl 1st rac

===

Acq. Operator : SYSTEM Seq. Line : 5
Acq. Instrument : 1260 Location : 67
Injection Date : 12/5/2018 6:24:01 PM Inj : 1
Inj Volume : 5.000 µl
Acq. Method : E:\DATA\XSM-In33\In33 181205 04-1 234-Cl 2018-12-05 16-44-00\In33 AS-98-0.5mL-234nm-25min 234-Cl.M
Last changed : 12/5/2018 4:44:00 PM by SYSTEM
Analysis Method : E:\DATA\XSM-In33\In33 181205 04-1 234-Cl 2018-12-05 16-44-00\In33 AS-98-0.5mL-234nm-25min 234-Cl.M (Sequence Method)
Last changed : 12/18/2018 7:15:06 PM by SYSTEM
(modified after loading)
Additional Info : Peak(s) manually integrated

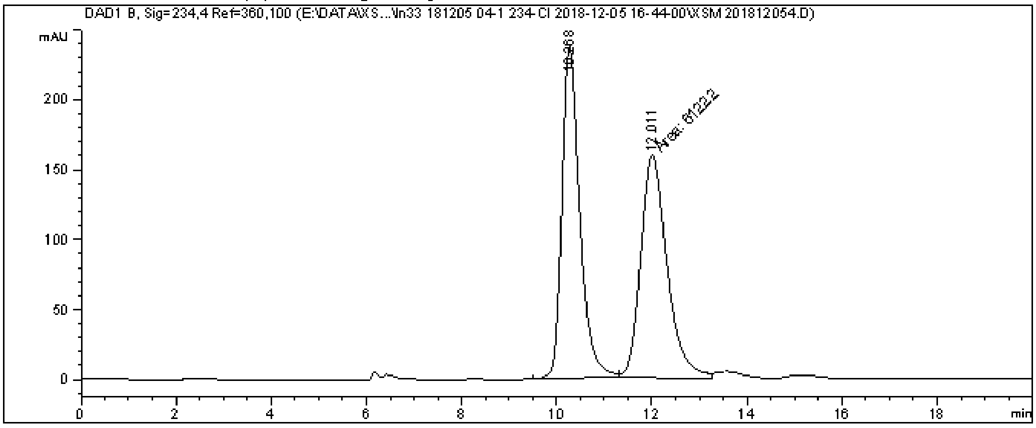

===

Area Percent Report

===

Sorted By : Signal
Multiplier : 1.0000
Dilution : 1.0000
Do not use Multiplier & Dilution Factor with ISTDs

Signal 1: DAD1 B, Sig=234,4 Ref=360,100

Peak #	RetTime [min]	Type	Width [min]	Area [mAU*s]	Height [mAU]	Area %
1	10.268	BV	0.3966	6192.99609	237.19498	50.2874
2	12.011	MM	0.6420	6122.20313	158.93069	49.7126

Totals : 1.23152e4 396.12567

===

*** End of Report ***

1260 12/18/2018 7:15:10 PM SYSTEM Page 1 of 1

Supplementary Figure 142. HPLC spectrum of (1*R**,3*S**,4*R**)-**3h**

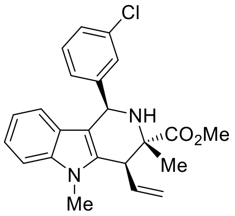

(1*R*,3*S*,4*R*)-**3h**

```
Data File E:\DATA\XSM-In33\In33 181205 04-1 234-Cl 2018-12-05 16-44-00\XSM 201812052.D
Sample Name: In33 04-1 3-Cl R+L opt

    =====================================================================
    Acq. Operator    : SYSTEM                        Seq. Line :    3
    Acq. Instrument  : 1260                           Location :   68
    Injection Date   : 12/5/2018 5:36:54 PM                Inj :    1
                                                    Inj Volume : 5.000 µl
    Acq. Method      : E:\DATA\XSM-In33\In33 181205 04-1 234-Cl 2018-12-05 16-44-00\In33 AS-98-0.
                       5mL-234nm-25min 234-Cl.M
    Last changed     : 12/5/2018 4:44:00 PM by SYSTEM
    Analysis Method  : E:\DATA\XSM-In33\In33 181205 04-1 234-Cl 2018-12-05 16-44-00\In33 AS-98-0.
                       5mL-234nm-25min 234-Cl.M (Sequence Method)
    Last changed     : 12/18/2018 7:15:06 PM by SYSTEM
                       (modified after loading)
    Additional Info  : Peak(s) manually integrated
```

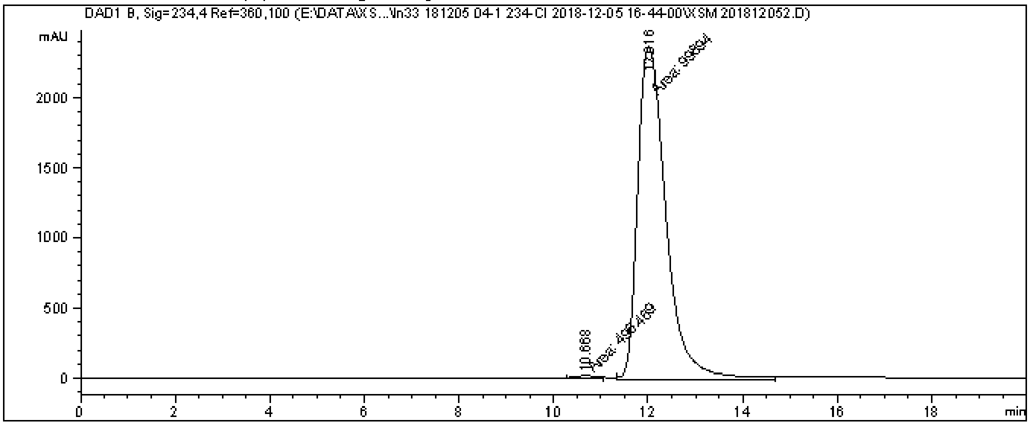


```
    =====================================================================
                             Area Percent Report
    =====================================================================

    Sorted By             :      Signal
    Multiplier            :      1.0000
    Dilution              :      1.0000
    Do not use Multiplier & Dilution Factor with ISTDs


    Signal 1: DAD1 B, Sig=234,4 Ref=360,100

    Peak RetTime Type  Width     Area      Height     Area
      #   [min]        [min]   [mAU*s]     [mAU]        %
    ----|-------|----|-------|----------|----------|--------|
       1  10.668 MM    0.4794  496.46942   17.26161   0.4945
       2  12.016 MM    0.6992 9.98940e4  2381.04370  99.5055

    Totals :                   1.00391e5  2398.30531


    =====================================================================
                              *** End of Report ***
```

1260 12/18/2018 7:16:39 PM SYSTEM Page 1 of 1

Supplementary Figure 143. HPLC spectrum of (1*R*,3*S*,4*R*)-**3h**

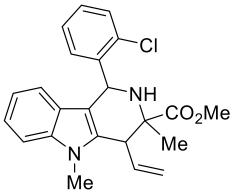

(1*R**,3*S**,4*R**)-**3i**

```
Data File E:\DATA\XSM-In33\In33 181205 04-1 234-Cl 2018-12-05 16-44-00\XSM 201812056.D
Sample Name: In33 04-1 2-Cl 1st rac

    ===================================================================
    Acq. Operator   : SYSTEM                        Seq. Line :    7
    Acq. Instrument : 1260                           Location :   65
    Injection Date  : 12/5/2018 7:15:55 PM                Inj :    1
                                                   Inj Volume : 5.000 µl
    Acq. Method     : E:\DATA\XSM-In33\In33 181205 04-1 234-Cl 2018-12-05 16-44-00\In33 AS-98-0.
                      5mL-234nm-25min 234-Cl.M
    Last changed    : 12/5/2018 4:44:00 PM by SYSTEM
    Analysis Method : E:\DATA\XSM-In33\In33 181205 04-1 234-Cl 2018-12-05 16-44-00\In33 AS-98-0.
                      5mL-234nm-25min 234-Cl.M (Sequence Method)
    Last changed    : 1/2/2019 5:47:12 PM by SYSTEM
                      (modified after loading)
    Additional Info : Peak(s) manually integrated
```

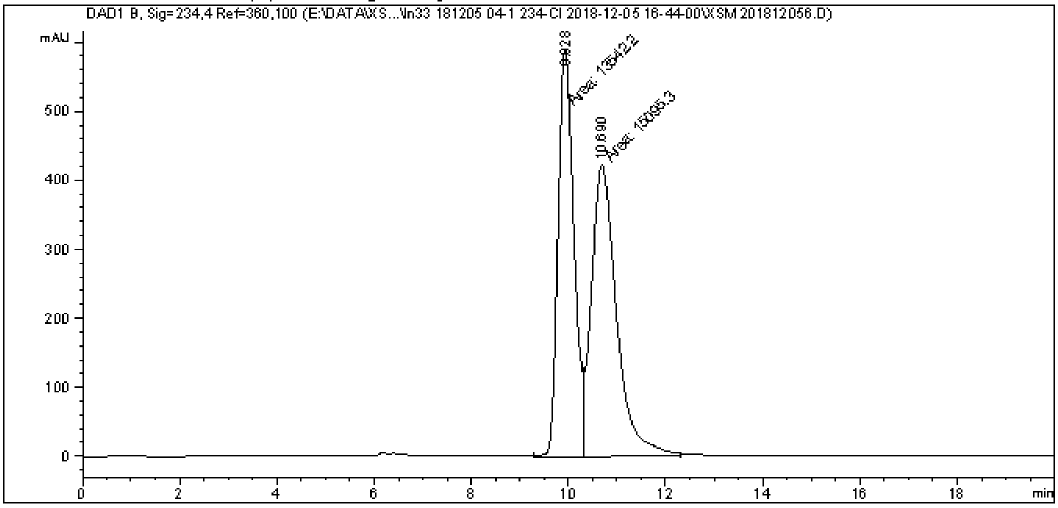


```
    ===================================================================
                             Area Percent Report
    ===================================================================

    Sorted By             :      Signal
    Multiplier            :      1.0000
    Dilution              :      1.0000
    Do not use Multiplier & Dilution Factor with ISTDs


    Signal 1: DAD1 B, Sig=234,4 Ref=360,100

    Peak RetTime Type  Width     Area      Height     Area
      #   [min]        [min]   [mAU*s]     [mAU]        %
    ----|-------|----|-------|----------|----------|--------|
       1   9.928 MF    0.3827 1.35422e4  589.69885  47.2883
       2  10.690 FM    0.5947 1.50953e4  423.07846  52.7117

    Totals :                  2.86374e4  1012.77731


    ===================================================================
                             *** End of Report ***

1260 1/2/2019 5:47:16 PM SYSTEM                                        Page 1 of 1
```

Supplementary Figure 144. HPLC spectrum of (1*R**,3*S**,4*R**)-**3i**

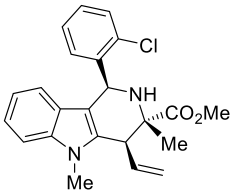

(1*R*,3*S*,4*R*)-**3i**

```
Data File E:\DATA\XSM-In33\In33 181205 04-1 234-Cl 2018-12-05 16-44-00\XSM 201812055.D
Sample Name: In33 04-1 2-Cl R+L opt

    =====================================================================
    Acq. Operator   : SYSTEM                        Seq. Line :   6
    Acq. Instrument : 1260                           Location :   66
    Injection Date  : 12/5/2018 6:50:00 PM                Inj :   1
                                                   Inj Volume : 5.000 µl
    Acq. Method     : E:\DATA\XSM-In33\In33 181205 04-1 234-Cl 2018-12-05 16-44-00\In33 AS-98-0.
                      5mL-234nm-25min 234-Cl.M
    Last changed    : 12/5/2018 4:44:00 PM by SYSTEM
    Analysis Method : E:\DATA\XSM-In33\In33 181205 04-1 234-Cl 2018-12-05 16-44-00\In33 AS-98-0.
                      5mL-234nm-25min 234-Cl.M (Sequence Method)
    Last changed    : 1/2/2019 5:47:12 PM by SYSTEM
                      (modified after loading)
    Additional Info : Peak(s) manually integrated
```

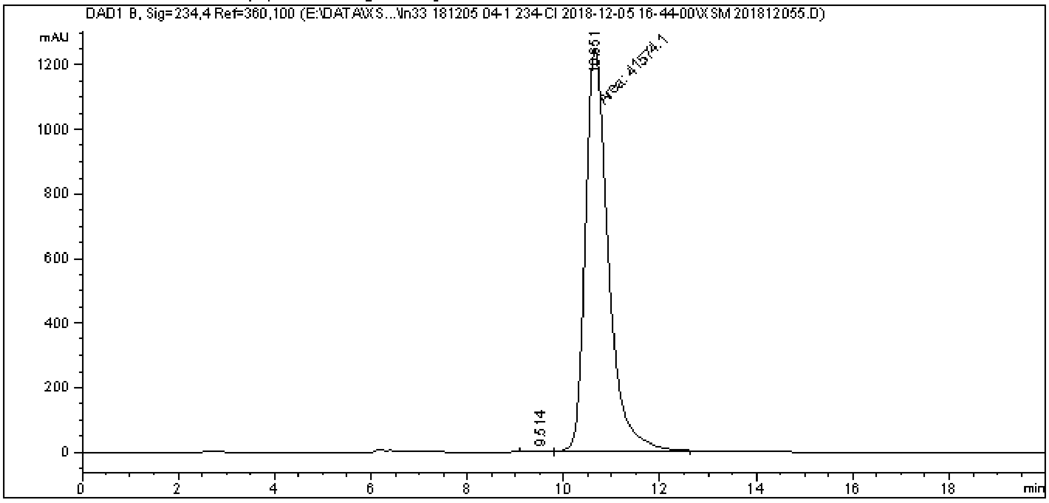


```
    =====================================================================
                             Area Percent Report
    =====================================================================

    Sorted By             :      Signal
    Multiplier            :      1.0000
    Dilution              :      1.0000
    Do not use Multiplier & Dilution Factor with ISTDs


    Signal 1: DAD1 B, Sig=234,4 Ref=360,100

    Peak RetTime Type  Width     Area      Height     Area
      #   [min]        [min]   [mAU*s]     [mAU]        %
    ----|-------|----|-------|----------|----------|--------|
       1   9.514 BB    0.2212   41.83960    2.35537   0.1005
       2  10.651 MF    0.5566 4.15741e4 1244.78259  99.8995

    Totals :                  4.16159e4  1247.13796


    =====================================================================
                              *** End of Report ***
```

1260 1/2/2019 5:47:28 PM SYSTEM Page 1 of 1

Supplementary Figure 145. HPLC spectrum of (1*R*,3*S*,4*R*)-**3i**

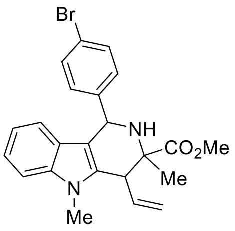

(1*R**,3*S**,4*R**)-**3j**

```
Data File E:\DATA\XSM\In33 180530 03-37 4-Br-N-F-T 2018-05-30 16-04-37\XSM 180530.D
Sample Name: In33 03-37 4-Br 1st rac

   =====================================================================
   Acq. Operator   : SYSTEM                         Seq. Line :    1
   Acq. Instrument : 1260                            Location :   13
   Injection Date  : 5/30/2018 4:05:33 PM                 Inj :    1
                                                   Inj Volume : 5.000 µl
   Acq. Method     : E:\DATA\XSM\In33 180530 03-37 4-Br-N-F-T 2018-05-30 16-04-37\In33 AS-98-2-
                     234nm-20min-noD.M
   Last changed    : 5/30/2018 4:04:37 PM by SYSTEM
   Analysis Method : E:\DATA\XSM\In33 180530 03-37 4-Br-N-F-T 2018-05-30 16-04-37\In33 AS-98-2-
                     234nm-20min-noD.M (Sequence Method)
   Last changed    : 10/8/2018 3:01:12 PM by SYSTEM
                     (modified after loading)
   Additional Info : Peak(s) manually integrated
```

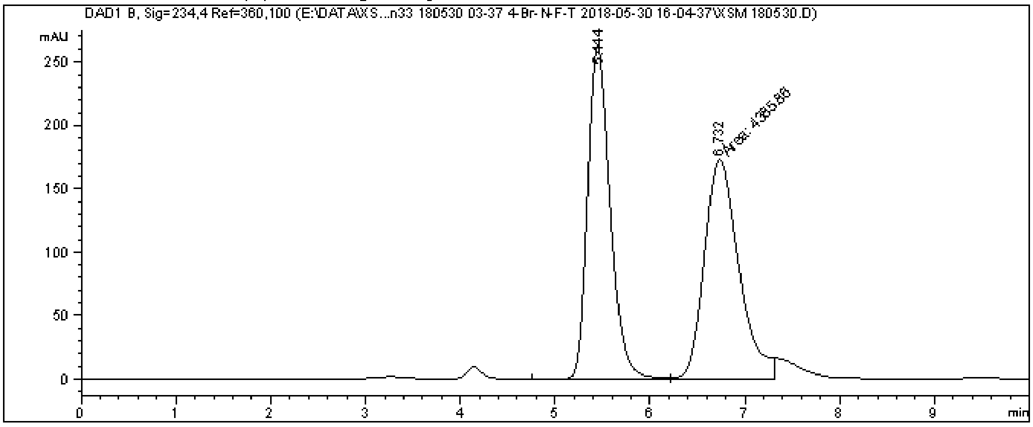


```
   =====================================================================
                            Area Percent Report
   =====================================================================

   Sorted By             :      Signal
   Multiplier            :      1.0000
   Dilution              :      1.0000
   Do not use Multiplier & Dilution Factor with ISTDs


   Signal 1: DAD1 B, Sig=234,4 Ref=360,100

   Peak RetTime Type  Width     Area      Height     Area
     #   [min]        [min]   [mAU*s]     [mAU]        %
   ----|-------|----|-------|----------|----------|--------|
      1   5.444 BV    0.2547 4331.99512  262.23544  49.6910
      2   6.732 MF    0.4225 4385.86426  173.00990  50.3090

   Totals :                  8717.85938  435.24535


   =====================================================================
                             *** End of Report ***
```

1260 10/8/2018 3:01:17 PM SYSTEM   Page 1 of 1

Supplementary Figure 146. HPLC spectrum of (1*R**,3*S**,4*R**)-**3j**

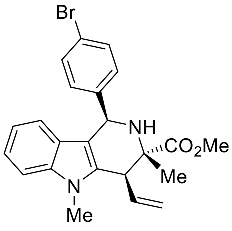

(1*R*,3*S*,4*R*)-**3j**

```
Data File E:\DATA\XSM\In33 180530 03-37 4-Br-N-F-T 2018-05-30 16-04-37\XSM 1805301.D
Sample Name: In33 03-34 4-Br R+L opt

    =====================================================================
    Acq. Operator   : SYSTEM                          Seq. Line :    2
    Acq. Instrument : 1260                             Location :   14
    Injection Date  : 5/30/2018 4:26:27 PM                  Inj :    1
                                                     Inj Volume : 5.000 µl
    Acq. Method     : E:\DATA\XSM\In33 180530 03-37 4-Br-N-F-T 2018-05-30 16-04-37\In33 AS-98-2-
                      234nm-20min-noD.M
    Last changed    : 5/30/2018 4:04:37 PM by SYSTEM
    Analysis Method : E:\DATA\XSM\In33 180530 03-37 4-Br-N-F-T 2018-05-30 16-04-37\In33 AS-98-2-
                      234nm-20min-noD.M (Sequence Method)
    Last changed    : 10/8/2018 3:01:12 PM by SYSTEM
                      (modified after loading)
    Additional Info : Peak(s) manually integrated
```

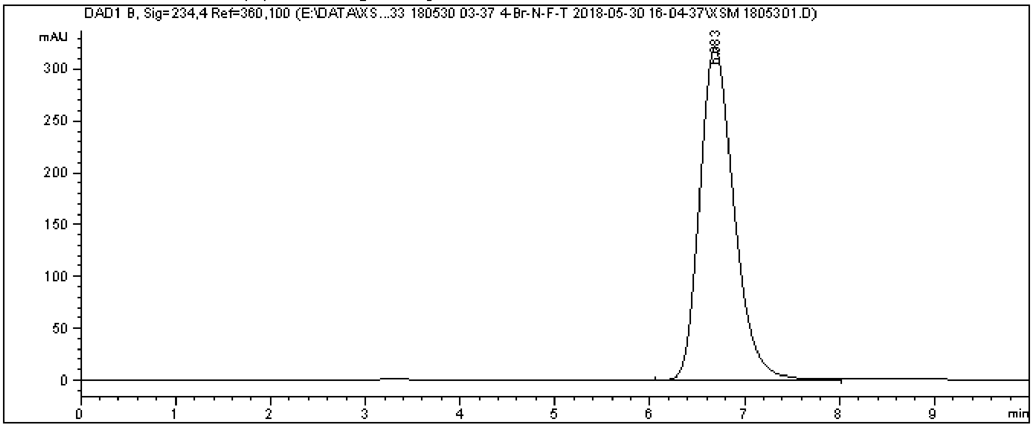


```
    =====================================================================
                             Area Percent Report
    =====================================================================

    Sorted By             :      Signal
    Multiplier            :      1.0000
    Dilution              :      1.0000
    Do not use Multiplier & Dilution Factor with ISTDs


    Signal 1: DAD1 B, Sig=234,4 Ref=360,100

    Peak RetTime Type  Width     Area      Height     Area
      #   [min]        [min]   [mAU*s]     [mAU]        %
    ----|-------|----|-------|----------|----------|--------|
       1   6.683 BB    0.3810 8027.47363  321.83548 100.0000

    Totals :                  8027.47363  321.83548


    =====================================================================
                              *** End of Report ***
```


Supplementary Figure 147. HPLC spectrum of (1*R*,3*S*,4*R*)-**3j**

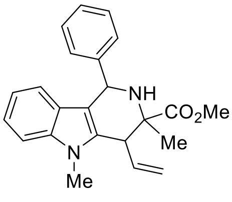

(1*R**,3*S**,4*R**)-**3k**

```
Data File E:\DATA\XSM\In33 180427 03-16 Ar-Ph 2018-04-27 21-46-08\XSM-20180427 Ar-Ph1.D
Sample Name: In33 03-16 Ar-Ph 1st rac

    =====================================================================
    Acq. Operator   : SYSTEM                        Seq. Line :   2
    Acq. Instrument : 1260                           Location :   19
    Injection Date  : 4/27/2018 10:12:58 PM               Inj :   1
                                                   Inj Volume : 5.000 µl
    Acq. Method     : E:\DATA\XSM\In33 180427 03-16 Ar-Ph 2018-04-27 21-46-08\In33 1st rac 0.5mL
                      234nm noD.M
    Last changed    : 4/27/2018 9:46:08 PM by SYSTEM
    Analysis Method : E:\DATA\XSM\In33 180427 03-16 Ar-Ph 2018-04-27 21-46-08\In33 1st rac 0.5mL
                      234nm noD.M (Sequence Method)
    Last changed    : 10/8/2018 2:44:20 PM by SYSTEM
                      (modified after loading)
    Additional Info : Peak(s) manually integrated
```

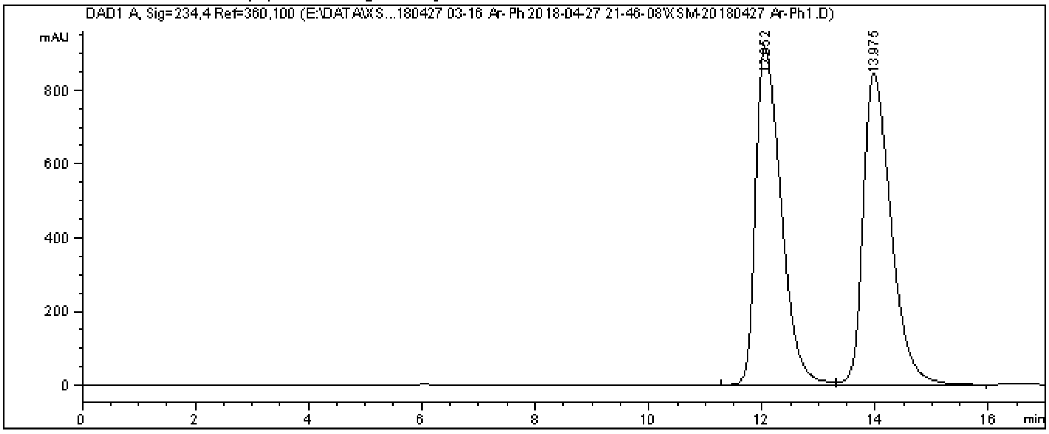


```
    =====================================================================
                             Area Percent Report
    =====================================================================

    Sorted By             :      Signal
    Multiplier            :      1.0000
    Dilution              :      1.0000
    Do not use Multiplier & Dilution Factor with ISTDs


    Signal 1: DAD1 A, Sig=234,4 Ref=360,100

    Peak RetTime Type  Width     Area      Height     Area
      #   [min]        [min]   [mAU*s]     [mAU]        %
    ----|-------|----|-------|----------|----------|--------|
       1  12.052 BV    0.4986 2.86482e4   916.64539  49.8368
       2  13.975 VB    0.5341 2.88358e4   845.15515  50.1632

    Totals :                  5.74840e4  1761.80054


    =====================================================================
                              *** End of Report ***
```

1260 10/8/2018 2:44:24 PM SYSTEM Page 1 of 1

Supplementary Figure 148. HPLC spectrum of (1*R**,3*S**,4*R**)-**3k**

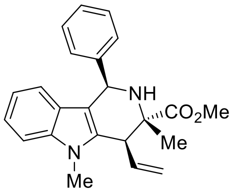

(1*R*,3*S*,4*R*)-**3k**

```
Data File E:\DATA\XSM\In33 180427 03-16 Ar-Ph 2018-04-27 21-46-08\XSM-20180427 Ar-Ph.D
Sample Name: In33 03-16 Ar-Ph L+R opt

    =====================================================================
    Acq. Operator   : SYSTEM                        Seq. Line :    1
    Acq. Instrument : 1260                           Location :   18
    Injection Date  : 4/27/2018 9:47:03 PM                Inj :    1
                                                  Inj Volume : 5.000 µl
    Acq. Method     : E:\DATA\XSM\In33 180427 03-16 Ar-Ph 2018-04-27 21-46-08\In33 1st rac 0.5mL
                      234nm noD.M
    Last changed    : 4/27/2018 9:46:08 PM by SYSTEM
    Analysis Method : E:\DATA\XSM\In33 180427 03-16 Ar-Ph 2018-04-27 21-46-08\In33 1st rac 0.5mL
                      234nm noD.M (Sequence Method)
    Last changed    : 10/8/2018 2:44:20 PM by SYSTEM
                      (modified after loading)
    Additional Info : Peak(s) manually integrated
```

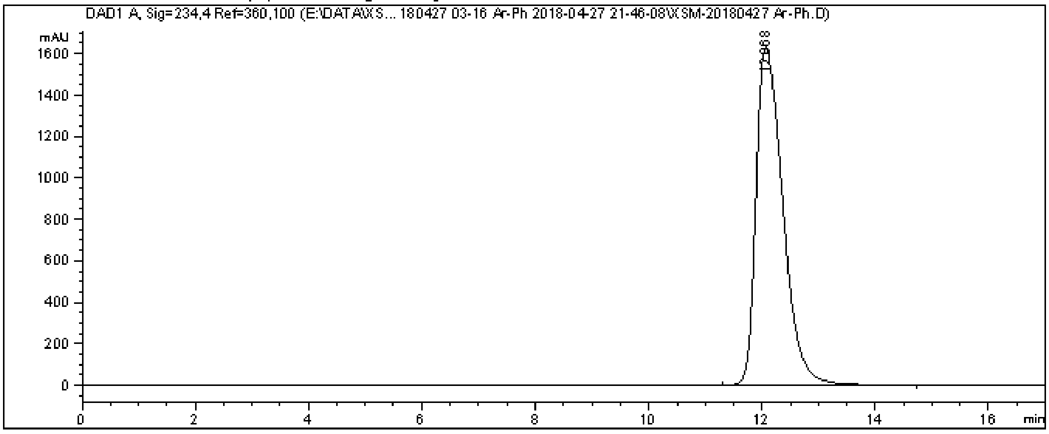


```
    =====================================================================
                             Area Percent Report
    =====================================================================

    Sorted By             :      Signal
    Multiplier            :      1.0000
    Dilution              :      1.0000
    Do not use Multiplier & Dilution Factor with ISTDs


    Signal 1: DAD1 A, Sig=234,4 Ref=360,100

    Peak RetTime Type  Width     Area      Height     Area
      #   [min]        [min]   [mAU*s]     [mAU]        %
    ----|-------|----|-------|----------|----------|--------|
       1  12.068 BB    0.5253 5.35665e4  1630.49695 100.0000

    Totals :                  5.35665e4  1630.49695


    =====================================================================
                              *** End of Report ***
```


Supplementary Figure 149. HPLC spectrum of (1*R*,3*S*,4*R*)-**3k**

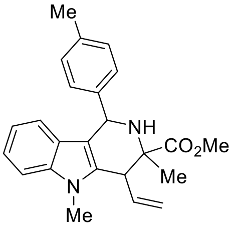

(1*R**,3*S**,4*R**)-**3l**

```
Data File E:\DATA\XSM\In33 180521 03-30 24-MeC6H4 2018-05-21 11-01-15\XSM 03-30.D
Sample Name: In33 03-30 4-Me 1st rac

    =====================================================================
    Acq. Operator   : SYSTEM                        Seq. Line :    1
    Acq. Instrument : 1260                           Location :   15
    Injection Date  : 5/21/2018 11:02:11 AM               Inj :    1
                                                   Inj Volume : 5.000 µl
    Acq. Method     : E:\DATA\XSM\In33 180521 03-30 24-MeC6H4 2018-05-21 11-01-15\In33 AS-98-2-
                      254nm-20min-noD 24-MeC6H4.M
    Last changed    : 5/21/2018 11:01:16 AM by SYSTEM
    Analysis Method : E:\DATA\XSM\In33 180521 03-30 24-MeC6H4 2018-05-21 11-01-15\In33 AS-98-2-
                      254nm-20min-noD 24-MeC6H4.M (Sequence Method)
    Last changed    : 10/8/2018 2:56:33 PM by SYSTEM
                      (modified after loading)
    Additional Info : Peak(s) manually integrated
```

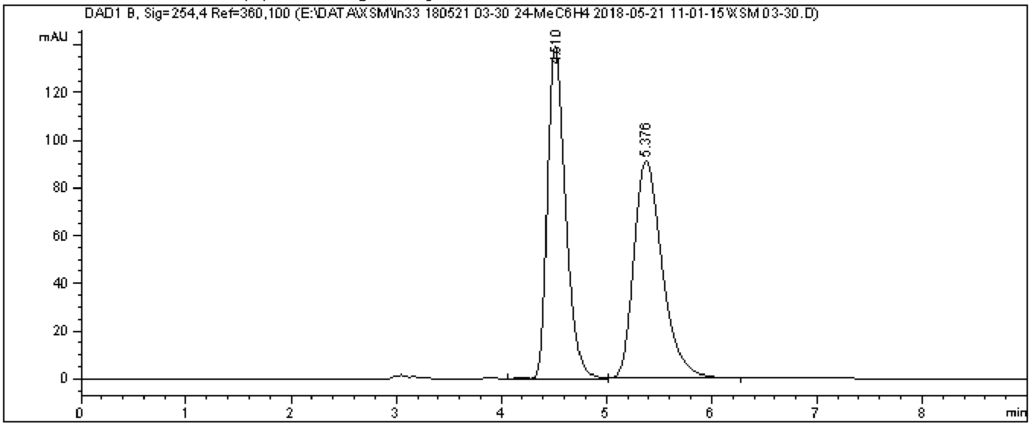


```
    =====================================================================
                             Area Percent Report
    =====================================================================

    Sorted By             :      Signal
    Multiplier            :      1.0000
    Dilution              :      1.0000
    Do not use Multiplier & Dilution Factor with ISTDs


    Signal 1: DAD1 B, Sig=254,4 Ref=360,100

    Peak RetTime Type  Width     Area      Height     Area
      #   [min]        [min]   [mAU*s]     [mAU]        %
    ----|-------|----|-------|----------|----------|--------|
       1   4.510 VV R  0.1836 1667.88477  139.35078  49.5538
       2   5.376 VB    0.2858 1697.92041   90.93706  50.4462

    Totals :                  3365.80518  230.28784


    =====================================================================
                              *** End of Report ***
```


Supplementary Figure 150. HPLC spectrum of (1*R**,3*S**,4*R**)-**3l**

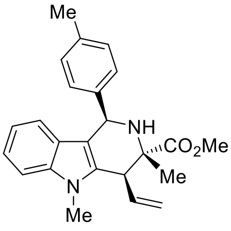

(1*R*,3*S*,4*R*)-**3l**

```
Data File E:\DATA\XSM\In33 180521 03-30 24-MeC6H4 2018-05-21 11-01-15\XSM 03-301.D
Sample Name: In33 03-30 4-Me R+L opt

    =====================================================================
    Acq. Operator   : SYSTEM                          Seq. Line :    2
    Acq. Instrument : 1260                             Location :   16
    Injection Date  : 5/21/2018 11:23:05 AM                 Inj :    1
                                                     Inj Volume : 5.000 µl
    Acq. Method     : E:\DATA\XSM\In33 180521 03-30 24-MeC6H4 2018-05-21 11-01-15\In33 AS-98-2-
                      254nm-20min-noD 24-MeC6H4.M
    Last changed    : 5/21/2018 11:01:16 AM by SYSTEM
    Analysis Method : E:\DATA\XSM\In33 180521 03-30 24-MeC6H4 2018-05-21 11-01-15\In33 AS-98-2-
                      254nm-20min-noD 24-MeC6H4.M (Sequence Method)
    Last changed    : 10/8/2018 2:56:33 PM by SYSTEM
                      (modified after loading)
    Additional Info : Peak(s) manually integrated
```

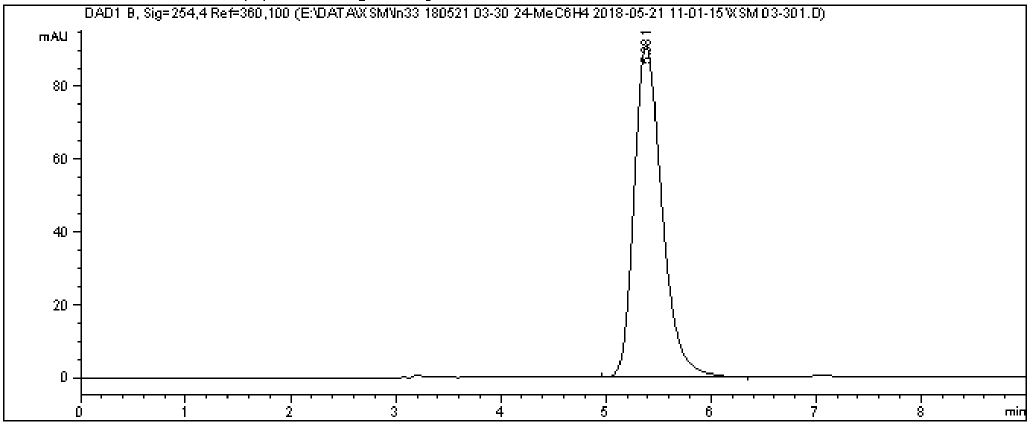


```
    =====================================================================
                             Area Percent Report
    =====================================================================

    Sorted By             :      Signal
    Multiplier            :      1.0000
    Dilution              :      1.0000
    Do not use Multiplier & Dilution Factor with ISTDs


    Signal 1: DAD1 B, Sig=254,4 Ref=360,100

    Peak RetTime Type  Width     Area      Height     Area
      #   [min]        [min]   [mAU*s]     [mAU]        %
    ----|-------|----|-------|----------|----------|--------|
       1   5.381 BB    0.2827 1671.62366   90.83202 100.0000

    Totals :                  1671.62366   90.83202


    =====================================================================
                              *** End of Report ***
```

1260 10/8/2018 2:56:46 PM SYSTEM Page 1 of 1

Supplementary Figure 151. HPLC spectrum of (1*R*,3*S*,4*R*)-**3l**

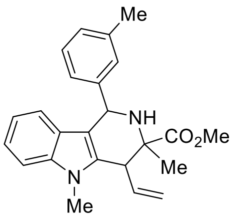

(1*R**,3*S**,4*R**)-**3m**

```
Data File E:\DATA\XSM\In33 180520 03-30 234-Me 2018-05-20 21-58-45\XSM 03-302.D
Sample Name: In33 03-30 3-Me 1st rac

    =====================================================================
    Acq. Operator   : SYSTEM                        Seq. Line :   3
    Acq. Instrument : 1260                           Location :   17
    Injection Date  : 5/20/2018 11:01:32 PM               Inj :   1
                                                Inj Volume : 5.000 µl
    Acq. Method     : E:\DATA\XSM\In33 180520 03-30 234-Me 2018-05-20 21-58-45\In33 1st 0.5mL
                      234nm 30min noD.M
    Last changed    : 5/20/2018 9:58:45 PM by SYSTEM
    Analysis Method : E:\DATA\XSM\In33 180520 03-30 234-Me 2018-05-20 21-58-45\In33 1st 0.5mL
                      234nm 30min noD.M (Sequence Method)
    Last changed    : 10/8/2018 2:55:00 PM by SYSTEM
                      (modified after loading)
    Additional Info : Peak(s) manually integrated
```

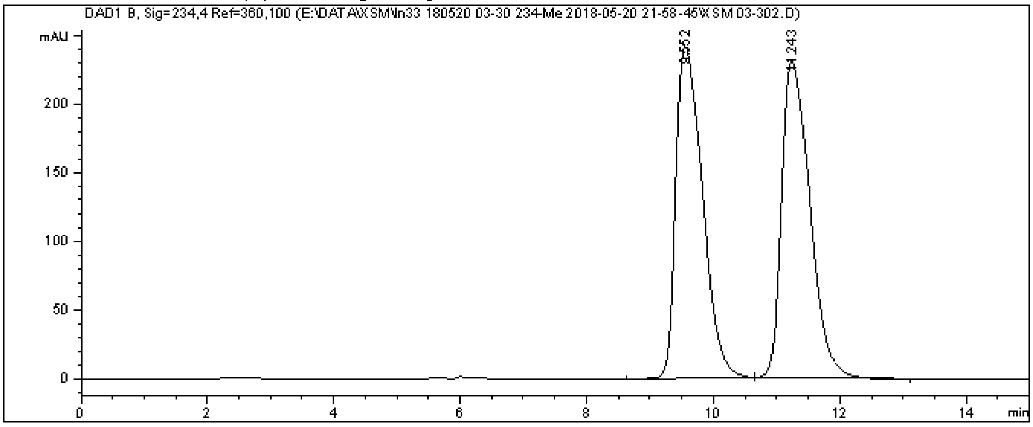


```
    =====================================================================
                         Area Percent Report
    =====================================================================

    Sorted By             :      Signal
    Multiplier            :      1.0000
    Dilution              :      1.0000
    Do not use Multiplier & Dilution Factor with ISTDs


    Signal 1: DAD1 B, Sig=234,4 Ref=360,100

    Peak RetTime Type  Width     Area      Height     Area
      #   [min]        [min]   [mAU*s]     [mAU]        %
    ----|-------|----|-------|----------|----------|--------|
       1   9.552 BB    0.4792 7064.94580  241.58951  49.7243
       2  11.243 BB    0.5015 7143.29785  230.54137  50.2757

    Totals :                  1.42082e4   472.13087


    =====================================================================
                          *** End of Report ***
```

1260 10/8/2018 2:55:15 PM SYSTEM   Page 1 of 1

Supplementary Figure 152. HPLC spectrum of (1*R**,3*S**,4*R**)-**3m**

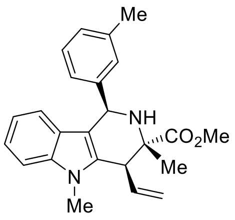

(1*R*,3*S*,4*R*)-**3m**

```
Data File E:\DATA\XSM\In33 180520 03-30 234-Me 2018-05-20 21-58-45\XSM 03-303.D
Sample Name: In33 03-29 3-Me R+L opt

    =====================================================================
    Acq. Operator   : SYSTEM                          Seq. Line :    4
    Acq. Instrument : 1260                             Location :   18
    Injection Date  : 5/20/2018 11:32:30 PM                 Inj :    1
                                                     Inj Volume : 5.000 µl
    Acq. Method     : E:\DATA\XSM\In33 180520 03-30 234-Me 2018-05-20 21-58-45\In33 1st 0.5mL
                      234nm 30min noD.M
    Last changed    : 5/20/2018 9:58:45 PM by SYSTEM
    Analysis Method : E:\DATA\XSM\In33 180520 03-30 234-Me 2018-05-20 21-58-45\In33 1st 0.5mL
                      234nm 30min noD.M (Sequence Method)
    Last changed    : 10/8/2018 2:55:00 PM by SYSTEM
                      (modified after loading)
    Additional Info : Peak(s) manually integrated
```

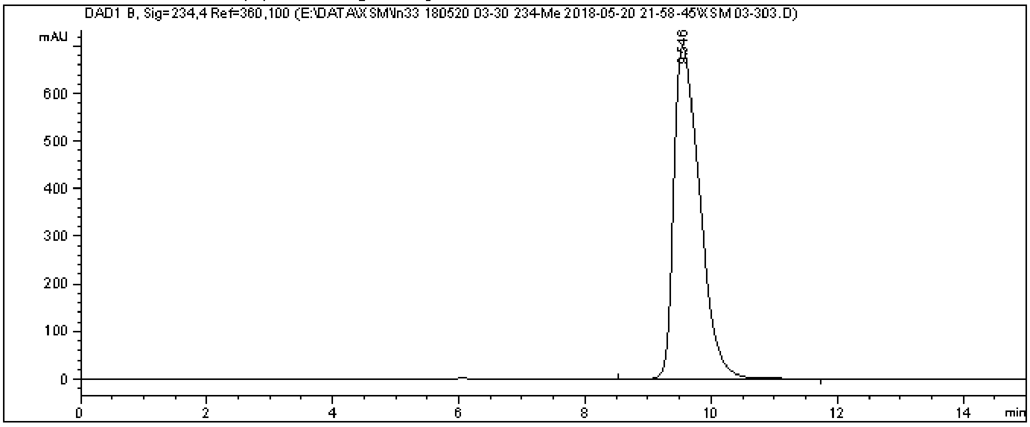


```
    =====================================================================
                         Area Percent Report
    =====================================================================

    Sorted By             :      Signal
    Multiplier            :      1.0000
    Dilution              :      1.0000
    Do not use Multiplier & Dilution Factor with ISTDs


    Signal 1: DAD1 B, Sig=234,4 Ref=360,100

    Peak RetTime Type  Width     Area      Height     Area
      #   [min]        [min]   [mAU*s]     [mAU]        %
    ----|-------|----|-------|----------|----------|--------|
       1   9.546 BB    0.4467 2.00753e4   699.69135 100.0000

    Totals :                  2.00753e4   699.69135


    =====================================================================
                          *** End of Report ***
```

1260 10/8/2018 2:55:04 PM SYSTEM   Page 1 of 1

Supplementary Figure 153. HPLC spectrum of (1*R*,3*S*,4*R*)-**3m**

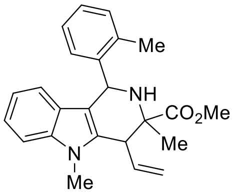

(1*R**,3*S**,4*R**)-**3n**

```
Data File E:\DATA\XS...3 190101 03-30 2-Me IC-IA-0.5mL 2019-01-01 22-35-13\XSM 201901012.D
Sample Name: In33 03-30 2-Me 1st rac IC-IA

    =====================================================================
    Acq. Operator   : SYSTEM                        Seq. Line :    3
    Acq. Instrument : 1260                           Location :   80
    Injection Date  : 1/1/2019 11:19:01 PM                Inj :    1
                                                   Inj Volume : 5.000 µl
    Acq. Method     : E:\DATA\XSM\In33 190101 03-30 2-Me IC-IA-0.5mL 2019-01-01 22-35-13\In33 IC-
                      IA 99-0.5mL-30min-234nm 2-Me rac.M
    Last changed    : 1/1/2019 10:35:13 PM by SYSTEM
    Analysis Method : E:\DATA\XSM\In33 190101 03-30 2-Me IC-IA-0.5mL 2019-01-01 22-35-13\In33 IC-
                      IA 99-0.5mL-30min-234nm 2-Me rac.M (Sequence Method)
    Last changed    : 1/2/2019 10:46:28 AM by SYSTEM
                      (modified after loading)
    Additional Info : Peak(s) manually integrated
```

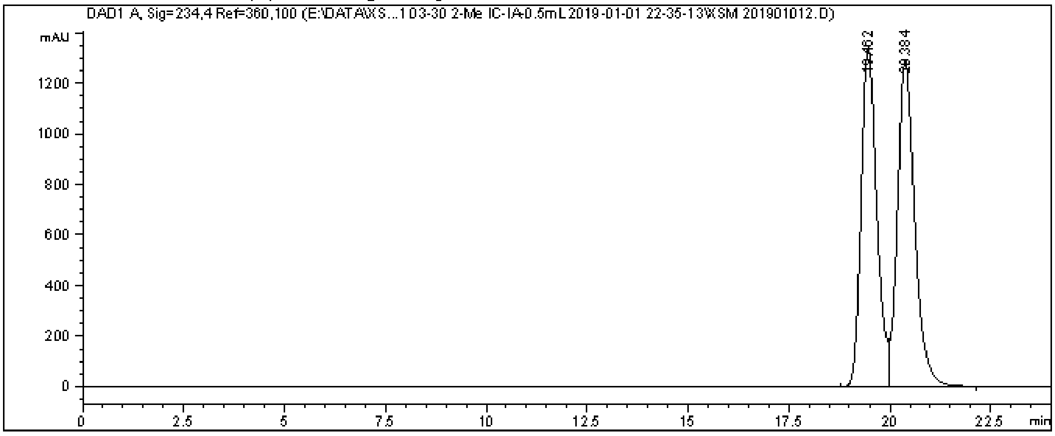


```
    =====================================================================
                             Area Percent Report
    =====================================================================

    Sorted By             :      Signal
    Multiplier            :      1.0000
    Dilution              :      1.0000
    Do not use Multiplier & Dilution Factor with ISTDs


    Signal 1: DAD1 A, Sig=234,4 Ref=360,100

    Peak RetTime Type  Width     Area      Height     Area
      #   [min]        [min]   [mAU*s]     [mAU]        %
    ----|-------|----|-------|----------|----------|--------|
       1  19.462 BV    0.4037 3.60135e4  1343.62476  48.3725
       2  20.384 VB    0.4490 3.84368e4  1292.40662  51.6275

    Totals :                  7.44502e4  2636.03137


    =====================================================================
                             *** End of Report ***
```

1260 1/2/2019 10:46:33 AM SYSTEM   Page 1 of 1

Supplementary Figure 154. HPLC spectrum of (1*R**,3*S**,4*R**)-**3n**

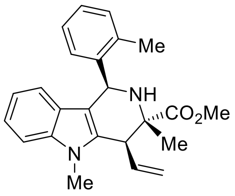

(1*R*,3*S*,4*R*)-**3n**

Data File E:\DATA\XS...33 190101 03-30 2-Me IC-IA-0.5mL 2019-01-01 22-35-13\XSM 20190101.D
Sample Name: In33 03-30 2-Me R+L opt IC-IA

```
=====================================================================
Acq. Operator   : SYSTEM                        Seq. Line :   1
Acq. Instrument : 1260                           Location :   79
Injection Date  : 1/1/2019 10:36:37 PM                Inj :   1
                                               Inj Volume : 5.000 µl
Acq. Method     : E:\DATA\XSM\In33 190101 03-30 2-Me IC-IA-0.5mL 2019-01-01 22-35-13\In33 IC-
                  IA 99-0.5mL-30min-234nm 2-Me rac.M
Last changed    : 1/1/2019 10:35:13 PM by SYSTEM
Analysis Method : E:\DATA\XSM\In33 190101 03-30 2-Me IC-IA-0.5mL 2019-01-01 22-35-13\In33 IC-
                  IA 99-0.5mL-30min-234nm 2-Me rac.M (Sequence Method)
Last changed    : 1/2/2019 10:46:28 AM by SYSTEM
                  (modified after loading)
Additional Info : Peak(s) manually integrated
```

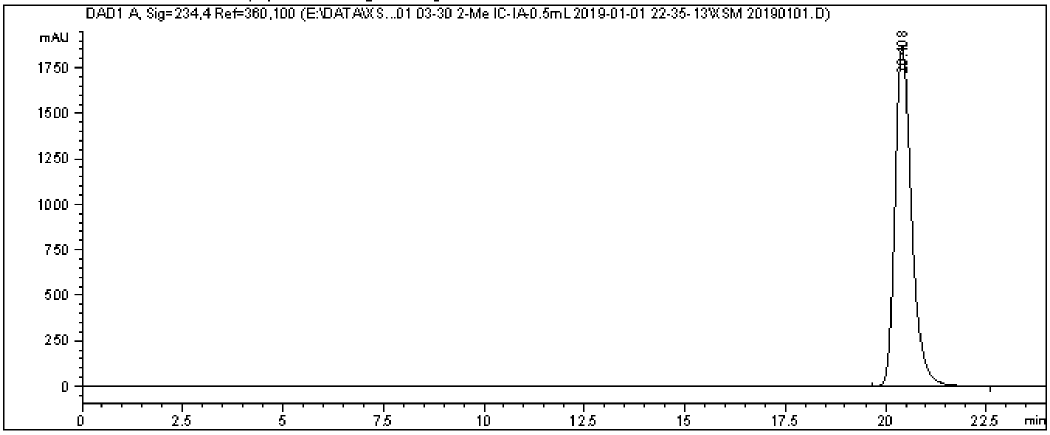


```
=====================================================================
                         Area Percent Report
=====================================================================

Sorted By             :      Signal
Multiplier            :      1.0000
Dilution              :      1.0000
Do not use Multiplier & Dilution Factor with ISTDs


Signal 1: DAD1 A, Sig=234,4 Ref=360,100

Peak RetTime Type  Width     Area      Height     Area
  #   [min]        [min]   [mAU*s]     [mAU]        %
----|-------|----|-------|----------|----------|--------|
   1  20.408 BB    0.3919 5.39703e4  1863.31104 100.0000

Totals :                  5.39703e4  1863.31104


=====================================================================
                          *** End of Report ***
```

1260 1/2/2019 10:46:59 AM SYSTEM Page 1 of 1

Supplementary Figure 155. HPLC spectrum of (1*R*,3*S*,4*R*)-**3n**

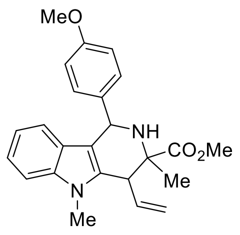

(1*R**,3*S**,4*R**)-**3o**

Data File E:\DATA\XSM\In33 180529 4MeO-Fur-Thien 2018-05-29 16-49-01\XSM 180529.D
Sample Name: In33 03-37 4-MeO 1st rac

```
=====================================================================
Acq. Operator   : SYSTEM                        Seq. Line :   1
Acq. Instrument : 1260                           Location :   15
Injection Date  : 5/29/2018 4:50:01 PM                Inj :   1
                                               Inj Volume : 5.000 µl
Acq. Method     : E:\DATA\XSM\In33 180529 4MeO-Fur-Thien 2018-05-29 16-49-01\In33 1st rac 0.
                  5mL 234nm noD.M
Last changed    : 5/29/2018 4:49:02 PM by SYSTEM
Analysis Method : E:\DATA\XSM\In33 180529 4MeO-Fur-Thien 2018-05-29 16-49-01\In33 1st rac 0.
                  5mL 234nm noD.M (Sequence Method)
Last changed    : 10/8/2018 2:46:26 PM by SYSTEM
                  (modified after loading)
Additional Info : Peak(s) manually integrated
```

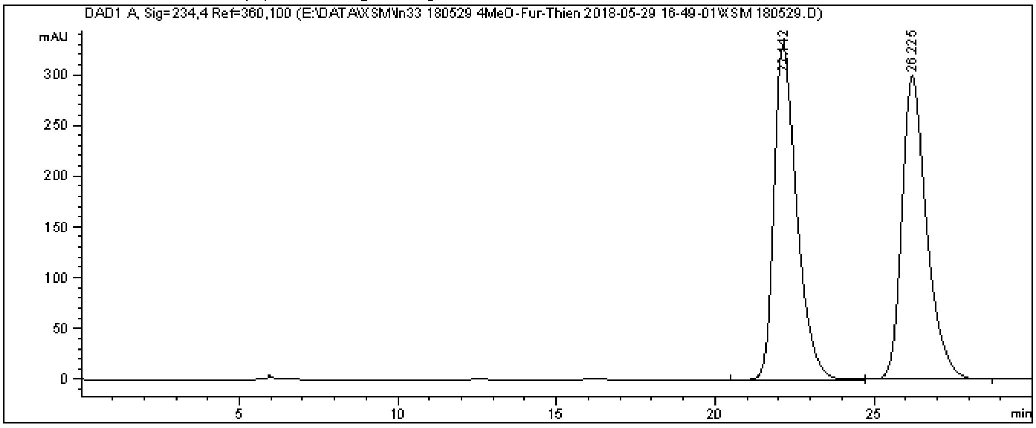


```
=====================================================================
                         Area Percent Report
=====================================================================

Sorted By             :      Signal
Multiplier            :      1.0000
Dilution              :      1.0000
Do not use Multiplier & Dilution Factor with ISTDs


Signal 1: DAD1 A, Sig=234,4 Ref=360,100

Peak RetTime Type  Width     Area      Height     Area
  #   [min]        [min]   [mAU*s]     [mAU]        %
----|-------|----|-------|----------|----------|--------|
   1  22.142 BB    0.7523 1.64949e4   328.21844  50.3150
   2  26.225 BB    0.8216 1.62884e4   298.95908  49.6850

Totals :                  3.27833e4   627.17752


=====================================================================
                          *** End of Report ***
```

1260 10/8/2018 2:47:04 PM SYSTEM Page 1 of 1

Supplementary Figure 156. HPLC spectrum of (1*R**,3*S**,4*R**)-**3o**

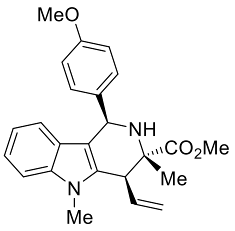

(1*R*,3*S*,4*R*)-**3o**

```
Data File E:\DATA\XSM\In33 180529 4MeO-Fur-Thien 2018-05-29 16-49-01\XSM 1805291.D
Sample Name: In33 03-36 4-MeO R+L opt

    =====================================================================
    Acq. Operator   : SYSTEM                          Seq. Line :    2
    Acq. Instrument : 1260                             Location :   16
    Injection Date  : 5/29/2018 5:20:57 PM                  Inj :    1
                                                     Inj Volume : 5.000 µl
    Acq. Method     : E:\DATA\XSM\In33 180529 4MeO-Fur-Thien 2018-05-29 16-49-01\In33 1st rac 0.
                      5mL 234nm noD.M
    Last changed    : 5/29/2018 4:49:02 PM by SYSTEM
    Analysis Method : E:\DATA\XSM\In33 180529 4MeO-Fur-Thien 2018-05-29 16-49-01\In33 1st rac 0.
                      5mL 234nm noD.M (Sequence Method)
    Last changed    : 10/8/2018 2:46:26 PM by SYSTEM
                      (modified after loading)
    Additional Info : Peak(s) manually integrated
```

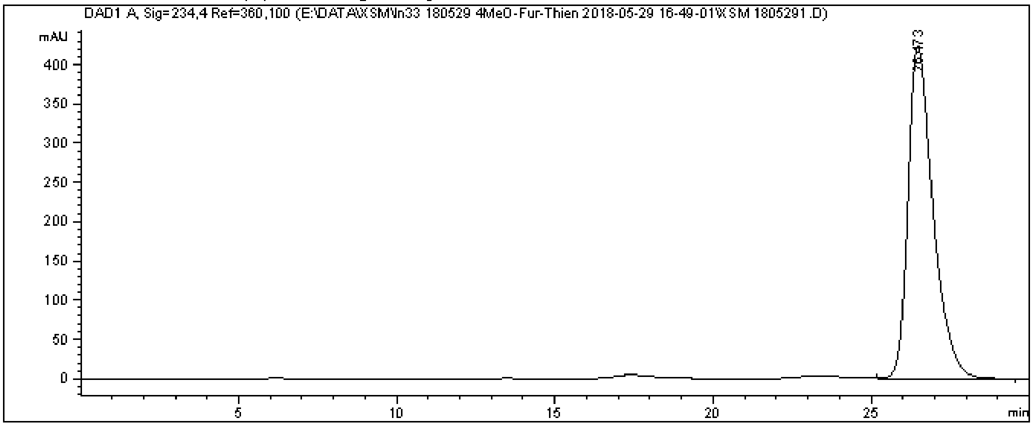


```
    =====================================================================
                             Area Percent Report
    =====================================================================

    Sorted By             :      Signal
    Multiplier            :      1.0000
    Dilution              :      1.0000
    Do not use Multiplier & Dilution Factor with ISTDs


    Signal 1: DAD1 A, Sig=234,4 Ref=360,100

    Peak RetTime Type  Width     Area      Height     Area
      #   [min]        [min]   [mAU*s]     [mAU]        %
    ----|-------|----|-------|----------|----------|--------|
       1  26.473 BB    0.8327 2.33045e4  423.06076 100.0000

    Totals :                  2.33045e4  423.06076


    =====================================================================
                              *** End of Report ***
```

1260 10/8/2018 2:46:28 PM SYSTEM Page 1 of 1

Supplementary Figure 157. HPLC spectrum of (1*R*,3*S*,4*R*)-**3o**

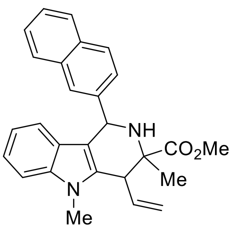

(1*R**,3*S**,4*R**)-**3p**

Data File E:\DATA\XSM\In33 190103 03-36 Naphthyl 1st 2019-01-03 10-25-13\XSM 201901022.D
Sample Name: In33 03-37 Naphthyl 1st rac

```
=====================================================================
Acq. Operator   : SYSTEM                          Seq. Line :   3
Acq. Instrument : 1260                             Location :   70
Injection Date  : 1/3/2019 10:58:14 AM                  Inj :   1
                                                 Inj Volume : 5.000 µl
Acq. Method     : E:\DATA\XSM\In33 190103 03-36 Naphthyl 1st 2019-01-03 10-25-13\In33 AS-98-
                  254nm-20min Naphthyl 1st.M
Last changed    : 1/3/2019 10:25:14 AM by SYSTEM
Analysis Method : E:\DATA\XSM\In33 190103 03-36 Naphthyl 1st 2019-01-03 10-25-13\In33 AS-98-
                  254nm-20min Naphthyl 1st.M (Sequence Method)
Last changed    : 1/3/2019 11:36:17 AM by SYSTEM
                  (modified after loading)
Additional Info : Peak(s) manually integrated
```

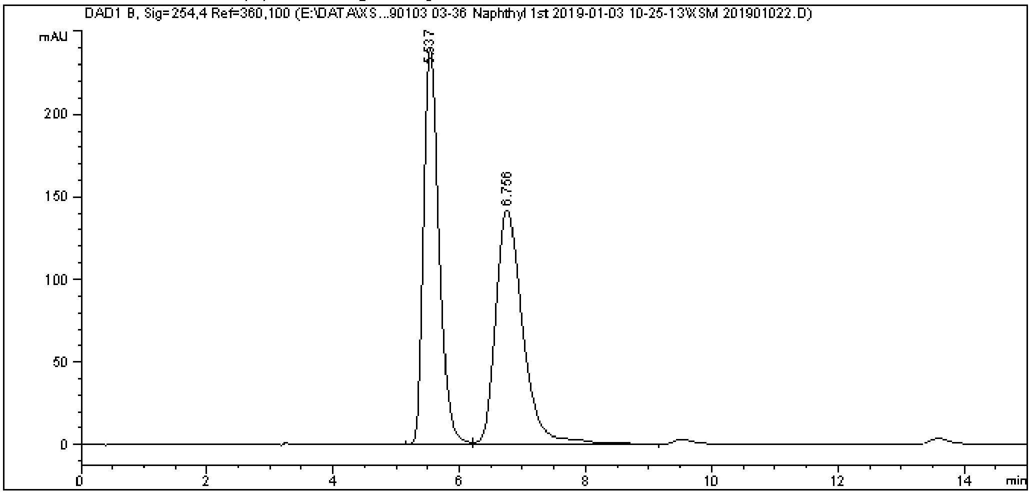


```
=====================================================================
                      Area Percent Report
=====================================================================

Sorted By             :      Signal
Multiplier            :      1.0000
Dilution              :      1.0000
Do not use Multiplier & Dilution Factor with ISTDs


Signal 1: DAD1 B, Sig=254,4 Ref=360,100

Peak RetTime Type  Width     Area      Height      Area
  #   [min]        [min]   [mAU*s]     [mAU]        %
----|-------|----|-------|----------|----------|--------|
   1   5.537 BV    0.2647 4113.77148  239.17046  49.2677
   2   6.756 VB    0.4562 4236.06104  141.91727  50.7323

Totals :                  8349.83252  381.08772


=====================================================================
                        *** End of Report ***
```

1260 1/3/2019 11:36:27 AM SYSTEM Page 1 of 1

Supplementary Figure 158. HPLC spectrum of (1*R**,3*S**,4*R**)-**3p**

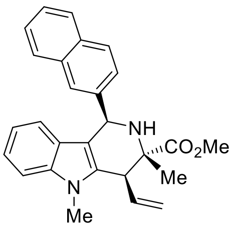

(1*R*,3*S*,4*R*)-**3p**

Data File E:\DATA\XSM\In33 190103 03-36 Naphthyl 1st 2019-01-03 10-25-13\XSM 20190102.D
Sample Name: In33 03-36 Naphthyl R+L opt

===

Acq. Operator : SYSTEM Seq. Line : 1
Acq. Instrument : 1260 Location : 69
Injection Date : 1/3/2019 10:26:13 AM Inj : 1
Inj Volume : 5.000 µl
Acq. Method : E:\DATA\XSM\In33 190103 03-36 Naphthyl 1st 2019-01-03 10-25-13\In33 AS-98-254nm-20min Naphthyl 1st.M
Last changed : 1/3/2019 10:25:14 AM by SYSTEM
Analysis Method : E:\DATA\XSM\In33 190103 03-36 Naphthyl 1st 2019-01-03 10-25-13\In33 AS-98-254nm-20min Naphthyl 1st.M (Sequence Method)
Last changed : 1/3/2019 11:36:17 AM by SYSTEM
(modified after loading)
Additional Info : Peak(s) manually integrated

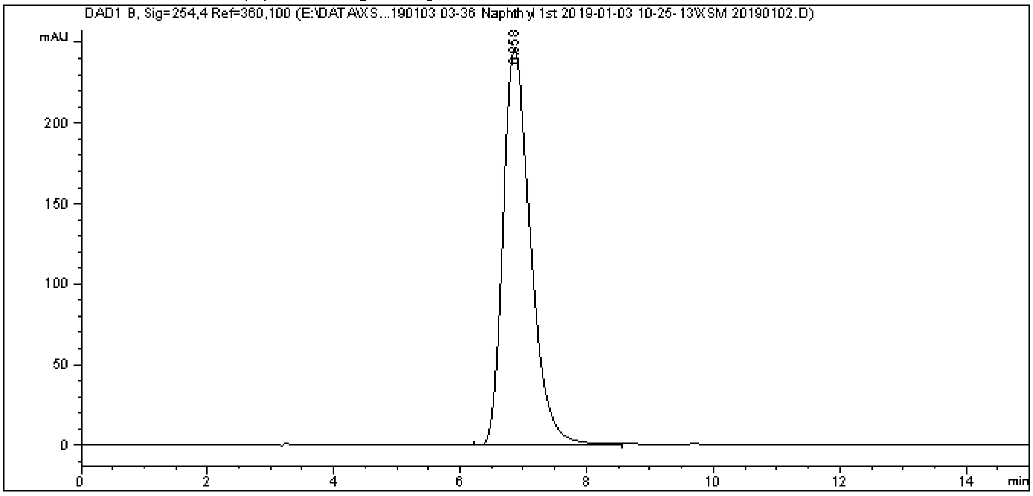

===

Area Percent Report

===

Sorted By : Signal
Multiplier : 1.0000
Dilution : 1.0000
Do not use Multiplier & Dilution Factor with ISTDs

Signal 1: DAD1 B, Sig=254,4 Ref=360,100

Peak #	RetTime [min]	Type	Width [min]	Area [mAU*s]	Height [mAU]	Area %
1	6.858	BB	0.4620	7405.11865	245.46161	100.0000

Totals : 7405.11865 245.46161

===

*** End of Report ***

1260 1/3/2019 11:36:40 AM SYSTEM Page 1 of 1

Supplementary Figure 159. HPLC spectrum of (1*R*,3*S*,4*R*)-**3p**

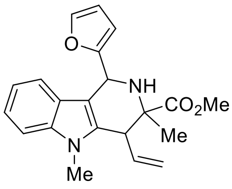

(1*R**,3*S**,4*R**)-**3q**

Data File E:\DATA\XSM\In33 180530 03-37 4-Br-N-F-T 2018-05-30 16-04-37\XSM 1805304.D
Sample Name: In33 03-37 Furyl 1st rac

===

Acq. Operator : SYSTEM Seq. Line : 5
Acq. Instrument : 1260 Location : 17
Injection Date : 5/30/2018 5:29:19 PM Inj : 1
Inj Volume : 5.000 µl
Acq. Method : E:\DATA\XSM\In33 180530 03-37 4-Br-N-F-T 2018-05-30 16-04-37\In33 AS-98-2-234nm-20min-noD.M
Last changed : 5/30/2018 4:04:37 PM by SYSTEM
Analysis Method : E:\DATA\XSM\In33 180530 03-37 4-Br-N-F-T 2018-05-30 16-04-37\In33 AS-98-2-234nm-20min-noD.M (Sequence Method)
Last changed : 10/7/2018 9:56:46 PM by SYSTEM
(modified after loading)
Additional Info : Peak(s) manually integrated

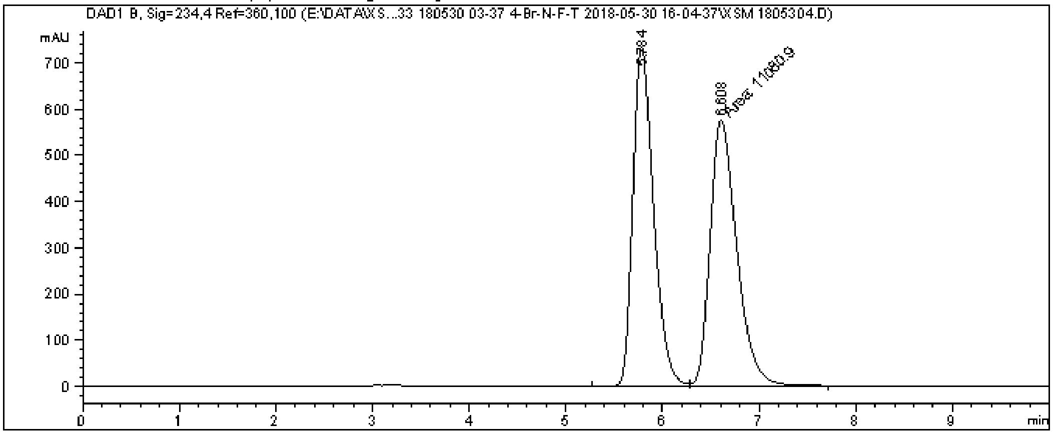

===
Area Percent Report
===

Sorted By : Signal
Multiplier : 1.0000
Dilution : 1.0000
Do not use Multiplier & Dilution Factor with ISTDs

Signal 1: DAD1 B, Sig=234,4 Ref=360,100

Peak #	RetTime [min]	Type	Width [min]	Area [mAU*s]	Height [mAU]	Area %
1	5.784	BV	0.2282	1.09642e4	734.11713	49.7352
2	6.608	MF	0.3208	1.10809e4	575.60492	50.2648

Totals : 2.20451e4 1309.72205

===
*** End of Report ***

1260 10/7/2018 9:57:28 PM SYSTEM Page 1 of 1

Supplementary Figure 160. HPLC spectrum of (1*R**,3*S**,4*R**)-**3q**

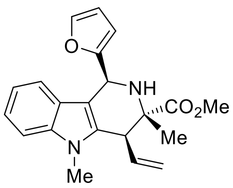

(1*R*,3*S*,4*R*)-**3q**

```
Data File E:\DATA\XSM\In33 180530 03-37 4-Br-N-F-T 2018-05-30 16-04-37\XSM 1805305.D
Sample Name: In33 03-36 Furyl R+L opt

    =====================================================================
    Acq. Operator   : SYSTEM                       Seq. Line :   6
    Acq. Instrument : 1260                          Location :   18
    Injection Date  : 5/30/2018 5:50:16 PM               Inj :   1
                                                  Inj Volume : 5.000 µl
    Acq. Method     : E:\DATA\XSM\In33 180530 03-37 4-Br-N-F-T 2018-05-30 16-04-37\In33 AS-98-2-
                      234nm-20min-noD.M
    Last changed    : 5/30/2018 4:04:37 PM by SYSTEM
    Analysis Method : E:\DATA\XSM\In33 180530 03-37 4-Br-N-F-T 2018-05-30 16-04-37\In33 AS-98-2-
                      234nm-20min-noD.M (Sequence Method)
    Last changed    : 10/7/2018 9:56:46 PM by SYSTEM
                      (modified after loading)
    Additional Info : Peak(s) manually integrated
```

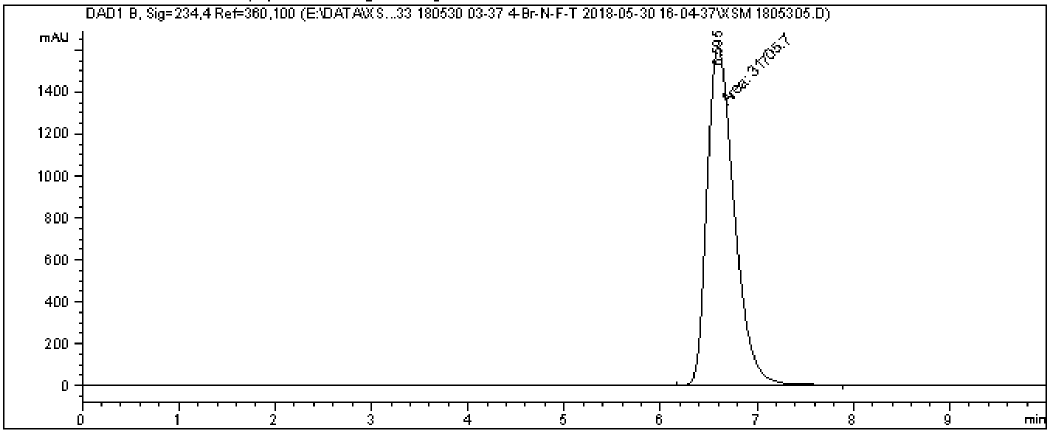


```
    =====================================================================
                          Area Percent Report
    =====================================================================

    Sorted By             :      Signal
    Multiplier            :      1.0000
    Dilution              :      1.0000
    Do not use Multiplier & Dilution Factor with ISTDs


    Signal 1: DAD1 B, Sig=234,4 Ref=360,100

    Peak RetTime Type  Width     Area      Height     Area
      #   [min]        [min]   [mAU*s]     [mAU]        %
    ----|-------|----|-------|----------|----------|--------|
       1   6.595 MF    0.3275 3.17057e4  1613.70227 100.0000

    Totals :                  3.17057e4  1613.70227


    =====================================================================
                             *** End of Report ***
```

1260 10/7/2018 9:56:51 PM SYSTEM   Page 1 of 1

Supplementary Figure 161. HPLC spectrum of (1*R*,3*S*,4*R*)-**3q**

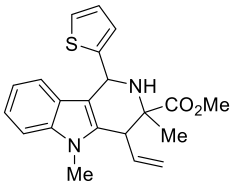

(1*R**,3*S**,4*R**)-**3r**

```
Data File E:\DATA\XSM\In33 180530 03-37 4-Br-N-F-T 2018-05-30 16-04-37\XSM 1805306.D
Sample Name: In33 03-37 Thienyl 1st rac

=====================================================================
Acq. Operator   : SYSTEM                         Seq. Line :    7
Acq. Instrument : 1260                            Location :   19
Injection Date  : 5/30/2018 6:11:11 PM                 Inj :    1
                                                Inj Volume : 5.000 µl
Acq. Method     : E:\DATA\XSM\In33 180530 03-37 4-Br-N-F-T 2018-05-30 16-04-37\In33 AS-98-2-
                  234nm-20min-noD.M
Last changed    : 5/30/2018 4:04:37 PM by SYSTEM
Analysis Method : E:\DATA\XSM\In33 180530 03-37 4-Br-N-F-T 2018-05-30 16-04-37\In33 AS-98-2-
                  234nm-20min-noD.M (Sequence Method)
Last changed    : 10/8/2018 3:09:43 PM by SYSTEM
                  (modified after loading)
Additional Info : Peak(s) manually integrated
```

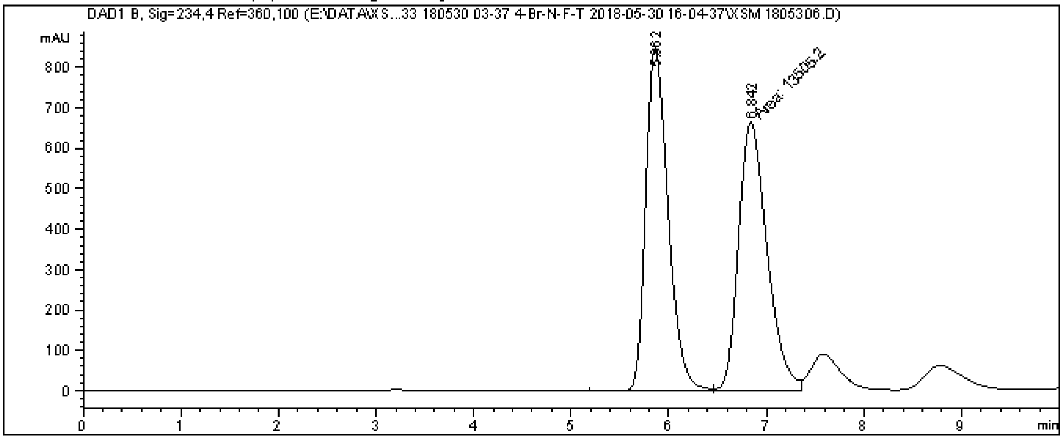


```
=====================================================================
                         Area Percent Report
=====================================================================

Sorted By             :      Signal
Multiplier            :      1.0000
Dilution              :      1.0000
Do not use Multiplier & Dilution Factor with ISTDs


Signal 1: DAD1 B, Sig=234,4 Ref=360,100
```

Peak #	RetTime [min]	Type	Width [min]	Area [mAU*s]	Height [mAU]	Area %
1	5.862	BV	0.2444	1.35542e4	848.46832	50.0906
2	6.842	MF	0.3389	1.35052e4	664.23364	49.9094
Totals :				2.70594e4	1512.70197	

```
=====================================================================
                         *** End of Report ***
```

1260 10/8/2018 3:09:45 PM SYSTEM   Page 1 of 1

Supplementary Figure 162. HPLC spectrum of (1*R**,3*S**,4*R**)-**3r**

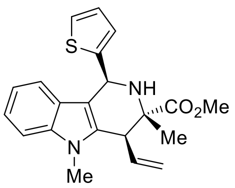

(1*R*,3*S*,4*R*)-**3r**

Data File E:\DATA\XSM\In33 180530 03-37 4-Br-N-F-T 2018-05-30 16-04-37\XSM 1805307.D
Sample Name: In33 03-36 Thienyl R+L opt

===

Acq. Operator : SYSTEM Seq. Line : 8
Acq. Instrument : 1260 Location : 20
Injection Date : 5/30/2018 6:32:11 PM Inj : 1
Inj Volume : 5.000 µl
Acq. Method : E:\DATA\XSM\In33 180530 03-37 4-Br-N-F-T 2018-05-30 16-04-37\In33 AS-98-2-234nm-20min-noD.M
Last changed : 5/30/2018 4:04:37 PM by SYSTEM
Analysis Method : E:\DATA\XSM\In33 180530 03-37 4-Br-N-F-T 2018-05-30 16-04-37\In33 AS-98-2-234nm-20min-noD.M (Sequence Method)
Last changed : 10/8/2018 3:09:43 PM by SYSTEM
(modified after loading)
Additional Info : Peak(s) manually integrated

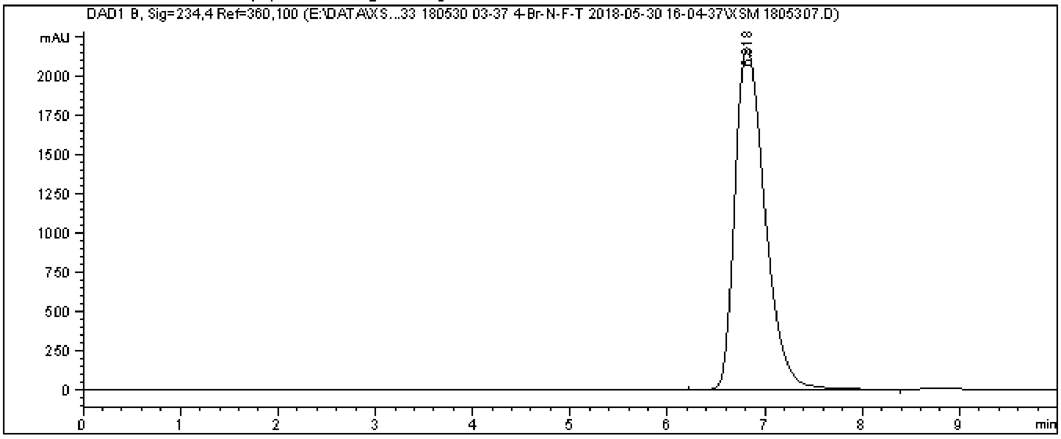

===

Area Percent Report

===

Sorted By : Signal
Multiplier : 1.0000
Dilution : 1.0000
Do not use Multiplier & Dilution Factor with ISTDs

Signal 1: DAD1 B, Sig=234,4 Ref=360,100

Peak #	RetTime [min]	Type	Width [min]	Area [mAU*s]	Height [mAU]	Area %
1	6.818	BB	0.3306	4.68628e4	2181.35010	100.0000

Totals : 4.68628e4 2181.35010

===

*** End of Report ***

1260 10/8/2018 3:10:14 PM SYSTEM Page 1 of 1

Supplementary Figure 163. HPLC spectrum of (1*R*,3*S*,4*R*)-**3r**

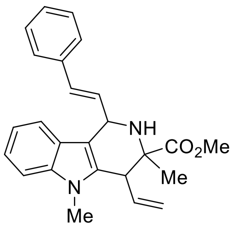

(1*R**,3*S**,4*R**)-**3s**

Data File E:\DATA\XS...3-53 Cinnamyl 1st 2018-07-02 14-49-15\XSM 20180702 03-53 Cinnamyl.D
Sample Name: 03-53 Cinn 1st rac

===

Acq. Operator : SYSTEM   Seq. Line : 1
Acq. Instrument : 1260   Location : 76
Injection Date : 7/2/2018 2:50:17 PM   Inj : 1
Inj Volume : 5.000 µl
Acq. Method : E:\DATA\XSM-In33\In33 180702 03-53 Cinnamyl 1st 2018-07-02 14-49-15\In33 Cinn AS-98-1mL-254nm-noD-10min.M
Last changed : 7/2/2018 2:49:15 PM by SYSTEM
Analysis Method : E:\DATA\XSM-In33\In33 180702 03-53 Cinnamyl 1st 2018-07-02 14-49-15\In33 Cinn AS-98-1mL-254nm-noD-10min.M (Sequence Method)
Last changed : 10/7/2018 8:25:08 PM by SYSTEM
(modified after loading)

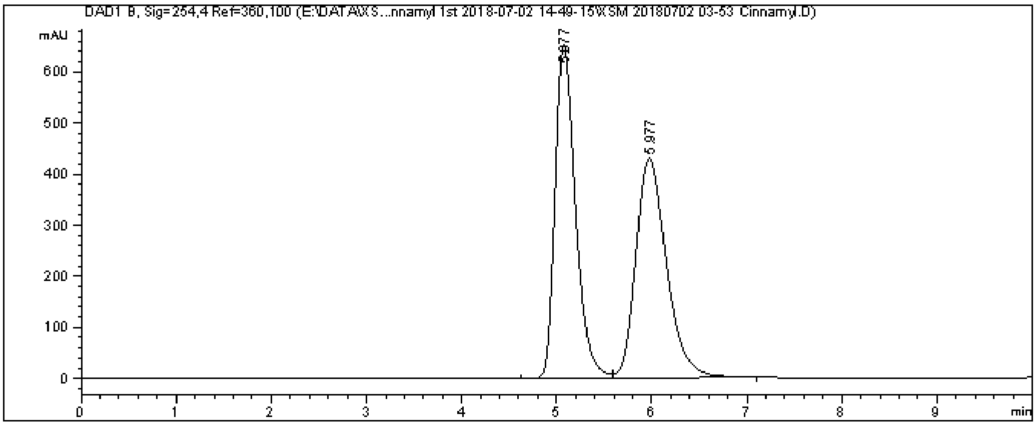

===

Area Percent Report

===

Sorted By : Signal
Multiplier : 1.0000
Dilution : 1.0000
Do not use Multiplier & Dilution Factor with ISTDs

Signal 1: DAD1 B, Sig=254,4 Ref=360,100

Peak #	RetTime [min]	Type	Width [min]	Area [mAU*s]	Height [mAU]	Area %
1	5.077	BV	0.2271	9676.55859	652.09113	50.2501
2	5.977	VB	0.3423	9580.22559	429.46201	49.7499

Totals : 1.92568e4 1081.55313

===

*** End of Report ***

1260 10/7/2018 8:25:11 PM SYSTEM   Page 1 of 1

Supplementary Figure 164. HPLC spectrum of (1*R**,3*S**,4*R**)-**3s**

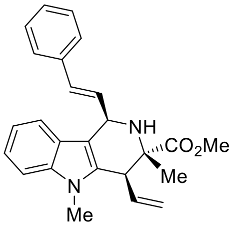

(1*R*,3*S*,4*R*)-**3s**

```
Data File E:\DATA\XS...-53 Cinnamyl 1st 2018-07-02 14-49-15\XSM 20180702 03-53 Cinnamyl1.D
Sample Name: 03-41 Cinn R+L opt

    =====================================================================
    Acq. Operator   : SYSTEM                          Seq. Line :    2
    Acq. Instrument : 1260                             Location :    88
    Injection Date  : 7/2/2018 3:01:14 PM                   Inj :    1
                                                     Inj Volume : 5.000 µl
    Acq. Method     : E:\DATA\XSM-In33\In33 180702 03-53 Cinnamyl 1st 2018-07-02 14-49-15\In33
                      Cinn AS-98-1mL-254nm-noD-10min.M
    Last changed    : 7/2/2018 2:49:15 PM by SYSTEM
    Analysis Method : E:\DATA\XSM-In33\In33 180702 03-53 Cinnamyl 1st 2018-07-02 14-49-15\In33
                      Cinn AS-98-1mL-254nm-noD-10min.M (Sequence Method)
    Last changed    : 10/7/2018 8:25:08 PM by SYSTEM
                      (modified after loading)
    Additional Info : Peak(s) manually integrated
```

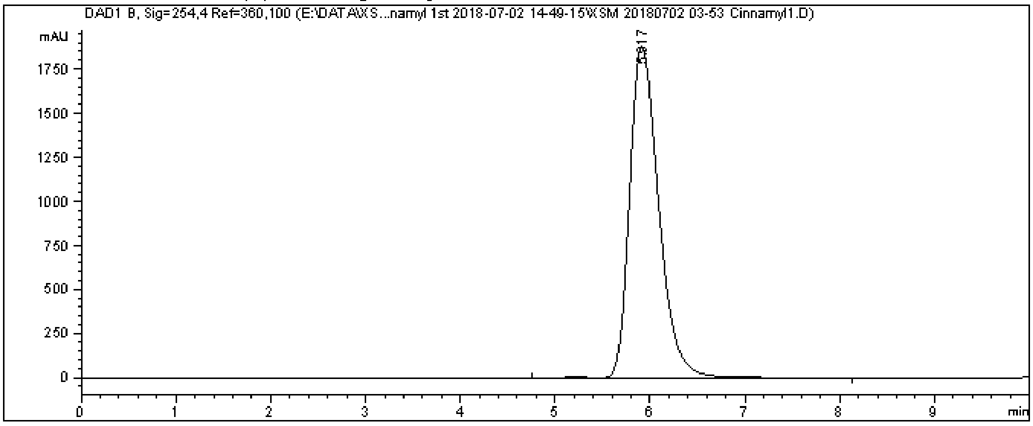


```
    =====================================================================
                             Area Percent Report
    =====================================================================

    Sorted By             :      Signal
    Multiplier            :      1.0000
    Dilution              :      1.0000
    Do not use Multiplier & Dilution Factor with ISTDs


    Signal 1: DAD1 B, Sig=254,4 Ref=360,100

    Peak RetTime Type  Width     Area      Height     Area
      #   [min]        [min]   [mAU*s]     [mAU]        %
    ----|-------|----|-------|----------|----------|--------|
       1   5.917 VB R  0.3290 4.03041e4  1883.49219 100.0000

    Totals :                  4.03041e4  1883.49219


    =====================================================================
                              *** End of Report ***


1260 10/7/2018 8:28:51 PM SYSTEM                                      Page 1 of 1
```

Supplementary Figure 165. HPLC spectrum of (1*R*,3*S*,4*R*)-**3s**

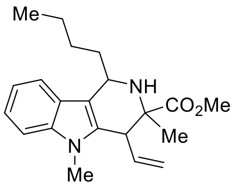

(1*R**,3*S**,4*R**)-**3t**

```
Data File E:\DATA\XS...80702 03-51 n-Bu 1st 2018-07-02 17-15-03\XSM 20180702 03-53 n-Bu1.D
Sample Name: 03-51 n-Bu 1st rac

    =====================================================================
    Acq. Operator   : SYSTEM                        Seq. Line :   2
    Acq. Instrument : 1260                           Location :   79
    Injection Date  : 7/2/2018 5:37:07 PM                 Inj :   1
                                                   Inj Volume : 15.000 µl
    Acq. Method     : E:\DATA\XSM-In33\In33 180702 03-51 n-Bu 1st 2018-07-02 17-15-03\In33 n-Bu
                      ID-98-0.5-20min-noD-15uL.M
    Last changed    : 7/2/2018 5:15:03 PM by SYSTEM
    Analysis Method : E:\DATA\XSM-In33\In33 180702 03-51 n-Bu 1st 2018-07-02 17-15-03\In33 n-Bu
                      ID-98-0.5-20min-noD-15uL.M (Sequence Method)
    Last changed    : 10/8/2018 3:15:26 PM by SYSTEM
                      (modified after loading)
    Additional Info : Peak(s) manually integrated
```

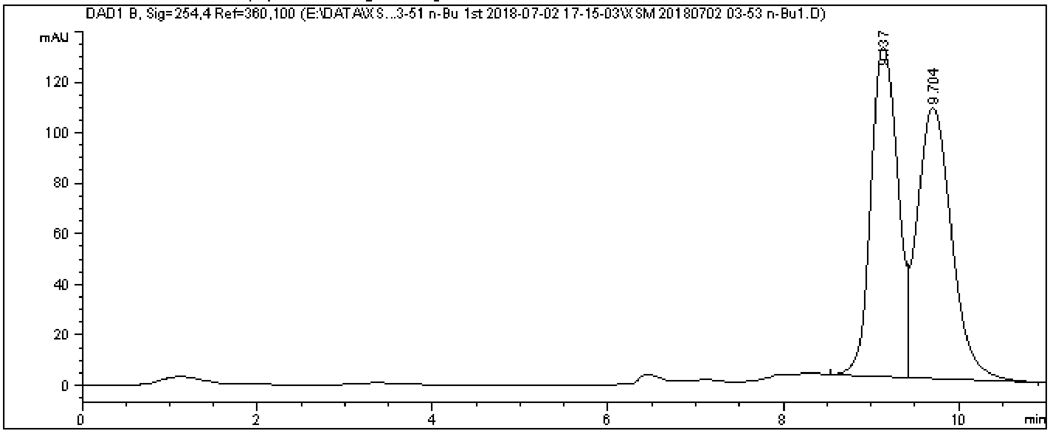


```
    =====================================================================
                             Area Percent Report
    =====================================================================

    Sorted By             :      Signal
    Multiplier            :      1.0000
    Dilution              :      1.0000
    Do not use Multiplier & Dilution Factor with ISTDs


    Signal 1: DAD1 B, Sig=254,4 Ref=360,100

    Peak RetTime Type  Width     Area      Height     Area
      #   [min]        [min]   [mAU*s]     [mAU]        %
    ----|-------|----|-------|----------|----------|--------|
       1   9.137 BV    0.3384 2797.18579  130.33398  48.9638
       2   9.704 VB    0.4217 2915.58276  107.08229  51.0362

    Totals :                  5712.76855  237.41628


    =====================================================================
                             *** End of Report ***
```


Supplementary Figure 166. HPLC spectrum of (1*R**,3*S**,4*R**)-**3t**

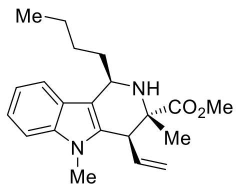

(1*R*,3*S*,4*R*)-**3t**

```
Data File E:\DATA\XS...180702 03-51 n-Bu 1st 2018-07-02 17-15-03\XSM 20180702 03-53 n-Bu.D
Sample Name: 03-51 n-Bu R+L opt

    =====================================================================
    Acq. Operator   : SYSTEM                        Seq. Line :   1
    Acq. Instrument : 1260                           Location :   90
    Injection Date  : 7/2/2018 5:16:06 PM                 Inj :   1
                                                  Inj Volume : 15.000 µl
    Acq. Method     : E:\DATA\XSM-In33\In33 180702 03-51 n-Bu 1st 2018-07-02 17-15-03\In33 n-Bu
                      ID-98-0.5-20min-noD-15uL.M
    Last changed    : 7/2/2018 5:15:03 PM by SYSTEM
    Analysis Method : E:\DATA\XSM-In33\In33 180702 03-51 n-Bu 1st 2018-07-02 17-15-03\In33 n-Bu
                      ID-98-0.5-20min-noD-15uL.M (Sequence Method)
    Last changed    : 10/8/2018 3:15:26 PM by SYSTEM
                      (modified after loading)
    Additional Info : Peak(s) manually integrated
```

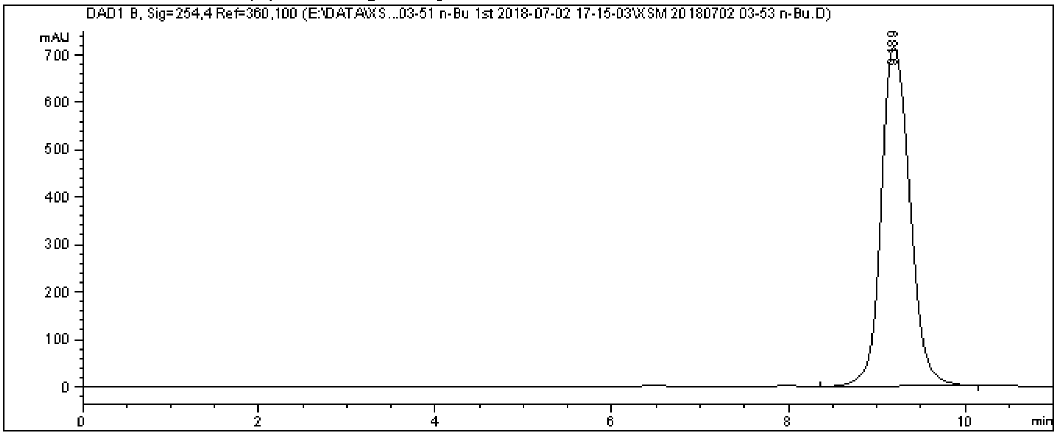


```
    =====================================================================
                            Area Percent Report
    =====================================================================

    Sorted By             :      Signal
    Multiplier            :      1.0000
    Dilution              :      1.0000
    Do not use Multiplier & Dilution Factor with ISTDs


    Signal 1: DAD1 B, Sig=254,4 Ref=360,100

    Peak RetTime Type  Width     Area      Height     Area
      #   [min]        [min]   [mAU*s]     [mAU]        %
    ----|-------|----|-------|----------|----------|--------|
       1   9.189 BB    0.3608 1.62232e4   714.69580 100.0000

    Totals :                  1.62232e4   714.69580


    =====================================================================
                             *** End of Report ***
```


Supplementary Figure 167. HPLC spectrum of (1*R*,3*S*,4*R*)-**3t**

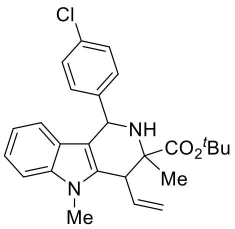

(1*R**,3*S**,4*R**)-**3u**

Data File E:\DATA\XSM\In33 180912 XSM 4-Cl-tBu 1st 2018-09-12 12-44-16\XSM 201809121.D
Sample Name: In33 03-95 4-Cl-tBu 1st rac

==

Acq. Operator : SYSTEM Seq. Line : 2
Acq. Instrument : 1260 Location : 67
Injection Date : 9/12/2018 1:07:22 PM Inj : 1
Inj Volume : 5.000 µl
Acq. Method : E:\DATA\XSM\In33 180912 XSM 4-Cl-tBu 1st 2018-09-12 12-44-16\In33 1st OD-98 -0.5mL-238nm 4-Cl-tBu.M
Last changed : 9/12/2018 12:44:16 PM by SYSTEM
Analysis Method : E:\DATA\XSM\In33 180912 XSM 4-Cl-tBu 1st 2018-09-12 12-44-16\In33 1st OD-98 -0.5mL-238nm 4-Cl-tBu.M (Sequence Method)
Last changed : 1/2/2019 5:20:27 PM by SYSTEM
(modified after loading)
Additional Info : Peak(s) manually integrated

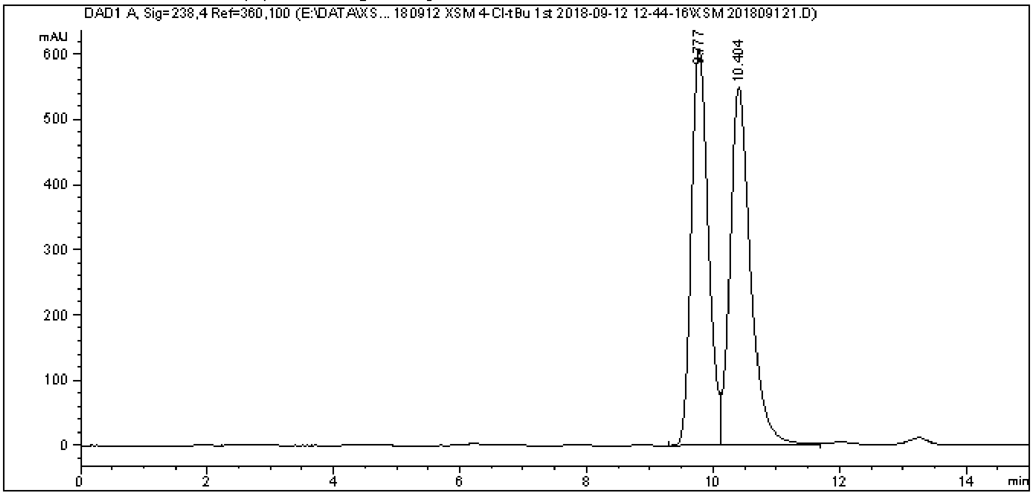

==

Area Percent Report

==

Sorted By : Signal
Multiplier : 1.0000
Dilution : 1.0000
Do not use Multiplier & Dilution Factor with ISTDs

Signal 1: DAD1 A, Sig=238,4 Ref=360,100

Peak #	RetTime [min]	Type	Width [min]	Area [mAU*s]	Height [mAU]	Area %
1	9.777	BV	0.2857	1.12280e4	607.32733	47.7466
2	10.404	VB	0.3373	1.22877e4	548.55389	52.2534

Totals : 2.35157e4 1155.88123

==

*** End of Report ***

1260 1/2/2019 5:20:30 PM SYSTEM Page 1 of 1

Supplementary Figure 168. HPLC spectrum of (1*R**,3*S**,4*R**)-**3u**

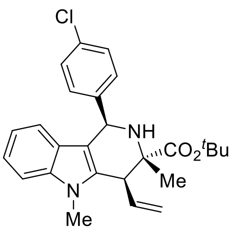

(1*R*,3*S*,4*R*)-**3u**

```
Data File E:\DATA\XSM\In33 180912 XSM 4-Cl-tBu 2018-09-12 16-16-18\XSM 20180908.D
Sample Name: In33 03-93 4-Cl-tBu R+L opt

=====================================================================
Acq. Operator   : SYSTEM                        Seq. Line :    1
Acq. Instrument : 1260                           Location :   68
Injection Date  : 9/12/2018 4:17:56 PM                Inj :    1
                                               Inj Volume : 5.000 µl
Acq. Method     : E:\DATA\XSM\In33 180912 XSM 4-Cl-tBu 2018-09-12 16-16-18\In33 1st OD-99-0.
                  5mL-238nm 4-Cl-tBu.M
Last changed    : 9/12/2018 4:16:19 PM by SYSTEM
Analysis Method : E:\DATA\XSM\In33 180912 XSM 4-Cl-tBu 2018-09-12 16-16-18\In33 1st OD-99-0.
                  5mL-238nm 4-Cl-tBu.M (Sequence Method)
Last changed    : 1/2/2019 5:21:06 PM by SYSTEM
                  (modified after loading)
Additional Info : Peak(s) manually integrated
```

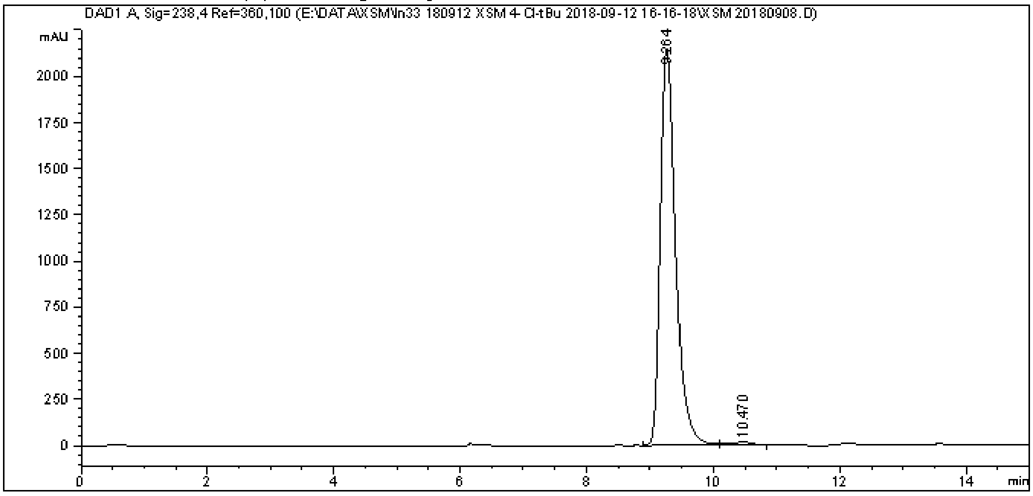


```
=====================================================================
                         Area Percent Report
=====================================================================

Sorted By             :      Signal
Multiplier            :      1.0000
Dilution              :      1.0000
Do not use Multiplier & Dilution Factor with ISTDs


Signal 1: DAD1 A, Sig=238,4 Ref=360,100

Peak RetTime Type  Width     Area      Height     Area
  #   [min]        [min]   [mAU*s]     [mAU]        %
----|-------|----|-------|----------|----------|--------|
   1   9.264 BB    0.2585 3.62836e4  2143.71753  99.3170
   2  10.470 BB    0.2539  249.53914   14.71065   0.6830

Totals :                  3.65331e4  2158.42818


=====================================================================
                          *** End of Report ***
```

1260 1/2/2019 5:21:08 PM SYSTEM   Page 1 of 1

Supplementary Figure 169. HPLC spectrum of (1*R*,3*S*,4*R*)-**3u**

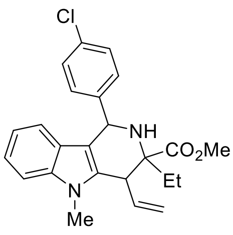

(1*R**,3*S**,4*R**)-**3v**

```
Data File E:\DATA\XSM-In33\In33 180831 03-79 a-Et1st 2018-08-31 15-06-43\XSM 20180831-21.D
Sample Name: In33 03-92 a-Et 1st rac

=====================================================================
Acq. Operator   : SYSTEM                         Seq. Line :    2
Acq. Instrument : 1260                            Location :   63
Injection Date  : 8/31/2018 3:23:34 PM                 Inj :    1
                                                Inj Volume : 5.000 µl
Acq. Method     : E:\DATA\XSM-In33\In33 180831 03-79 a-Et1st 2018-08-31 15-06-43\In33 1st AS-
                  98-1mL-236nm-noD-15min a-Et.M
Last changed    : 8/31/2018 3:06:43 PM by SYSTEM
Analysis Method : E:\DATA\XSM-In33\In33 180831 03-79 a-Et1st 2018-08-31 15-06-43\In33 1st AS-
                  98-1mL-236nm-noD-15min a-Et.M (Sequence Method)
Last changed    : 10/7/2018 9:07:15 PM by SYSTEM
                  (modified after loading)
Additional Info : Peak(s) manually integrated
```

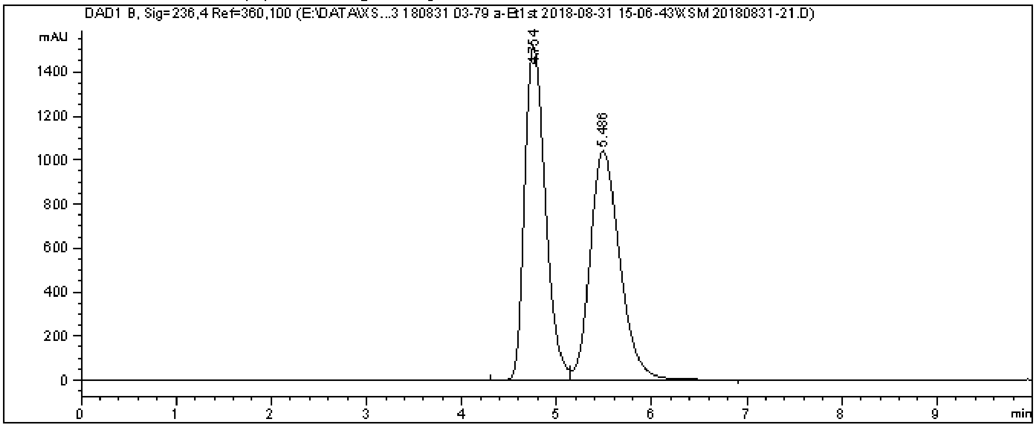


```
=====================================================================
                         Area Percent Report
=====================================================================

Sorted By             :      Signal
Multiplier            :      1.0000
Dilution              :      1.0000
Do not use Multiplier & Dilution Factor with ISTDs


Signal 1: DAD1 B, Sig=236,4 Ref=360,100

Peak RetTime Type  Width     Area      Height     Area
  #   [min]        [min]   [mAU*s]     [mAU]        %
----|-------|----|-------|----------|----------|--------|
   1   4.754 BV    0.2342 2.29155e4 1516.93030  50.4745
   2   5.486 VB    0.3341 2.24847e4 1040.44324  49.5255

Totals :                  4.54002e4 2557.37354


=====================================================================
                          *** End of Report ***
```

1260 10/7/2018 9:07:48 PM SYSTEM　　　Page 1 of 1

Supplementary Figure 170. HPLC spectrum of (1*R**,3*S**,4*R**)-**3v**

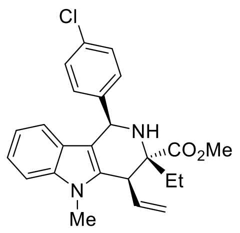

(1*R*,3*S*,4*R*)-**3v**

Data File E:\DATA\XSM-In33\In33 180831 03-79 a-Et1st 2018-08-31 15-06-43\XSM 20180831-2.D
Sample Name: In33 03-79 a-Et R+L opt

===

Acq. Operator : SYSTEM   Seq. Line : 1
Acq. Instrument : 1260   Location : 64
Injection Date : 8/31/2018 3:07:37 PM   Inj : 1
Inj Volume : 5.000 µl
Acq. Method : E:\DATA\XSM-In33\In33 180831 03-79 a-Et1st 2018-08-31 15-06-43\In33 1st AS-98-1mL-236nm-noD-15min a-Et.M
Last changed : 8/31/2018 3:06:43 PM by SYSTEM
Analysis Method : E:\DATA\XSM-In33\In33 180831 03-79 a-Et1st 2018-08-31 15-06-43\In33 1st AS-98-1mL-236nm-noD-15min a-Et.M (Sequence Method)
Last changed : 10/7/2018 9:07:15 PM by SYSTEM
(modified after loading)
Additional Info : Peak(s) manually integrated

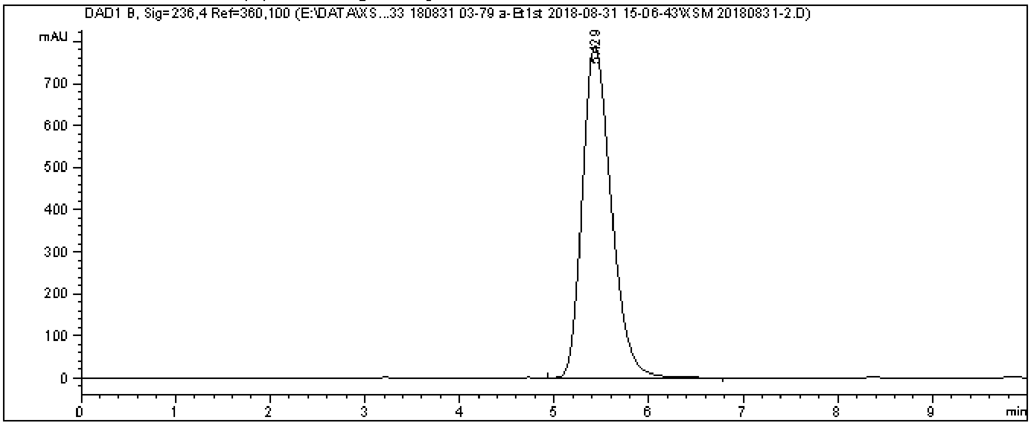

===

Area Percent Report

===

Sorted By : Signal
Multiplier : 1.0000
Dilution : 1.0000
Do not use Multiplier & Dilution Factor with ISTDs

Signal 1: DAD1 B, Sig=236,4 Ref=360,100

Peak #	RetTime [min]	Type	Width [min]	Area [mAU*s]	Height [mAU]	Area %
1	5.429	BB	0.3302	1.67557e4	787.60681	100.0000

Totals : 1.67557e4 787.60681

===

*** End of Report ***

1260 10/7/2018 9:07:20 PM SYSTEM   Page 1 of 1

Supplementary Figure 171. HPLC spectrum of (1*R*,3*S*,4*R*)-**3v**

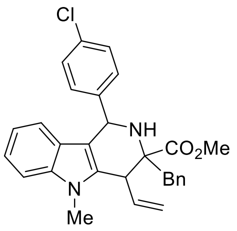

(1*R**,3*S**,4*R**)-**3w**

```
Data File E:\DATA\XSM\In33 181004 XSM 03-113 a-Bn 2018-10-04 15-22-56\XSM-1810041.D
Sample Name: In33 03-113 a-Bn 1st rac

    ==================================================================
    Acq. Operator   : SYSTEM                         Seq. Line :    2
    Acq. Instrument : 1260                            Location :   69
    Injection Date  : 10/4/2018 3:46:03 PM                 Inj :    1
                                                    Inj Volume : 5.000 µl
    Acq. Method     : E:\DATA\XSM\In33 181004 XSM 03-113 a-Bn 2018-10-04 15-22-56\In33 1st rac IE
                      -98-236nm-20min a-Bn.M
    Last changed    : 10/4/2018 3:22:57 PM by SYSTEM
    Analysis Method : E:\DATA\XSM\In33 181004 XSM 03-113 a-Bn 2018-10-04 15-22-56\In33 1st rac IE
                      -98-236nm-20min a-Bn.M (Sequence Method)
    Last changed    : 1/2/2019 5:09:23 PM by SYSTEM
                      (modified after loading)
    Additional Info : Peak(s) manually integrated
```

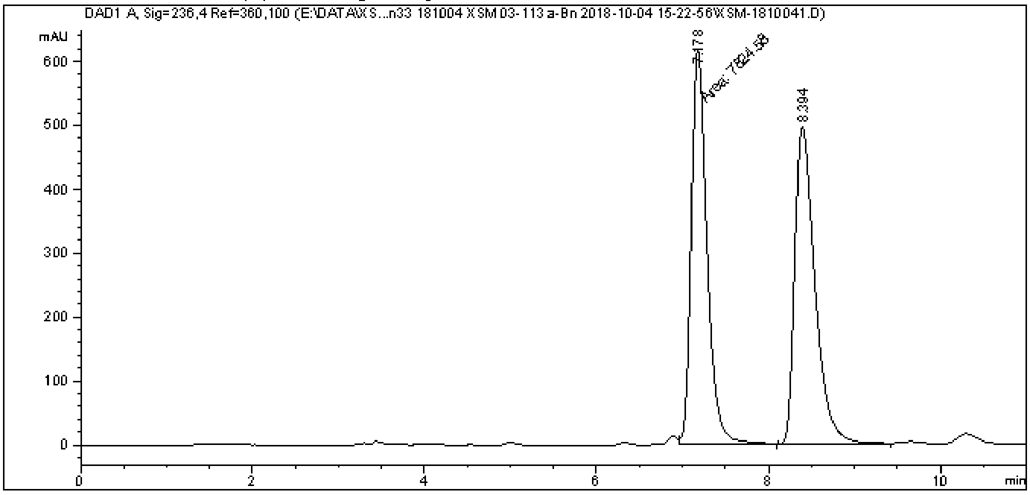


```
    ==================================================================
                             Area Percent Report
    ==================================================================

    Sorted By             :      Signal
    Multiplier            :      1.0000
    Dilution              :      1.0000
    Do not use Multiplier & Dilution Factor with ISTDs


    Signal 1: DAD1 A, Sig=236,4 Ref=360,100

    Peak RetTime Type  Width     Area      Height     Area
      #   [min]        [min]   [mAU*s]     [mAU]        %
    ----|-------|----|-------|----------|----------|--------|
       1   7.178 FM    0.2108 7824.58398  618.49780  49.2750
       2   8.394 BB    0.2465 8054.82324  495.89856  50.7250

    Totals :                  1.58794e4  1114.39636


    ==================================================================
                             *** End of Report ***
```

1260 1/2/2019 5:10:37 PM SYSTEM   Page 1 of 1

Supplementary Figure 172. HPLC spectrum of (1*R**,3*S**,4*R**)-**3w**

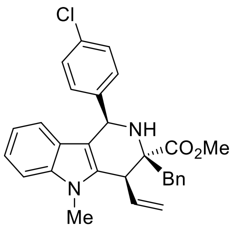

(1*R*,3*S*,4*R*)-**3w**

Data File E:\DATA\XSM\In33 181004 XSM 03-113 a-Bn 2018-10-04 15-22-56\XSM-181004.D
Sample Name: In33 03-113 a-Bn R+L opt

==

Acq. Operator : SYSTEM Seq. Line : 1
Acq. Instrument : 1260 Location : 70
Injection Date : 10/4/2018 3:24:32 PM Inj : 1
Inj Volume : 5.000 µl
Acq. Method : E:\DATA\XSM\In33 181004 XSM 03-113 a-Bn 2018-10-04 15-22-56\In33 1st rac IE -98-236nm-20min a-Bn.M
Last changed : 10/4/2018 3:22:57 PM by SYSTEM
Analysis Method : E:\DATA\XSM\In33 181004 XSM 03-113 a-Bn 2018-10-04 15-22-56\In33 1st rac IE -98-236nm-20min a-Bn.M (Sequence Method)
Last changed : 1/2/2019 5:09:23 PM by SYSTEM
(modified after loading)
Additional Info : Peak(s) manually integrated

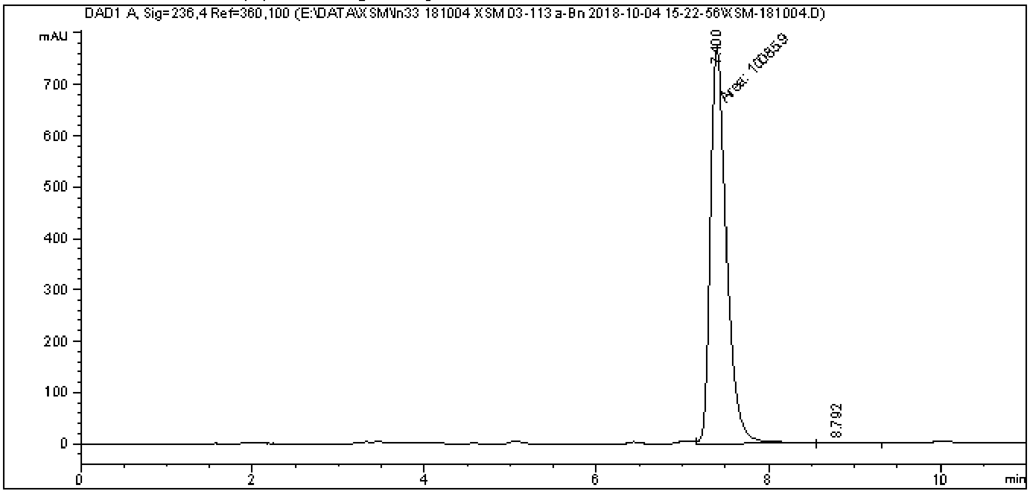

==

Area Percent Report

==

Sorted By : Signal
Multiplier : 1.0000
Dilution : 1.0000
Do not use Multiplier & Dilution Factor with ISTDs

Signal 1: DAD1 A, Sig=236,4 Ref=360,100

Peak #	RetTime [min]	Type	Width [min]	Area [mAU*s]	Height [mAU]	Area %
1	7.400	FM	0.2188	1.00859e4	768.44086	99.7616
2	8.792	BB	0.1963	24.09840	1.48532	0.2384

Totals : 1.01100e4 769.92618

==

*** End of Report ***

1260 1/2/2019 5:09:27 PM SYSTEM Page 1 of 1

Supplementary Figure 173. HPLC spectrum of (1*R*,3*S*,4*R*)-**3w**

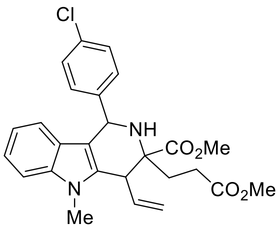

(1*R**,3*S**,4*R**)-**3x**

Data File E:\DATA\XSM\In33 181010 03-127 a-Glu IE 95 2018-10-10 14-17-35\XSM 201810101.D
Sample Name: In33 03-127 a-Glu 1st rac

===

Acq. Operator : SYSTEM Seq. Line : 2
Acq. Instrument : 1260 Location : 69
Injection Date : 10/10/2018 2:50:46 PM Inj : 1
Inj Volume : 5.000 µl
Acq. Method : E:\DATA\XSM\In33 181010 03-127 a-Glu IE 95 2018-10-10 14-17-35\In33 1st rac IE-95-40min-DAD a-Glu.M
Last changed : 10/10/2018 2:17:35 PM by SYSTEM
Analysis Method : E:\DATA\XSM\In33 181010 03-127 a-Glu IE 95 2018-10-10 14-17-35\In33 1st rac IE-95-40min-DAD a-Glu.M (Sequence Method)
Last changed : 1/2/2019 4:58:02 PM by SYSTEM
(modified after loading)
Additional Info : Peak(s) manually integrated

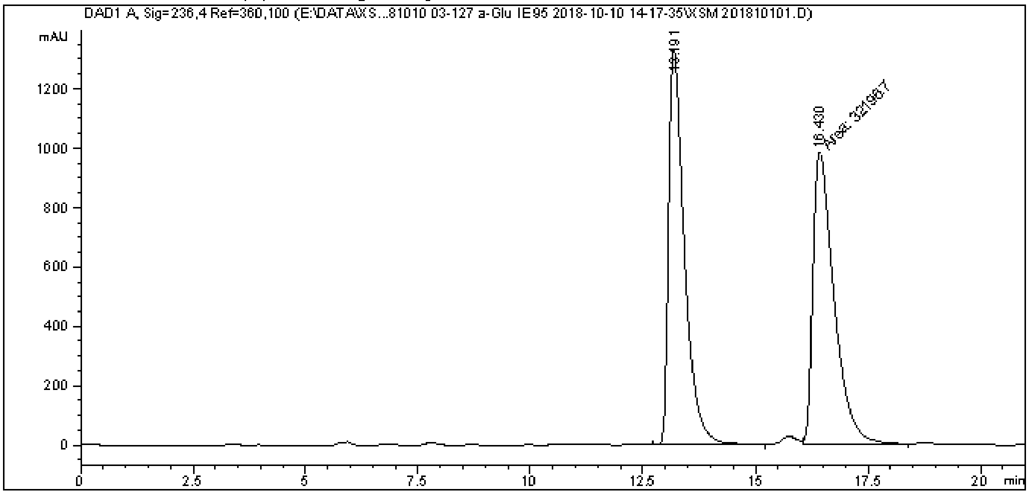

===

Area Percent Report

===

Sorted By : Signal
Multiplier : 1.0000
Dilution : 1.0000
Do not use Multiplier & Dilution Factor with ISTDs

Signal 1: DAD1 A, Sig=236,4 Ref=360,100

Peak #	RetTime [min]	Type	Width [min]	Area [mAU*s]	Height [mAU]	Area %
1	13.191	BB	0.3648	3.28503e4	1331.48438	50.5024
2	16.430	FM	0.5459	3.21967e4	983.03931	49.4976

Totals : 6.50470e4 2314.52368

===

*** End of Report ***

1260 1/2/2019 4:58:04 PM SYSTEM Page 1 of 1

Supplementary Figure 174. HPLC spectrum of (1*R**,3*S**,4*R**)-**3x**

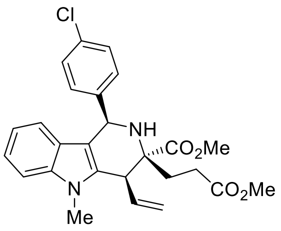

(1*R*,3*S*,4*R*)-**3x**

```
Data File E:\DATA\XSM\In33 181010 03-127 a-Glu IE 95 2018-10-10 14-17-35\XSM 20181010.D
Sample Name: In33 03-127 a-Glu R+L opt

    =====================================================================
    Acq. Operator   : SYSTEM                          Seq. Line :    1
    Acq. Instrument : 1260                             Location :   70
    Injection Date  : 10/10/2018 2:19:13 PM                 Inj :    1
                                                     Inj Volume : 5.000 µl
    Acq. Method     : E:\DATA\XSM\In33 181010 03-127 a-Glu IE 95 2018-10-10 14-17-35\In33 1st rac
                      IE-95-40min-DAD a-Glu.M
    Last changed    : 10/10/2018 2:17:35 PM by SYSTEM
    Analysis Method : E:\DATA\XSM\In33 181010 03-127 a-Glu IE 95 2018-10-10 14-17-35\In33 1st rac
                      IE-95-40min-DAD a-Glu.M (Sequence Method)
    Last changed    : 1/2/2019 4:58:02 PM by SYSTEM
                      (modified after loading)
    Additional Info : Peak(s) manually integrated
```

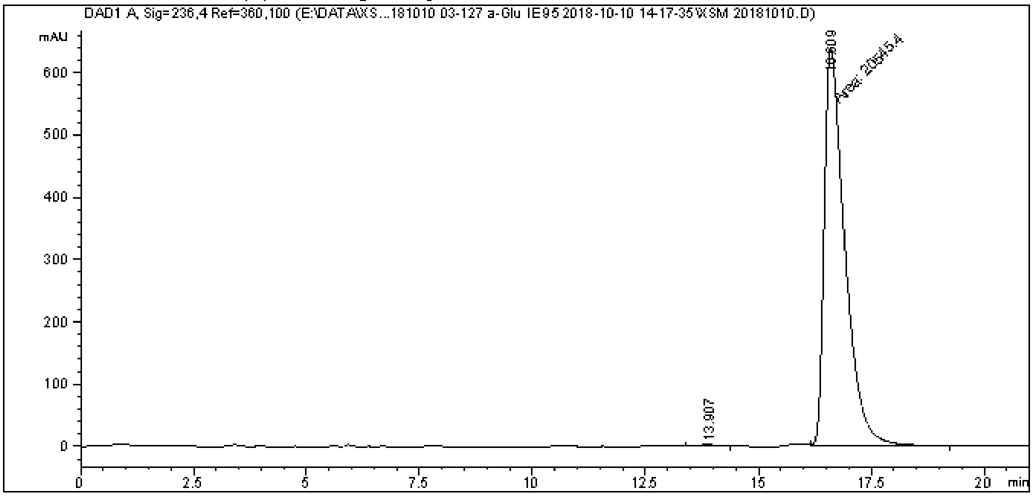


```
    =====================================================================
                             Area Percent Report
    =====================================================================

    Sorted By             :      Signal
    Multiplier            :      1.0000
    Dilution              :      1.0000
    Do not use Multiplier & Dilution Factor with ISTDs


    Signal 1: DAD1 A, Sig=236,4 Ref=360,100

    Peak RetTime Type  Width     Area      Height     Area
      #   [min]        [min]   [mAU*s]     [mAU]        %
    ----|-------|----|-------|----------|----------|--------|
       1  13.907 BB    0.3574   66.44785    2.18721   0.3224
       2  16.609 FM    0.5373 2.05454e4  637.28357  99.6776

    Totals :                  2.06118e4  639.47078


    =====================================================================
                             *** End of Report ***
```

1260 1/2/2019 4:58:43 PM SYSTEM Page 1 of 1

Supplementary Figure 175. HPLC spectrum of (1*R*,3*S*,4*R*)-**3x**

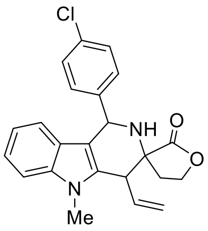

(1*R**,3*S**,4*R**)-**3y**

Data File E:\DATA\XS...3 180702 03-53 a-L 1st 2018-07-02 12-58-47\XSM 20180702 03-53 a-L.D
Sample Name: 03-53 a-L 1st rac

===

Acq. Operator : SYSTEM Seq. Line : 1
Acq. Instrument : 1260 Location : 78
Injection Date : 7/2/2018 12:59:52 PM Inj : 1
Inj Volume : 15.000 µl
Acq. Method : E:\DATA\XSM-In33\In33 180702 03-53 a-L 1st 2018-07-02 12-58-47\In33 a-L AS-92-1mL-254nm-noD-15uL.M
Last changed : 7/2/2018 1:39:28 PM by SYSTEM
(modified after loading)
Analysis Method : E:\DATA\XSM-In33\In33 180702 03-53 a-L 1st 2018-07-02 12-58-47\In33 a-L AS-92-1mL-254nm-noD-15uL.M (Sequence Method)
Last changed : 10/7/2018 8:18:07 PM by SYSTEM
(modified after loading)
Additional Info : Peak(s) manually integrated

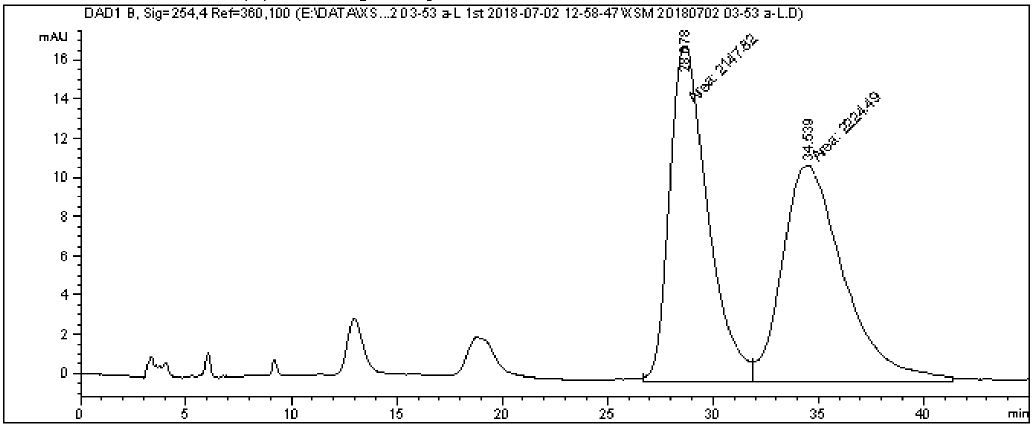

===

Area Percent Report

===

Sorted By : Signal
Multiplier : 1.0000
Dilution : 1.0000
Do not use Multiplier & Dilution Factor with ISTDs

Signal 1: DAD1 B, Sig=254,4 Ref=360,100

Peak #	RetTime [min]	Type	Width [min]	Area [mAU*s]	Height [mAU]	Area %
1	28.678	MF	2.0939	2147.82495	17.09606	49.1233
2	34.539	FM	3.3571	2224.49341	11.04360	50.8767
Totals :				4372.31836	28.13966	

===

*** End of Report ***

1260 10/7/2018 8:18:12 PM SYSTEM Page 1 of 1

Supplementary Figure 176. HPLC spectrum of (1*R**,3*S**,4*R**)-**3y**

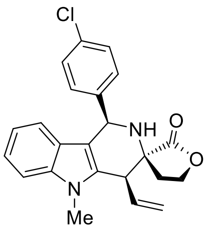

(1*R*,3*S*,4*R*)-**3y**

```
Data File E:\DATA\XS... 180702 03-53 a-L 1st 2018-07-02 12-58-47\XSM 20180702 03-53 a-L1.D
Sample Name: 03-53 a-L R+L opt

=====================================================================
Acq. Operator   : SYSTEM                          Seq. Line :    2
Acq. Instrument : 1260                             Location :   89
Injection Date  : 7/2/2018 1:50:55 PM                   Inj :    1
                                                 Inj Volume : 15.000 µl
Acq. Method     : E:\DATA\XSM-In33\In33 180702 03-53 a-L 1st 2018-07-02 12-58-47\In33 a-L AS-
                  92-1mL-254nm-noD-15uL.M
Last changed    : 7/2/2018 1:39:28 PM by SYSTEM
Analysis Method : E:\DATA\XSM-In33\In33 180702 03-53 a-L 1st 2018-07-02 12-58-47\In33 a-L AS-
                  92-1mL-254nm-noD-15uL.M (Sequence Method)
Last changed    : 10/7/2018 8:18:46 PM by SYSTEM
                  (modified after loading)
Additional Info : Peak(s) manually integrated
```

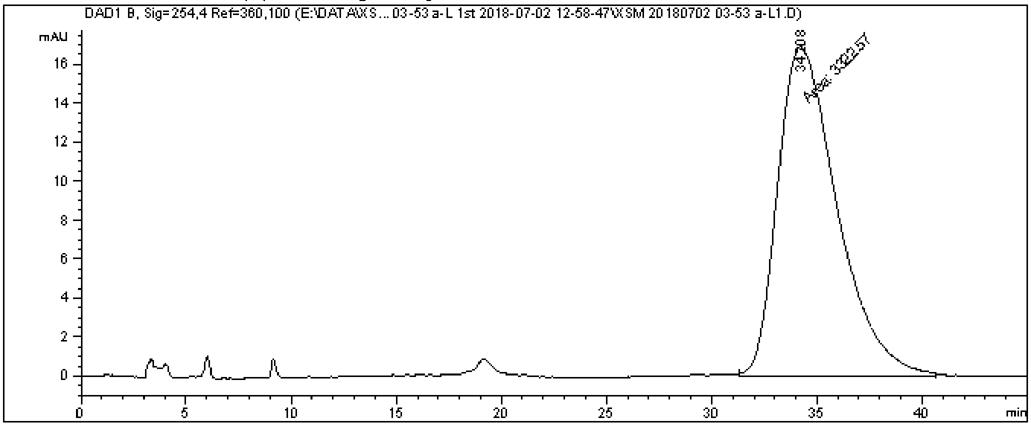


```
=====================================================================
                        Area Percent Report
=====================================================================

Sorted By             :      Signal
Multiplier            :      1.0000
Dilution              :      1.0000
Do not use Multiplier & Dilution Factor with ISTDs


Signal 1: DAD1 B, Sig=254,4 Ref=360,100

Peak RetTime Type  Width     Area      Height     Area
  #   [min]        [min]   [mAU*s]     [mAU]       %
----|-------|----|-------|----------|----------|--------|
   1  34.208 MM    3.2704 3322.56860   16.93274 100.0000

Totals :                  3322.56860   16.93274


=====================================================================
                          *** End of Report ***
```

1260 10/7/2018 8:18:48 PM SYSTEM Page 1 of 1

Supplementary Figure 177. HPLC spectrum of (1*R*,3*S*,4*R*)-**3y**

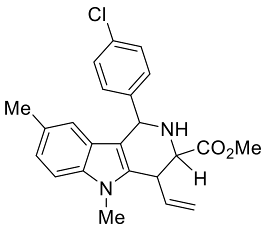

(1*R**,3*S**,4*R**)-**3z**

```
Data File E:\DATA\XS...230 04-12 2Cl-23Me-5Mein-aH 1st 2018-12-30 17-30-14\XSM 201812301.D
Sample Name: In33 04-12 5-Me-in-aH 1st rac

=====================================================================
Acq. Operator   : SYSTEM                          Seq. Line :   2
Acq. Instrument : 1260                             Location :   60
Injection Date  : 12/30/2018 6:02:10 PM                 Inj :   1
                                                Inj Volume : 5.000 µl
Acq. Method     : E:\DATA\XSM\In33 181230 04-12 2Cl-23Me-5Mein-aH 1st 2018-12-30 17-30-14
                  \In33 AD-98-226nm-30min 5-Me-in-aH.M
Last changed    : 12/30/2018 5:30:15 PM by SYSTEM
Analysis Method : E:\DATA\XSM\In33 181230 04-12 2Cl-23Me-5Mein-aH 1st 2018-12-30 17-30-14
                  \In33 AD-98-226nm-30min 5-Me-in-aH.M (Sequence Method)
Last changed    : 1/2/2019 11:03:14 AM by SYSTEM
                  (modified after loading)
Additional Info : Peak(s) manually integrated
```

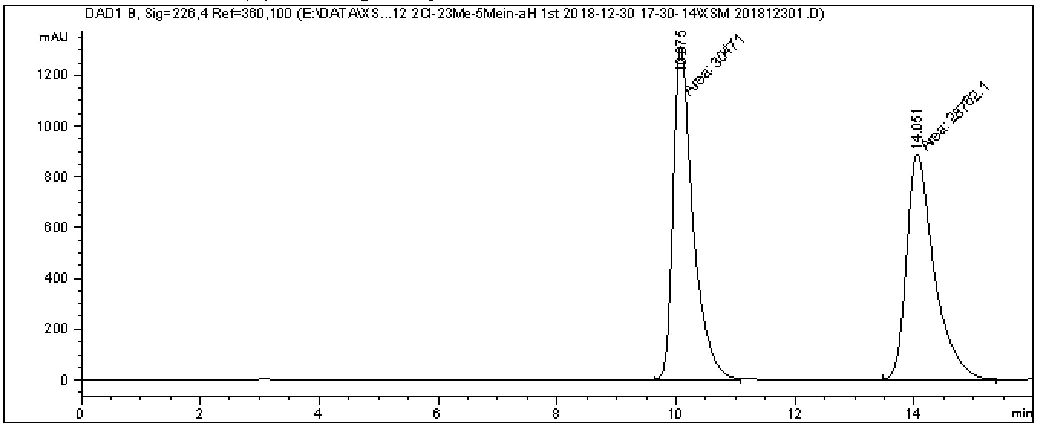


```
=====================================================================
                          Area Percent Report
=====================================================================

Sorted By             :      Signal
Multiplier            :      1.0000
Dilution              :      1.0000
Do not use Multiplier & Dilution Factor with ISTDs


Signal 1: DAD1 B, Sig=226,4 Ref=360,100

Peak RetTime Type  Width     Area      Height     Area
  #   [min]        [min]   [mAU*s]     [mAU]        %
----|-------|----|-------|----------|----------|--------|
   1  10.075 MF    0.3868 3.04710e4 1312.89771  51.4425
   2  14.051 MF    0.5388 2.87621e4  889.71027  48.5575

Totals :                  5.92331e4 2202.60797


=====================================================================
                          *** End of Report ***
```

1260 1/2/2019 11:03:17 AM SYSTEM   Page 1 of 1

Supplementary Figure 178. HPLC spectrum of (1*R**,3*S**,4*R**)-**3z**

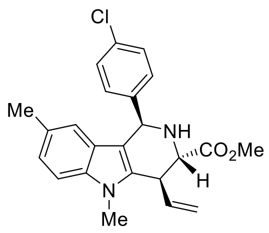

(1*R*,3*S*,4*R*)-**3z**

Data File E:\DATA\XS...230 04-12 2Cl-23Me-5Mein-aH 1st 2018-12-30 17-30-14\XSM 201812308.D
Sample Name: In33 04-12 5-Me-in-aH R+L opt

===

Acq. Operator : SYSTEM Seq. Line : 9
Acq. Instrument : 1260 Location : 59
Injection Date : 12/30/2018 9:38:51 PM Inj : 1
Inj Volume : 5.000 µl
Acq. Method : E:\DATA\XSM\In33 181230 04-12 2Cl-23Me-5Mein-aH 1st 2018-12-30 17-30-14 \In33 AD-98-226nm-30min 5-Me-in-aH.M
Last changed : 12/30/2018 5:30:15 PM by SYSTEM
Analysis Method : E:\DATA\XSM\In33 181230 04-12 2Cl-23Me-5Mein-aH 1st 2018-12-30 17-30-14 \In33 AD-98-226nm-30min 5-Me-in-aH.M (Sequence Method)
Last changed : 1/2/2019 11:03:14 AM by SYSTEM
(modified after loading)
Additional Info : Peak(s) manually integrated

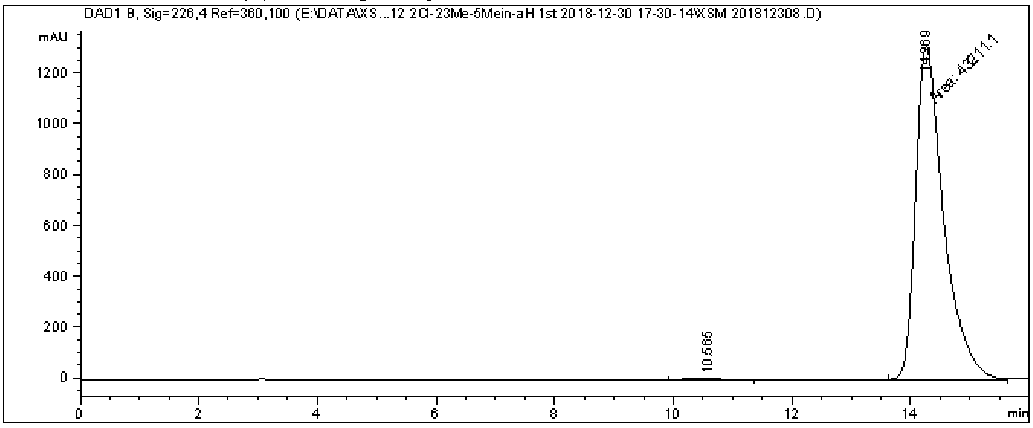

===

Area Percent Report

===

Sorted By : Signal
Multiplier : 1.0000
Dilution : 1.0000
Do not use Multiplier & Dilution Factor with ISTDs

Signal 1: DAD1 B, Sig=226,4 Ref=360,100

Peak #	RetTime [min]	Type	Width [min]	Area [mAU*s]	Height [mAU]	Area %
1	10.565	BB	0.4555	190.93904	5.66090	0.4399
2	14.269	MF	0.5491	4.32111e4	1311.52319	99.5601

Totals : 4.34021e4 1317.18410

===

*** End of Report ***

1260 1/2/2019 11:06:07 AM SYSTEM Page 1 of 1

Supplementary Figure 179. HPLC spectrum of (1*R*,3*S*,4*R*)-**3z**

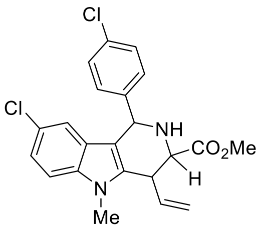

(1*R**,3*S**,4*R**)-**3A**

Data File E:\DATA\XSM\In33 181208 04-10 5-Cl-in-aH 4p div 2018-12-08 17-42-51\XSM 181207.D
Sample Name: In33 04-10 5-Cl-in-aH rac 4p div

===

Acq. Operator : SYSTEM Seq. Line : 1
Acq. Instrument : 1260 Location : 66
Injection Date : 12/8/2018 5:44:20 PM Inj : 1
Inj Volume : 5.000 µl
Acq. Method : E:\DATA\XSM\In33 181208 04-10 5-Cl-in-aH 4p div 2018-12-08 17-42-51\In33 AD -98-40min-238nm 5Cl-in-aH 4p div.M
Last changed : 12/8/2018 5:42:51 PM by SYSTEM
Analysis Method : E:\DATA\XSM\In33 181208 04-10 5-Cl-in-aH 4p div 2018-12-08 17-42-51\In33 AD -98-40min-238nm 5Cl-in-aH 4p div.M (Sequence Method)
Last changed : 1/2/2019 11:55:33 AM by SYSTEM
(modified after loading)
Additional Info : Peak(s) manually integrated

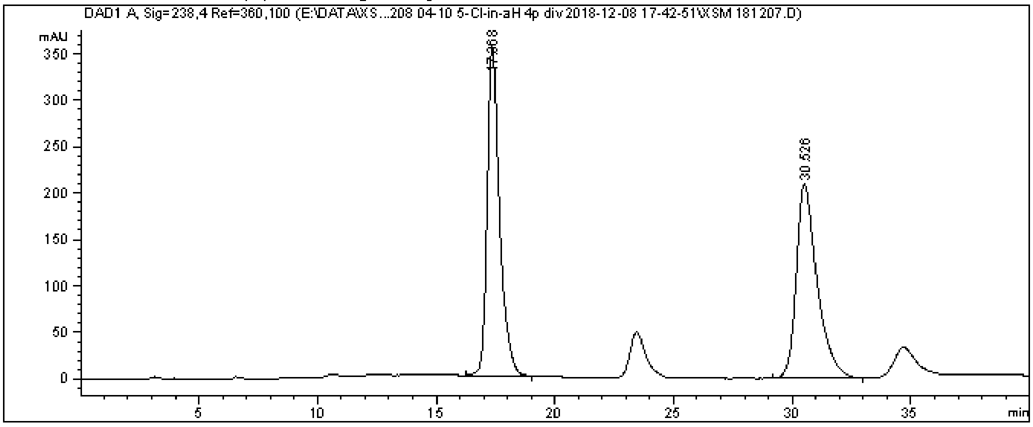

===

Area Percent Report

===

Sorted By : Signal
Multiplier : 1.0000
Dilution : 1.0000
Do not use Multiplier & Dilution Factor with ISTDs

Signal 1: DAD1 A, Sig=238,4 Ref=360,100

Peak #	RetTime [min]	Type	Width [min]	Area [mAU*s]	Height [mAU]	Area %
1	17.368	BB	0.5281	1.27770e4	354.80240	49.9110
2	30.526	BB	0.8524	1.28226e4	209.10309	50.0890

Totals : 2.55996e4 563.90549

===

*** End of Report ***

1260 1/2/2019 11:56:13 AM SYSTEM Page 1 of 1

Supplementary Figure 180. HPLC spectrum of (1*R**,3*S**,4*R**)-**3A**

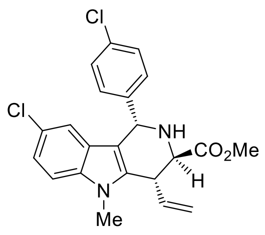

(1*S*,3*R*,4*S*)-**3A**

```
Data File E:\DATA\XS...33 181208 04-10 5-Cl-in-aH 4p div 2018-12-08 17-42-51\XSM 1812071.D
Sample Name: In33 04-10 5-Cl-in-aH S+D opt

=====================================================================
Acq. Operator   : SYSTEM                         Seq. Line :    2
Acq. Instrument : 1260                            Location :   67
Injection Date  : 12/8/2018 6:25:48 PM                 Inj :    1
                                                Inj Volume : 5.000 µl
Acq. Method     : E:\DATA\XSM\In33 181208 04-10 5-Cl-in-aH 4p div 2018-12-08 17-42-51\In33 AD
                  -98-40min-238nm 5Cl-in-aH 4p div.M
Last changed    : 12/8/2018 5:42:51 PM by SYSTEM
Analysis Method : E:\DATA\XSM\In33 181208 04-10 5-Cl-in-aH 4p div 2018-12-08 17-42-51\In33 AD
                  -98-40min-238nm 5Cl-in-aH 4p div.M (Sequence Method)
Last changed    : 1/2/2019 11:55:33 AM by SYSTEM
                  (modified after loading)
Additional Info : Peak(s) manually integrated
```

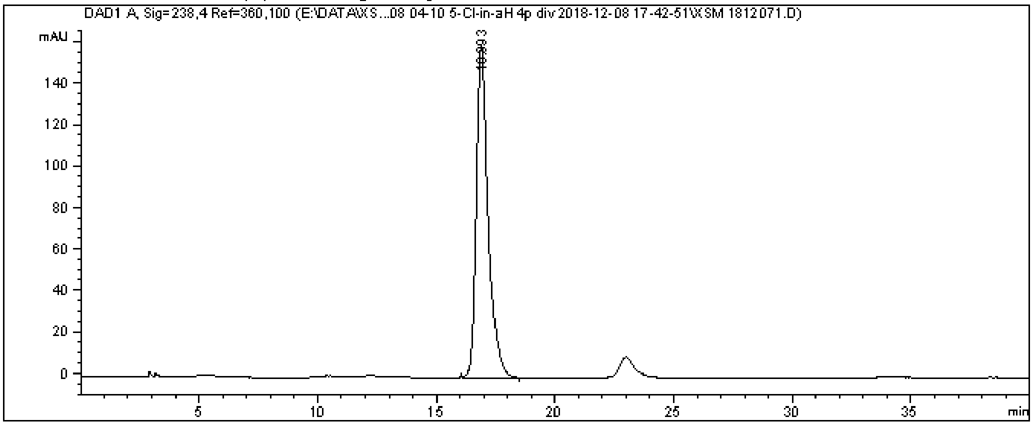


```
=====================================================================
                         Area Percent Report
=====================================================================

Sorted By             :      Signal
Multiplier            :      1.0000
Dilution              :      1.0000
Do not use Multiplier & Dilution Factor with ISTDs


Signal 1: DAD1 A, Sig=238,4 Ref=360,100

Peak RetTime Type  Width     Area      Height     Area
  #   [min]        [min]   [mAU*s]     [mAU]        %
----|-------|----|-------|----------|----------|--------|
   1  16.893 BB    0.5104 5577.32080  159.73375 100.0000

Totals :                  5577.32080  159.73375


=====================================================================
                          *** End of Report ***
```


Supplementary Figure 181. HPLC spectrum of (1*R*,3*S*,4*R*)-**3A**

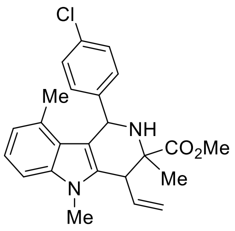

(1*R**,3*S**,4*R**)-**3B**

Data File E:\DATA\XS...17 03-68&77 4-Me-in 1st 2018-08-17 13-36-11\XSM 20180817 4-Me-in1.D
Sample Name: In33 03-77 4-Me-in 1st rac

```
=====================================================================
Acq. Operator   : SYSTEM                        Seq. Line :    2
Acq. Instrument : 1260                           Location :   62
Injection Date  : 8/17/2018 1:59:05 PM                Inj :    1
                                               Inj Volume : 1.000 µl
Acq. Method     : E:\DATA\XSM\In33 180817 03-68&77 4-Me-in 1st 2018-08-17 13-36-11\In33 As-98
                  -1mL-238nm-20min-noD 567-Me-in.M
Last changed    : 8/17/2018 1:36:12 PM by SYSTEM
Analysis Method : E:\DATA\XSM\In33 180817 03-68&77 4-Me-in 1st 2018-08-17 13-36-11\In33 As-98
                  -1mL-238nm-20min-noD 567-Me-in.M (Sequence Method)
Last changed    : 10/8/2018 3:28:36 PM by SYSTEM
                  (modified after loading)
Additional Info : Peak(s) manually integrated
```

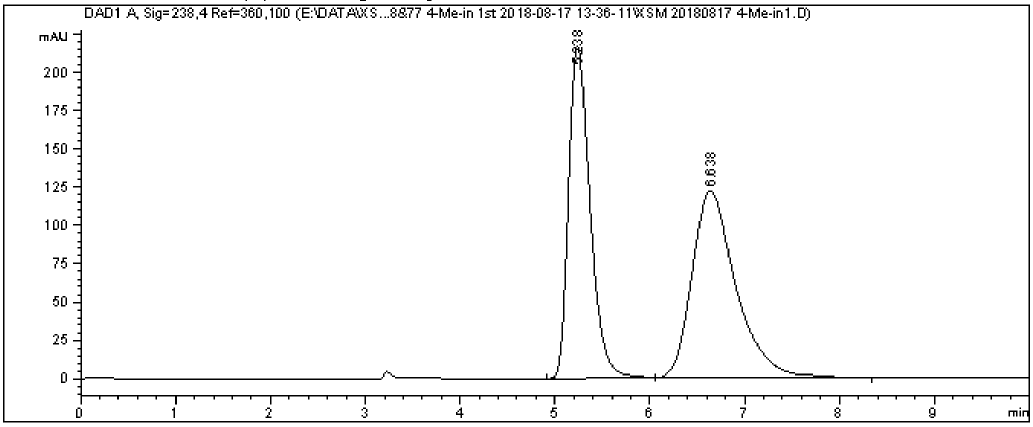


```
=====================================================================
                         Area Percent Report
=====================================================================

Sorted By             :      Signal
Multiplier            :      1.0000
Dilution              :      1.0000
Do not use Multiplier & Dilution Factor with ISTDs


Signal 1: DAD1 A, Sig=238,4 Ref=360,100

Peak RetTime Type  Width     Area      Height     Area
  #   [min]        [min]   [mAU*s]     [mAU]        %
----|-------|----|-------|----------|----------|--------|
   1   5.238 BB    0.2391 3402.60376  216.83220  46.5855
   2   6.638 BB    0.4757 3901.40088  121.79879  53.4145

Totals :                  7304.00464  338.63099


=====================================================================
                          *** End of Report ***
```

1260 10/8/2018 3:28:39 PM SYSTEM   Page 1 of 1

Supplementary Figure 182. HPLC spectrum of (1*R**,3*S**,4*R**)-**3B**

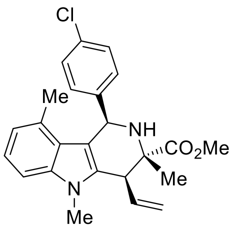

(1*R*,3*S*,4*R*)-**3B**

```
Data File E:\DATA\XS...817 03-68&77 4-Me-in 1st 2018-08-17 13-36-11\XSM 20180817 4-Me-in.D
Sample Name: In33 03-68 4-Me-in R+L opt

    =====================================================================
    Acq. Operator   : SYSTEM                        Seq. Line :    1
    Acq. Instrument : 1260                           Location :   63
    Injection Date  : 8/17/2018 1:37:35 PM                Inj :    1
                                                   Inj Volume : 1.000 µl
    Acq. Method     : E:\DATA\XSM\In33 180817 03-68&77 4-Me-in 1st 2018-08-17 13-36-11\In33 As-98
                      -1mL-238nm-20min-noD 567-Me-in.M
    Last changed    : 8/17/2018 1:36:12 PM by SYSTEM
    Analysis Method : E:\DATA\XSM\In33 180817 03-68&77 4-Me-in 1st 2018-08-17 13-36-11\In33 As-98
                      -1mL-238nm-20min-noD 567-Me-in.M (Sequence Method)
    Last changed    : 10/8/2018 3:28:36 PM by SYSTEM
                      (modified after loading)
    Additional Info : Peak(s) manually integrated
```

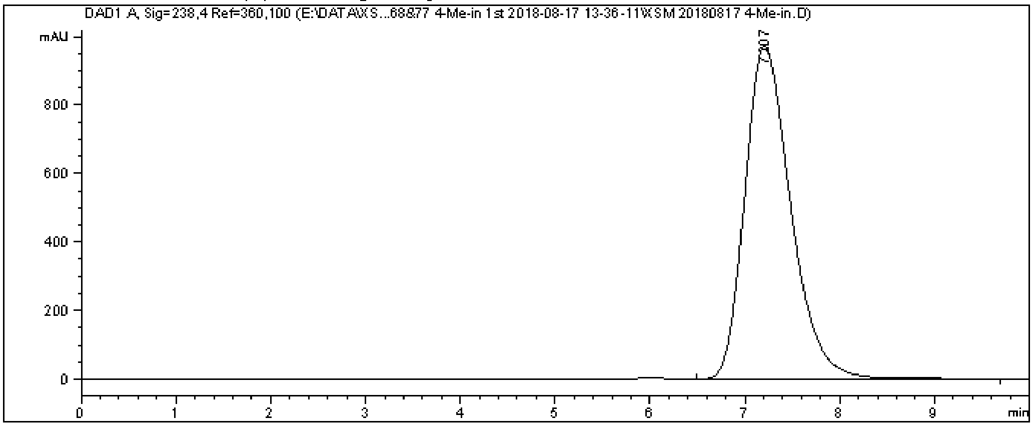


```
    =====================================================================
                             Area Percent Report
    =====================================================================

    Sorted By             :      Signal
    Multiplier            :      1.0000
    Dilution              :      1.0000
    Do not use Multiplier & Dilution Factor with ISTDs


    Signal 1: DAD1 A, Sig=238,4 Ref=360,100

    Peak RetTime Type  Width     Area      Height     Area
      #   [min]        [min]   [mAU*s]     [mAU]        %
    ----|-------|----|-------|----------|----------|--------|
       1   7.207 BB    0.5156 3.28032e4  972.28595 100.0000

    Totals :                  3.28032e4  972.28595


    =====================================================================
                              *** End of Report ***
```


Supplementary Figure 183. HPLC spectrum of (1*R*,3*S*,4*R*)-**3B**

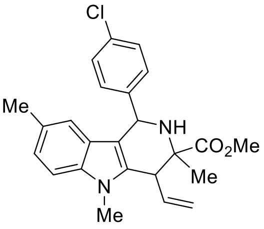

(1*R**,3*S**,4*R**)-**3C**

```
Data File E:\DATA\XS... 03-75 567-Me-in 1st 2018-08-15 12-49-10\In33 20180815 567-Me-in1.D
Sample Name: In33 03-73 5-Me-in 1st rac

=====================================================================
Acq. Operator   : SYSTEM                        Seq. Line :    2
Acq. Instrument : 1260                           Location :   62
Injection Date  : 8/15/2018 1:12:07 PM                Inj :    1
                                               Inj Volume : 1.000 µl
Acq. Method     : E:\DATA\XSM-In33\In33 180815 03-75 567-Me-in 1st 2018-08-15 12-49-10\In33
                  As-98-1mL-238nm-20min-noD 567-Me-in.M
Last changed    : 8/15/2018 12:49:10 PM by SYSTEM
Analysis Method : E:\DATA\XSM-In33\In33 180815 03-75 567-Me-in 1st 2018-08-15 12-49-10\In33
                  As-98-1mL-238nm-20min-noD 567-Me-in.M (Sequence Method)
Last changed    : 10/7/2018 8:33:41 PM by SYSTEM
                  (modified after loading)
Additional Info : Peak(s) manually integrated
```

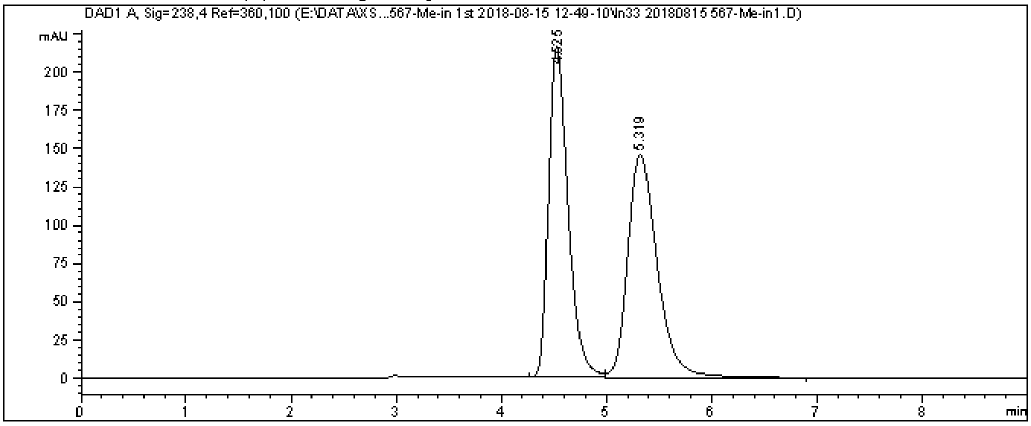


```
=====================================================================
                         Area Percent Report
=====================================================================

Sorted By             :      Signal
Multiplier            :      1.0000
Dilution              :      1.0000
Do not use Multiplier & Dilution Factor with ISTDs


Signal 1: DAD1 A, Sig=238,4 Ref=360,100

Peak RetTime Type  Width     Area      Height     Area
  #   [min]        [min]   [mAU*s]     [mAU]        %
----|-------|----|-------|----------|----------|--------|
   1   4.525 BV    0.1976 2794.13916  216.39355  49.6816
   2   5.319 VB    0.2967 2829.95410  145.63527  50.3184

Totals :                  5624.09326  362.02882


=====================================================================
                          *** End of Report ***
```

1260 10/7/2018 8:35:16 PM SYSTEM   Page 1 of 1

Supplementary Figure 184. HPLC spectrum of (1*R**,3*S**,4*R**)-**3C**

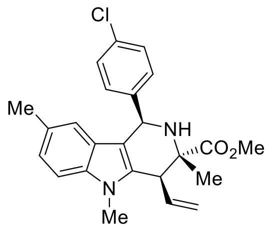

(1*R*,3*S*,4*R*)-**3C**

```
Data File E:\DATA\XS...5 03-75 567-Me-in 1st 2018-08-15 12-49-10\In33 20180815 567-Me-in.D
Sample Name: In33 03-73 5-Me-in R+L opt

=====================================================================
Acq. Operator   : SYSTEM                          Seq. Line :   1
Acq. Instrument : 1260                             Location :   66
Injection Date  : 8/15/2018 12:50:39 PM                 Inj :   1
                                                 Inj Volume : 1.000 µl
Acq. Method     : E:\DATA\XSM-In33\In33 180815 03-75 567-Me-in 1st 2018-08-15 12-49-10\In33
                  As-98-1mL-238nm-20min-noD 567-Me-in.M
Last changed    : 8/15/2018 12:49:10 PM by SYSTEM
Analysis Method : E:\DATA\XSM-In33\In33 180815 03-75 567-Me-in 1st 2018-08-15 12-49-10\In33
                  As-98-1mL-238nm-20min-noD 567-Me-in.M (Sequence Method)
Last changed    : 10/7/2018 8:33:41 PM by SYSTEM
                  (modified after loading)
```

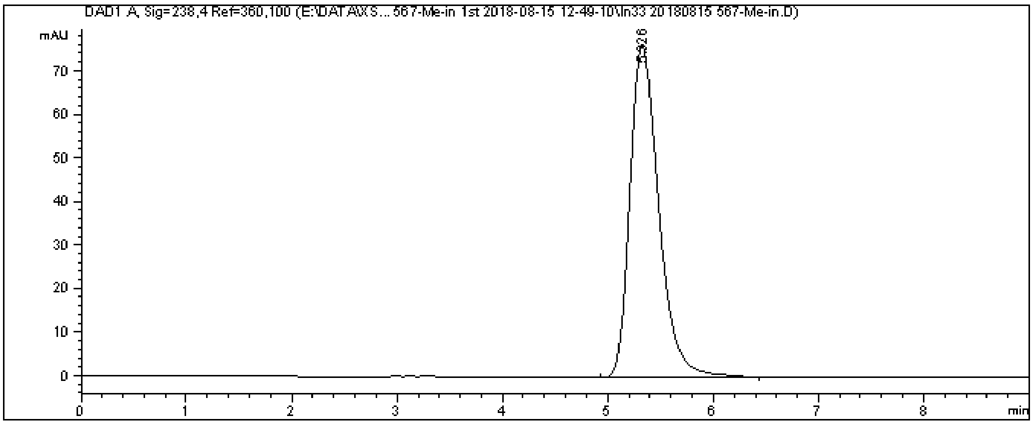


```
=====================================================================
                       Area Percent Report
=====================================================================

Sorted By             :      Signal
Multiplier            :      1.0000
Dilution              :      1.0000
Do not use Multiplier & Dilution Factor with ISTDs


Signal 1: DAD1 A, Sig=238,4 Ref=360,100

Peak RetTime Type  Width     Area      Height     Area
  #   [min]        [min]   [mAU*s]     [mAU]        %
----|-------|----|-------|----------|----------|--------|
   1   5.326 BB    0.2903 1447.38354   76.31286 100.0000

Totals :                  1447.38354   76.31286


=====================================================================
                         *** End of Report ***
```

1260 10/7/2018 8:35:37 PM SYSTEM Page 1 of 1

Supplementary Figure 185. HPLC spectrum of (1*R*,3*S*,4*R*)-**3C**

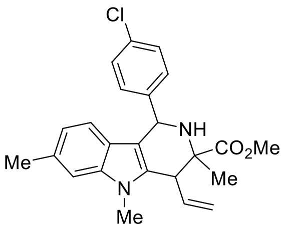

(1*R**,3*S**,4*R**)-**3D**

```
Data File E:\DATA\XS... 03-75 567-Me-in 1st 2018-08-15 12-49-10\In33 20180815 567-Me-in3.D
Sample Name: In33 03-73 6-Me-in 1st rac

  =====================================================================
  Acq. Operator   : SYSTEM                        Seq. Line :    4
  Acq. Instrument : 1260                           Location :   63
  Injection Date  : 8/15/2018 1:55:06 PM                Inj :    1
                                                 Inj Volume : 1.000 µl
  Acq. Method     : E:\DATA\XSM-In33\In33 180815 03-75 567-Me-in 1st 2018-08-15 12-49-10\In33
                    As-98-1mL-238nm-20min-noD 567-Me-in.M
  Last changed    : 8/15/2018 12:49:10 PM by SYSTEM
  Analysis Method : E:\DATA\XSM-In33\In33 180815 03-75 567-Me-in 1st 2018-08-15 12-49-10\In33
                    As-98-1mL-238nm-20min-noD 567-Me-in.M (Sequence Method)
  Last changed    : 10/7/2018 8:33:41 PM by SYSTEM
                    (modified after loading)
  Additional Info : Peak(s) manually integrated
```

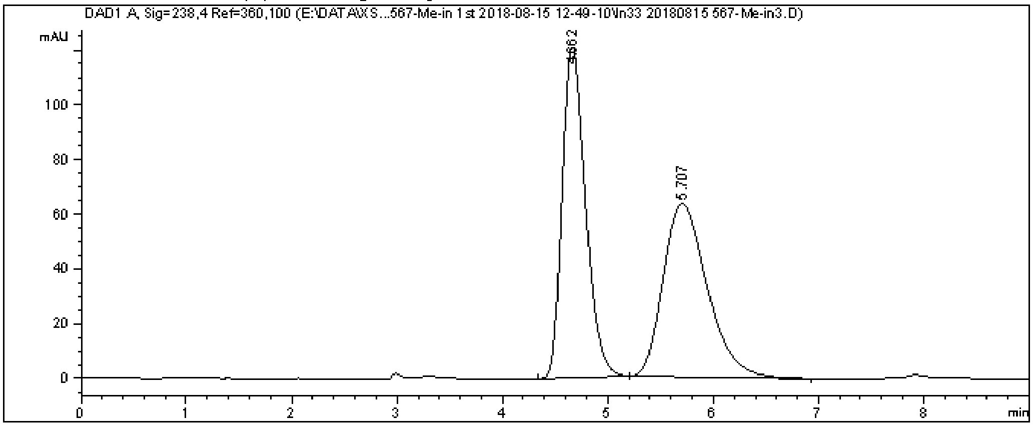


```
  =====================================================================
                          Area Percent Report
  =====================================================================

  Sorted By             :      Signal
  Multiplier            :      1.0000
  Dilution              :      1.0000
  Do not use Multiplier & Dilution Factor with ISTDs


  Signal 1: DAD1 A, Sig=238,4 Ref=360,100

  Peak RetTime Type  Width     Area      Height     Area
    #   [min]        [min]   [mAU*s]     [mAU]        %
  ----|-------|----|-------|----------|----------|--------|
     1   4.662 BB    0.2367 1867.12244  121.20483  50.3574
     2   5.707 BB    0.4417 1840.61865   63.39560  49.6426

  Totals :                  3707.74109  184.60043


  =====================================================================
                           *** End of Report ***
```


Supplementary Figure 186. HPLC spectrum of (1*R**,3*S**,4*R**)-**3D**

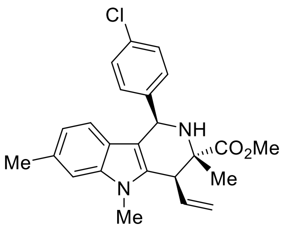

(1*R*,3*S*,4*R*)-**3D**

```
Data File E:\DATA\XS... 03-75 567-Me-in 1st 2018-08-15 12-49-10\In33 20180815 567-Me-in2.D
Sample Name: In33 03-74 6-Me-in R+L opt

    =====================================================================
    Acq. Operator   : SYSTEM                         Seq. Line :    3
    Acq. Instrument : 1260                            Location :   67
    Injection Date  : 8/15/2018 1:33:38 PM                 Inj :    1
                                                    Inj Volume : 1.000 µl
    Acq. Method     : E:\DATA\XSM-In33\In33 180815 03-75 567-Me-in 1st 2018-08-15 12-49-10\In33
                      As-98-1mL-238nm-20min-noD 567-Me-in.M
    Last changed    : 8/15/2018 12:49:10 PM by SYSTEM
    Analysis Method : E:\DATA\XSM-In33\In33 180815 03-75 567-Me-in 1st 2018-08-15 12-49-10\In33
                      As-98-1mL-238nm-20min-noD 567-Me-in.M (Sequence Method)
    Last changed    : 10/7/2018 8:33:41 PM by SYSTEM
                      (modified after loading)
    Additional Info : Peak(s) manually integrated
```

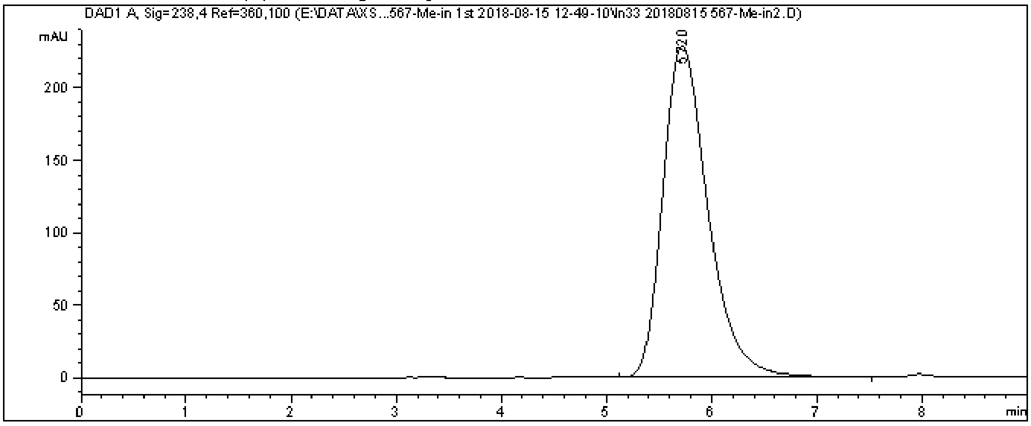


```
    =====================================================================
                             Area Percent Report
    =====================================================================

    Sorted By             :      Signal
    Multiplier            :      1.0000
    Dilution              :      1.0000
    Do not use Multiplier & Dilution Factor with ISTDs


    Signal 1: DAD1 A, Sig=238,4 Ref=360,100

    Peak RetTime Type  Width     Area      Height     Area
      #   [min]        [min]   [mAU*s]     [mAU]        %
    ----|-------|----|-------|----------|----------|--------|
       1   5.720 BB    0.4514 6790.19629  228.06514 100.0000

    Totals :                  6790.19629  228.06514


    =====================================================================
                              *** End of Report ***
```


Supplementary Figure 187. HPLC spectrum of (1*R*,3*S*,4*R*)-**3D**

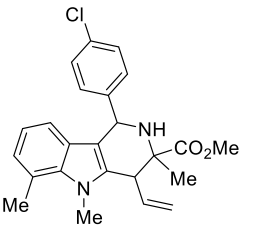

(1*R**,3*S**,4*R**)-**3E**

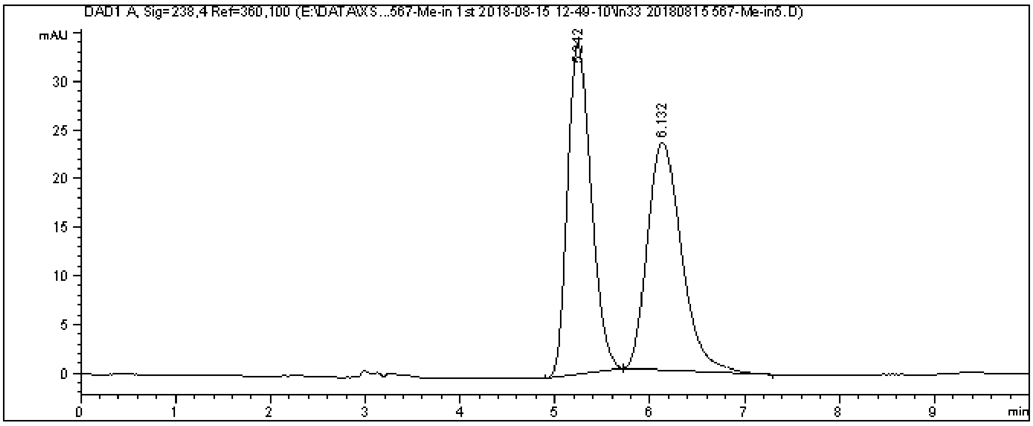

```
Data File E:\DATA\XS... 03-75 567-Me-in 1st 2018-08-15 12-49-10\In33 20180815 567-Me-in5.D
Sample Name: In33 03-73 7-Me-in 1st rac

    =====================================================================
    Acq. Operator   : SYSTEM                        Seq. Line :   6
    Acq. Instrument : 1260                           Location :   64
    Injection Date  : 8/15/2018 2:38:09 PM                Inj :   1
                                                   Inj Volume : 1.000 µl
    Acq. Method     : E:\DATA\XSM-In33\In33 180815 03-75 567-Me-in 1st 2018-08-15 12-49-10\In33
                      As-98-1mL-238nm-20min-noD 567-Me-in.M
    Last changed    : 8/15/2018 12:49:10 PM by SYSTEM
    Analysis Method : E:\DATA\XSM-In33\In33 180815 03-75 567-Me-in 1st 2018-08-15 12-49-10\In33
                      As-98-1mL-238nm-20min-noD 567-Me-in.M (Sequence Method)
    Last changed    : 10/7/2018 8:31:47 PM by SYSTEM
                      (modified after loading)
```



```
    =====================================================================
                             Area Percent Report
    =====================================================================

    Sorted By             :      Signal
    Multiplier            :      1.0000
    Dilution              :      1.0000
    Do not use Multiplier & Dilution Factor with ISTDs


    Signal 1: DAD1 A, Sig=238,4 Ref=360,100

    Peak RetTime Type  Width     Area      Height     Area
      #   [min]        [min]   [mAU*s]     [mAU]        %
    ----|-------|----|-------|----------|----------|--------|
       1   5.242 BB    0.2635  587.10394   33.82989  50.0286
       2   6.132 BB    0.3811  586.43256   23.41905  49.9714

    Totals :                   1173.53650   57.24894


    =====================================================================
                              *** End of Report ***
```

1260 10/7/2018 8:31:53 PM SYSTEM Page 1 of 1

Supplementary Figure 188. HPLC spectrum of (1*R**,3*S**,4*R**)-**3E**

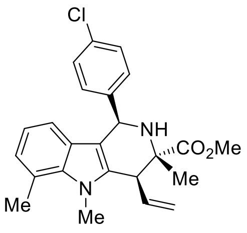

(1*R*,3*S*,4*R*)-**3E**

```
Data File E:\DATA\XS... 03-75 567-Me-in 1st 2018-08-15 12-49-10\In33 20180815 567-Me-in4.D
Sample Name: In33 03-74 7-Me-in R+L opt

    =====================================================================
    Acq. Operator   : SYSTEM                        Seq. Line :    5
    Acq. Instrument : 1260                           Location :   68
    Injection Date  : 8/15/2018 2:16:38 PM                Inj :    1
                                                   Inj Volume : 1.000 µl
    Acq. Method     : E:\DATA\XSM-In33\In33 180815 03-75 567-Me-in 1st 2018-08-15 12-49-10\In33
                      As-98-1mL-238nm-20min-noD 567-Me-in.M
    Last changed    : 8/15/2018 12:49:10 PM by SYSTEM
    Analysis Method : E:\DATA\XSM-In33\In33 180815 03-75 567-Me-in 1st 2018-08-15 12-49-10\In33
                      As-98-1mL-238nm-20min-noD 567-Me-in.M (Sequence Method)
    Last changed    : 10/7/2018 8:32:22 PM by SYSTEM
                      (modified after loading)
    Additional Info : Peak(s) manually integrated
```

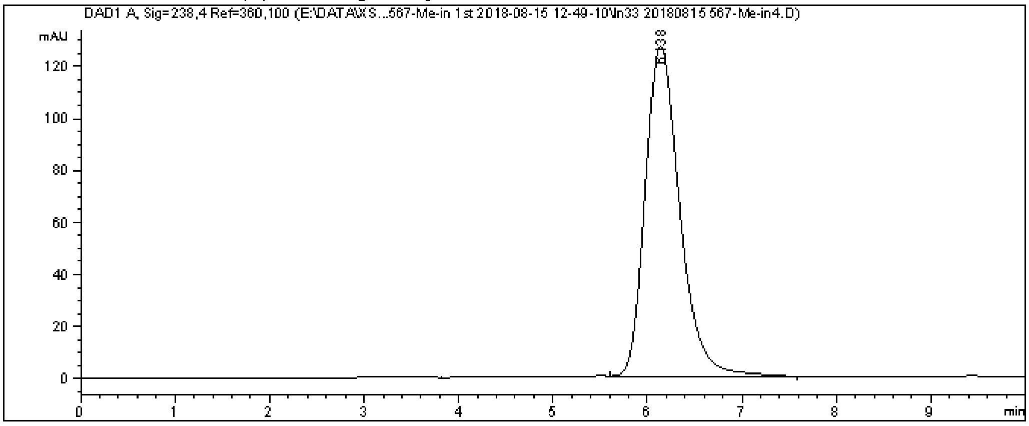


```
    =====================================================================
                             Area Percent Report
    =====================================================================

    Sorted By             :      Signal
    Multiplier            :      1.0000
    Dilution              :      1.0000
    Do not use Multiplier & Dilution Factor with ISTDs


    Signal 1: DAD1 A, Sig=238,4 Ref=360,100

    Peak RetTime Type  Width     Area      Height     Area
      #   [min]        [min]   [mAU*s]     [mAU]        %
    ----|-------|----|-------|----------|----------|--------|
       1   6.138 BB    0.3885 3212.19531  126.82314 100.0000

    Totals :                  3212.19531  126.82314


    =====================================================================
                              *** End of Report ***
```

1260 10/7/2018 8:32:24 PM SYSTEM Page 1 of 1

Supplementary Figure 189. HPLC spectrum of (1*R*,3*S*,4*R*)-**3E**

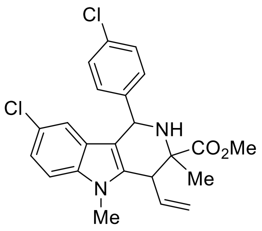

(1*R**,3*S**,4*R**)-**3F**

Data File E:\DATA\XS...180719 03-65 5-Cl&Br-in 1st rac 2018-07-20 00-05-23\XSM 201807193.D
Sample Name: In33 03-65 5-Cl-in 1st rac

===

Acq. Operator : SYSTEM Seq. Line : 4
Acq. Instrument : 1260 Location : 87
Injection Date : 7/20/2018 12:49:11 AM Inj : 1
Inj Volume : 5.000 µl
Acq. Method : E:\DATA\XSM\In33 180719 03-65 5-Cl&Br-in 1st rac 2018-07-20 00-05-23\In33 5-Cl-in 1st AS-98-1mL-248nm-noD.M
Last changed : 7/20/2018 12:05:23 AM by SYSTEM
Analysis Method : E:\DATA\XSM\In33 180719 03-65 5-Cl&Br-in 1st rac 2018-07-20 00-05-23\In33 5-Cl-in 1st AS-98-1mL-248nm-noD.M (Sequence Method)
Last changed : 10/7/2018 9:37:32 PM by SYSTEM
(modified after loading)
Additional Info : Peak(s) manually integrated

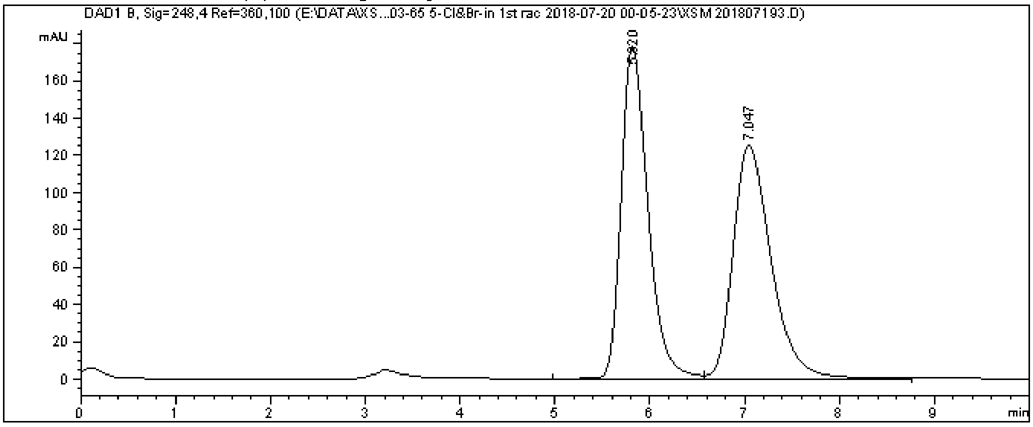

===

Area Percent Report

===

Sorted By : Signal
Multiplier : 1.0000
Dilution : 1.0000
Do not use Multiplier & Dilution Factor with ISTDs

Signal 1: DAD1 B, Sig=248,4 Ref=360,100

Peak #	RetTime [min]	Type	Width [min]	Area [mAU*s]	Height [mAU]	Area %
1	5.820	BV	0.3005	3499.47021	178.64288	50.1244
2	7.047	VB	0.4257	3482.09717	125.50950	49.8756

Totals : 6981.56738 304.15238

===

*** End of Report ***

1260 10/7/2018 9:39:19 PM SYSTEM Page 1 of 1

Supplementary Figure 190. HPLC spectrum of (1*R**,3*S**,4*R**)-**3F**

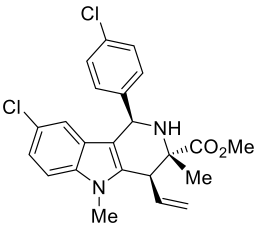

(1*R*,3*S*,4*R*)-**3F**

```
Data File E:\DATA\XS...180719 03-65 5-Cl&Br-in 1st rac 2018-07-20 00-05-23\XSM 201807192.D
Sample Name: In33 03-64 5-Cl-in R+L opt

    =====================================================================
    Acq. Operator   : SYSTEM                          Seq. Line :    3
    Acq. Instrument : 1260                             Location :   88
    Injection Date  : 7/20/2018 12:38:17 AM                 Inj :    1
                                                 Inj Volume : 5.000 µl
    Acq. Method     : E:\DATA\XSM\In33 180719 03-65 5-Cl&Br-in 1st rac 2018-07-20 00-05-23\In33 5
                      -Cl-in 1st AS-98-1mL-248nm-noD.M
    Last changed    : 7/20/2018 12:05:23 AM by SYSTEM
    Analysis Method : E:\DATA\XSM\In33 180719 03-65 5-Cl&Br-in 1st rac 2018-07-20 00-05-23\In33 5
                      -Cl-in 1st AS-98-1mL-248nm-noD.M (Sequence Method)
    Last changed    : 10/7/2018 9:37:32 PM by SYSTEM
                      (modified after loading)
    Additional Info : Peak(s) manually integrated
```

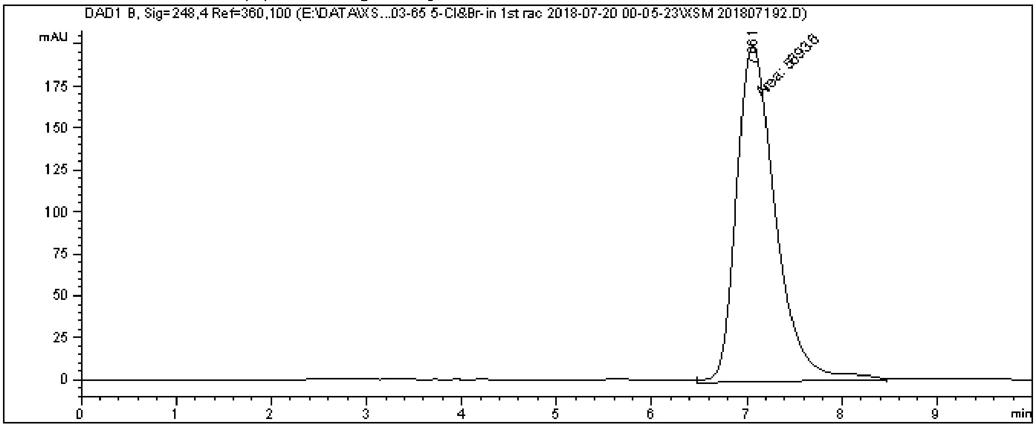


```
    =====================================================================
                             Area Percent Report
    =====================================================================

    Sorted By             :      Signal
    Multiplier            :      1.0000
    Dilution              :      1.0000
    Do not use Multiplier & Dilution Factor with ISTDs


    Signal 1: DAD1 B, Sig=248,4 Ref=360,100

    Peak RetTime Type  Width     Area      Height     Area
      #   [min]        [min]   [mAU*s]     [mAU]        %
    ----|-------|----|-------|----------|----------|--------|
       1   7.061 MM    0.4749 5693.60254  199.81715 100.0000

    Totals :                  5693.60254  199.81715


    =====================================================================
                              *** End of Report ***
```

1260 10/7/2018 9:38:50 PM SYSTEM

Page 1 of 1

Supplementary Figure 191. HPLC spectrum of (1*R*,3*S*,4*R*)-**3F**

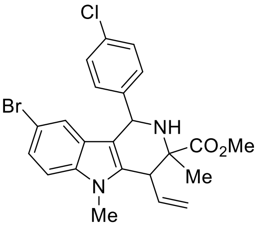

(1*R**,3*S**,4*R**)-**3G**

```
Data File E:\DATA\XS...180721 03-65 5-Cl&Br-in 1st rac 2018-07-21 00-39-20\XSM 201807191.D
Sample Name: In33 03-65 5-Br-in 1st rac

    =====================================================================
    Acq. Operator   : SYSTEM                         Seq. Line :   2
    Acq. Instrument : 1260                            Location :   89
    Injection Date  : 7/21/2018 1:01:15 AM                 Inj :   1
                                                    Inj Volume : 5.000 µl
    Acq. Method     : E:\DATA\XSM\In33 180721 03-65 5-Cl&Br-in 1st rac 2018-07-21 00-39-20\In33 5
                      -Br-in 1st AS-98-1mL-246nm-noD.M
    Last changed    : 7/21/2018 12:39:20 AM by SYSTEM
    Analysis Method : E:\DATA\XSM\In33 180721 03-65 5-Cl&Br-in 1st rac 2018-07-21 00-39-20\In33 5
                      -Br-in 1st AS-98-1mL-246nm-noD.M (Sequence Method)
    Last changed    : 10/7/2018 9:17:25 PM by SYSTEM
                      (modified after loading)
    Additional Info : Peak(s) manually integrated
```

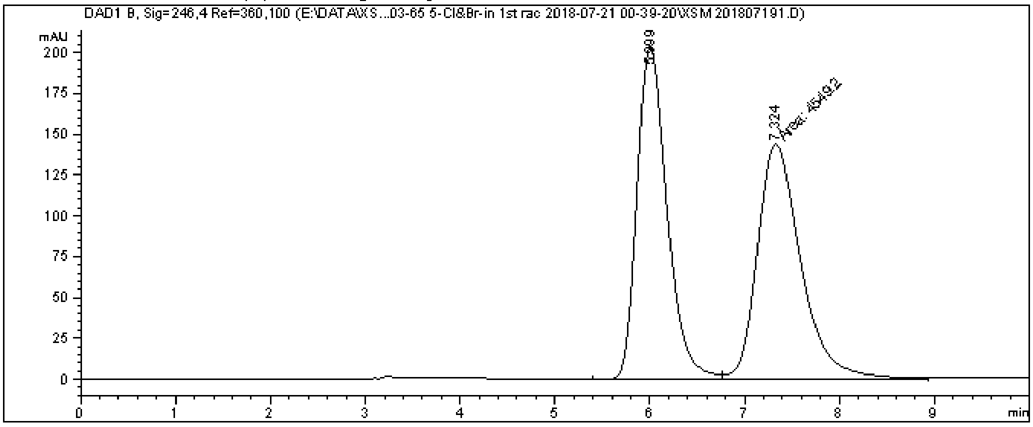


```
    =====================================================================
                             Area Percent Report
    =====================================================================

    Sorted By             :      Signal
    Multiplier            :      1.0000
    Dilution              :      1.0000
    Do not use Multiplier & Dilution Factor with ISTDs


    Signal 1: DAD1 B, Sig=246,4 Ref=360,100

    Peak RetTime Type  Width     Area      Height     Area
      #   [min]        [min]   [mAU*s]     [mAU]        %
    ----|-------|----|-------|----------|----------|--------|
       1   5.999 BV    0.3379 4472.33398  203.93315  49.5740
       2   7.324 MF    0.5267 4549.20020  143.96623  50.4260

    Totals :                  9021.53418  347.89938


    =====================================================================
                              *** End of Report ***
```

1260 10/7/2018 9:17:35 PM SYSTEM Page 1 of 1

Supplementary Figure 192. HPLC spectrum of (1*R**,3*S**,4*R**)-**3G**

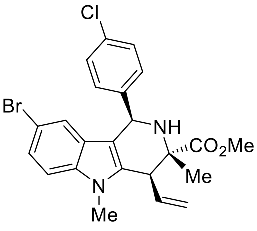

(1*R*,3*S*,4*R*)-**3G**

```
Data File E:\DATA\XS... 180721 03-65 5-Cl&Br-in 1st rac 2018-07-21 00-39-20\XSM 20180719.D
Sample Name: In33 03-64 5-Br-in R+L opt

=====================================================================
Acq. Operator   : SYSTEM                          Seq. Line :   1
Acq. Instrument : 1260                             Location :   90
Injection Date  : 7/21/2018 12:40:16 AM                 Inj :   1
                                                 Inj Volume : 5.000 µl
Acq. Method     : E:\DATA\XSM\In33 180721 03-65 5-Cl&Br-in 1st rac 2018-07-21 00-39-20\In33 5
                  -Br-in 1st AS-98-1mL-246nm-noD.M
Last changed    : 7/21/2018 12:39:20 AM by SYSTEM
Analysis Method : E:\DATA\XSM\In33 180721 03-65 5-Cl&Br-in 1st rac 2018-07-21 00-39-20\In33 5
                  -Br-in 1st AS-98-1mL-246nm-noD.M (Sequence Method)
Last changed    : 1/4/2019 3:44:49 PM by SYSTEM
                  (modified after loading)
Additional Info : Peak(s) manually integrated
```

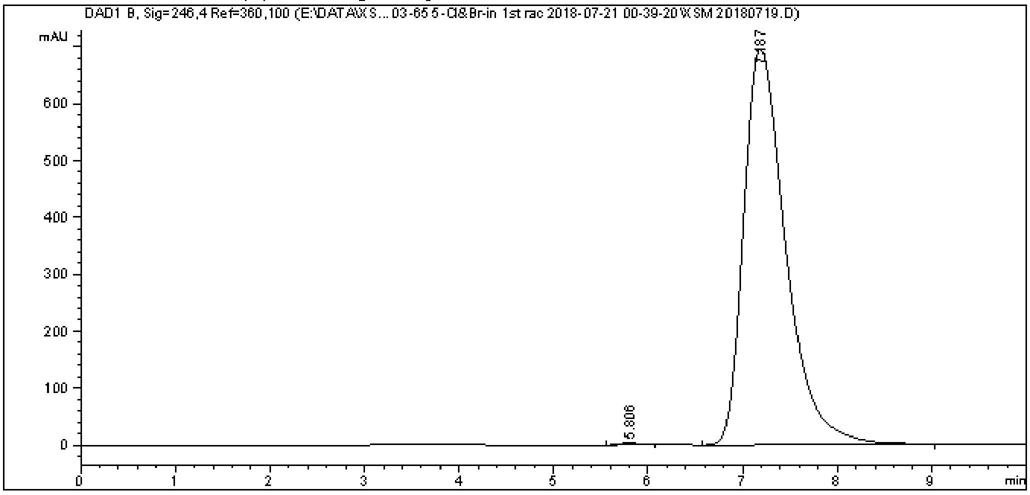


```
=====================================================================
                         Area Percent Report
=====================================================================

Sorted By             :      Signal
Multiplier            :      1.0000
Dilution              :      1.0000
Do not use Multiplier & Dilution Factor with ISTDs


Signal 1: DAD1 B, Sig=246,4 Ref=360,100

Peak RetTime Type  Width     Area      Height     Area
  #   [min]        [min]   [mAU*s]     [mAU]        %
----|-------|----|-------|----------|----------|--------|
   1   5.806 BB    0.2429   37.00833    2.49690   0.1722
   2   7.187 BB    0.4722 2.14517e4  694.81281  99.8278

Totals :                  2.14887e4  697.30971


=====================================================================
                          *** End of Report ***
```


Supplementary Figure 193. HPLC spectrum of (1*R*,3*S*,4*R*)-**3G**

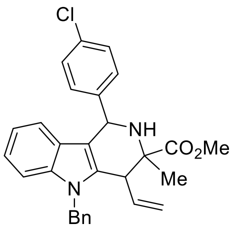

(1*R**,3*S**,4*R**)-**3H**

Data File E:\DATA\XSM\In33 180714 03-61 N-Bn 1st 2018-07-14 20-53-39\XSM-201807141.D
Sample Name: In33 03-61 N-Bn 1st rac

```
=====================================================================
Acq. Operator   : SYSTEM                          Seq. Line :    2
Acq. Instrument : 1260                             Location :    7
Injection Date  : 7/14/2018 9:15:39 PM                  Inj :    1
                                                 Inj Volume : 15.000 µl
Acq. Method     : E:\DATA\XSM\In33 180714 03-61 N-Bn 1st 2018-07-14 20-53-39\In33 1st AS-98-0
                  .5mL-228nm N-Bn.M
Last changed    : 7/14/2018 8:53:39 PM by SYSTEM
Analysis Method : E:\DATA\XSM\In33 180714 03-61 N-Bn 1st 2018-07-14 20-53-39\In33 1st AS-98-0
                  .5mL-228nm N-Bn.M (Sequence Method)
Last changed    : 10/7/2018 9:43:59 PM by SYSTEM
                  (modified after loading)
Additional Info : Peak(s) manually integrated
```

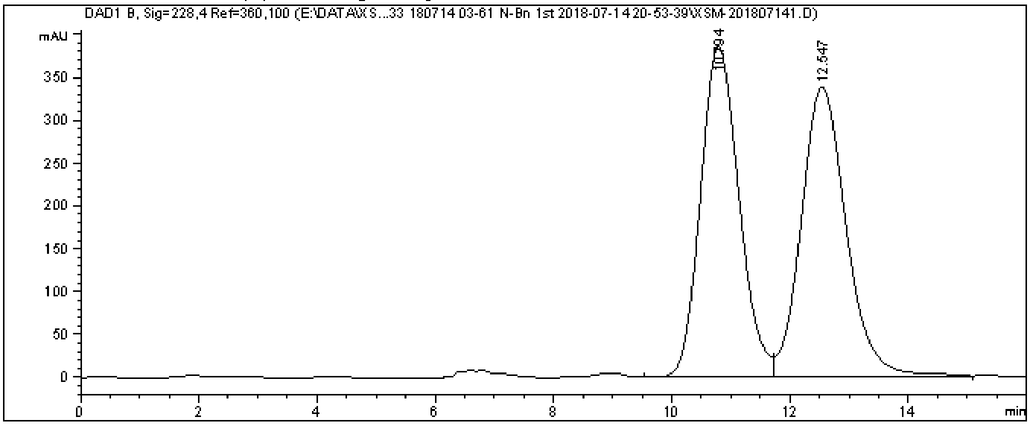


```
=====================================================================
                         Area Percent Report
=====================================================================

Sorted By             :      Signal
Multiplier            :      1.0000
Dilution              :      1.0000
Do not use Multiplier & Dilution Factor with ISTDs


Signal 1: DAD1 B, Sig=228,4 Ref=360,100

Peak RetTime Type  Width     Area      Height     Area
  #   [min]        [min]   [mAU*s]     [mAU]        %
----|-------|----|-------|----------|----------|--------|
   1  10.794 BV    0.6737 1.73875e4  385.85895  49.5282
   2  12.547 VB    0.8059 1.77188e4  337.80804  50.4718

Totals :                  3.51063e4   723.66699


=====================================================================
                          *** End of Report ***
```


Supplementary Figure 194. HPLC spectrum of (1*R**,3*S**,4*R**)-**3H**

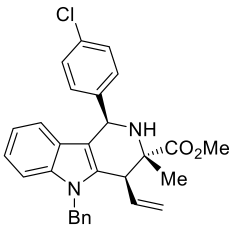

(1*R*,3*S*,4*R*)-**3H**

Data File E:\DATA\XSM\In33 180714 03-61 N-Bn 1st 2018-07-14 20-53-39\XSM-20180714.D
Sample Name: In33 03-58 N-Bn R+L opt

===

Acq. Operator : SYSTEM Seq. Line : 1
Acq. Instrument : 1260 Location : 8
Injection Date : 7/14/2018 8:54:37 PM Inj : 1
Inj Volume : 15.000 µl
Acq. Method : E:\DATA\XSM\In33 180714 03-61 N-Bn 1st 2018-07-14 20-53-39\In33 1st AS-98-0 .5mL-228nm N-Bn.M
Last changed : 7/14/2018 8:53:39 PM by SYSTEM
Analysis Method : E:\DATA\XSM\In33 180714 03-61 N-Bn 1st 2018-07-14 20-53-39\In33 1st AS-98-0 .5mL-228nm N-Bn.M (Sequence Method)
Last changed : 10/7/2018 9:43:59 PM by SYSTEM
(modified after loading)
Additional Info : Peak(s) manually integrated

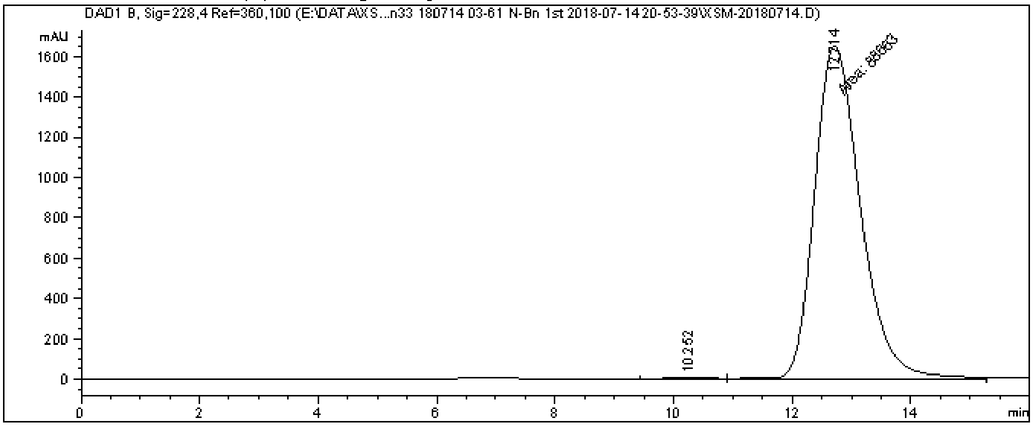

===

Area Percent Report

===

Sorted By : Signal
Multiplier : 1.0000
Dilution : 1.0000
Do not use Multiplier & Dilution Factor with ISTDs

Signal 1: DAD1 B, Sig=228,4 Ref=360,100

Peak #	RetTime [min]	Type	Width [min]	Area [mAU*s]	Height [mAU]	Area %
1	10.252	BB	0.5677	288.47180	7.25743	0.3243
2	12.714	MF	0.8955	8.86630e4	1650.15454	99.6757

Totals : 8.89515e4 1657.41197

===

*** End of Report ***

1260 10/7/2018 9:44:07 PM SYSTEM Page 1 of 1

Supplementary Figure 195. HPLC spectrum of (1*R*,3*S*,4*R*)-**3H**

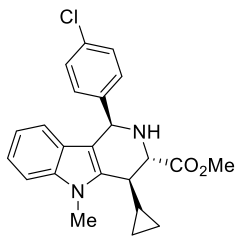

($1R^*,3S^*,4R^*$)-**4**

```
Data File E:\DATA\XSM\In33 190612 aH H2&Cc DAD div 2019-06-12 22-54-46\XSM 201906121.D
Sample Name: In33 04-125 4-Cl-aH CH2N2 4p div

    =====================================================================
    Acq. Operator   : SYSTEM                        Seq. Line :    2
    Acq. Instrument : 1260                           Location :   80
    Injection Date  : 6/12/2019 11:38:09 PM               Inj :    1
                                                   Inj Volume : 5.000 µl
    Acq. Method     : E:\DATA\XSM\In33 190612 aH H2&Cc DAD div 2019-06-12 22-54-46\In33 AD-98-
                      40min-282nm 4-Cl-aH div.M
    Last changed    : 6/12/2019 10:54:48 PM by SYSTEM
    Analysis Method : E:\DATA\XSM\In33 190612 aH H2&Cc DAD div 2019-06-12 22-54-46\In33 AD-98-
                      40min-282nm 4-Cl-aH div.M (Sequence Method)
    Last changed    : 7/12/2019 11:30:29 AM by SYSTEM
                      (modified after loading)
```

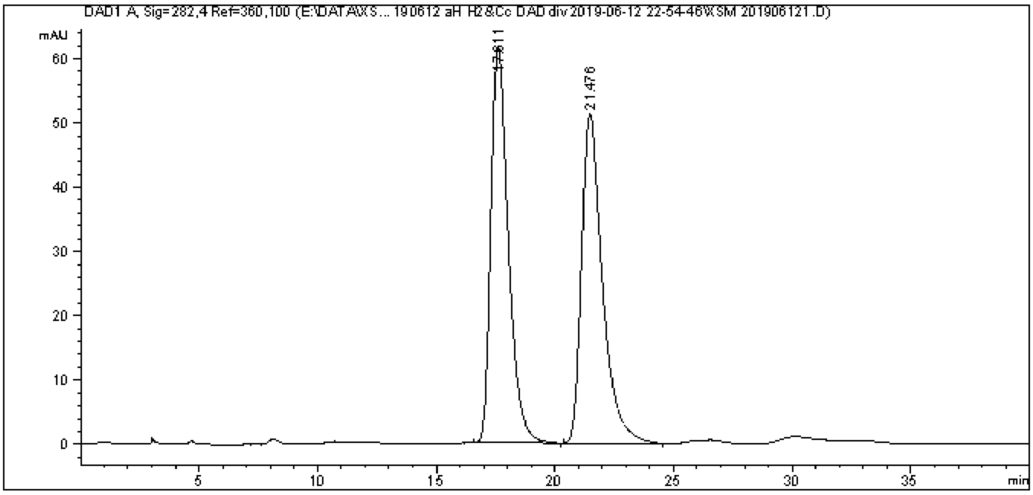


```
    =====================================================================
                         Area Percent Report
    =====================================================================

    Sorted By             :      Signal
    Multiplier            :      1.0000
    Dilution              :      1.0000
    Do not use Multiplier & Dilution Factor with ISTDs


    Signal 1: DAD1 A, Sig=282,4 Ref=360,100

    Peak RetTime Type  Width     Area      Height     Area
      #   [min]        [min]   [mAU*s]     [mAU]        %
    ----|-------|----|-------|----------|----------|--------|
       1  17.611 BB    0.7288 3127.87769   61.35547  49.9906
       2  21.476 BB    0.8468 3129.04858   51.21708  50.0094

    Totals :                  6256.92627  112.57256


    =====================================================================
                          *** End of Report ***
```

1260 7/12/2019 11:30:33 AM SYSTEM   Page 1 of 1

Supplementary Figure 196. HPLC spectrum of ($1R^*,3S^*,4R^*$)-**4**

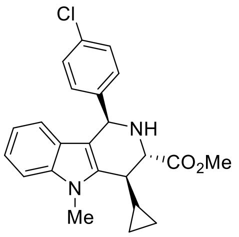

(1*R*,3*S*,4*R*)-**4**

Data File E:\DATA\XSM\In33 190711 04-129 aH HBpin opt 2019-07-11 16-55-26\XSM 201907113.D
Sample Name: In33 04-125 4-Cl-aH CH2N2 R+L SM

===

Acq. Operator : SYSTEM Seq. Line : 4
Acq. Instrument : 1260 Location : 90
Injection Date : 7/11/2019 6:21:00 PM Inj : 1
Inj Volume : 5.000 µl
Acq. Method : E:\DATA\XSM\In33 190711 04-129 aH HBpin opt 2019-07-11 16-55-26\In33 AD-98-40min-282nm 4-Cl-aH div.M
Last changed : 7/11/2019 5:16:13 PM by SYSTEM
Analysis Method : E:\DATA\XSM\In33 190711 04-129 aH HBpin opt 2019-07-11 16-55-26\In33 AD-98-40min-282nm 4-Cl-aH div.M (Sequence Method)
Last changed : 7/11/2019 9:24:31 PM by SYSTEM
(modified after loading)
Additional Info : Peak(s) manually integrated

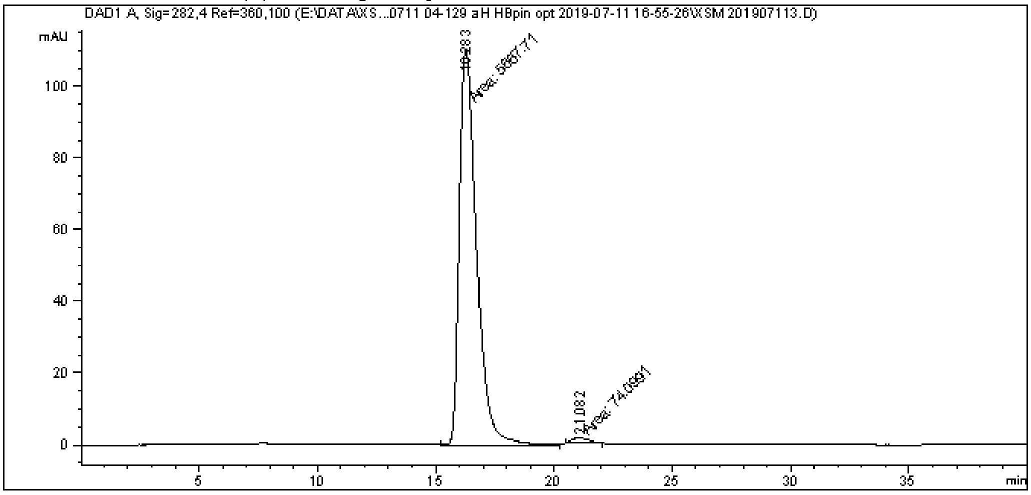

===
Area Percent Report
===

Sorted By : Signal
Multiplier : 1.0000
Dilution : 1.0000
Do not use Multiplier & Dilution Factor with ISTDs

Signal 1: DAD1 A, Sig=282,4 Ref=360,100

Peak #	RetTime [min]	Type	Width [min]	Area [mAU*s]	Height [mAU]	Area %
1	16.283	MM	0.8550	5667.71289	110.48399	98.7095
2	21.082	MM	0.8759	74.09912	1.41000	1.2905

Totals : 5741.81201 111.89399

===
*** End of Report ***

1260 7/11/2019 9:33:16 PM SYSTEM Page 1 of 1

Supplementary Figure 197. HPLC spectrum of (1*R*,3*S*,4*R*)-**4**

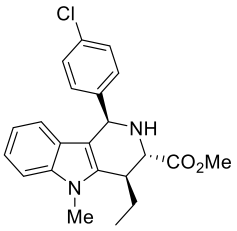

(1*R**,3*S**,4*R**)-**5**

```
Data File E:\DATA\XSM\In33 190713 04-140 4-Cl-aH H2 rac 2019-07-13 23-01-29\XSM 20190713.D
Sample Name: In33 04-140 4-Cl-aH H2 1st rac

    =====================================================================
    Acq. Operator   : SYSTEM                          Seq. Line :   1
    Acq. Instrument : 1260                             Location :   50
    Injection Date  : 7/13/2019 11:03:02 PM                 Inj :   1
                                                     Inj Volume : 5.000 µl
    Acq. Method     : E:\DATA\XSM\In33 190713 04-140 4-Cl-aH H2 rac 2019-07-13 23-01-29\In33 IE-
                      98-20min-232nm 4-Cl-aH H2.M
    Last changed    : 7/13/2019 11:17:48 PM by SYSTEM
                      (modified after loading)
    Analysis Method : E:\DATA\XSM\In33 190713 04-140 4-Cl-aH H2 rac 2019-07-13 23-01-29\In33 IE-
                      98-20min-232nm 4-Cl-aH H2.M (Sequence Method)
    Last changed    : 7/13/2019 11:25:50 PM by SYSTEM
                      (modified after loading)
    Additional Info : Peak(s) manually integrated
```

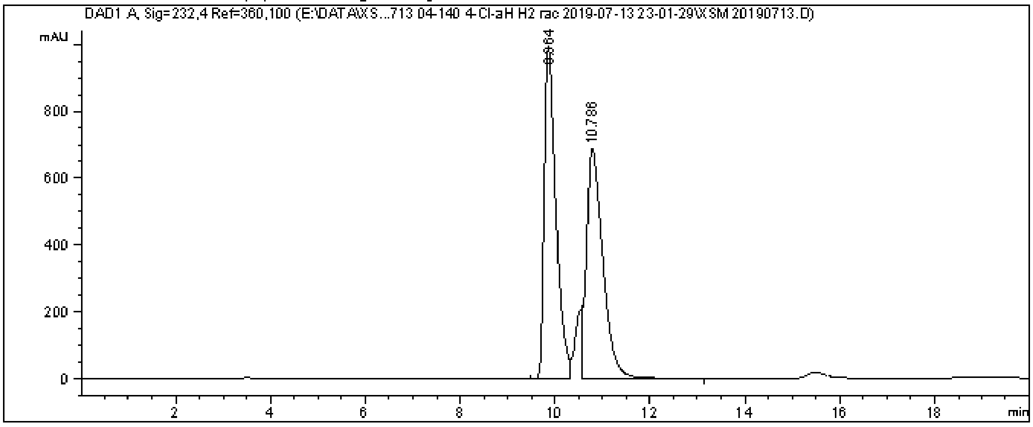


```
    =====================================================================
                             Area Percent Report
    =====================================================================

    Sorted By             :      Signal
    Multiplier            :      1.0000
    Dilution              :      1.0000
    Do not use Multiplier & Dilution Factor with ISTDs


    Signal 1: DAD1 A, Sig=232,4 Ref=360,100

    Peak RetTime Type  Width     Area      Height     Area
      #   [min]        [min]   [mAU*s]     [mAU]        %
    ----|-------|----|-------|----------|----------|--------|
       1   9.864 BV    0.2546 1.69340e4  994.99597  50.3875
       2  10.786 VB    0.3650 1.66735e4  689.77911  49.6125

    Totals :                  3.36075e4  1684.77509


    =====================================================================
                              *** End of Report ***
```

1260 7/13/2019 11:25:53 PM SYSTEM Page 1 of 1

Supplementary Figure 198. HPLC spectrum of (1*R**,3*S**,4*R**)-**5**

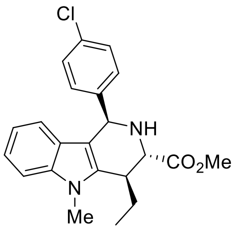

(1*R*,3*S*,4*R*)-**5**

```
Data File E:\DATA\XS... 190618 aH H2&CH2N2&DIBAL-H opt 2019-06-17 23-17-48\XSM 201906122.D
Sample Name: In33 04-78 4-Cl-aH H2 R+L opt

    =====================================================================
    Acq. Operator   : SYSTEM                        Seq. Line :   3
    Acq. Instrument : 1260                           Location :   69
    Injection Date  : 6/18/2019 12:32:41 AM               Inj :   1
                                                  Inj Volume : 5.000 µl
    Acq. Method     : E:\DATA\XSM\In33 190618 aH H2&CH2N2&DIBAL-H opt 2019-06-17 23-17-48\In33 IE
                      -98-20min-232nm 4-Cl-aH H2.M
    Last changed    : 6/17/2019 11:17:49 PM by SYSTEM
    Analysis Method : E:\DATA\XSM\In33 190618 aH H2&CH2N2&DIBAL-H opt 2019-06-17 23-17-48\In33 IE
                      -98-20min-232nm 4-Cl-aH H2.M (Sequence Method)
    Last changed    : 7/13/2019 11:17:30 PM by SYSTEM
                      (modified after loading)
    Additional Info : Peak(s) manually integrated
```

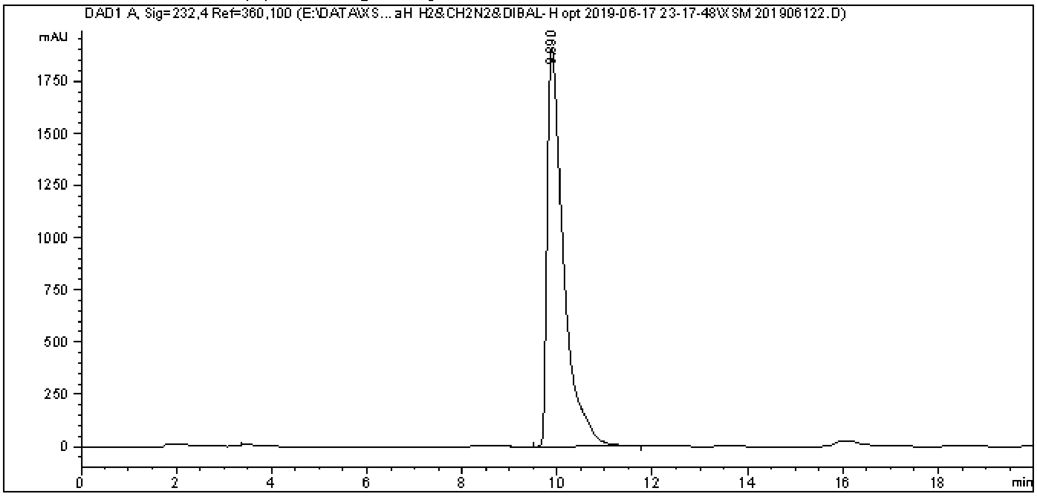


```
    =====================================================================
                             Area Percent Report
    =====================================================================

    Sorted By             :      Signal
    Multiplier            :      1.0000
    Dilution              :      1.0000
    Do not use Multiplier & Dilution Factor with ISTDs


    Signal 1: DAD1 A, Sig=232,4 Ref=360,100

    Peak RetTime Type  Width     Area      Height     Area
      #   [min]        [min]   [mAU*s]     [mAU]        %
    ----|-------|----|-------|----------|----------|--------|
       1   9.890 BB    0.3472 4.55006e4  1901.31982 100.0000

    Totals :                  4.55006e4  1901.31982


    =====================================================================
                              *** End of Report ***

1260 7/13/2019 11:17:32 PM SYSTEM                                         Page 1 of 1
```

Supplementary Figure 199. HPLC spectrum of (1*R*,3*S*,4*R*)-**5**

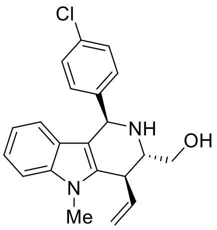

(1*R**,3*S**,4*R**)-**6**

```
Data File E:\DATA\XS...503 04-81&83 aH-DIBAL-H&Ph2N-aH 2019-05-03 20-30-56\XSM 201905031.D
Sample Name: In33 04-83 4-Cl-aH DIBAL-H rac

    =====================================================================
    Acq. Operator   : SYSTEM                          Seq. Line :    2
    Acq. Instrument : 1260                             Location :   70
    Injection Date  : 5/3/2019 9:03:59 PM                   Inj :    1
                                                     Inj Volume : 5.000 µl
    Acq. Method     : E:\DATA\XSM\In33 190503 04-81&83 aH-DIBAL-H&Ph2N-aH 2019-05-03 20-30-56
                      \In33 AD-85-40min-282nm 4-Cl-aH DIBAL-H.M
    Last changed    : 5/3/2019 8:30:56 PM by SYSTEM
    Analysis Method : E:\DATA\XSM\In33 190503 04-81&83 aH-DIBAL-H&Ph2N-aH 2019-05-03 20-30-56
                      \In33 AD-85-40min-282nm 4-Cl-aH DIBAL-H.M (Sequence Method)
    Last changed    : 7/12/2019 11:33:30 AM by SYSTEM
                      (modified after loading)
    Additional Info : Peak(s) manually integrated
```

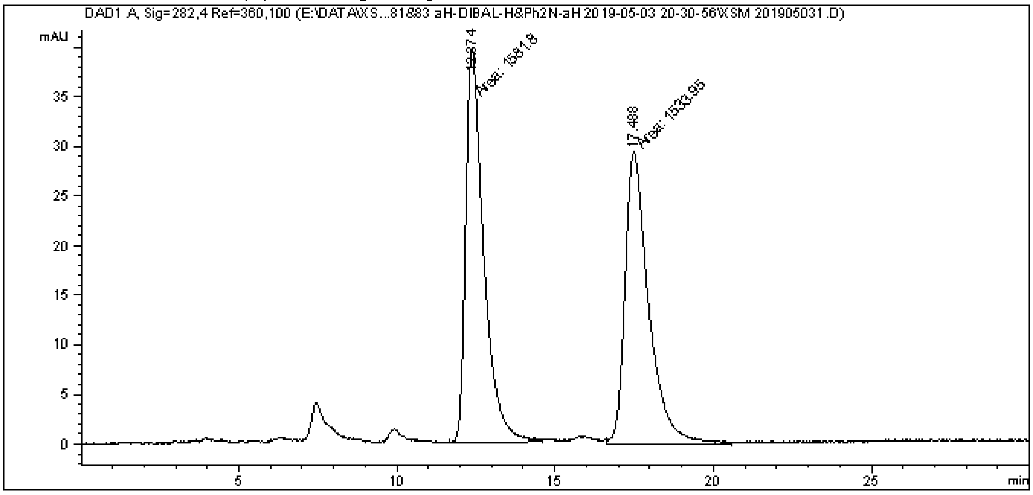


```
    =====================================================================
                         Area Percent Report
    =====================================================================

    Sorted By            :      Signal
    Multiplier           :      1.0000
    Dilution             :      1.0000
    Do not use Multiplier & Dilution Factor with ISTDs


    Signal 1: DAD1 A, Sig=282,4 Ref=360,100

    Peak RetTime Type  Width     Area      Height     Area
      #   [min]        [min]   [mAU*s]     [mAU]        %
    ----|-------|----|-------|----------|----------|--------|
       1  12.374 MM    0.6643 1581.80078   39.68710  50.7679
       2  17.488 MM    0.8704 1533.95032   29.37140  49.2321

    Totals :                  3115.75110   69.05849


    =====================================================================
                           *** End of Report ***

1260 7/12/2019 11:33:33 AM SYSTEM                                     Page 1 of 1
```

Supplementary Figure 200. HPLC spectrum of (1*R**,3*S**,4*R**)-**6**

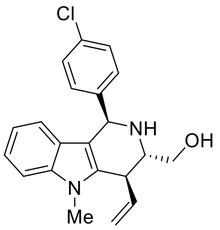

(1*R*,3*S*,4*R*)-**6**

Data File E:\DATA\XSM\In33 190708 aH DIBAL-H R&L opt 2019-07-08 16-38-32\XSM 20190708.D
Sample Name: In33 04-83 4-Cl-aH DIBAL-H R+L opt

```
=====================================================================
Acq. Operator   : SYSTEM                          Seq. Line :   1
Acq. Instrument : 1260                             Location :   90
Injection Date  : 7/8/2019 4:40:04 PM                   Inj :   1
                                                 Inj Volume : 5.000 µl
Acq. Method     : E:\DATA\XSM\In33 190708 aH DIBAL-H R&L opt 2019-07-08 16-38-32\In33 AD-85-
                  40min-282nm 4-Cl-aH DIBAL-H.M
Last changed    : 7/8/2019 4:38:33 PM by SYSTEM
Analysis Method : E:\DATA\XSM\In33 190708 aH DIBAL-H R&L opt 2019-07-08 16-38-32\In33 AD-85-
                  40min-282nm 4-Cl-aH DIBAL-H.M (Sequence Method)
Last changed    : 7/8/2019 7:25:49 PM by SYSTEM
                  (modified after loading)
Additional Info : Peak(s) manually integrated
```

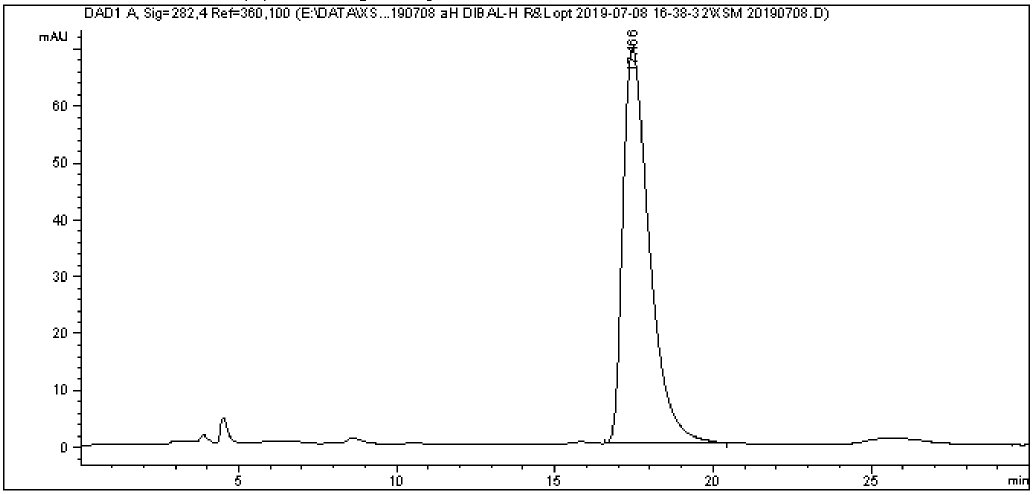


```
=====================================================================
                         Area Percent Report
=====================================================================

Sorted By             :      Signal
Multiplier            :      1.0000
Dilution              :      1.0000
Do not use Multiplier & Dilution Factor with ISTDs


Signal 1: DAD1 A, Sig=282,4 Ref=360,100

Peak RetTime Type  Width     Area      Height     Area
  #   [min]        [min]   [mAU*s]     [mAU]        %
----|-------|----|-------|----------|----------|--------|
   1  17.466 BB    0.8165 4043.36670   69.22164 100.0000

Totals :                  4043.36670   69.22164


=====================================================================
                          *** End of Report ***
```

1260 7/8/2019 7:25:51 PM SYSTEM   Page 1 of 1

Supplementary Figure 201. HPLC spectrum of (1*R*,3*S*,4*R*)-**6**

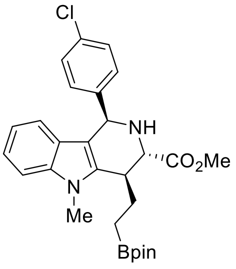

(1*R**,3*S**,4*R**)-**7**

```
Data File E:\DATA\XSM\In33 190709 04-129 4-Cl-aH HBpin 2019-07-09 12-51-58\XSM 201907091.D
Sample Name: In33 04-130 4-Cl-aH HBpin rac

    =====================================================================
    Acq. Operator   : SYSTEM                          Seq. Line :    2
    Acq. Instrument : 1260                             Location :   60
    Injection Date  : 7/9/2019 1:18:55 PM                   Inj :    1
                                                     Inj Volume : 5.000 µl
    Acq. Method     : E:\DATA\XSM\In33 190709 04-129 4-Cl-aH HBpin 2019-07-09 12-51-58\In33 OD-95
                      -228nm-30min aH HBpin.M
    Last changed    : 7/9/2019 1:17:13 PM by SYSTEM
    Analysis Method : E:\DATA\XSM\In33 190709 04-129 4-Cl-aH HBpin 2019-07-09 12-51-58\In33 OD-95
                      -228nm-30min aH HBpin.M (Sequence Method)
    Last changed    : 7/12/2019 11:27:53 AM by SYSTEM
                      (modified after loading)
    Additional Info : Peak(s) manually integrated
```

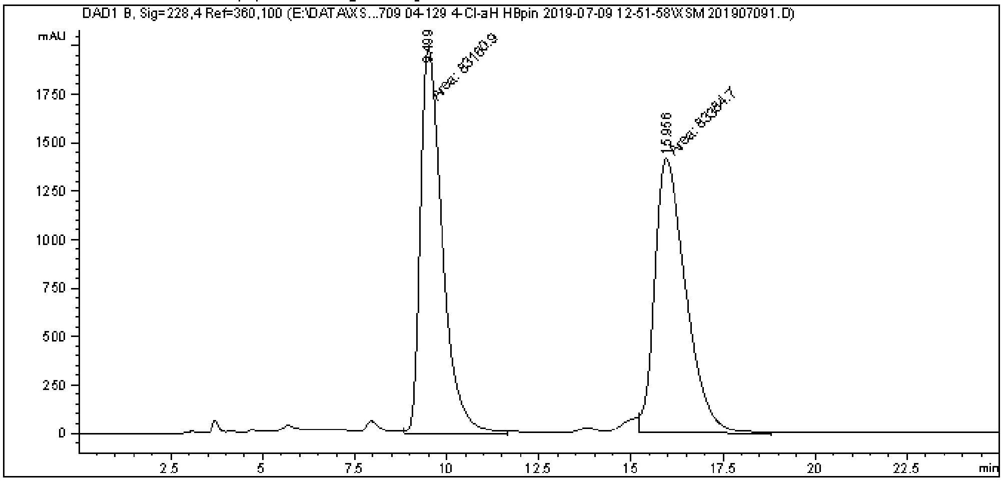


```
    =====================================================================
                             Area Percent Report
    =====================================================================

    Sorted By             :      Signal
    Multiplier            :      1.0000
    Dilution              :      1.0000
    Do not use Multiplier & Dilution Factor with ISTDs


    Signal 1: DAD1 B, Sig=228,4 Ref=360,100

    Peak RetTime Type  Width     Area      Height     Area
      #   [min]        [min]   [mAU*s]     [mAU]        %
    ----|-------|----|-------|----------|----------|--------|
       1   9.499 MM    0.6960 8.31609e4  1991.51025  49.9328
       2  15.956 FM    0.9812 8.33847e4  1416.34082  50.0672

    Totals :                  1.66546e5  3407.85107


    =====================================================================
                             *** End of Report ***

1260 7/12/2019 11:27:56 AM SYSTEM                                          Page 1 of 1
```

Supplementary Figure 202. HPLC spectrum of (1*R**,3*S**,4*R**)-**7**

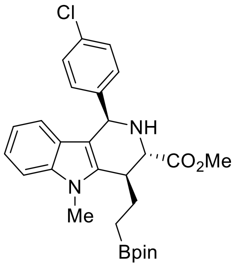

(1*R*,3*S*,4*R*)-**7**

```
Data File E:\DATA\XSM\In33 190711 04-129 aH HBpin opt 2019-07-11 16-55-26\XSM 20190711.D
Sample Name: In33 04-129 4-Cl-aH HBpin R+L opt

    =====================================================================
    Acq. Operator   : SYSTEM                          Seq. Line :    1
    Acq. Instrument : 1260                             Location :   89
    Injection Date  : 7/11/2019 4:56:22 PM                  Inj :    1
                                                     Inj Volume : 5.000 µl
    Acq. Method     : E:\DATA\XSM\In33 190711 04-129 aH HBpin opt 2019-07-11 16-55-26\In33 OD-95-
                      228nm-30min aH HBpin.M
    Last changed    : 7/11/2019 4:55:26 PM by SYSTEM
    Analysis Method : E:\DATA\XSM\In33 190711 04-129 aH HBpin opt 2019-07-11 16-55-26\In33 OD-95-
                      228nm-30min aH HBpin.M (Sequence Method)
    Last changed    : 7/11/2019 9:36:32 PM by SYSTEM
                      (modified after loading)
    Additional Info : Peak(s) manually integrated
```

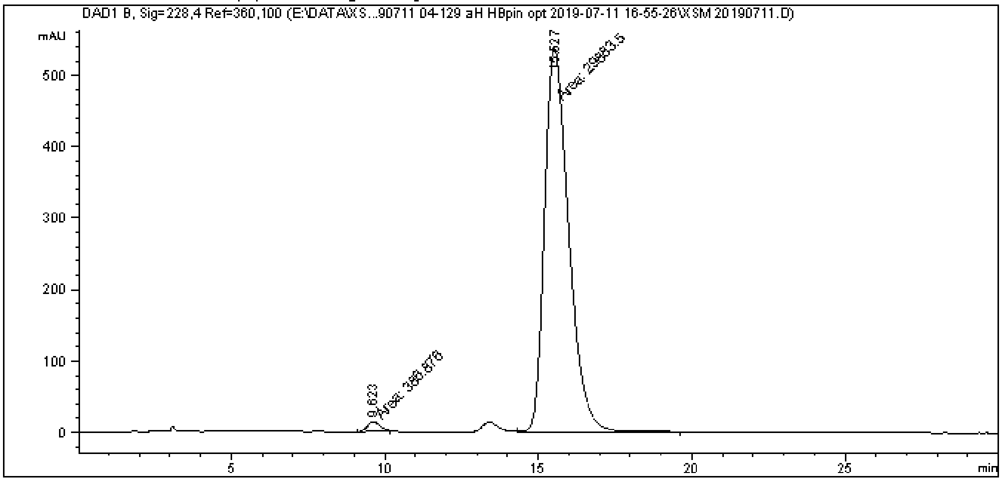


```
    =====================================================================
                             Area Percent Report
    =====================================================================

    Sorted By             :      Signal
    Multiplier            :      1.0000
    Dilution              :      1.0000
    Do not use Multiplier & Dilution Factor with ISTDs


    Signal 1: DAD1 B, Sig=228,4 Ref=360,100

    Peak RetTime Type  Width     Area      Height     Area
      #   [min]        [min]   [mAU*s]     [mAU]        %
    ----|-------|----|-------|----------|----------|--------|
       1   9.623 MM    0.4774  386.87567   13.50646   1.2781
       2  15.527 MM    0.9265 2.98835e4   537.56525  98.7219

    Totals :                  3.02704e4   551.07171


    =====================================================================
                              *** End of Report ***
```

1260 7/11/2019 9:39:27 PM SYSTEM   Page 1 of 1

Supplementary Figure 203. HPLC spectrum of (1*R*,3*S*,4*R*)-**7**

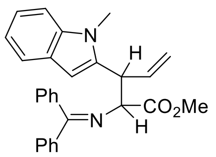

(2*R**,3*S**)-**9** and (2*R**,3*R**)-**9**

Data File E:\DATA\XSM\In33 190426 04-81 Ph2N-aH OD 1st 2019-04-26 22-32-16\XSM 201904262.D
Sample Name: In33 04-81 Ph2N-aH 1st rac

===

Acq. Operator : SYSTEM Seq. Line : 3
Acq. Instrument : 1260 Location : 80
Injection Date : 4/26/2019 11:36:47 PM Inj : 1
Inj Volume : 5.000 µl
Acq. Method : E:\DATA\XSM\In33 190426 04-81 Ph2N-aH OD 1st 2019-04-26 22-32-16\In33 OD-98-1mL-226nm-30min Ph2N-aH 1st.M
Last changed : 4/26/2019 10:32:16 PM by SYSTEM
Analysis Method : E:\DATA\XSM\In33 190426 04-81 Ph2N-aH OD 1st 2019-04-26 22-32-16\In33 OD-98-1mL-226nm-30min Ph2N-aH 1st.M (Sequence Method)
Last changed : 6/7/2019 4:07:04 PM by SYSTEM
(modified after loading)
Additional Info : Peak(s) manually integrated

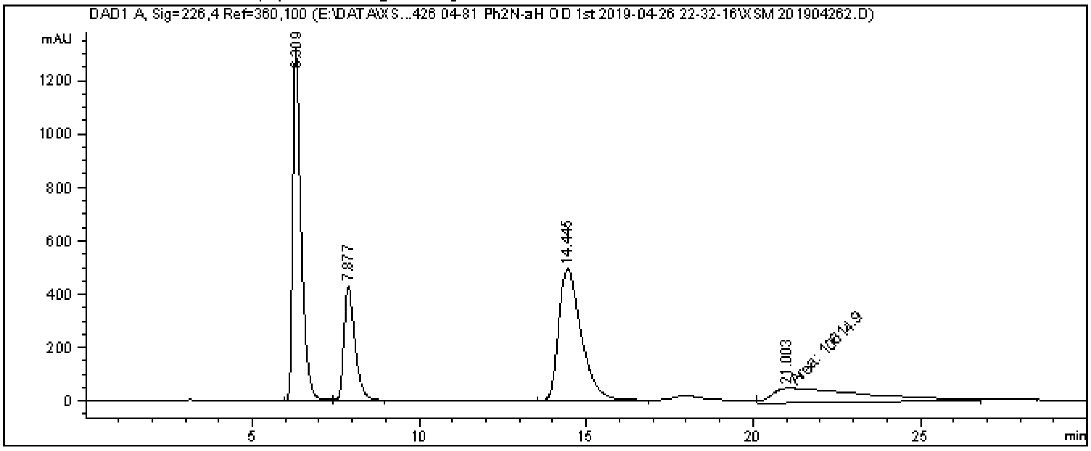

===

Area Percent Report

===

Sorted By : Signal
Multiplier : 1.0000
Dilution : 1.0000
Do not use Multiplier & Dilution Factor with ISTDs

Signal 1: DAD1 A, Sig=226,4 Ref=360,100

Peak #	RetTime [min]	Type	Width [min]	Area [mAU*s]	Height [mAU]	Area %
1	6.309	BV R	0.2607	2.32634e4	1319.42493	34.7684
2	7.877	VB	0.3342	9833.45313	429.37863	14.6966
3	14.445	BV R	0.6574	2.31979e4	494.38535	34.6705
4	21.003	MM	3.1997	1.06149e4	55.29118	15.8645

Totals : 6.69097e4 2298.48008

===

*** End of Report ***

1260 6/7/2019 4:07:09 PM SYSTEM Page 1 of 1

Supplementary Figure 204. HPLC spectrum of (2*R**,3*S**)-**9** and (2*R**,3*R**)-**9**

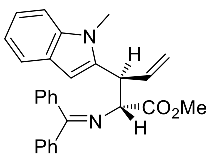

(2*R*,3*S*)-**9**

```
Data File E:\DATA\XSM\In33 190426 04-81 Ph2N-aH OD 1st 2019-04-26 22-32-16\XSM 20190426.D
Sample Name: In33 04-78 Ph2N-aH S+D opt

    =====================================================================
    Acq. Operator   : SYSTEM                          Seq. Line :   1
    Acq. Instrument : 1260                             Location :   78
    Injection Date  : 4/26/2019 10:33:43 PM                 Inj :   1
                                                     Inj Volume : 5.000 µl
    Acq. Method     : E:\DATA\XSM\In33 190426 04-81 Ph2N-aH OD 1st 2019-04-26 22-32-16\In33 OD-98
                      -1mL-226nm-30min Ph2N-aH 1st.M
    Last changed    : 4/26/2019 10:32:16 PM by SYSTEM
    Analysis Method : E:\DATA\XSM\In33 190426 04-81 Ph2N-aH OD 1st 2019-04-26 22-32-16\In33 OD-98
                      -1mL-226nm-30min Ph2N-aH 1st.M (Sequence Method)
    Last changed    : 6/7/2019 4:07:04 PM by SYSTEM
                      (modified after loading)
    Additional Info : Peak(s) manually integrated
```

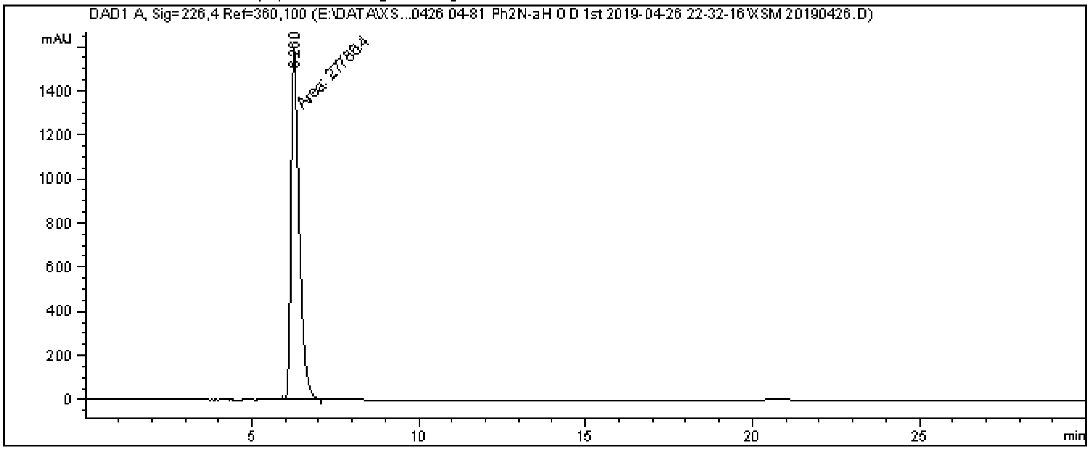


```
    =====================================================================
                         Area Percent Report
    =====================================================================

    Sorted By             :      Signal
    Multiplier            :      1.0000
    Dilution              :      1.0000
    Do not use Multiplier & Dilution Factor with ISTDs


    Signal 1: DAD1 A, Sig=226,4 Ref=360,100

    Peak RetTime Type  Width     Area      Height     Area
      #   [min]        [min]   [mAU*s]     [mAU]        %
    ----|-------|----|-------|----------|----------|--------|
       1   6.260 MF    0.2912 2.77864e4  1590.33875 100.0000

    Totals :                  2.77864e4  1590.33875


    =====================================================================
                          *** End of Report ***
```

1260 6/7/2019 4:08:00 PM SYSTEM Page 1 of 1

Supplementary Figure 205. HPLC spectrum of (2*R*,3*S*)-**9**

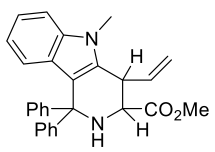

($3R^*,4S^*$)-**10** and ($3R^*,4R^*$)-**10**

Data File E:\DATA\XSM\In33 190511 04-94 Ph2N-aH P-S 2019-05-14 20-39-23\XSM 201905141.D
Sample Name: In33 04-104 Ph2N-aH P-S rac

===

Acq. Operator : SYSTEM   Seq. Line : 2
Acq. Instrument : 1260   Location : 80
Injection Date : 5/14/2019 9:07:24 PM   Inj : 1
Inj Volume : 5.000 µl
Acq. Method : E:\DATA\XSM\In33 190511 04-94 Ph2N-aH P-S 2019-05-14 20-39-23\In33 AD-98-25min-226nm Ph2N-aH P-S.M
Last changed : 5/14/2019 8:39:23 PM by SYSTEM
Analysis Method : E:\DATA\XSM\In33 190511 04-94 Ph2N-aH P-S 2019-05-14 20-39-23\In33 AD-98-25min-226nm Ph2N-aH P-S.M (Sequence Method)
Last changed : 5/25/2019 12:17:14 PM by SYSTEM
(modified after loading)
Additional Info : Peak(s) manually integrated

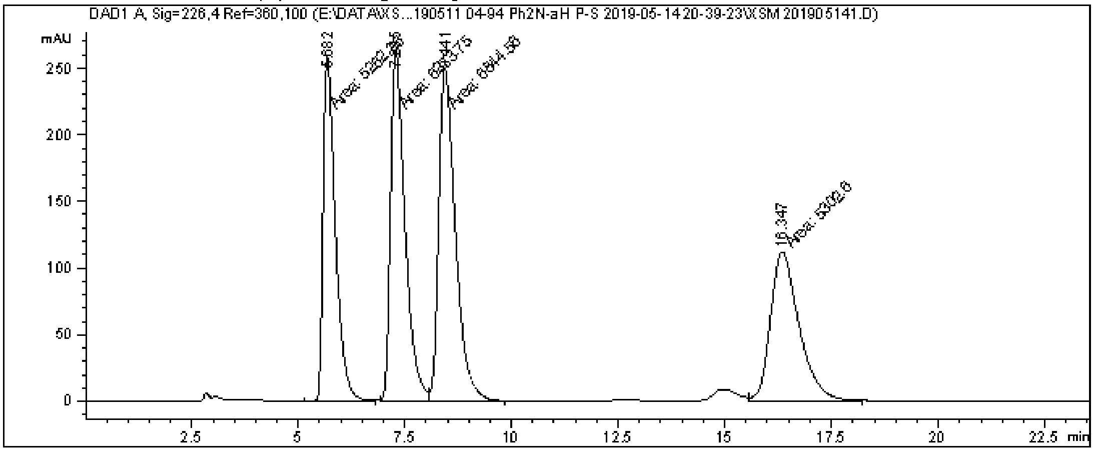

===

Area Percent Report

===

Sorted By : Signal
Multiplier : 1.0000
Dilution : 1.0000
Do not use Multiplier & Dilution Factor with ISTDs

Signal 1: DAD1 A, Sig=226,4 Ref=360,100

Peak #	RetTime [min]	Type	Width [min]	Area [mAU*s]	Height [mAU]	Area %
1	5.682	MM	0.3384	5262.25977	259.18512	22.2194
2	7.285	MF	0.3942	6273.75098	265.25311	26.4903
3	8.441	FM	0.4560	6844.55518	250.16869	28.9005
4	16.347	MM	0.7851	5302.60205	112.56172	22.3897
Totals :				2.36832e4	887.16864	

===

*** End of Report ***

1260 5/25/2019 12:17:19 PM SYSTEM   Page 1 of 1

Supplementary Figure 206. HPLC spectrum of ($3R^*,4S^*$)-**10** and ($3R^*,4R^*$)-**10**

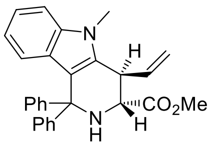

(3*R*,4*S*)-**10**

Data File E:\DATA\XS...90615 04-125 Ph2N-aH P-S S&D opt 2019-06-15 20-14-08\XSM 20190615.D
Sample Name: In33 04-125 Ph2N-aH S+D P-S opt

===

Acq. Operator : SYSTEM Seq. Line : 1
Acq. Instrument : 1260 Location : 78
Injection Date : 6/15/2019 8:15:40 PM Inj : 1
Inj Volume : 5.000 µl
Acq. Method : E:\DATA\XSM\In33 190615 04-125 Ph2N-aH P-S S&D opt 2019-06-15 20-14-08\In33 AD-98-25min-226nm Ph2N-aH P-S.M
Last changed : 6/15/2019 8:14:08 PM by SYSTEM
Analysis Method : E:\DATA\XSM\In33 190615 04-125 Ph2N-aH P-S S&D opt 2019-06-15 20-14-08\In33 AD-98-25min-226nm Ph2N-aH P-S.M (Sequence Method)
Last changed : 6/17/2019 11:04:31 PM by SYSTEM
(modified after loading)
Additional Info : Peak(s) manually integrated

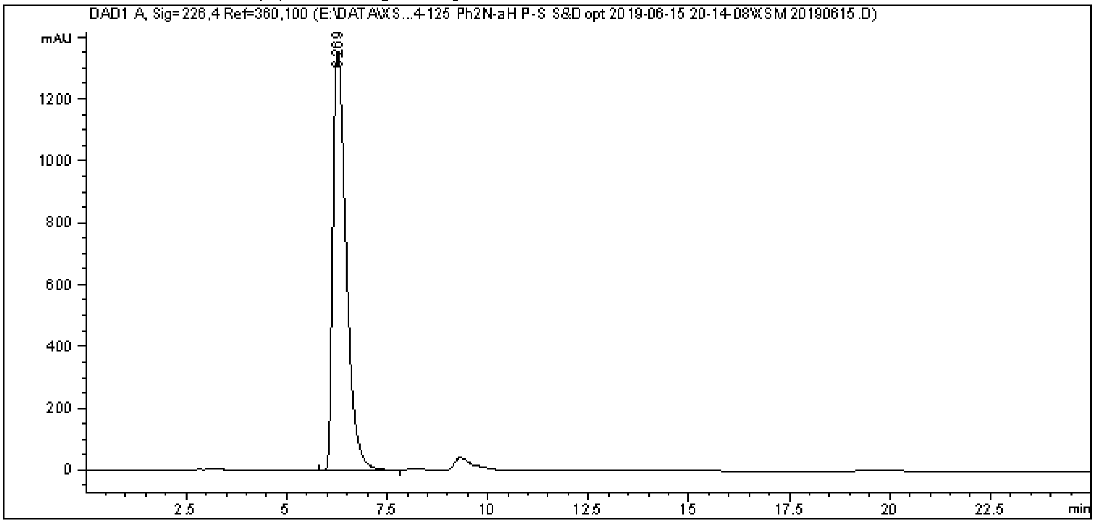

===

Area Percent Report

===

Sorted By : Signal
Multiplier : 1.0000
Dilution : 1.0000
Do not use Multiplier & Dilution Factor with ISTDs

Signal 1: DAD1 A, Sig=226,4 Ref=360,100

Peak #	RetTime [min]	Type	Width [min]	Area [mAU*s]	Height [mAU]	Area %
1	6.269	BB	0.3562	3.10651e4	1351.41418	100.0000

Totals : 3.10651e4 1351.41418

===

*** End of Report ***

1260 6/17/2019 11:04:34 PM SYSTEM Page 1 of 1

Supplementary Figure 207. HPLC spectrum of (3*R*,4*S*)-**10**

Supplementary References

1. Wang, C. J., Liang, G., Xue, Z. Y. & Gao, F. Highly enantioselective 1,3-dipolar cycloaddition of azomethine ylides catalyzed by Copper(I)/TF-BiphamPhos complexes. *J. Am. Chem. Soc*. **130**, 17250-17251 (2008).

2. Sha, F., Tao, Y., Tang, C. Y., Zhang, F. & Wu, X. Y. Construction of benzo[*c*]carbazoles and their antitumor derivatives through the Diels–Alder reaction of 2-alkenylindoles and arynes. *J. Org. Chem*. **80**, 8122-8133 (2015).

3. Richards, C. J. & Mulvaney, A. W. Synthesis of phosphinoferrocenyloxazolines. New ligands for asymmetric catalysis. *Tetrahedron: Asymmetry* **7**, 1419-1430 (1996).

4. Smith, C. R., Mans, D. J. & Rajanbabu, T. V. (*R*)-2,2'-Binaphthoyl-(*S,S*)-di(1-phenylethyl) aminophosphine. Scalable protocols for the syntheses of phosphoramidite (Feringa) ligands. *Org. Synth.* **85**, 238-247 (2008).

5. Spangenberg, T., Schoenfelder, A., Breit, B. & Mann, A. A new method for the synthesis of chiral β-branched α-amino acids. *Org. Lett.* **9**, 3881-3884 (2007).

6. Huang, L., Dai, L.-X. & You, S.-L. Enantioselective synthesis of indole-annulated medium-sized rings. *J. Am. Chem. Soc.* **138**, 5793-5796 (2016).

7. Yamamoto, Y., Fujikawa, R., Umemoto, T. & Miyaura, N. Iridium-catalyzed hydroboration of alkenes with pinacolborane. *Tetrahedron* **60**, 10695-10700 (2004).
